# The efficacy and safety of omalizumab in the treatment of asthma: an overview of systematic reviews and meta analyses

**DOI:** 10.3389/fmed.2025.1755023

**Published:** 2026-01-16

**Authors:** Dongze Li, Zhuang Wang, Lijie Hou, Na Wang, Bing Tian, Xiaofei Xie, Yinan Guo, Yongji Wang

**Affiliations:** 1 College of Traditional Chinese Medicine, Changchun University of Chinese Medicine, Changchun, Jilin, China; 2 Department of Pediatric Internal Medicine One, Affiliated Hospital of Changchun University of Chinese Medicine, Changchun, Jilin, China

**Keywords:** bronchial asthma, efficacy, omalizumab, overview of systematic reviews and meta analyses, safety

## Abstract

**Introduction:**

Omalizumab, an anti-IgE monoclonal antibody, is an established biologic for asthma. This overview synthesized evidence from systematic reviews on its efficacy and safety.

**Methods:**

We systematically searched multiple electronic databases from inception to July 19, 2025. The methodological quality, risk of bias, reporting quality, and certainty of evidence of the included systematic reviews were assessed using the AMSTAR-2, ROBIS, PRISMA 2020, and GRADE tools, respectively. The degree of overlap among primary studies across reviews was measured using the Corrected Covered Area (CCA).

**Results:**

Nineteen studies were included. The CCA calculation indicated a moderate level of overlap (CCA ≈ 0.1421). Most demonstrated low risk of bias, high methodological/reporting quality, and provided moderate-to-high certainty evidence. Omalizumab significantly reduced asthma exacerbation rates and improved overall treatment effectiveness, asthma control, and quality of life. It also facilitated a reduction in daily corticosteroid use, with a confirmed favorable safety profile.

**Conclusion:**

This overview confirms that omalizumab is an effective and safe add-on therapy for asthma. Its mechanism of neutralizing free IgE translates into significant clinical benefits, including reduced exacerbations and improved patient outcomes, establishing it as a valuable therapeutic option.

**Systematic review registration:**

https://www.crd.york.ac.uk/PROSPERO/, CRD420251107860.

## 1 Introduction

Bronchial asthma is a chronic airway disease with significant heterogeneity (1), whose core pathological mechanism involves chronic immune-inflammatory responses mediated by various inflammatory cells such as eosinophils, mast cells, and T lymphocytes, along with their released effector mediators (2, 3), leading to the characteristic pathological triad of airway hyperresponsiveness, persistent inflammatory infiltration, and airway structural remodeling (1, 4). The disease is characterized by recurrent episodes of wheezing, breathlessness, chest tightness, or cough, which are typically triggered by allergens, cold air, physicochemical stimuli, or respiratory infections, and exhibit a distinct circadian rhythm with nocturnal and early morning exacerbations (5–7). The current standardized treatment for asthma primarily follows a stepwise strategy combining inhaled corticosteroid (ICS) and long-acting *β*₂-agonists. However, clinical observations reveal that some patients with refractory asthma exhibit a persistent eosinophilic inflammatory phenotype, maintaining poor symptom control and acute exacerbation risks even under optimized treatment (8–10). This therapeutic dilemma underscores the urgency and clinical importance of developing and applying targeted biologics against specific inflammatory pathways (10, 11).

Omalizumab, a humanized monoclonal antibody targeting IgE (12), effectively inhibits the Th2-type inflammatory cascade mediated by the IgE-FcεRI-mast cell/eosinophil axis by neutralizing free IgE with high affinity and blocking its interaction with FcεRI (13–15), thereby establishing a precision immune-targeted therapy system for patients with moderate-to-severe allergic asthma. This therapy has demonstrated significant clinical benefits in multiple clinical trials (16–21). Randomized controlled trials (RCTs) are regarded as the gold standard in the evidence hierarchy of evidence-based medicine, while systematic reviews/meta-analyses (SRs/MAs) derived from RCTs provide higher-level evidence. However, current SRs/MAs on omalizumab for the treatment of bronchial asthma remain fragmented, and this dispersion severely limits the quality of evidence for clinical decision-makers to develop standardized treatment guidelines. Therefore, this study aims to conduct an overview of SRs and MAs to establish an evidence-based decision-making framework for omalizumab treatment, providing high-level evidence for clinical practice that integrates scientific rigor with practical applicability.

## 2 Materials and methods

### 2.1 Protocol registration

The protocol of this study was prospectively registered on PROSPERO (registration number: CRD420251107860) prior to study initiation. The registration was completed before the literature search implementation, and the actual search strategy, study inclusion criteria, and statistical analysis methods strictly adhered to the pre-registered study protocol.

### 2.2 Inclusion criteria

① The main intervention in the RCTs was omalizumab monotherapy or combination therapy, and the study subjects were limited to patients with bronchial asthma. ② The experimental group received omalizumab-based treatment, while the control group received placebo or standard of care (SOC). ③ The study population consisted of bronchial asthma patients, with no restrictions on age, gender, or region. ④ The included studies were RCTs. ⑤ Participants and/or physicians were unaware of the treatment allocation (blinded design: single-blind or double-blind). ⑥ Participants were randomly assigned to the omalizumab treatment group or the placebo/SOC group.

### 2.3 Exclusion criteria

① Duplicate literature. ② Literature with unavailable full text or incomplete data. ③ Review articles, commentaries, or conference abstracts. ④ Literature deviating from the research theme. ⑤ Studies where the primary treated disease was not bronchial asthma. ⑥ Studies where the treatment group did not receive omalizumab or omalizumab combined with conventional therapy, and/or the control group did not receive placebo or SOC.

### 2.4 Search strategy

We systematically searched eight electronic databases, including PubMed, Embase, Cochrane Library, Web of Science, CNKI, VIP, WANFANG, and CBM, from their inception to July 19, 2025, to identify all SRs and MAs on omalizumab for the treatment of bronchial asthma. Basic Search Logic: Omalizumab AND Asthma AND (Systematic Review OR Meta-analysis). Complete and reproducible search strategies for each database are detailed in Appendix 1.

### 2.5 Article screening and data extraction

This study adopted a dual-independent literature screening model. Two researchers systematically trained in methodology independently conducted the literature search in designated databases according to a predefined search strategy. After the search, the researchers employed a cross-verification method to independently screen and check the retrieved results. In case of discrepancies during the screening process, a third researcher with senior professional title and extensive experience in evidence-based medicine was consulted for arbitration to ensure the objectivity and accuracy of the literature screening. After finalizing the included literature through the aforementioned process, the research team systematically collected the following key information using a standardized data extraction form: ① basic study characteristics, ② methodological features, ③ intervention protocols, ④ quality assessment elements, ⑤ primary outcome measures, and ⑥ study conclusions. The entire data extraction process was conducted through dual independent back-to-back operations with consistency verification to ensure accuracy and completeness of the collected data.

### 2.6 Extraction of repetition rate

Given that SRs and MAs were characterized by the comprehensive retrieval and synthesis of existing literature, the same primary study was frequently included in multiple SRs/MAs. This led to a risk of bias due to double-counting of data. To address this, an overlap matrix between SRs/MAs and the included primary studies was constructed, and the Corrected Covered Area (CCA) was calculated to quantify the degree of overlap among primary studies across multiple SRs/MAs 16. The formula was as follows: CCA = (n-r)/(rc-r), where n represented the total number of inclusions of primary studies across all SRs/MAs before removing duplicates, r denoted the number of unique primary studies after deduplication, and c was the number of SRs/MAs included in the current analysis. The degree of overlap was interpreted based on the following CCA thresholds: CCA ≤ 5% indicated slight overlap, 5% < CCA ≤ 10% suggested moderate overlap, 10% < CCA ≤ 15% represented high overlap, and CCA > 15% reflected very high overlap. This method provided a quantitative tool for assessing literature overlap across syntheses, which was critical for the appropriate interpretation of evidence in research domains with multiple overlapping reviews.

### 2.7 Quality assessment

#### 2.7.1 Risk of bias assessment

This study employed the Risk of Bias in Systematic Reviews (ROBIS) tool (22) to assess the risk of bias in the included SRs/MAs. The evaluation process consisted of three progressive stages: first, a relevance assessment to determine the alignment of the literature with the research topic; second, a judgment of the risk of bias in the MAs process, focusing on the manifestations and severity of potential biases during implementation; and third, a determination of the overall risk of bias in the MAs by synthesizing information from all stages. The ROBIS tool presets signature core questions at each evaluation stage, targeting key nodes of bias risk, with response options including “yes,” “probably yes,” “no,” “probably no,” and “no information.” Through standardized selection and systematic analysis of these options, the risk of bias in MAs can be clearly categorized as low risk, high risk, or unclear, providing a standardized basis for evaluating the reliability of research conclusions.

#### 2.7.2 Methodological quality assessment

The methodological quality of the included SRs/MAs was assessed using A Measurement Tool to Assess Systematic Reviews 2 (AMSTAR-2) (23, 24), which provides a rigorous and standardized operational framework. This tool consists of 16 structured assessment items, among which items 2, 4, 7, 9, 11, 13, and 15 are defined as critical domains and occupy a central position in quality evaluation. For each item, the evaluation results are categorized into three levels: “compliance,” “non-compliance,” or “partial compliance.” By comprehensively considering the number of flaws in both critical and non-critical items, the methodological quality can be precisely classified into four grades: high, moderate, low, or critically low. This refined hierarchical system provides highly specific and accurate quality assessment criteria for research and practice in related fields, which not only facilitates the scientific evaluation of study reliability and validity from a methodological perspective but also establishes a solid methodological foundation for the interpretation of research findings, the translation of evidence, and decision-making. It holds significant academic value and practical importance for advancing the quality and standardization of research in this field.

#### 2.7.3 The evaluation of report quality

The reporting quality of the included studies in the SRs/MAs was evaluated using the Preferred Reporting Items for Systematic Reviews and Meta-Analyses 2020 (PRISMA 2020) (25, 26). The PRISMA 2020 reporting guidelines consist of seven sections, including title, abstract, introduction, methods, results, discussion, and other information, encompassing 27 items and 42 sub-items. The PRISMA2020 statement evaluates the reporting completeness of each item using a three-level scoring system: fully reported (Y) scores 1 point, partially reported (PY) scores 0.5 points, and not reported (N) scores 0 points, with a maximum score of 42. Based on the total score, the reporting completeness is categorized into three levels: a score of 80% or above (33–42 points) indicates “relatively complete reporting” and is rated as high quality; a score above 60% (25–32 points) indicates “reporting with some deficiencies” and is rated as moderate quality; while a score below 60% (less than 25 points) suggests “relatively serious information omission” and is rated as low quality.

#### 2.7.4 Quality assessment of evidence

The GRADE system (27, 28) was employed to standardize the evidence quality grading of SRs/MAs included in the study. This grading process was not a unidimensional judgment but rather a comprehensive integration of multiple core elements, including inherent limitations of studies (such as design flaws or insufficient sample size), inconsistency among study results, indirectness of evidence, imprecision of effect estimates, and potential publication bias. Through rigorous synthesis and evaluation, the evidence quality was ultimately classified into four levels: “high,” “moderate,” “low,” or “very low.” Additionally, key statistical indicators such as the I^2^ value, 95% confidence intervals, and *p* values of effect estimates were systematically extracted to provide robust quantitative support for the evidence quality assessment. This approach ensured the scientific rigor, objectivity, and methodological soundness of the entire evaluation process, thereby establishing a reliable evidence base for subsequent evidence-based decision-making.

### 2.8 Qualitative analysis

For outcome measures included in the SRs/MAs but not meeting the criteria for quantitative synthesis, a qualitative analysis strategy should be adopted. Specifically, this involves integrating narrative summarization with critical appraisal methods, employing thematic analysis to accurately extract core themes from textual descriptions and graphical interpretations in relevant studies. During this process, it is essential to systematically synthesize the main findings of each study regarding the outcome measures, clearly delineating consensus conclusions and heterogeneous results. Subsequently, based on these analytical outcomes, future research directions should be distilled and proposed to provide comprehensive and in-depth qualitative evidence for subsequent decision-making, ensuring scientific rigor and rationality. This approach not only fully exploits the informational value of non-quantitative outcome measures but also, through systematic organization and in-depth analysis of research findings, offers a more solid theoretical foundation and practical guidance for academic research and clinical application in the field, thereby facilitating the effective translation of research outcomes into real-world practice.

## 3 Results

### 3.1 Literature screening results

**Figure 1:**
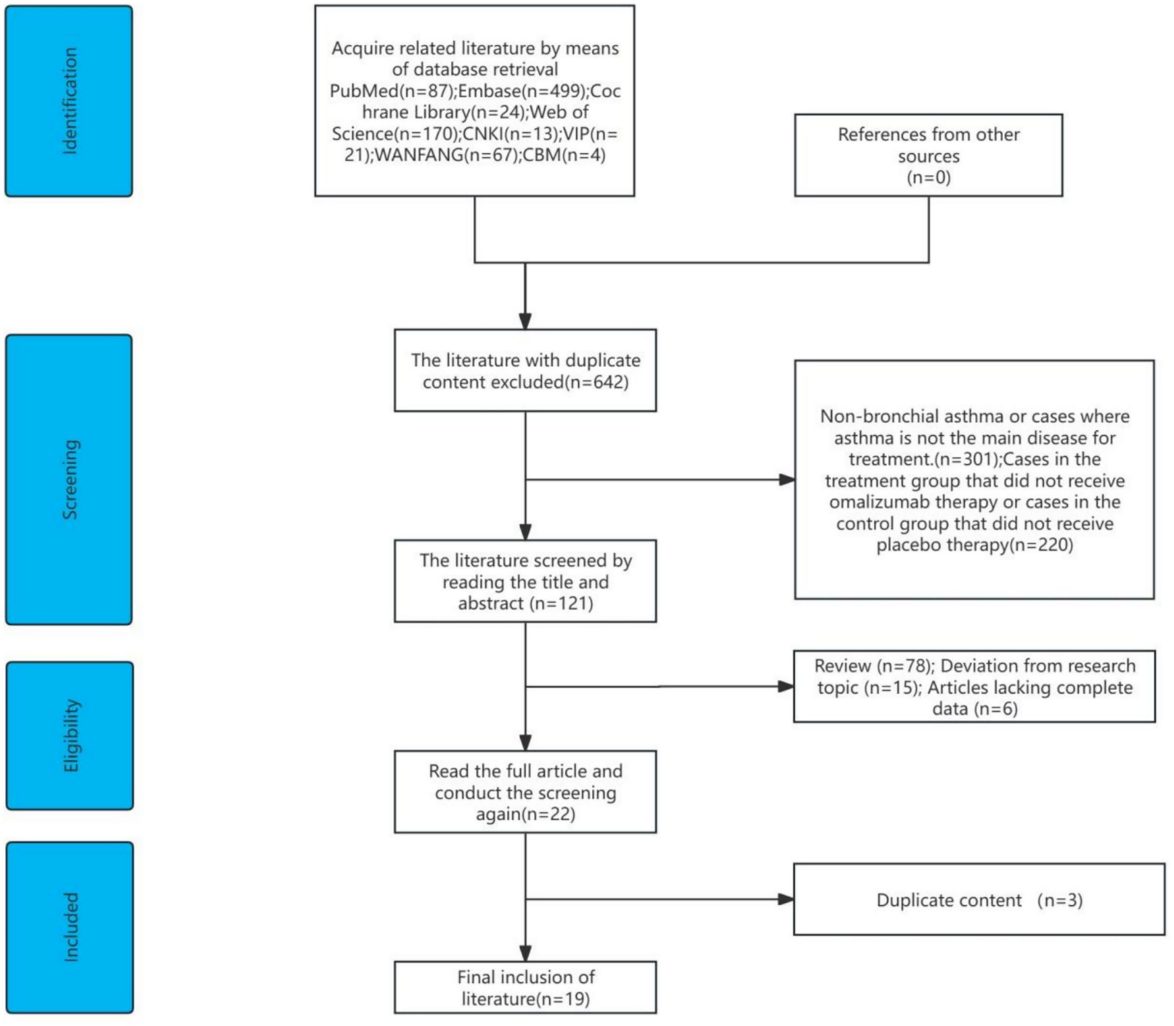
Literature screening process.

The initial search yielded 885 articles, of which 240 duplicates were removed using EndNote X9. After screening titles and abstracts, 304 studies unrelated to bronchial asthma or not primarily focused on asthma treatment were excluded, along with 220 articles with interventions not meeting the inclusion criteria and 78 review articles. Following full-text evaluation, 15 studies with irrelevant research topics, 6 studies lacking complete data, and 3 studies with duplicate content were excluded, resulting in the final inclusion of 19 eligible studies. The screening process is shown in Figure 1.

### 3.2 The basic characteristics of the included literature

A total of 19 studies were included, comprising 12 English and 7 Chinese publications, with 4 being dissertations and the remaining journal articles published between 2013 and 2024. In these studies, the treatment group received Omalizumab plus SOC, while the control group received placebo plus SOC. Regarding outcome measures, 18 studies evaluated asthma acute exacerbation, and 18 reported Asthma Quality of Life Questionnaire (AQLQ) scores. Pulmonary function condition was assessed in 17 studies, while safety profile and adverse events were documented in 16 studies. ICS reduction was examined in 14 studies, and Asthma Control Test (ACT) scores were reported in 9 studies. The Global Evaluation of Treatment Effectiveness (GETE) excellent/good rate was analyzed in 9 studies, while changes in serum total IgE levels were observed in 7 studies. Reduction of rescue medication and rating of asthma treatment effectiveness were each evaluated in 6 studies. Effective rate was reported in 4 studies, study withdrawal status in 3 studies, cost-effectiveness in 2 studies, and some studies investigated the effect of treatment duration, seasonal efficacy, and antiviral role. Regarding methodological quality, 17 studies utilized the Cochrane risk-of-bias assessment tool, one study employed the Jadad scale, and one study conducted a risk-of-bias evaluation without specifying the assessment tool. The baseline characteristics of the included studies are presented in Table 1.

**Table 1:** Characteristics of the included literature.

First author and year	Number of literatures/sample size	Intervention measures		Bias Risk measurement tool	Endpoint measure	The main conclusions of the author
		Treatment group	Control group			
Lin Mai, 2013 (29)	13/4195	Omalizumab + SOC	Placebo+ SOC	Cochrane	①②③④⑤⑥⑦	In the treatment of asthma, omalizumab can decrease the acute exacerbation of asthma and ICS use, and it is safer to improve the therapeutic effects and quality of life.
Changzhi Liu, 2013 (30)	12/4987	Omalizumab + SOC	Placebo+ SOC	Cochrane	①②③④⑤⑥⑦⑧⑨	Data indicate that the efficacy of add-on omalizumab in patients with refractory asthma is accompanied by an acceptable safety profile.
Tianwen Lai, 2015 (31)	6/2749	Omalizumab + SOC	Placebo	Cochrane	①②③④⑤⑥⑦⑧➉	Omalizumab improves quality of life and reduces steroid use in uncontrolled allergic asthma (≥52 weeks), with fewer serious side effects. It may lower exacerbations but lacks strong evidence for refractory cases. Costly but cost-effective in severe asthma (Step 5/6). Limited pediatric data. Future needs: tapering protocols and predictive biomarkers.
Gustavo J. Rodrigo, 2015 (32)	3/1381	Omalizumab + SOC	Placebo+ SOC	Cochrane	①③④⑤⑥⑦⑧⑪	Data indicate that the efficacy of an add-on omalizumab in patients with moderate-to-severe allergic asthma uncontrolled with recommended inhaled steroid treatment is accompanied by an acceptable safety profile.
Weiwei Miao, 2015 (33)	10/5171	Omalizumab + SOC	SOC	Jadad	①③⑤⑥⑦⑫	The addition of omalizumab to standard asthma therapy reduces asthma exacerbations, decreases ICS and rescue medication use, and improves the quality of life in severe asthma patients.
Chunmei Ji, 2016 (34)	15/6260	Omalizumab	Placebo	Cochrane	①③④⑤⑥⑦⑧⑫⑬	This MAs provides high—quality evidence for the efficacy and safety of omalizumab for treating patients with persistent uncontrolled allergic asthma and long-terIll therapy, at least 52 weeks, with omalizumab was recommended due to its advantage of high efficacy and low serious adverse events.
Jonathan Corren, 2018 (35)	26/(N/A)	Omalizumab + SOC	Placebo+ SOC	Cochrane	②⑤⑥⑧⑨	PROs are an integral part of outcome assessment in clinical trials related to asthma. The RCTs reviewed demonstrate that omalizumab treatment improves PROs in patients with moderate-tosevere persistent allergic asthma, particularly symptom control and HRQoL.
Shan Mou, 2019 (36)	12/3971	Omalizumab + SOC	Placebo+ SOC	Cochrane	①②③⑥	The addition of omalizumab to standard therapy in patients with refractory allergic asthma can improve asthma related quality of life, relieve symptoms and reduce severe asthma exacerbations.
Daniel P. Henriksen, 2020 (37)	28/6507	Omalizumab	Placebo/ SOC	Cochrane	①③⑤⑥⑦⑨⑪	Omalizumab provides clinically relevant improvements in exacerbation rate among children, adolescents, and adults and in OCS-reduction among adults. New studies incorporating a guideline-approached definition of severe asthma are warranted.
Zhuo Fu, 2021 (38)	4/1380	Omalizumab + SOC	Placebo+ SOC	Cochrane	①②③⑤⑥⑦⑨⑪⑭	These findings suggested that omalizumab had beneficial effects on moderate-to-severe asthma in children. Patients may benefit more from long-term use of omalizumab. In addition, omalizumab reduces the rate of serious adverse events requiring hospitalizations.
Hongyu Jiang, 2021 (39)	8/1756	Omalizumab + SOC	Placebo+ SOC	Cochrane	①②⑤⑥⑦⑨	Omalizumab can effectively improve the clinical indicators of children with moderate-to-severe allergic asthma, significantly reduce the incidence of severe adverse events, and improve the quality of life of children.
Yaqin Wang, 2021 (40)	4/1678	Omalizumab + SOC	Placebo+ SOC	Cochrane	①⑦⑧➉	Omalizumab reduced rate of asthma exacerbation in children and adolescents, improved patients’ quality of life and was well tolerated.
Xueqin Chen, 2022 (41)	15/6316	Omalizumab + SOC	SOC	Cochrane	①②③④⑤⑥⑦⑨⑫	Omalizumab is an ideal adjunctive treatment for refractory allergic asthma with good efficacy and safety. Further RCTs are needed to determine the appropriate duration of treatment.
Grazia Fenu, 2023 (42)	4/1239	Omalizumab + SOC	Placebo+ SOC	N/A	①③⑤⑥⑦⑮⑯	Our systematic review confirms the known findings that omalizumab therapy decreases asthma exacerbation rate and reduces background therapy inhaled steroid dose. Therefore, add-on therapy with omalizumab shows a good efficacy and safety profile, thus proving to be a useful additional therapeutic option.
Dandan Lang, 2023 (43)	7/2682	Omalizumab + SOC	Placebo+ SOC	Cochrane	①②⑤⑥⑦⑫⑬	In treating IgE (immunoglobulin E)-mediated asthma in children, adding oral (or subcutaneous) omalizumab to a glucocorticoid regimen can enhance the effectiveness of treatment, reduce the probability of significant exacerbation during treatment, and reduce the incidence of serious adverse reactions.
Junwen Ruan, 2023 (44)	11/1956	Omalizumab + SOC	SOC	Cochrane	①⑤⑥⑦⑨⑫⑬	Omalizumab, combined with conventional therapy, improves clinical efficacy, ACT scores, FEV1, and FEV1%Pred in moderate-to-severe asthma patients while reducing IgE, FeNO levels, and asthma exacerbation frequency. However, it does not significantly affect ACQ or AQLQ. Safety-wise, omalizumab reduces adverse reactions (AR), including in patients on ICS + LABA therapy for up to 24 weeks.
Kuankuan Xue, 2023 (45)	12/4527	Omalizumab + SOC	Placebo+ SOC	Cochrane	①③⑤⑥⑦⑫	For moderate-to-severe allergic asthma patients with frequent exacerbations or uncontrolled symptoms despite high-dose ICS + LABA therapy (after ruling out poor compliance, incorrect inhaler use, smoking, comorbidities, or misdiagnosis), omalizumab add-on therapy can provide clinical benefit.
Junyi Liao, 2024 (46)	11/3578	Omalizumab + SOC	Placebo+ SOC	Cochrane	①③⑤⑥⑨	The combination of omalizumab with conventional treatment can improve the lung function of patients with moderate-to-severe asthma to a certain extent.
Xiang Liu, 2024 (47)	9/2101	Omalizumab + SOC	Placebo+ SOC	Cochrane	①③⑤⑥⑦⑨⑫⑬	Omalizumab is more effective than conventional therapy for moderate-to-severe allergic asthma in children, improving asthma control, reducing exacerbations, lowering IgE levels, and enhancing lung/immune function. Safety profiles are similar, but omalizumab has fewer serious adverse events. Early use is recommended when conventional treatment fails to control symptoms.

① Asthma acute exacerbation; ② GETE excellent/good rate; ③ ICS reduction; ④ Reduction of rescue medication; ⑤ Pulmonary function condition; ⑥ AQLQ score; ⑦ Safety profile and adverse events; ⑧ Rating of asthma treatment effectiveness; ⑨ ACT; ⑩ Cost-effectiveness; ⑪ Study withdrawal status; ⑫ Changes in serum total IgE levels; ⑬ Effective rate; ⑭ Effect of treatment duration; ⑮ Seasonal efficacy; ⑯ Antiviral role.

### 3.3 Duplication rate of the original literature

This study included 19 SRs/MAs, collectively referencing 210 primary studies. After deduplication, 51 unique studies remained. The CCA was calculated as (210–51)/(51 × 19–51) ≈ 0.1421, indicating a moderate level of overlap among primary studies across the included SRs/MAs. While this reflects repeated inclusion of certain key studies, all analyses were conducted after rigorous deduplication and methodological adjustments to minimize potential bias, thereby supporting the robustness of the synthesized evidence.

### 3.4 Risk of bias assessment results

All included studies (29–47) were deemed eligible (“passed”) in Phase 1 of the ROBIS assessment. In Phase 2, all studies were judged to be at low risk of bias for the first two domains: study eligibility criteria and identification and selection of studies. However, within the third domain (data collection and study appraisal), one study (30) was assessed as having an undeterminable risk of bias due to incomplete reporting of primary research data and a lack of clearly described verification procedures for data extraction and risk-of-bias assessment in the original studies. Another study (47) in the same domain was rated as high risk of bias, primarily because several of its included primary studies failed to adequately describe randomization or blinding procedures. In the fourth domain (synthesis and findings), one study (35) was judged to have an unclear risk of bias as it did not provide justification for the chosen meta-analysis model, lacked heterogeneity analysis, and did not assess potential publication bias. Despite these specific concerns in individual domains, all studies (29–47) were ultimately evaluated as having a low overall risk of bias in Phase 3 of the ROBIS tool, with no studies being classified as high or unclear risk at this final stage.

### 3.5 Results of methodological quality assessment

Among the included SRs/MAs, 12 studies were rated as high quality (30, 31, 33, 34, 36–38, 41, 43–45, 47), 6 as low quality (29, 32, 35, 40, 42, 46), and 1 as very low quality (39). For the critical items, all 19 studies reported item 4, while the compliance rates for other critical items were as follows: item 11 (18/94.74%), item 9 (18/94.74%), item 13 (14/73.68%), item 7 (12/63.16%), item 15 (9/47.37%), and item 2 (4/21.05%). For non-critical items, all 19 studies reported items 1, 3, and 8, with compliance rates for other non-critical items as follows: item 5 (18/94.74%), item 6 (17/89.47%), item 14 (16/84.21%), item 12 (12/75%), item 16 (10/52.63%), and item 10 (2/10.53%). The detailed methodological quality assessment of the included literature is presented in Table 2.

**Table 2:** Scores for each item of AMSTAR-2.

The included studies	1	2	3	4	5	6	7	8	9	10	11	12	13	14	15	16	Total score
Lin Mai, 2013 (29)	Y	PY	Y	Y	Y	Y	N	Y	Y	N	Y	PY	PY	Y	N	N	10.5
Changzhi Liu, 2013 (30)	Y	PY	Y	Y	Y	Y	PY	Y	Y	N	Y	PY	PY	Y	Y	N	12
Tianwen Lai, 2015 (31)	Y	Y	Y	Y	Y	Y	PY	Y	Y	N	Y	Y	Y	Y	Y	Y	14.5
Gustavo J. Rodrigo, 2015 (32)	Y	PY	Y	Y	Y	Y	Y	Y	Y	Y	Y	Y	Y	Y	N	Y	14.5
Weiwei Miao, 2015 (33)	Y	PY	Y	Y	Y	Y	Y	Y	Y	N	Y	PY	Y	Y	Y	N	12
Chunmei Ji, 2016 (34)	Y	PY	Y	Y	Y	Y	Y	Y	Y	N	Y	Y	Y	Y	Y	Y	14.5
Jonathan Corren, 2018 (35)	Y	PY	Y	Y	Y	Y	Y	Y	Y	N	N	N	Y	PY	N	Y	10
Shan Mou, 2019 (36)	Y	Y	Y	Y	Y	Y	Y	Y	Y	PY	Y	Y	Y	Y	Y	PY	15.5
Daniel P. Henriksen, 2020 (37)	Y	Y	Y	Y	Y	Y	Y	Y	Y	PY	Y	Y	Y	Y	PY	Y	15
Zhuo Fu, 2021 (38)	Y	PY	Y	Y	Y	Y	Y	Y	PY	N	Y	PY	PY	Y	Y	Y	13
Hongyu Jiang, 2021 (39)	Y	N	Y	Y	Y	Y	PY	Y	Y	N	Y	PY	PY	PY	N	N	10
Yaqin Wang, 2021 (40)	Y	PY	Y	Y	Y	Y	PY	Y	Y	N	Y	PY	PY	PY	N	Y	11.5
Xueqin Chen, 2022 (41)	Y	PY	Y	Y	Y	Y	Y	Y	Y	N	Y	Y	Y	Y	Y	Y	14.5
Grazia Fenu, 2023 (42)	Y	Y	Y	Y	PY	PY	Y	Y	Y	Y	Y	Y	Y	Y	N	Y	14
Dandan Lang, 2023 (43)	Y	PY	Y	Y	Y	Y	Y	Y	Y	PY	Y	Y	Y	Y	Y	Y	15
Junwen Ruan, 2023 (44)	Y	PY	Y	Y	Y	PY	Y	Y	Y	N	Y	Y	Y	Y	Y	N	13
Kuankuan Xue, 2023 (45)	Y	PY	Y	Y	Y	Y	Y	Y	Y	N	Y	Y	Y	Y	PY	N	13
Junyi Liao, 2024 (46)	Y	PY	Y	Y	Y	Y	PY	Y	Y	N	Y	Y	Y	Y	N	N	12
Xiang Liu, 2024 (47)	Y	PY	Y	Y	Y	Y	PY	Y	Y	N	Y	Y	Y	Y	PY	N	12.5

Fully reported (Y) scores 1 point, partially reported (PY) scores 0.5 points, and not reported (N) scores 0 points.

### 3.6 Results of quality assessment

**Figure 2:**
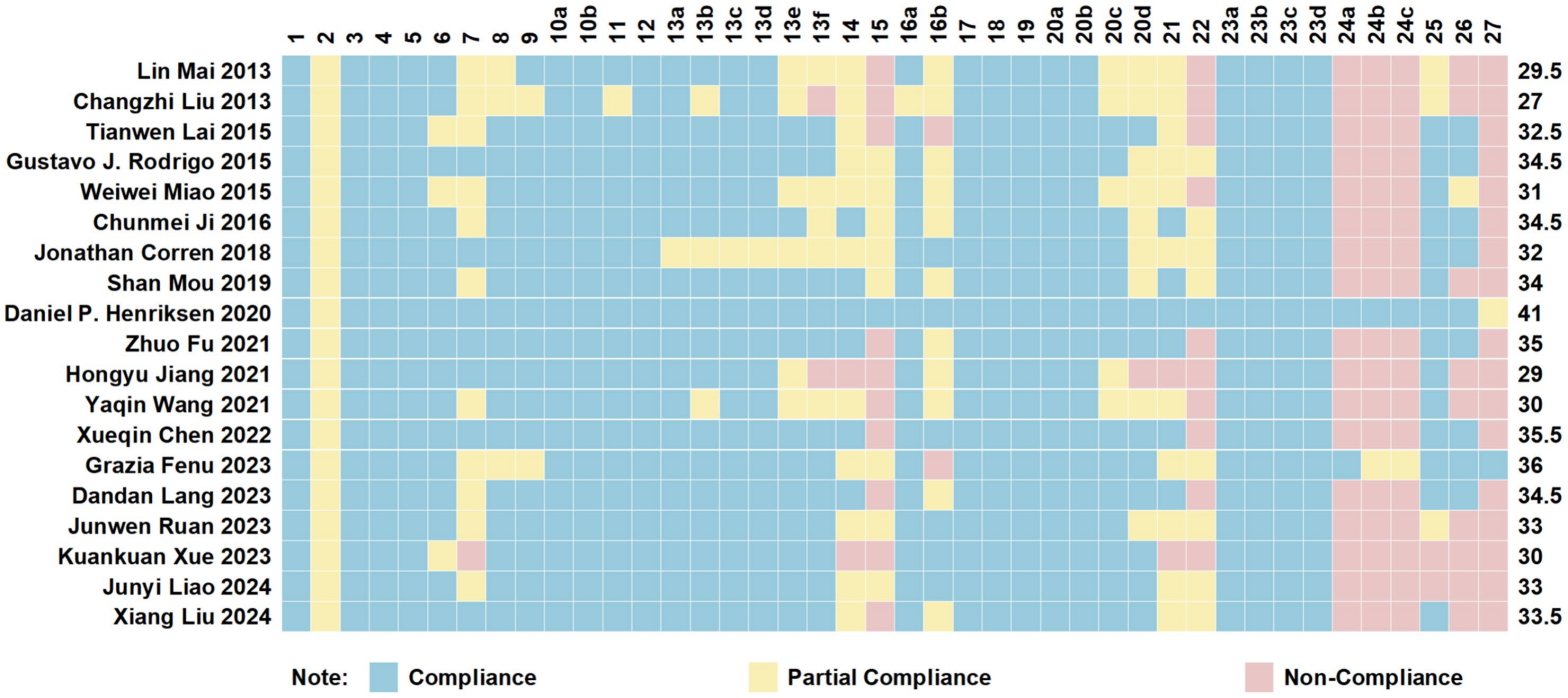
Cartesian heatmap of the scores of each item in PRISMA 2020.

The PRISMA 2020 checklist has a maximum score of 42 points, covering aspects such as the abstract, introduction, methods, results, and discussion, with detailed explanations provided for scoring. The included studies had PRISMA 2020 scores ranging from 27 to 41, with an average score of 32.92. Among them, 11 articles were of high quality (32, 34, 36–38, 41–44, 46, 47), while 8 were of moderate quality (29–31, 33, 35, 39, 40, 45). Specific details are presented in Figure 2.

### 3.7 Results of evidence quality assessment

The GRADE tool was used to evaluate the quality of evidence for the integrated outcome effect measures across the included studies, encompassing a total of 105 integrated effect outcome measures. The results demonstrated that 22 integrated effect outcome measures were rated as high quality, 34 as moderate quality, 25 as low quality, and 24 as extremely low quality. Specific details are presented in Table 3.

**Table 3:** Evidence quality assessment.

The included studies	Endpoint measure	Downgrading factor					Effect size	95%CI	I^2^/%	*p*	Evidence quality
		RB	IC	ID	IP	PB					
Lin Mai, 2013 (29)	Acute exacerbation rate of asthma	−1^①^	0	0	0	−1^⑤^	RR = 0.71	[0.65,0.77]	38	*p* < 0.00001	Low
	GETE excellent/ good rate	−1^①^	−1^②^	0	0	−1^⑤^	RR = 1.61	[1.32,1.97]	65	*p* < 0.00001	Extremely low
	ICS reduction	−1^①^	0	0	0	−1^⑤^	RR = 1.40	[1.29,1.52]	0	*p* < 0.00001	Low
	AQLQ score (Total score improvement ≥ 0.5 points)	−1^①^	0	0	−1^④^	−1^⑤^	RR = 1.61	[1.02,2.53]	87	0.04	Extremely low
	AQLQ score (Total score improvement ≥ 1.5 points)	0	−1^②^	0	−1^④^	−1^⑤^	RR = 2.90	[1.02,8.30]	79	0.05	Extremely low
	Overall adverse reaction rate	−1^①^	0	0	0	0	RR = 1.01	[0.98,1.04]	2	0.53	Medium
	Severe adverse reaction rate	0	0	0	0	0	RR = 0.94	[0.68,1.28]	25	0.68	High
Changzhi Liu, 2013 (30)	Number of asthma exacerbations (stable steroid phase)	−1^①^	−1^②^	0	0	0	RR = 0.64	[0.53,0.78]	59	*p* < 0.00001	Low
	Number of asthma exacerbations (steroid reduction phase)	−1^①^	0	0	0	0	RR = 0.57	[0.48,0.68]	0	*p* < 0.00001	Medium
	Number of patients with complete withdrawal of ICS	−1^①^	−1^②^	0	0	0	RR = 1.80	[1.42,2.28]	54	*p* < 0.00001	Low
	Number of patients with ICS reduction >50%	−1^①^	0	0	0	0	RR = 1.35	[1.26,1.46]	17	*p* < 0.00001	Medium
	Number of patients with GETE rated as “excellent” or “good”	−1^①^	0	0	0	0	RR = 1.43	[1.32,1.55]	0	*p* < 0.00001	Medium
	Dosage of reliever medication (salbutamol)	−1^①^	0	0	0	0	WMD = −0.73	[−1.04,−0.42]	0	*p* < 0.00001	Medium
	Asthma symptom score	−1^①^	0	0	0	0	WMD = −0.46	[−0.63,−0.29]	0	*p* < 0.00001	Medium
	PEF	−1^①^	0	0	−1^④^	0	WMD = 3.56	[−5.05,12.18]	0	0.42	Low
	FEV1	−1^①^	0	0	−1^④^	0	WMD = 68.31	[−23.45,160.07]	0	0.14	Low
	Total adverse reactions	−1^①^	0	0	0	0	RR = 0.99	[0.96,1.02]	27	0.63	Medium
Tianwen Lai, 2015 (31)	Rate of asthma exacerbations	−1^①^	0	0	0	0	RR = 0.63	[0.55,0.71]	0	*p* < 0.00001	Medium
	Complete withdrawal from ICS therapy	−1^①^	0	0	0	0	RR = 1.86	[1.51,2.29]	0	*p* < 0.0001	Medium
	GETE	−1^①^	0	0	0	0	RR = 1.54	[1.38,1.72]	0	*p* < 0.00001	Medium
	AQLQ score	−1^①^	0	0	0	0	RR = 2.08	[1.03,4.20]	0	0.04	Medium
	Adverse events	0	0	0	0	0	RR = 0.97	[0.93,1.01]	3	0.11	High
Gustavo J. Rodrigo, 2015 (32)	Asthma exacerbations	−1^①^	0	0	0	0	RR = 0.69	[0.59,0.80]	0	*p* < 0.00001	Medium
	Asthma symptom score	−1^①^	0	0	0	−1^⑤^	MD = 0.12	[0.04,0.20]	0	0.005	Low
	Pulmonary function	−1^①^	0	0	0	−1^⑤^	SMD = 0.07	[−0.08,0.23]	0	0.36	Low
	Adverse events	−1^①^	0	0	0	0	RR = 1.02	[0.96,1.09]	9	0.50	Medium
Weiwei Miao, 2015 (33)	Rate of exacerbation (Stable-steroid phase)	−1^①^	−1^②^	0	0	0	RR = 0.55	[0.43,0.70]	90	*p* < 0.00001	Low
	Rate of exacerbation (Steroid-reduction phase)	0	0	0	0	0	RR = 0.53	[0.48,0.60]	28	*p* < 0.00001	High
	Dosage of glucocorticoid	0	−1^②^	0	0	0	RR = 1.51	[1.24,1.84]	76	*p* < 0.001	Medium
	Rate of emergency visits	0	−1^②^	0	0	0	RR = 0.63	[0.28,1.44]	96	0.27	Medium
	AQLQ score	0	0	0	0	0	RR = 1.25	[1.13,1.38]	43	*p* < 0.00001	High
Chunmei Ji, 2016 (34)	Asthma exacerbation	0	−1^②^	0	0	0	RR = 0.69	[0.59,0.81]	0	*p* < 0.00001	Medium
	Reduction in medication	0	−1^②^	0	0	0	RR = 1.36	[0.78,2.39]	82	*p* < 0.00001	Medium
	Adverse events	0	0	0	0	0	RR = 1.00	[0.98,1.03]	0	*p* > 0.05	High
	Serious adverse events	0	0	0	0	0	RR = 0.70	[0.49,0.99]	82	0.05	High
Shan Mou, 2019 (36)	severe exacerbations	0	0	0	0	0	RR = 0.51	[0.41,0.64]	0	*p* < 0.00001	High
	Proportion of patients with IGETE rated as “excellent” or “good” (stable steroid dose phase)	0	0	0	0	0	RR = 1.36	[1.25,1.49]	0	*p* < 0.00001	High
	Proportion of patients with IGETE rated as “excellent” or “good”(steroid dose reduction phase)	0	0	0	0	0	RR = 1.48	[1.36,1.61]	0	*p* < 0.00001	High
	Proportion of patients with AQLQ score ≥1.5 (stable steroid dose phase)	0	0	0	0	0	RR = 1.56	[1.32,1.85]	0	*p* < 0.00001	High
	Proportion of patients with AQLQ score ≥1.5 (steroid dose reduction phase)	0	0	0	0	0	RR = 1.90	[1.45,2.49]	0	*p* < 0.00001	High
Daniel P. Henriksen, 2020 (37)	Exacerbation rate	−1^①^	−1^②^	−1^③^	−1^④^	0	RR = 0.63	[0.50,0.79]	60	0.04	Extremely low
	Asthma control	−1^①^	−1^②^	−1^③^	−1^④^	−1^⑤^	SMD = 0.36	[−0.58,−0.13]	85	*p* < 0.0001	Extremely low
	Quality of life (QoL)	−1^①^	−1^②^	−1^③^	−1^④^	−1^⑤^	MD = 0.58	[0.06,0.1.11]	95	*p* < 0.00001	Extremely low
	SAEs	−1^①^	0	−1^③^	−1^④^	0	RR = 0.85	[0.69,1.04]	0	0.77	Extremely low
	Drop-out rate	−1^①^	−1^②^	−1^③^	−1^④^	0	RR = 0.77	[0.59,1.01]	59	0.06	Extremely low
Zhuo Fu, 2021 (38)	Asthma exacerbations rate	−1^①^	0	0	0	0	OR = 0.51	[0.44,0.58]	30.8	*p* < 0.001	Medium
	GETE	−1^①^	0	0	0	0	OR = 2.75	[2.45,3.09]	95.2	*p* < 0.0001	Medium
	Decrease in ICS dose	−1^①^	0	0	0	0	N/A	[−151.19,−64.81]	0	*p* < 0.001	Medium
	Incidence of severe adverse events	−1^①^	0	0	0	0	OR = 0.36	[0.22,0.57]	N/A	*p* < 0.001	Medium
Hongyu Jiang, 2021 (39)	Exacerbation incidence of asthma	−1^①^	0	0	0	−1^⑤^	RR = 0.68	[0.55,0.85]	0	0.0007	Low
	GETE excellent rate	−1^①^	0	0	0	−1^⑤^	RR = 1.39	[1.23,1.58]	0	*p* < 0.00001	Low
	C-ACT score	−1^①^	0	0	0	−1^⑤^	MD = 0.76	[0.31,1.21]	0	0.001	Low
	Forced expiratory volume in 1 s to predicted value, FEV1%	−1^①^	0	0	0	−1^⑤^	MD = 0.62	[−0.89,2.13]	0	0.42	Low
	Forced expiratory volume in 1 s to forced vital capacity, FEV1/FVC	−1^①^	0	0	−1^④^	−1^⑤^	MD = 0.10	[−0.98,0.79]	0	0.83	Extremely low
	Time of absence from school, days	−1^①^	−1^②^	0	0	−1^⑤^	MD = 0.30	[−0.57,−0.02]	91	0.04	Extremely low
	Serious adverse event, SAE incidence	−1^①^	0	0	0	−1^⑤^	RR = 0.54	[0.39,0.76]	41	0.0004	Low
Yaqin Wang, 2021 (40)	Rate of asthma exacerbation	−1^①^	0	0	0	−1^⑤^	RR = 0.63	[0.56,0.71]	31	*p* < 0.00001	Low
Xueqin Chen, 2022 (41)	Asthma exacerbations (stable steroid phase)	0	0	0	0	0	RR = 0.69	[0.63,0.75]	39	*p* < 0.001	High
	Asthma exacerbations (steroid reduction phase)	0	0	0	0	0	RR = 0.55	[0.46,0.66]	41	*p* < 0.001	High
	Emergency visits	0	0	0	0	0	RR = 0.53	[0.38,0.73]	0	*p* < 0.001	High
	ICS reduction >50%	0	0	0	0	0	RR = 1.35	[1.25,1.45]	22	*p* < 0.001	High
	ICS complete withdrawal	0	−1^②^	0	0	0	RR = 1.80	[1.41,2.31]	57	*p* < 0.001	Medium
	AQLQ score improvement >1.5 points	0	0	0	0	0	RR = 1.81	[1.51,2.17]	47	*p* < 0.001	High
	AQLQ score improvement >0.5 points	0	−1^②^	0	0	0	RR = 1.32	[1.13,1.54]	75	*p* < 0.001	Medium
	Use of rescue bronchodilators	−1^①^	0	0	0	0	RR = 0.78	[0.67,0.92]	33	0.003	Medium
	iGETE rated as “excellent” or “good”	−1^①^	−1^②^	0	0	0	RR = 1.56	[1.35,1.81]	78	*p* < 0.001	Low
	Adverse events	−1^①^	0	0	0	0	RR = 1.01	[0.98,1.03]	44	0.640	Medium
Grazia Fenu, 2023 (42)	Asthma exacerbation rate	0	0	0	0	0	OR = 0.44	[0.35,0.56]	N/A	*p* < 0.001	High
	Reduction in ICS use	−1^①^	0	0	0	0	MD = −108	[−151.19,−64.81]	N/A	*p* < 0.01	Medium
Dandan Lang, 2023 (43)	GETE response rate	1^①^	−1^②^	0	0	0	RR = 1.24	[1.09,1.41]	54	0.09	Low
	Incidence of significant clinical exacerbation within 24 weeks	1^①^	−1^②^	0	0	0	RR = 0.55	[0.35,0.85]	86	*p* < 0.001	Low
	Incidence of significant clinical exacerbation within 52 weeks	1^①^	−1^②^	0	0	0	RR = 0.52	[0.39,0.71]	80	*p* < 0.001	Low
	Total incidence of adverse reactions	0	0	0	0	0	RR = 1.00	[0.98,1.03]	0	0.479	High
	Incidence of serious adverse reactions	0	0	0	0	0	RR = 0.53	[0.36,0.77]	0	0.001	High
Junwen Ruan, 2023 (44)	Total clinical effective rate	−1^①^	−1^②^	0	0	0	RR = 1.39	[1.02,1.89]	78	0.04	Low
	ACT	0	0	0	0	0	MD = 3.41	[3.04,3.79]	0	*p* < 0.00001	High
	ACQ	−1^①^	−1^②^	0	−1^④^	0	MD = −0.66	[−1.50,0.17]	93	0.12	Extremely low
	AQLQ	−1^①^	−1^②^	0	−1^④^	−1^⑤^	MD = 0.66	[−0.28,1.60]	89	0.17	Extremely low
	IgE	−1^①^	−1^②^	0	−1^④^	0	MD = −139.79	[−247.64,−31.95]	92	0.01	Extremely low
	FeNO	−1^①^	−1^②^	0	−1^④^	0	MD = −11.81	[−23.30,−0.33]	82	0.04	Extremely low
	FEV1 ncreased value	−1^①^	−1^②^	0	0	0	MD = 0.19	[0.08,0.30]	88	0.0005	Low
	FEV1%Pred increased value	−1^①^	−1^②^	0	−1^④^	0	MD = 6.43	[0.94,11.93]	100	0.02	Extremely low
	Number of acute exacerbation of asthma	0	0	0	0	0	RR = 0.85	[0.75,0.95]	0	0.004	High
	Frequency of acute exacerbation of asthma	0	0	0	−1^④^	0	MD = −0.70	[−1.33,−0.07]	0	0.03	Medium
	Adverse reaction	0	0	0	0	0	RR = 0.84	[0.74,0.97]	0	0.01	High
Kuankuan Xue, 2023 (45)	The proportion of patients with at least one acute asthma attack	−1^①^	−1^②^	0	0	0	RR = 0.76	[0.62,0.93]	86	0.008	Low
	Frequency of acute asthma attacks in patients	−1^①^	−1^②^	0	0	0	WMD = −0.31	[−0.52,−0.10]	96	*p* < 0.0001	Low
	Change in total AQLQ score from baseline during the study period	−1^①^	0	0	0	0	WMD = 0.27	[0.16,0.37]	0	*p* < 0.0001	Medium
	Incidence of adverse reactions in patients during the study period	−1^①^	0	0	0	0	RR = 0.99	[0.95,1.02]	37	0.37	Medium
	Incidence of severe adverse reactions in patients during the study period	−1^①^	0	0	0	0	RR = 0.83	[0.58,1.14]	40	0.14	Medium
Junyi Liao, 2024 (46)	FEV1%	−1^①^	−1^②^	0	0	−1^⑤^	MD = 3.91	[1.89,5.94]	64	0.0002	Extremely low
	mPEF	−1^①^	−1^②^	0	−1^④^	−1^⑤^	MD = 3.64	[−22.17,−29.45]	64	0.78	Extremely low
	FEV1	−1^①^	0	0	0	−1^⑤^	MD = 0.09	[0.05,0.13]	45	*p* < 0.0001	Low
Xiang Liu, 2024 (47)	Overall effective rate of treatment	−1^①^	−1^②^	0	0	−1^⑤^	RR = 1.17	[1.06,1.29]	0	0.002	Low
	Asthma control score	0	0	0	0	−1^⑤^	MD = 3.51	[3.33,3.69]	0	*p* < 0.001	Medium
	Acute asthma attack rate	0	0	0	0	−1^⑤^	RR = 0.53	[0.45,0.63]	0	*p* < 0.001	Medium
	FEV1	−1^①^	−1^②^	0	0	−1^⑤^	MD = 8.65	[6.32,10.97]	0	*p* < 0.001	Extremely low
	FEV1/FVC	−1^①^	−1^②^	0	−1^④^	−1^⑤^	MD = 3.36	[−3.76,10.47]	98	0.35	Extremely low
	IgE	−1^①^	−1^②^	0	0	−1^⑤^	MD = −85.79	[−100.69,−70.89]	51	*p* < 0.001	Extremely low
	CD3	−1^①^	0	0	−1^④^	−1^⑤^	MD = 7.51	[5.19,9.83]	0	*p* < 0.001	Extremely low
	CD4	−1^①^	0	0	−1^④^	−1^⑤^	MD = 5.88	[4.46,7.29]	0	*p* < 0.001	Extremely low
	IgA	−1^①^	0	0	−1^④^	−1^⑤^	MD = 0.98	[0.81,1.15]	0	*p* < 0.001	Extremely low
	IgG	−1^①^	0	0	−1^④^	−1^⑤^	MD = 1.43	[0.70,2.15]	0	*p* < 0.001	Extremely low
	Incidence of adverse reactions	0	0	0	0	−1^⑤^	RR = 0.97	[0.92,1.03]	0	0.29	Medium
	Incidence of serious adverse reactions	0	0	0	0	−1^⑤^	RR = 0.44	[0.29,0.68]	0	0.0002	Medium

① Methodological quality of included studies was low, with biases in randomization, allocation concealment, and blinding; ② The heterogeneity was large and low confidence interval overlap; ③ The population was not broadly representative; ④ Small sample size, 95% confidence intervals include null values; ⑤ Few studies were included, the funnel plot was not symmetrical, Egger’s test found that publication bias or results were positive and there was no publication bias evaluation.

### 3.8 Qualitative analysis

#### 3.8.1 Acute episode rate

Multiple systematic reviews and meta-analyses, comprising 18 studies (29–34, 36–47), have evaluated the effect of omalizumab on acute asthma exacerbations. The aggregated evidence consistently indicates that omalizumab significantly reduces the risk of acute exacerbations. This benefit is observed in both adult and pediatric/adolescent populations with asthma (29, 34, 37). Specifically in children and adolescents, omalizumab significantly reduces the relative risk of acute asthma exacerbations (40). Omalizumab effectively lowers exacerbation risk in patients with refractory or allergic asthma, regardless of whether they are receiving stable maintenance doses or are undergoing tapering of ICS (30, 32, 33, 36, 40, 41). Notably, treatment of longer duration (typically ≥ 30–52 weeks) is associated with a more substantial reduction in exacerbation risk (31, 34, 38, 43). Furthermore, the evidence demonstrates that omalizumab significantly decreases both the proportion of patients experiencing at least one acute exacerbation and the overall exacerbation rate (39, 42, 45). This efficacy extends to specific clinical subgroups, including patients with severe asthma and those receiving add-on therapy to regimens containing ICS combined with a long-acting *β*₂-agonist (LABA) (38, 44). Ultimately, these effects translate into meaningful clinical benefits, including reductions in severe exacerbations, asthma-related emergency department visits and hospitalizations, and the need for systemic corticosteroids (42, 46, 47).

#### 3.8.2 GETE excellent rate

The qualitative analysis of GETE in this study indicated that nine studies demonstrated a clear and consistent positive effect of omalizumab in improving overall treatment effectiveness assessments across different asthma patient populations (29–31, 35, 36, 38, 39, 41, 43). Specifically, the treatment significantly increased the GETE excellent/good response rate in adult patients with allergic bronchial asthma, refractory asthma, and persistent uncontrolled allergic asthma, with robust long-term (≥52 weeks) efficacy observed across different subgroups (29–31). For patients with moderate-to-severe persistent allergic asthma, omalizumab also significantly enhanced their self-assessed (GETE) excellent/good response rate and provided sustained improvement in overall treatment experience (35). Regarding investigator assessment, omalizumab consistently and significantly improved the iGETE excellent/good response rate regardless of whether inhaled corticosteroids were at a stable or tapering dose (36). In pediatric asthma populations, including those with moderate-to-severe or severe asthma and IgE-mediated allergic asthma (38, 39, 43), the treatment similarly led to significant improvements in the investigator’s global evaluation. A meta-analysis further supported that omalizumab significantly increased the iGETE excellent/good response rate, though it noted that heterogeneity in study design might influence the interpretation of results (41).

#### 3.8.3 Adverse events

A comprehensive safety analysis across 14 systematic reviews/meta-analyses found no significant difference in overall adverse reaction rates for omalizumab compared to control groups (conventional therapy or placebo) (29–31, 34, 41, 43, 45–47). Most studies also reported no significant increase in serious adverse events (29, 30, 41, 45), with several indicating a lower incidence versus control (31, 34, 38–40, 43, 47), particularly in pediatric/adolescent patients (35, 38–40, 42, 43) and with long-term treatment (≥52 weeks) (34). Common reactions were typically mild to moderate and self-limiting, including injection-site reactions (Refs. 30, 41, 46), headache (29–31, 34, 41, 42, 45), upper respiratory infections (29–31, 34, 38, 41, 45), sinusitis (29, 34, 38, 41, 45), and influenza-like symptoms (29, 30, 41). Although one study noted increased cardiovascular/cerebrovascular events (33), most analyses showed no significant rise in anaphylaxis (30, 32, 41), malignancy (30, 32, 34, 41), or death (32, 45). Serious events were frequently related to asthma exacerbations and not directly attributed to omalizumab (31, 38). In summary, evidence supports a favorable safety profile for omalizumab in both adult and pediatric/adolescent allergic asthma patients (29–32, 35, 38–47).

#### 3.8.4 Quality of life

A SRs/MAs encompassing 14 studies (29–37, 39, 41, 44, 45, 47) investigated the impact of omalizumab on quality of life in asthma patients, with 8 studies (29, 31, 33, 34, 36, 41, 45, 46) demonstrating significant improvement. In adult patients, the enhancement in AQLQ scores was statistically significant, particularly with long-term treatment (≥52 weeks). Comparative analyses of treatment regimens (31, 36) revealed that omalizumab combined with ICS or ICS + LABA yielded superior quality-of-life benefits compared to conventional therapy. However, studies involving pediatric asthma patients (37, 39) did not show statistically significant improvements in quality of life. Additionally, study (30) confirmed that omalizumab’s efficacy correlated with improved GETE ratings and reduced acute exacerbations, though study (31) suggested its cost-effectiveness might be limited to non-smokers. Notably, studies (32, 44) reported no significant difference in quality-of-life improvement when omalizumab was administered during stable disease or in combination with LABA/ICS + LABA, with study (32) further indicating its inability to prevent childhood asthma onset. Collectively, studies (33, 45, 46) support omalizumab’s significant efficacy in improving quality of life in adults with refractory asthma, though higher-quality evidence is needed to confirm clinical benefits for pediatric patients.

#### 3.8.5 Asthma control status

The existing SRs/MAs, including nine studies (30, 35, 37–39, 41, 44, 46, 47), investigated the effect of omalizumab therapy on asthma control. In adult patients, omalizumab demonstrated a statistically significant improvement in asthma control, though it did not reach the minimal clinically important difference, and the evidence quality was low (37). Among children and adolescents, the results were heterogeneous: some studies (35, 39, 44, 47) reported that omalizumab significantly increased C-ACT/ACT scores, consistent with reductions in acute exacerbations and improvements in lung function, whereas others [30] found no clinically meaningful change in ACT total scores at 24–48 weeks, with only the 4–11-year subgroup showing a statistically significant—but potentially clinically insignificant—difference at 48 weeks. Additionally, when combined with conventional therapy, omalizumab led to more pronounced improvements in ACT scores, and the effect was not influenced by baseline treatment regimens (e.g., ICS/LABA) (44, 46, 47).

#### 3.8.6 Reduction in ICS dosage

Thirteen studies (29–34, 36–38, 41, 42, 46, 47) in the existing SRs/MAs investigated the effect of omalizumab on ICS reduction in asthma patients, all of which (29–34, 36–38, 41, 42, 46, 47) demonstrated that omalizumab significantly facilitated ICS dose reduction. In adult patients, the proportion achieving >50% ICS reduction markedly increased, with more pronounced effects observed during long-term treatment (≥52 weeks) (34, 41). Regarding efficacy across different treatment phases, studies (29–31, 41, 46) indicated that omalizumab significantly reduced ICS dosage during both the steroid reduction and follow-up periods, while also increasing the proportion of patients who completely discontinued ICS. For pediatric asthma patients, studies (37, 38) showed that although omalizumab reduced ICS dose, the clinical relevance did not reach statistical significance. Additionally, research (46, 47) confirmed that the therapeutic effect of omalizumab was associated with reduced acute exacerbations and ICS tapering, with severe steroid-dependent patients achieving a reduction in ICS dose from 12.0 mg/d to 5.0 mg/d. Collectively, the findings (29–34, 41, 46, 47) suggest that omalizumab significantly improves steroid dependency in adults with refractory asthma, though higher-quality evidence is still needed to confirm its clinical benefits in pediatric patients.

## 4 Discussion

### 4.1 Efficacy and safety of omalizumab in the treatment of asthma

In recent years, the emergence of biologics has brought groundbreaking advances in the precision treatment of severe asthma by targeting key cytokines or immune molecules driving airway inflammation, thereby enabling precise modulation of disease progression. In this field, omalizumab, as the first approved anti-IgE monoclonal antibody for asthma treatment (48), exhibits a distinct targeted and upstream regulatory mechanism (49). This drug binds with high specificity to free IgE in circulation at a 1:1 molecular ratio, with its binding site overlapping the FcεRI receptor-binding domain of the IgE molecule, thereby competitively blocking the binding of IgE to FcεRI receptors on mast cells and basophils (50–53). This leads to the downregulation of high-affinity receptor expression on the surface of these effector cells, fundamentally suppressing cell degranulation upon allergen stimulation and reducing the release of inflammatory mediators such as histamine and leukotrienes, thus interrupting the cascade amplification of type I hypersensitivity reactions. At the therapeutic level, the clinical value of omalizumab has been confirmed by multiple high-quality evidence-based medical studies. In patients with moderate-to-severe allergic asthma, the addition of omalizumab to conventional therapy significantly reduces the annual exacerbation rate (54, 55) and demonstrates a notable preventive effect against seasonal allergen-induced acute attacks (56). It effectively improves lung function, with sustained benefits maintained during continued treatment (57). For patients with long-term oral corticosteroid dependence, this agent enables gradual tapering of steroid dosage, with some even achieving complete discontinuation (58), thereby significantly mitigating systemic adverse effects such as osteoporosis and glucose dysregulation associated with prolonged steroid use. Meanwhile, the AQLQ assessment revealed significant improvements in patients’ scores across dimensions such as activity limitation, symptom control, and psychological status (59), which were directly associated with reduced airway inflammatory burden and decreased fear of acute exacerbations. This study demonstrated that omalizumab, in terms of efficacy, when combined with conventional therapy, significantly reduces the acute exacerbation rate in patients with bronchial asthma, improves the GETE excellent response rate, enhances quality of life and asthma control, facilitates the reduction of ICS, and exhibits universal applicability for allergic asthma patients of varying severity levels. In terms of safety, omalizumab has consistently demonstrated a favorable overall tolerability profile in patients (60), with its adverse reaction spectrum closely related to the drug’s mechanism of action and administration route. The most common adverse reactions are local injection site reactions, manifested as mild to moderate redness, swelling, pruritus, or induration (61), which typically occur within hours after injection and are transient (usually <72 h). The incidence of these reactions gradually decreases with prolonged treatment cycles, and treatment interruption is generally unnecessary. Regarding systemic safety, the incidence of severe allergic reactions (e.g., anaphylaxis) is extremely low and mostly occurs after the first dose, underscoring the need for at least 2 h of post-administration observation. There is no evidence suggesting an increased risk of infection, malignancy, or cardiovascular events (62). This study confirms that omalizumab maintains a favorable safety profile, with no additional safety risks observed when added to conventional therapy, supporting its wider clinical adoption.

While this overview supports the overall efficacy and safety of omalizumab in asthma treatment, the certainty of evidence and strength of conclusions vary across different outcome measures and patient populations. For instance, evidence supporting the reduction in acute exacerbations is of higher quality, leading to more robust conclusions; whereas evidence for improvements in quality of life and asthma control scores is limited by heterogeneity or methodological limitations in some studies, necessitating cautious interpretation. Furthermore, responses to omalizumab may differ among children versus adults, across asthma phenotypes, and with varying treatment durations. Clinical decision-making should therefore incorporate individualized assessment based on specific patient characteristics.

### 4.2 Quality assessment

The study, based on a comprehensive evaluation using the ROBIS risk of bias assessment, PRISMA 2020 reporting guidelines, AMSTAR-2 methodological quality criteria, and GRADE evidence grading system, reveals that these SRs/MAs exhibit a cascade of methodological flaws, including insufficient transparency in preregistration (undisclosed PROSPERO registration number/protocol version), absence of ROBIS assessment leading to uncontrolled bias, incomplete conflict-of-interest statements, omission of PRISMA flowcharts undermining study traceability, undisclosed Boolean search strategies across multiple databases reflecting inadequate retrieval rigor, small sample sizes with wide confidence intervals reducing precision of effect estimates, unaddressed heterogeneity (I^2^ > 50%) due to lack of meta-regression/subgroup analysis, and uncorrected publication bias indicated by funnel plot asymmetry—ultimately resulting in systematic downgrading of evidence certainty (GRADE) and compromised external validity. Although the evidence for core outcomes (e.g., exacerbation rate) is highly consistent, issues such as incomplete retrieval and inadequate bias assessment in low- and critically low-quality studies may lead to overestimated conclusions for some secondary outcomes (e.g., seasonal efficacy, antiviral effect), which require careful consideration in clinical practice based on individual patient characteristics.

To address the aforementioned limitations, future researchers conducting SRs should strictly adhere to evidence-based research standards and enhance study quality through multidimensional optimization. During the protocol design phase, prospective registration on the PROSPERO platform (including core elements such as research questions, search strategies, and analysis plans) must be completed, with protocol version numbers clearly labeled to ensure traceability and consistency. The ROBIS tool should be systematically applied for bias risk assessment, identifying potential biases and control measures, with results fully reported. For literature reporting, the PRISMA 2020 guidelines must be rigorously followed: the flow diagram should explicitly specify exclusion reasons at each stage, complemented by transparent inclusion/exclusion criteria to improve reproducibility. Search strategies should disclose Boolean logic for multiple databases and gray literature retrieval paths to ensure verifiability. In statistical analysis, meta-regression or subgroup analysis should be employed to explore sources of heterogeneity when I^2^ > 50%. For imprecise effect estimates due to small samples, merging homogeneous studies to increase sample size, applying random-effects models for weight adjustment, and trial sequential analysis for statistical power assessment are recommended. Contour-enhanced funnel plots and Egger’s regression should be used to evaluate publication bias, with confidence interval widths explicitly noted for interpretation. During evidence synthesis, the GRADE framework should classify “small samples with wide confidence intervals” as a downgrade factor for imprecision and “I^2^ > 50%” as a downgrade factor for inconsistency. Clinical applicability discussions should compare population characteristics between included studies and target populations, analyze intervention adaptability across settings, and cautiously assess external validity to construct clinically translatable evidence.

Methodological rigor, evaluated via AMSTAR-2, ROBIS, PRISMA 2020, and GRADE, critically determines both the validity and the interpretative limitations of this overview’s conclusions. While robust syntheses strongly support omalizumab’s efficacy in reducing exacerbations and its overall safety, identified shortcomings—such as inadequate protocol registration, unaddressed heterogeneity, and insufficient bias assessment—systematically lower the certainty of evidence for specific secondary outcomes. Consequently, while the primary therapeutic benefit is well-substantiated, conclusions regarding the magnitude of effect for nuanced endpoints must be interpreted with caution, reflecting the graded certainty inherent in the underlying evidence base.

Based on the GRADE assessment, this overview provides high or moderate certainty evidence that omalizumab reduces asthma exacerbations, decreases oral corticosteroid use, improves treatment response rates, and demonstrates a favorable safety profile. However, evidence remains low or very low certainty for improvements in certain quality-of-life scores, asthma control in specific subgroups (e.g., children), and changes in some immunological markers, largely due to heterogeneity, small samples, or methodological limitations.

### 4.3 Limitation

This study has several limitation: ① The quantitative analysis revealed a certain degree of heterogeneity among the included studies; however, based on the current clinical evidence, the therapeutic benefits of this intervention demonstrated clear statistical and clinical significance. Although sensitivity and subgroup analyses were systematically conducted to explore potential sources of heterogeneity, the specific causes remain incompletely elucidated. ② The included literature spanned from 2013 to 2024, and due to the constraints of the time period, earlier studies exhibited certain limitations in methodological quality, reporting quality, and evidence quality compared to more recent research, thereby reducing the robustness and reliability of the synthesized evidence across the entire timeframe. ③ The researchers may have introduced some subjectivity during the study process, which could affect the objectivity and accuracy of the evaluation results.

### 4.4 Clinical implications and positioning of omalizumab in the current biologic landscape

Based on the synthesized evidence, omalizumab demonstrates subgroup-specific efficacy profiles. In adults with moderate-to-severe allergic asthma, it consistently reduces exacerbations, improves treatment response, and facilitates ICS reduction—effects enhanced with longer treatment (≥52 weeks). For children/adolescents with allergic asthma, it lowers exacerbation risk with a favorable safety profile, though evidence for quality-of-life and symptom control improvements remains inconsistent. In corticosteroid-dependent severe asthma, it exhibits a clear steroid-sparing effect. These findings support phenotype-driven, individualized treatment approaches.

This overview is primarily based on systematic reviews of RCTs, which validate the efficacy and safety of omalizumab under controlled conditions. However, real-world studies (RWS) provide evidence that more closely reflects clinical practice, revealing the long-term effectiveness and tolerability of the drug in complex and diverse patient populations. For instance, a real-world study with a follow-up period of up to 16 years has demonstrated that omalizumab achieves sustained long-term reductions in exacerbation rates, significant improvements in quality of life and asthma control, and exhibits a persistent steroid-sparing effect, with long-term safety profiles consistent with RCT findings (63). This further supports the long-term benefits of omalizumab in reducing exacerbations, improving quality of life, and decreasing healthcare resource utilization. Future research should further integrate RCT and real-world evidence to comprehensively evaluate the application value of omalizumab across various clinical scenarios.

The evidence synthesized in this overview reinforces omalizumab’s role as a foundational biologic therapy for moderate-to-severe allergic asthma, particularly in patients with elevated IgE and evidence of allergic sensitization. Its mechanism—neutralizing free IgE and disrupting the IgE-FcεRI axis—provides a targeted upstream intervention that translates into consistent reductions in exacerbations, improved symptom control, and enhanced quality of life. In the evolving landscape of asthma biologics, omalizumab occupies a distinct niche: it is often considered a first-line biologic option for allergic phenotypes. It is recommended as an add-on therapy for patients with severe allergic asthma inadequately controlled by high-dose inhaled corticosteroids plus long-acting *β*₂-agonists (64, 65). Notably, the positioning of omalizumab relative to other biologics (e.g., anti-IL-5/IL-5R, anti-IL-4Rα) is increasingly guided by biomarker-driven phenotyping; omalizumab remains the preferred choice for patients with a clear allergic component, whereas eosinophil-targeted agents are reserved for those with prominent eosinophilic inflammation.

## 5 Conclusion

According to currently published SRs/MAs, omalizumab demonstrates significant clinical efficacy in the treatment of bronchial asthma, effectively reducing acute exacerbation rates, improving asthma control, and significantly enhancing patients’ quality of life (AQLQ scores) and overall treatment excellent rates. Omalizumab exhibits a favorable safety profile in asthma management, primarily manifesting as mild to moderate injection-site reactions of short duration, with rare occurrences of severe allergic reactions, supporting its long-term clinical safety. Based on existing evidence, this study further proposes the following inferences: ① Omalizumab may achieve precise regulation of Th2-type inflammation through targeting the IgE-FcεRI axis, with its therapeutic effects demonstrating universality across allergic asthma patients of varying severity; ② Omalizumab may possess a “dual-phase mechanism of action,” characterized by rapid reduction of acute exacerbation risk in the short term and sustained symptom improvement through inhibition of airway remodeling in the long term; ③ Omalizumab may exert a “steroid-sparing effect” in steroid-dependent asthma patients, potentially reducing long-term steroid use; ④ Omalizumab may have potential disease-modifying effects, with early intervention possibly delaying asthma progression. However, the SRs/MAs included in this study exhibited certain heterogeneity in methodological quality, reporting quality, and evidence quality. Some studies demonstrated inherent heterogeneity in results due to the intrinsic characteristics of their original designs, yet these variations precisely reflect the diversity and complexity of research scenarios. Building upon this foundation, our systematic integration and synthesis can still provide valuable reference perspectives for clinical practice and exploration in this field, facilitating further clarification of research directions and core issues. Based on these inferences, future studies may employ multidimensional longitudinal cohort designs to elucidate the temporal biological effects of omalizumab on Th2 inflammatory pathways and its regulatory mechanisms in immune memory remodeling. Subsequent synergistic validation through real-world evidence and RCTs could systematically evaluate the spatiotemporal-specific effects of this targeted therapy on modifying the natural course of asthma. Ultimately, this may lead to establishing precision prediction models based on deep phenotyping stratification, providing translational medical decision-making support for the comprehensive management of allergic asthma.

## Data Availability

The original contributions presented in the study are included in the article/Supplementary material, further inquiries can be directed to the corresponding author.

## Appendix 1

### Database search strategies (using PubMed as an example)

Search date: July 19, 2025.

Search platform: PubMed.

Search terms: ((((((((((((((asthma[MeSH Terms]) OR (asthma[Title/Abstract])) OR (bronchial hyperreactivity[Title/Abstract])) OR (airway remodeling[Title/Abstract])) OR (airway inflammation[Title/Abstract])) OR (respiratory hypersensitivity[Title/Abstract])) OR (allergic asthma[Title/Abstract])) OR (Non-Allergic Asthma[Title/Abstract])) OR (Exercise-Induced Asthma[Title/Abstract])) OR (Occupational Asthma[Title/Abstract])) OR (Severe Asthma[Title/Abstract])) OR (Childhood Asthma[Title/Abstract])) OR (asthmatic[Title/Abstract])) AND (((Omalizumab[MeSH Terms]) OR (Omalizumab[Title/Abstract])) OR (Xolair[Title/Abstract]))) AND (((Meta-Analysis[Publication Type]) OR (Meta-Analysis[Title/Abstract])) OR (Systematic Review[Title/Abstract])).
