# Supplementary material for: The efficacy and safety of omalizumab in the treatment of asthma: an overview of systematic reviews and meta analyses
Source: Front Med (Lausanne). 2026 Jan 16;12:1755023. doi: 10.3389/fmed.2025.1755023 (PMC12855417; doi:10.3389/fmed.2025.1755023)
Supplement: Supplementary file 1 [file Supplementary_file_1.docx]

Research Process and Research Data

Contents

[1.Search Logic 2](#_Toc12639)

[2.Excluded literature and the reasons for exclusion](#_Toc19775) 43

[4.AMSTAR-2 4](#_Toc14575)9

[5.PRISMA 2020 6](#_Toc2063)5

[6.GRADE 1](#_Toc9305)29

**Search Logic**

Retrieval staff 1: Dr. Na Wang

Retrieval staff 2: Dr. Bing Tian

CNKI:13

(SU=哮喘 OR SU=哮喘持续状态 OR SU=支气管哮喘 OR SU=过敏性哮喘 OR SU=运动性哮喘 OR SU=夜间哮喘 OR SU=职业性哮喘 OR SU=难治性哮喘 OR SU=重症哮喘 OR SU=儿童哮喘 OR SU=成人哮喘 OR SU=咳嗽变异性哮喘 OR SU=哮喘儿童 OR SU=哮喘患儿 OR SU=哮喘治疗 OR SU=哮喘疗效 OR SU=哮喘发作 OR SU=哮喘病 OR SU=哮喘控制 OR SU=控制哮喘 OR SU=哮喘急性发作 OR SU=哮喘症状 OR SU=支气管高反应性 OR SU=气道重塑) AND (SU=奥马珠单抗 OR SU=索雷尔 OR SU=抗IgE单抗 OR SU=人源化抗IgE单克隆抗体 OR SU=重组DNA衍生人源化单克隆抗体) AND (SU=meta OR SU=meta分析 OR SU=’meta-analysis’ OR SU=’meta analysis’ OR SU=荟萃分析 OR SU=元分析 OR SU=系统评价)

WANFANG:67

主题:(哮喘 or 哮喘持续状态 or 支气管哮喘 or 过敏性哮喘 or 运动性哮喘 or 夜间哮喘 or 职业性哮喘 or 难治性哮喘 or 重症哮喘 or 儿童哮喘 or 成人哮喘 or 咳嗽变异性哮喘 or 哮喘儿童 or 哮喘患儿 or 哮喘治疗 or 哮喘疗效 or 哮喘发作 or 哮喘病 or 哮喘控制 or 控制哮喘 or 哮喘急性发作 or 哮喘症状 or 支气管高反应性 or 气道重塑) and 主题:(奥马珠单抗 or 索雷尔 or 抗IgE单抗 or 人源化抗IgE单克隆抗体 or 重组DNA衍生人源化单克隆抗体) and 主题:(meta or R“meta分析” or “meta-analysis” or “meta analysis” or 荟萃分析 or 元分析 or 系统评价)

VIP:21

(M=哮喘 OR M=哮喘持续状态 OR M=支气管哮喘 OR M=过敏性哮喘 OR M=运动性哮喘 OR M=夜间哮喘 OR M=职业性哮喘 OR M=难治性哮喘 OR M=重症哮喘 OR M=儿童哮喘 OR M=成人哮喘 OR M=咳嗽变异性哮喘 OR M=哮喘儿童 OR M=哮喘患儿 OR M=哮喘治疗 OR M=哮喘疗效 OR M=哮喘发作 OR M=哮喘病 OR M=哮喘控制 OR M=控制哮喘 OR M=哮喘急性发作 OR M=哮喘症状 OR M=支气管高反应性 OR M=气道重塑) AND (M=奥马珠单抗 OR M=索雷尔 OR M=抗IgE单抗 OR M=人源化抗IgE单克隆抗体 OR M=重组DNA衍生人源化单克隆抗体) AND (M=meta OR R=“meta分析” OR R= “meta-analysis” OR R=“meta analysis” OR R=荟萃分析 OR R=元分析 OR R=系统评价)

CBM:4

(("meta分析"[标题:智能] OR "系统评价"[标题:智能]) OR (("Meta分析"[不加权:扩展] OR "网络Meta分析"[不加权:扩展]) OR "系统评价(主题)"[不加权:扩展])) AND (("奥马珠单抗"[标题:智能] OR "索雷尔"[标题:智能] OR "抗IgE单抗"[标题:智能] OR "人源化抗IgE单克隆抗体"[标题:智能] OR "重组DNA衍生人源化单克隆抗体"[标题:智能]) OR ("奥马珠单抗"[不加权:扩展])) AND (("哮喘控制"[标题:智能] OR "控制哮喘"[标题:智能] OR "哮喘急性发作"[标题:智能] OR "哮喘症状"[标题:智能] OR "支气管高反应性"[标题:智能] OR "气道重塑"[标题:智能]) OR ("儿童哮喘"[标题:智能] OR "成人哮喘"[标题:智能] OR "咳嗽变异性哮喘"[标题:智能] OR "哮喘儿童"[标题:智能] OR "哮喘患儿"[标题:智能] OR "哮喘治疗"[标题:智能] OR "哮喘疗效"[标题:智能] OR "哮喘发作"[标题:智能] OR "哮喘病"[标题:智能]) OR ("哮喘"[标题:智能] OR "哮喘持续状态"[标题:智能] OR "支气管哮喘"[标题:智能] OR "过敏性哮喘"[标题:智能] OR "运动性哮喘"[标题:智能] OR "夜间哮喘"[标题:智能] OR "职业性哮喘"[标题:智能] OR "难治性哮喘"[标题:智能] OR "重症哮喘"[标题:智能]) OR ("哮喘"[不加权:扩展]))

PubMed:87

((((((((((((((asthma[MeSH Terms]) OR (asthma[Title/Abstract])) OR (bronchial hyperreactivity[Title/Abstract])) OR (airway remodeling[Title/Abstract])) OR (airway inflammation[Title/Abstract])) OR (respiratory hypersensitivity[Title/Abstract])) OR (allergic asthma[Title/Abstract])) OR (Non-Allergic Asthma[Title/Abstract])) OR (Exercise-Induced Asthma[Title/Abstract])) OR (Occupational Asthma[Title/Abstract])) OR (Severe Asthma[Title/Abstract])) OR (Childhood Asthma[Title/Abstract])) OR (asthmatic[Title/Abstract])) AND (((Omalizumab[MeSH Terms]) OR (Omalizumab[Title/Abstract])) OR (Xolair[Title/Abstract]))) AND (((Meta-Analysis[Publication Type]) OR (Meta-Analysis[Title/Abstract])) OR (Systematic Review[Title/Abstract]))

Embase:499

#1：'asthma'/exp OR asthma

#2：asthmatic

#3：'bronchial hyperreactivity'

#4：'airway remodeling'

#5：'respiratory hypersensitivity'

#6：'allergic asthma'

#7：'non-allergic asthma'

#8：'exercise-induced asthma'

#9：'occupational asthma'

#10：'severe asthma'

#11：'childhood asthma'

#12：#1 OR #2 OR #3 OR #4 OR #5 OR #6 OR #7 OR #8 OR #9 OR #10 OR #11

#13：'omalizumab'/exp OR omalizumab

#14:xolair

#15:#13 OR #14

#16:'meta analysis'/exp OR 'meta analysis'

#17:'systematic review'/exp OR 'systematic review'

#18:#16 OR #17

#19:#12 AND #15 AND #18

Cochrane Library:24

#1：MeSH descriptor: [Asthma] explode all trees

#2：(asthma):ti,ab,kw OR (bronchial hyperreactivity):ti,ab,kw OR (airway remodeling):ti,ab,kw OR (airway inflammation):ti,ab,kw OR (respiratory hypersensitivity):ti,ab,kw

#3：(allergic asthma):ti,ab,kw OR (Non-Allergic Asthma):ti,ab,kw OR (Exercise-Induced Asthma):ti,ab,kw OR (occupational asthma):ti,ab,kw OR (severe asthma):ti,ab,kw

#4：(asthmatic):ti,ab,kw

#5：#1 OR #2 OR #3 OR #4

#6：MeSH descriptor: [Omalizumab] explode all trees

#7：(Omalizumab):ti,ab,kw OR (Xolair):ti,ab,kw

#8：#6 OR #7

#9：MeSH descriptor: [Meta-Analysis] explode all trees

#10：MeSH descriptor: [Systematic Review] explode all trees

#11：(meta):ti,ab,kw OR (meta analysis):ti,ab,kw OR (meta-analysis):ti,ab,kw OR (systematic review):ti,ab,kw

#12：#9 OR #10 OR #11

#13：#5 AND #8 AND #12

Web of Science:170

#1：(((((((((((TS=(asthma)) OR TS=(bronchial hyperreactivity)) OR TS=(airway remodeling)) OR TS=(airway inflammation)) OR TS=(respiratory hypersensitivity)) OR TS=(allergic asthma)) OR TS=(Non-Allergic Asthma)) OR TS=(Exercise-Induced Asthma)) OR TS=(occupational asthma)) OR TS=(severe asthma)) OR TS=(childhood asthma)) OR TS=(asthmatic)

#2：(TS=(Omalizumab)) OR TS=(Xolair)

#3：(((TS=(meta)) OR TS=(meta analysis)) OR TS=(meta-analysis)) OR TS=(systematic review)

#4：#1 AND #2 AND #3

**Excluded literature and the reasons for exclusion**

Screener 1: Dr. Bing Tian

Screener 2: Dr. Na Wang

**Duplicate literature(n=243)**

1. 'Real-life' effectiveness studies of omalizumab in adult patients with severe allergic asthma: systematic review 2016
2. 'Real-life' effectiveness studies of omalizumab in adult patients with severe allergic asthma: systematic review 2016
3. 'Real-life' effectiveness studies of omalizumab in adult patients with severe allergic asthma: Systematic review 2016
4. Efficacy and safety of treatment with biologicals (benralizumab, dupilumab, mepolizumab, omalizumab and reslizumab) for severe eosinophilic asthma. A systematic review for the EAACI Guidelines - recommendations on the use of biologicals in severe asthma 2020
5. Efficacy and safety of treatment with biologicals (benralizumab, dupilumab, mepolizumab, omalizumab and reslizumab) for severe eosinophilic asthma 2020
6. Efficacy and safety of treatment with biologicals (benralizumab, dupilumab and omalizumab) for severe allergic asthma: A systematic review for the EAACI Guidelines - recommendations on the use of biologicals in severe asthma 2020
7. Efficacy and safety of treatment with biologicals (benralizumab, dupilumab and omalizumab) for severe allergic asthma: A systematic review for the EAACI Guidelines - recommendations on the use of biologicals in severe asthma 2020
8. Adverse events of biological therapy in chronic rhinosinusitis with nasal polyps: A systematic review 2022
9. Adverse events of biological therapy in chronic rhinosinusitis with nasal polyps: A systematic review 2022
10. Meta-analysis of observational studies of the effectiveness of omalizumab in the control of severe allergic asthma 2015
11. "Real-life" Effectiveness Studies of Omalizumab in Adult Patients with Severe Allergic Asthma: Meta-analysis 2017
12. "Real-life" Effectiveness Studies of Omalizumab in Adult Patients with Severe Allergic Asthma: Meta-analysis 2017
13. Economic evaluation of biological treatments in patients with severe asthma: a systematic review 2023
14. Feasibility of matching-adjusted indirect compar ison (MAIC) of omalizumab (OMA) vs. mepolizumab (MEPO) in moderate-to-severe asthma 2016
15. Omalizumab Improves Outcomes in Patients with Chronic Rhinosinusitis with Nasal Polyps Irrespective of Asthma Status 2020
16. Improvement in Smell Using Monoclonal Antibodies Among Patients With Chronic Rhinosinusitis With Nasal Polyps: A Systematic Review 2023
17. Improvement in Smell Using Monoclonal Antibodies Among Patients With Chronic Rhinosinusitis With Nasal Polyps: A Systematic Review 2023
18. Omalizumab in eosinophilic granulomatosis with polyangiitis: friend or foe? A systematic literature review 2020
19. Omalizumab in eosinophilic granulomatosis with polyangiitis: friend or foe? A systematic literature review 2020
20. Magnitude of effect of asthma treatments on Asthma Quality of Life Questionnaire and Asthma Control Questionnaire scores: Systematic review and network meta-analysis 2015
21. Biologics in allergic rhinitis 2023
22. Biologics in allergic rhinitis 2023
23. Gateways to Clinical Trials 2004
24. Management of Pediatric Urticaria with Review of the Literature on Chronic Spontaneous Urticaria in Children 2018
25. Systematic Review of Observational Studies and Rcts of Omalizumab in Severe Persistent Allergic Asthma and Meta-Analysis Feasibility Assessment 2014
26. Systematic review of observational studies and RCTS of omalizumab in severe persistent allergic asthma and meta-analysis feasibility assessment 2014
27. Mepolizumab for Treating Severe Eosinophilic Asthma: an Evidence Review Group Perspective of a NICE Single Technology Appraisal 2018
28. Real-World Effectiveness of Omalizumab in Severe Allergic Asthma: A Meta-Analysis of Observational Studies 2021
29. Real-World Effectiveness of Omalizumab in Severe Allergic Asthma: A Meta-Analysis of Observational Studies 2021
30. Asthma exacerbations and lung function decline in a pooled analysis of adolescents and adults from randomized controlled trials of omalizumab 2017
31. Update on Interventions in Prevention and Treatment of Pediatric Asthma 2018
32. The Impact of Monoclonal Antibodies on Airway Smooth Muscle Contractility in Asthma: A Systematic Review 2021
33. Oral Corticosteroids Dependence and Biologic Drugs in Severe Asthma: Myths or Facts? A Systematic Review of Real-World Evidence 2021
34. Oral Corticosteroids Dependence and Biologic Drugs in Severe Asthma: Myths or Facts? A Systematic Review of Real-World Evidence 2021
35. Monoclonal antibodies in severe asthma: is it worth it? 2019
36. Monoclonal antibodies in severe asthma: is it worth it? 2019
37. Safety of omalizumab in patients with chronic idiopathic/spontaneous urticaria (CIU/CSU): pooled analysis of three randomized, double-blind, placebo-controlled Phase III studies (ASTERIA I, ASTERIA II, and GLACIAL) 2015
38. Principal findings of systematic reviews for chronic treatment in childhood asthma 2015
39. Principal findings of systematic reviews for chronic treatment in childhood asthma 2015
40. Effects of omalizumab in children with asthma A protocol for systematic review and meta-analysis 2021
41. Effects of omalizumab in children with asthma: A protocol for systematic review and meta-analysis 2021
42. Incidence of Anti-Drug Antibodies to Monoclonal Antibodies in Asthma: A Systematic Review and Meta-Analysis 2023
43. Incidence of Anti-Drug Antibodies to Monoclonal Antibodies in Asthma: A Systematic Review and Meta-Analysis 2023
44. Systematic literature review of the epidemiology and clinical burden of chronic rhinosinusitis with nasal polyposis 2020
45. Efficacy and safety of omalizumab in patients with refractory allergic asthma: a meta‑analysis 2022
46. Effectiveness and Safety Studies of Omalizumab in Children and Adolescents With Moderate-To-Severe Asthma 2023
47. Effectiveness and Safety Studies of Omalizumab in Children and Adolescents With Moderate-To-Severe Asthma 2023
48. Efficacy of benralizumab for patients with severe, uncontrolled atopic asthma by serum immunoglobulin e concentrations 2017
49. Efficacy of benralizumab for patients with severe, uncontrolled atopic asthma by serum immunoglobulin e concentrations 2017
50. Efficacy of benralizumab for patients with severe, uncontrolled atopic asthma by serum immunoglobulin e concentrations 2018
51. Biologics for chronic rhinosinusitis 2020
52. Biologics for chronic rhinosinusitis 2020
53. Biologics for chronic rhinosinusitis 2021
54. Biologics for chronic rhinosinusitis 2021
55. Biologics for chronic rhinosinusitis 2020
56. Association Between Serum Total IgE Levels and Clinical Response to Omalizumab for Chronic Spontaneous Urticaria: A Systematic Review and Meta-Analysis 2023
57. Gender bias in clinical trials of biological agents for severe asthma: A systematic review 2021
58. Gender bias in clinical trials of biological agents for severe asthma: A systematic review 2021
59. Comparative effectiveness of mepolizumab and omalizumab in severe asthma: an indirect treatment comparison 2017
60. Comparative effectiveness of mepolizumab and omalizumab in severe asthma: An indirect treatment comparison 2017
61. Omalizumab and long-term quality of life outcomes in patients with moderate-to-severe allergic asthma: a systematic review 2019
62. Omalizumab and long-term quality of life outcomes in patients with moderate-to-severe allergic asthma: a systematic review 2019
63. Efficacy and safety of omalizumab in children and adolescents with moderate-to-severe asthma: A systematic literature review 2017
64. Patient-reported outcomes in moderate-to-severe allergic asthmatics treated with omalizumab: a systematic literature review of randomized controlled trials 2018
65. Patient-reported outcomes in moderate-to-severe allergic asthmatics treated with omalizumab: a systematic literature review of randomized controlled trials 2018
66. A systematic literature review of burden of illness in adults with uncontrolled moderate/severe asthma 2022
67. The effect of biologics in lung function and quality of life of patients with united airways disease: A systematic review 2024
68. Systematic review of models used in economic analyses in moderate-to-severe asthma and COPD 2016
69. Systematic review of models used in economic analyses in moderate-to-severe asthma and COPD 2016
70. 'Real-world' effectiveness of omalizumab in adults with severe allergic asthma: a meta-analysis 2021
71. 'Real-world' effectiveness of omalizumab in adults with severe allergic asthma: a meta-analysis 2021
72. Severe pediatric asthma therapy: Omalizumab-A systematic review and meta-analysis of efficacy and safety profile 2022
73. Severe pediatric asthma therapy: Omalizumab—A systematic review and meta-analysis of efficacy and safety profile 2022
74. Oral immunotherapy for peanut allergy: The con argument 2020
75. Comparing bronchial thermoplasty with biologicals for severe asthma: Systematic review and network meta-analysis 2023
76. Efficacy and safety of omalizumab in children with moderate-to-severe asthma: a meta-analysis 2020
77. Efficacy and safety of omalizumab in children with moderate-to-severe asthma: a meta-analysis 2021
78. Omalizumab may protect allergic patients against COVID-19: A systematic review 2023
79. Choice of instrument impacts healthcare decisions: effect of source of utility derived from the same patient population on cost-effectiveness 2017
80. Efficacy of omalizumab in children, adolescents, and adults with severe allergic asthma: a systematic review, meta-analysis, and call for new trials using current guidelines for assessment of severe asthma 2020
81. Efficacy of omalizumab in children, adolescents, and adults with severe allergic asthma: a systematic review, meta-analysis, and call for new trials using current guidelines for assessment of severe asthma 2020
82. Efficacy of omalizumab, an anti-immunoglobulin E antibody, in patients with allergic asthma at high risk of serious asthma-related morbidity and mortality 2001
83. Efficacy of omalizumab, an anti-immunoglobulin E antibody, in patients with allergic asthma at high risk of serious asthma-related morbidity and mortality 2001
84. Efficacy of omalizumab, an anti-immunoglobulin E antibody, in patients with allergic asthma at high risk of serious asthma-related morbidity and mortality 2001
85. Efficacy of omalizumab, an anti-immunoglobulin E antibody, in patients with allergic asthma at high risk of serious asthma-related morbidity and mortality 2001
86. Omalizumab for atopic dermatitis: case series and a systematic review of the literature 2017
87. Omalizumab for atopic dermatitis: case series and a systematic review of the literature 2017
88. Efficacy of omalizumab in reducing asthma exacerbation in asian patients: a pooled analysis of two randomized placebo-controlled studies 2019
89. Effect of mepolizumab in severe eosinophilic asthma according to omalizumab eligibility 2019
90. Effect of mepolizumab in severe eosinophilic asthma according to omalizumab eligibility 2019
91. The role of biologics in chronic rhinosinusitis: a systematic review 2020
92. The role of biologics in chronic rhinosinusitis: a systematic review 2020
93. Omalizumab in Allergic Bronchopulmonary Aspergillosis: A Systematic Review and Meta-Analysis 2023
94. Omalizumab in Allergic Bronchopulmonary Aspergillosis: A Systematic Review and Meta-Analysis 2023
95. Influence of prolonged treatment with omalizumab on the development of solid epithelial cancer in patients with atopic asthma and chronic idiopathic urticaria: A systematic review and meta-analysis 2019
96. Influence of prolonged treatment with omalizumab on the development of solid epithelial cancer in patients with atopic asthma and chronic idiopathic urticaria: A systematic review and meta-analysis 2019
97. Use of omalizumab for management of idiopathic anaphylaxis A systematic review and retrospective case series 2021
98. Prospects for new and emerging therapeutics in severe asthma: the role of biologics 2017
99. Asthma Cost-Effectiveness Analyses: Are We Using the Recommended Outcomes in Estimating Value? 2018
100. Pharmacoeconomic analysis of treatment of adult patients with severe uncontrolled asthma with omalizumab in Russia 2016
101. Pharmacoeconomic analysis of treatment of adult patients with severe uncontrolled asthma with omalizumab in Russia 2016
102. Pharmacoeconomic analysis of treatment of children with severe uncontrolled asthma with omalizumab in Russia 2016
103. Efficacy of Biologics in Severe, Uncontrolled Asthma Stratified by Blood Eosinophil Count: A Systematic Review 2023
104. ECONOMIC ASPECTS OF APPLICATION OF THE RUSSIAN BIOSIMILAR OMALIZUMAB IN PATIENTS WITH ATOPIC BRONCHIAL ASTHMA OF MODERATE TO SEVERE CLINICAL COURSES 2021
105. Delayed Pressure Urticaria: A Systematic Review of Treatment Options 2020
106. Symptomatic Dermographism: A Systematic Review of Treatment Options 2020
107. Biologic agents licensed for severe asthma: a systematic review and meta-analysis of randomised controlled trials 2024
108. Long-term efficacy and safety of omalizumab in patients with persistent uncontrolled allergic asthma: a systematic review and meta-analysis 2015
109. Long-term efficacy and safety of omalizumab in patients with persistent uncontrolled allergic asthma: a systematic review and meta-analysis 2015
110. Long-term efficacy and safety of omalizumab in patients with persistent uncontrolled allergic asthma: a systematic review and meta-analysis 2015
111. Safety and Tolerability of Omalizumab in Children with Allergic (IgE-Mediated) Asthma: A Systematic Review and Meta-Analysis 2023
112. Real-world Effectiveness of Mepolizumab in Severe Eosinophilic Asthma: A Systematic Review and Meta-analysis 2021
113. Beneficial effects of Omalizumab therapy in allergic bronchopulmonary aspergillosis: a synthesis review of published literature 2017
114. Predictive biomarkers for response to omalizumab in patients with severe allergic asthma: a meta-analysis 2022
115. Predictive biomarkers for response to omalizumab in patients with severe allergic asthma: a meta-analysis 2022
116. Effects of omalizumab on lung function in patients with moderate-to-severe allergic asthma: a systematic review and meta-analysis 2024
117. Effects of omalizumab on lung function in patients with moderate-to-severe allergic asthma: a systematic review and meta-analysis 2024
118. INVESTIGATING THE VALUE OF OMALIZUMAB IN THE TREATMENT OF SEVERE PERSISTENT ALLERGIC ASTHMA: A SYSTEMATIC REVIEW OF COST-EFFECTIVENESS STUDIES 2016
119. Advanced Biologic Therapies in the Management of Asthma in Children and Adolescents: A Comprehensive Network Meta-Analysis 2024
120. Advanced Biologic Therapies in the Management of Asthma in Children and Adolescents: A Comprehensive Network Meta-Analysis 2024
121. Adverse Events for Monoclonal Antibodies in Patients with Allergic Rhinitis: A Systematic Review and Meta-Analysis of Randomized Clinical Trials 2023
122. Head-To-Head Comparison of Biologic Efficacy in Asthma: What Have We Learned? 2025
123. Head-To-Head Comparison of Biologic Efficacy in Asthma: What Have We Learned? 2025
124. Efficacy and Safety of Omalizumab for the Treatment of Severe or Poorly Controlled Allergic Diseases in Children: A Systematic Review and Meta-Analysis 2022
125. Efficacy and Safety of Omalizumab for the Treatment of Severe or Poorly Controlled Allergic Diseases in Children: A Systematic Review and Meta-Analysis 2022
126. The clinical and pathological histology efficacy of biological therapy for severe asthma with a phenotype of type 2 inflammation - systematic review 2025
127. Short- and long-term real-world effectiveness of omalizumab in severe allergic asthma: systematic review of 42 studies published 2008-2018 2019
128. Short- and long-term real-world effectiveness of omalizumab in severe allergic asthma: systematic review of 42 studies published 2008-2018 2019
129. Effects of Therapeutic Antibodies on Gene and Protein Signatures in Asthma Patients: A Comparative Systematic Review 2022
130. Oral immunotherapy for food allergy: A Spanish guideline. Immunotherapy egg and milk Spanish guide (items guide). Part I: Cow milk and egg oral immunotherapy: Introduction, methodology, rationale, current state, indications contraindications and oral immunotherapy build-up phase 2017
131. Efficacy and safety of omalizumab in paediatric age: an update of literature data 2016
132. EFFICACY AND SAFETY OF OMALIZUMAB IN PAEDIATRIC AGE: AN UPDATE OF LITERATURE DATA 2016
133. Cost-Effectiveness of Biological Asthma Treatments: A Systematic Review and Recommendations for Future Economic Evaluations 2018
134. Cost-Effectiveness of Biological Asthma Treatments: A Systematic Review and Recommendations for Future Economic Evaluations 2018
135. Tezepelumab compared with other biologics for the treatment of severe asthma: a systematic review and indirect treatment comparison 2022
136. Tezepelumab compared with other biologics for the treatment of severe asthma: a systematic review and indirect treatment comparison 2022
137. The future of targeted therapy in chronic spontaneous urticaria 2024
138. The effectiveness of omalizumab in the control of severe uncontrolled asthma in Latin America. An exploratory systematic review and meta-analysis 2020
139. Omalizumab in Patients with Severe Asthma and Persistent Sputum Eosinophilia 2019
140. Omalizumab in Patients with Severe Asthma and Persistent Sputum Eosinophilia 2019
141. Omalizumab versus Mepolizumab as add-on therapy in asthma patients not well controlled on at least an inhaled corticosteroid: A network meta-analysis 2018
142. Omalizumab versus Mepolizumab as add-on therapy in asthma patients not well controlled on at least an inhaled corticosteroid: A network meta-analysis 2018
143. Asthma control in Brazil: a systematic review 2023
144. Measures to reduce maintenance therapy with oral corticosteroid in adults with severe asthma 2016
145. Current state of biologics in treating eosinophilic esophagitis 2023
146. Impact of omalizumab on quality-of-life outcomes in patients with moderate-to-severe allergic asthma 2006
147. Impact of omalizumab on quality-of-life outcomes in patients with moderate-to-severe allergic asthma 2006
148. Omalizumab reduces asthma exacerbations and oral corticosteroid use in patients with severe allergic asthma: a systematic review of observational studies 2019
149. Indirect comparison of bronchial thermoplasty versus omalizumab for uncontrolled severe asthma 2018
150. Impact of Pharmacological Treatments for Chronic Spontaneous Urticaria with an Inadequate Response to H1-Antihistamines on Health-Related Quality of Life: A Systematic Review and Network Meta-Analysis 2022
151. Omalizumab for the treatment of severe persistent allergic asthma: a systematic review and economic evaluation 2013
152. Omalizumab for the treatment of severe persistent allergic asthma: a systematic review and economic evaluation 2013
153. Omalizumab for asthma in adults and children 2014
154. Efficacy of Biologics in Reducing Exacerbations Requiring Hospitalization or an Emergency Department Visit in Patients with Moderate or Severe, Uncontrolled Asthma 2025
155. Efficacy of Biologics in Reducing Exacerbations Requiring Hospitalization or an Emergency Department Visit in Patients with Moderate or Severe, Uncontrolled Asthma 2025
156. Efficacy of Biologics in Reducing Exacerbations Requiring Hospitalization or an Emergency Department Visit in Patients with Moderate or Severe, Uncontrolled Asthma 2025
157. The safety of monoclonal antibodies in asthma 2016
158. Anti-IgE: A treatment option in allergic rhinitis? 2021
159. Anti-IgE: A treatment option in allergic rhinitis? 2021
160. Biologicals in atopic disease in pregnancy: An EAACI position paper 2021
161. Biologicals in atopic disease in pregnancy: An EAACI position paper 2021
162. Exploring the Interaction between Fractional Exhaled Nitric Oxide and Biologic Treatment in Severe Asthma: A Systematic Review 2023
163. Interventions for autumn exacerbations of asthma in children 2018
164. Interventions for autumn exacerbations of asthma in children 2018
165. Interventions for autumn exacerbations of asthma in children 2016
166. A comparison of the effectiveness of biologic therapies for asthma A systematic review and network meta-analysis 2023
167. Approval of the anti-IgE antibody omalizumab for the treatment of severe persistent bronchial asthma 2006
168. Prevalence, management and anaphylaxis risk of cold urticaria: a systematic review and meta-analysis 2022
169. Prevalence, management and anaphylaxis risk of cold urticaria: a systematic review and meta-analysis 2022
170. Prevalence, Management, and Anaphylaxis Risk of Cold Urticaria: A Systematic Review and Meta-Analysis 2022
171. Biologic Therapy in Pediatric Chronic Rhinosinusitis: A Systematic Review 2024
172. Biologic Therapy in Pediatric Chronic Rhinosinusitis: A Systematic Review 2024
173. Review: omalizumab reduces exacerbation and steroid use in chronic asthma: commentary 2007
174. Anti-IgE and Anti-IL5 Biologic Therapy in the Treatment of Nasal Polyposis: A Systematic Review and Meta-analysis 2017
175. Anti-IgE and Anti-IL5 Biologic Therapy in the Treatment of Nasal Polyposis: A Systematic Review and Meta-analysis 2017
176. Management of chronic rhinosinusitis with nasal polyps and coexisting asthma: A systematic review 2015
177. Management of chronic rhinosinusitis with nasal polyps and coexisting asthma: A systematic review 2015
178. Systematic review on the use of omalizumab for the treatment of asthmatic children and adolescents 2015
179. Systematic review on the use of omalizumab for the treatment of asthmatic children and adolescents 2015
180. Efficacy and Safety of Subcutaneous Omalizumab vs Placebo as Add-on Therapy to Corticosteroids for Children and Adults With Asthma A Systematic Review 2011
181. Efficacy and safety of subcutaneous omalizumab vs placebo as add-on therapy to corticosteroids for children and adults with asthma: a systematic review 2011
182. Cost Effectiveness of Pharmacological Treatments for Asthma: A Systematic Review 2018
183. Predictors of response to medications for asthma in pediatric patients: A systematic review of the literature 2020
184. Cost-utility of omalizumab for the treatment of uncontrolled moderate-to-severe persistent pediatric allergic asthma in a middle-income country 2021
185. Cost Effectiveness of Pharmacological Treatments for Asthma: A Systematic Review 2018
186. Predictors of response to medications for asthma in pediatric patients: A systematic review of the literature 2020
187. Cost-utility of omalizumab for the treatment of uncontrolled moderate-to-severe persistent pediatric allergic asthma in a middle-income country 2021
188. Omalizumab for the Treatment of Solar Urticaria: Case Series and Systematic Review of the Literature 2018
189. Effect of Biologic Therapies on Airway Hyperresponsiveness and Allergic Response: A Systematic Literature Review 2023
190. Effect of Biologic Therapies on Airway Hyper responsiveness and Allergic Response: A Systematic Literature Review 2023
191. Comparative effectiveness of mepolizumab and omalizumab in severe asthma: An indirect comparison 2016
192. Omalizumab for the treatment of inadequately controlled allergic rhinitis: a systematic review and meta-analysis of randomized clinical trials 2014
193. Omalizumab for the Treatment of Inadequately Controlled Allergic Rhinitis: A Systematic Review and Meta-Analysis of Randomized Clinical Trials 2014
194. Asthma and other recurrent wheezing disorders in children (chronic) 2012
195. Incremental net monetary benefit of biologic therapies in moderate to severe asthma: a systematic review and meta-analysis of economic evaluation studies 2023
196. Incremental net monetary benefit of biologic therapies in moderate to severe asthma: a systematic review and meta-analysis of economic evaluation studies 2023
197. Anti-IgE for chronic asthma 2003
198. Anti-IgE for chronic asthma in adults and children 2004
199. Anti-IgE for chronic asthma in adults and children 2006
200. Effect of monoclonal antibody drug therapy on mucosal biomarkers in airway disease: A systematic review 2020
201. Effect of monoclonal antibody drug therapy on mucosal biomarkers in airway disease: A systematic review 2020
202. International consensus statement on allergy and rhinology: Allergic rhinitis-2023 2023
203. International consensus statement on allergy and rhinology: Allergic rhinitis - 2023 2023
204. International Consensus Statement on Allergy and Rhinology: Allergic Rhinitis 2018
205. International Consensus Statement on Allergy and Rhinology: Allergic Rhinitis 2018
206. A comprehensive analysis on the safety of two biologics dupilumab and omalizumab 2024
207. A comprehensive analysis on the safety of two biologics dupilumab and omalizumab 2024
208. Meta-analysis of the adoption of omalizumab in the treatment of pediatric allergic diseases 2024
209. Off-Label Use of Monoclonal Antibodies for Eosinophilic Esophagitis in Humans: A Scoping Review 2024
210. Clinical Efficacy and Safety of Omalizumab in the Treatment of Allergic Rhinitis: A Systematic Review and Meta-analysis of Randomized Clinical Trials 2020
211. Combination of omalizumab with allergen immunotherapy versus immunotherapy alone for allergic diseases: A meta-analysis of randomized controlled trials 2024
212. Combination of omalizumab with allergen immunotherapy versus immunotherapy alone for allergic diseases: A meta-analysis of randomized controlled trials 2024
213. GA2LEN ANACARE consensus statement: Potential of omalizumab in food allergy management 2024
214. GA(2)LEN ANACARE consensus statement: Potential of omalizumab in food allergy management 2024
215. Omalizumab in IgE-Mediated Food Allergy: A Systematic Review and Meta-Analysis 2023
216. Omalizumab monotherapy reduced allergic symptoms and improved quality of life in patients with IgE-mediated food allergy: A systematic review and meta-analysis 2021
217. 评估FeNO水平对奥马珠单抗在重度过敏性哮喘患者早期治疗疗效的预测能力 %J 临床肺科杂志 2023
218. 奥马珠单抗治疗难治性过敏性哮喘疗效和安全性的荟萃分析 %J 中华医学杂志 %J National Medical Journal of China 2022
219. 奥马珠单抗治疗重度过敏性哮喘的药物经济学评价 %J 中国药房 2024
220. 奥马珠单抗治疗难治性过敏性哮喘疗效和安全性的荟萃分析 %J 中华医学杂志 2022
221. 奥马珠单抗治疗难治性变应性哮喘效果的Meta分析 %J 中国医药导报 2019
222. 奥马珠单抗治疗儿童中重度过敏性哮喘的有效性和安全性系统评价 2021
223. 奥马珠单抗治疗儿童中重度过敏性哮喘的有效性和安全性系统评价 %J 中国医院用药评价与分析 2021
224. 廖浚邑 (2024). 奥马珠单抗治疗对中重度过敏性哮喘患者肺功能改善的Meta分析 硕士, 重庆医科大学.
225. 廖浚邑 (2024). 奥马珠单抗治疗对中重度过敏性哮喘患者肺功能改善的Meta分析 硕士.
226. 奥马珠单抗治疗儿童哮喘的快速卫生技术评估 %J 中国循证医学杂志 2025
227. 刘香 (2024). 奥马珠单抗治疗儿童中重度过敏性哮喘有效性及安全性Meta分析 硕士, 南昌大学医学部.
228. 奥马珠单抗治疗难治性哮喘疗效与安全性的Meta分析 %J 中华临床医师杂志(电子版) 2013
229. 奥马珠单抗治疗儿童及青少年过敏性哮喘的系统评价 %J 中国药物评价 2021
230. 奥马佐单抗治疗过敏性支气管哮喘疗效与安全性的系统评价 %J 中国循证医学杂志 %J Chinese Journal of Evidence-Based Medicine 2013
231. 奥马珠单抗治疗难治性哮喘疗效的Meta分析 2015
232. 奥马珠单抗治疗难治性哮喘疗效的Meta分析 %J 中国呼吸与危重监护杂志 2015
233. 苗伟伟, 汪凤凤, 解卫平, 黄茂, 殷凯生 and 周林福 (2013). 奥马珠单抗治疗难治性哮喘疗效的Meta分析. 中华医学会呼吸病学年会——2013第十四次全国呼吸病学学术会议论文集. 大连: 560-560.
234. 苗伟伟, 汪凤凤, 解卫平, 黄茂, 殷凯生 and 周林福 (2013). 奥马珠单抗治疗难治性哮喘疗效的Meta分析. 中华医学会呼吸病学年会——2013（第十四次全国呼吸病学学术会议）, 中国辽宁大连.
235. 牟姗 (2019). 奥马珠单抗治疗难治性变应性哮喘效果的meta分析 硕士, 重庆医科大学.
236. 奥马珠单抗治疗难治性变应性哮喘效果的Meta分析 2019
237. 奥马珠单抗治疗难治性变应性哮喘效果的Meta分析 %J 中国医药导报 2019
238. 奥马珠单抗治疗儿童及青少年过敏性哮喘的系统评价 2021
239. 奥马珠单抗治疗儿童及青少年过敏性哮喘的系统评价 %J 中国药物评价 2021
240. 薛宽宽 (2023). 奥马珠单抗治疗中重度过敏性哮喘效果的Meta分析 硕士, 山西医科大学.
241. 奥马珠单抗治疗儿童中重度过敏性哮喘的有效性和安全性系统评价 %J 中国医院用药评价与分析 2021
242. 奥马珠单抗治疗难治性哮喘疗效的Meta分析 %J 中国呼吸与危重监护杂志 2015
243. 奥马珠单抗治疗儿童中重度支气管哮喘的临床综合评价 %J 临床药物治疗杂志 2024

**Non-bronchial asthma or cases where asthma is not the main disease for treatment.(n=304)**

1. CHEST 2015 Annual Meeting Abstracts 2015
2. Proceedings of the Canadian Society of Allergy and Clinical Immunology Annual Scientific Meeting 2015 2016
3. 2017 Canadian Respiratory Conference Abstracts: Part 1: Moderated Posters 2017
4. (2017). CADTH Common Drug Reviews. Clinical Review Report: Omalizumab (Xolair). Ottawa (ON), Canadian Agency for Drugs and Technologies in Health
5. Copyright © 2017 Canadian Agency for Drugs and Technologies in Health.
6. CHEST 2018 Annual Meeting Abstracts 2018
7. A systematic review of the impact of rhinitis and its treatment in severe asthma 2017
8. EAACI Biologicals Guidelines—dupilumab for children and adults with moderate-to-severe atopic dermatitis 2021
9. Efficacy and safety of treatment with omalizumab for chronic spontaneous urticaria: A systematic review for the EAACI Biologicals Guidelines 2021
10. Efficacy and safety of treatment with biologicals for severe chronic rhinosinusitis with nasal polyps: A systematic review for the EAACI guidelines 2021
11. Potential Treatments for Food Allergy 2015
12. Adverse events of biological therapy in chronic rhinosinusitis with nasal polyps: A systematic review 2022
13. Role of nemolizumab and omalizumab in management of atopic dermatitis: A review 2021
14. The evaluation of therapeutic outcomes of biologics in allergic fungal rhinosinusitis: a systematic review and meta-analysis 2025
15. Omalizumab for chronic urticaria in children younger than 12 years 2019
16. Total ige as a marker for chronic spontaneous urticaria 2021
17. Brazilian guideline for the use of immunobiologicals in chronic rhinosinusitis with nasal polyps ‒ 2024 update 2024
18. Randomized Comparative Clinical Study of First Global Omalizumab Biosimilar with Innovator Product in Moderate to Severe Persistent Asthma 2020
19. Dupilumab Leads to Clinical Improvements including the Acquisition of Tolerance to Causative Foods in Non-Eosinophilic Esophagitis Eosinophilic Gastrointestinal Disorders 2023
20. Impact of a Decade of Research Into Atopic Dermatitis 2023
21. The Impact of Monoclonal Antibody Usage on Hearing Outcomes: A Systematic Review 2025
22. Feasibility of matching-adjusted indirect compar ison (MAIC) of omalizumab (OMA) vs. mepolizumab (MEPO) in moderate-to-severe asthma 2016
23. DIFFERENCES IN ELIGIBILITY CRITERIA AND BASELINE PATIENT CHARACTERISTICS AMONGST TRIALS OF BIOLOGIC THERAPIES IN ASTHMA 2018
24. Evidence-based management of nasal polyposis by intranasal corticosteroids: From the cause to the clinic 2011
25. Omalizumab Improves Outcomes in Patients with Chronic Rhinosinusitis with Nasal Polyps Irrespective of Asthma Status 2020
26. Current and future treatment options for adult chronic rhinosinusitis: Focus on nasal polyposis 2015
27. Omalizumab and cancer risk: Current evidence in allergic asthma, chronic urticaria, and chronic rhinosinusitis with nasal polyps 2022
28. Is it time to reduce our phobia of inhaled corticosteroids? 2004
29. MSR47 Feasibility Assessment of Indirect Treatment Comparison of Biologics for Treating Chronic Rhinosinusitis with Nasal Polyps 2022
30. Biologic Therapies in HIV/AIDS Patients with Inflammatory Diseases: A Systematic Review of the Literature 2023
31. Barbosa, A., F. Magro, G. B. M. Buttelli, R. N. T. Prioli, G. Dorneles, B. Marmett, N. B. Schneider, C. Borges Migliavaca and M. Falavigna (2024). HTA161 Systematic Review With Indirect Comparison of Efficacy and Safety of Dupilumab Versus Omalizumab for Severe Asthma With Allergic Phenotype. 27: S385.
32. Reply to “Olfactory Function and Biologic Treatments: A Comment on Available Real-life Studies” 2023
33. Improvement in Smell Using Monoclonal Antibodies Among Patients With Chronic Rhinosinusitis With Nasal Polyps: A Systematic Review 2023
34. Biotherapy and treatment of adult primary chronic rhinosinusitis with nasal polyps: Cellular and molecular bases 2021
35. Omalizumab in eosinophilic granulomatosis with polyangiitis: friend or foe? A systematic literature review 2020
36. Hypersensitivity reactions to biologicals: An EAACI position paper 2022
37. Gateways to Clinical Trials 2006
38. A confluence of advanced treatment options for atopic dermatitis, eosinophilic lung diseases and chronic urticaria brought about by the revolutionary discovery of biologics and Janus kinase inhibitors 2022
39. Management of Pediatric Urticaria with Review of the Literature on Chronic Spontaneous Urticaria in Children 2018
40. Internalized stigma in chronic urticaria: A case–control study 2022
41. Comparing biologicals for severe chronic rhinosinusitis with nasal polyps: A network meta-analysis 2022
42. Drugs for airway disease 2016
43. Practical approach to diagnosis and treatment of ocular allergy: a 1-year systematic review 2007
44. New therapies for allergic rhinitis 2014
45. Omalizumab and the risk of malignancy: Results from a pooled analysis 2012
46. Comparison of Different Biologics for Treating Chronic Rhinosinusitis With Nasal Polyps: A Network Analysis 2022
47. Efficacy and Safety of Biologics for Chronic Rhinosinusitis With Nasal Polyps: A Meta-Analysis of Real-World Evidence 2025
48. House Dust Mite Respiratory Allergy: An Overview of Current Therapeutic Strategies 2015
49. Clinical Practice of Allergen Immunotherapy for Allergic Rhinoconjunctivitis and Asthma: An Expert Panel Report 2020
50. Effect of Omalizumab on Exacerbations and Glucocorticoid Use in Patients with Allergic Bronchopulmonary Aspergillosis: A Systematic Review and Meta-Analysis 2022
51. TIME TO CLINICALLY MEANINGFUL RESPONSE TO OMALIZUMAB IN PATIENTS WITH CHRONIC SPONTANEOUS URTICARIA 2022
52. Safety of omalizumab in patients with chronic idiopathic/spontaneous urticaria (CIU/CSU): Pooled analysis of three randomized, double-blind, placebo-controlled Phase III studies (ASTERIA I, ASTERIA II, and GLACIAL) 2015
53. Omalizumab: An effective anti-IgE treatment for allergic asthma and rhinitis 2004
54. What is the contribution of IgE to nasal polyposis? 2021
55. Systematic literature review of the epidemiology and clinical burden of chronic rhinosinusitis with nasal polyposis 2020
56. Diagnosis and management of eosinophilic otitis media: a systematic review 2021
57. Efficacy of Biologics in Patients with Allergic Bronchopulmonary Aspergillosis: A Systematic Review and Meta-Analysis 2024
58. Knowledge Mapping of COVID-19 and Asthma/ Allergic Rhinitis: A Visual and Bibliometric Analysis 2025
59. Dermatologic uses of omalizumabtitle* 2017
60. Biologics for chronic rhinosinusitis 2021
61. Systemic treatments for atopic dermatitis (eczema): Systematic review and network meta-analysis of randomized trials 2023
62. Association Between Serum Total IgE Levels and Clinical Response to Omalizumab for Chronic Spontaneous Urticaria: A Systematic Review and Meta-Analysis 2023
63. International ERS/ATS guidelines on definition, evaluation and treatment of severe asthma 2014
64. 2021 American College of Rheumatology/Vasculitis Foundation Guideline for the Management of Antineutrophil Cytoplasmic Antibody–Associated Vasculitis 2021
65. From IgE to clinical trials of allergic rhinitis 2015
66. Omalizumab and pregnancy: A case report 2013
67. Allergen immunotherapy: Immunomodulatory treatment for allergic diseases 2006
68. Biologics and Allergy Immunotherapy in the Treatment of Allergic Diseases 2020
69. Common characteristics of upper and lower airways in rhinitis and asthma: ARIA update, in collaboration with GA2LEN 2007
70. Omalizumab: Treatment of allergic rhinitis treatment of asthma 2002
71. Omalizumab for the Treatment of Bullous Pemphigoid: A Systematic Review of Efficacy and Safety 2022
72. Treatment for food allergy: Current status and unmet needs 2023
73. Anti-immunoglobulin E for food allergy 2023
74. Beta-2 Agonists May be Superior to Epinephrine to Relieve Severe Anaphylactic Uterine Contractions 2021
75. The role of allergen challenge chambers in the evaluation of anti-allergic medication: An international consensus paper 2006
76. Corticosteroids in respiratory diseases in children 2012
77. Does the use of efficacy or effectiveness evidence in cost-effectiveness analysis matter? 2017
78. Anaphylaxis in children: Current understanding and key issues in diagnosis and treatment 2012
79. Anti-IgE therapy with omalizumab in asthma and allergic rhinitis 2005
80. Another Therapy for Chronic Rhinosinusitis With Nasal Polyps 2023
81. The effect of biologics in lung function and quality of life of patients with united airways disease: A systematic review 2024
82. Chronic inducible urticaria: A systematic review of treatment options 2018
83. Chronic Rhinosinusitis and Aspirin-Exacerbated Respiratory Disease 2016
84. Omalizumab 2001
85. Systematic review of models used in economic analyses in moderate-to-severe asthma and COPD 2016
86. Effect of omalizumab on glucocorticoid use and lung function in the treatment of allergic bronchopulmonary aspergillosis in people with cystic fibrosis: a systematic review and meta-analysis 2022
87. Effect of omalizumab on glucocorticoid use and lung function in patients with allergic bronchopulmonary aspergillosis -A comorbidity subgroup analysis 2023
88. Does the combination of omalizumab and Subcutaneous allergen immunotherapy (SCIT) have an add-on benefit to the efficacy and safety of allergen immunotherapy in asthma and allergic rhinitis? Systematic review 2017
89. Omalizumab Induced Acute Motor Conduction Block Neuropathy: Case report and literature review 2020
90. Efficacy and Safety of Ligelizumab in Chronic Spontaneous Urticaria: Results from the Phase-3 Pivotal Trials 2023
91. Targeted therapy in eosinophilic chronic obstructive pulmonary disease 2021
92. Oral immunotherapy for peanut allergy: The con argument 2020
93. Evolution and emergence of therapeutic monoclonal antibodies what cardiologists need to know 2013
94. A systematic review protocol: health economic evaluations of immunotherapy and biologics for food allergy management 2024
95. Allergen immunotherapy: 100 years, but it does not look like 2012
96. Aco (Asthma–copd overlap) is independent from copd, a case in favor: A systematic review 2021
97. Current concepts and therapeutic strategies for allergic rhinitis 2005
98. Omalizumab may protect allergic patients against COVID-19: A systematic review 2023
99. Management paradigms for chronic rhinosinusitis in individuals with asthma: An evidence-based review with recommendations 2023
100. The Combination of Dupilumab with Other Monoclonal Antibodies 2023
101. Update on oral immunotherapy for egg allergy 2017
102. Retail Food Equivalents for PosteOral Immunotherapy Dosing in the Omalizumab as Monotherapy and as Adjunct Therapy to Multi-Allergen Oral Immunotherapy in Food-Allergic Children and Adults (OUtMATCH) Clinical Trial 2023
103. Efficacy and Safety of Specific Immunotherapy Combined with Biologics in Allergic Rhinitis and Asthma: A Systematic Review and Network Meta-Analysis 2025
104. New approaches to allergen immunotherapy 2018
105. Choice of instrument impacts healthcare decisions: Effect of source of utility derived from the same patient population on cost-effectiveness 2017
106. Medical Management of Headache and Facial Pain in CRS: A Systematic Review and Meta-Analysis 2024
107. Omalizumab for atopic dermatitis: case series and a systematic review of the literature 2017
108. Measuring adherence to therapy in airways disease 2021
109. Effectiveness of ustekinumab in patients with atopic dermatitis: analysis of real-world evidence 2022
110. Aspirin exacerbated respiratory disease (AERD): molecular and cellular diagnostic & prognostic approaches 2021
111. A systematic review of the diagnostic accuracy of volatile organic compounds in airway diseases and their relation to markers of type-2 inflammation 2021
112. Evolution of anti-IgE treatment 2016
113. Role of immunotherapy in chronic rhinosinusitis: A systematic review 2019
114. The role of biologics in chronic rhinosinusitis: a systematic review 2020
115. Cardiovascular and cerebrovascular events among patients receiving omalizumab: pooled analysis of patient-level data from 25 randomized, double-blind, placebo-controlled clinical trials 2017
116. Omalizumab for chronic urticaria: A case series and overview of the literature 2012
117. Statement of the spanish society of allergology and clinical immunology on provocation tests with aspirin/nonsteroidal anti-inflammatory drugs 2020
118. Emerging treatments for dermatologic diseases in infants, children, and adolescents: a systematic review of clinical trials on biologics and small molecule inhibitors 2025
119. Prevention and Management of High-Burden Noncommunicable Diseases in School-Age Children: A Systematic Review 2022
120. Anti-IgE therapy for allergic bronchopulmonary aspergillosis in people with cystic fibrosis 2013
121. Anti-IgE therapy for allergic bronchopulmonary aspergillosis in people with cystic fibrosis 2015
122. Anti-IgE therapy for allergic bronchopulmonary aspergillosis in people with cystic fibrosis 2018
123. Anti-IgE therapy for allergic bronchopulmonary aspergillosis in people with cystic fibrosis 2021
124. Omalizumab in Allergic Bronchopulmonary Aspergillosis: A Systematic Review and Meta-Analysis 2023
125. Influence of prolonged treatment with omalizumab on the development of solid epithelial cancer in patients with atopic asthma and chronic idiopathic urticaria: A systematic review and meta-analysis 2019
126. Clinical outcomes of patients with idiopathic anaphylaxis receiving omalizumab 2021
127. Use of omalizumab for management of idiopathic anaphylaxis: A systematic review and retrospective case series 2021
128. Child with atopic dermatitis 2015
129. MARKERS OF TH2 INFLAMMATION IN PATIENTS WITH CHRONIC SPONTANEOUS URTICARIA: A SYSTEMATIC LITERATURE REVIEW 2018
130. Total IgE as a biomarker of omalizumab response in chronic spontaneous urticaria: A meta-analysis 2024
131. Hypersensitivity and immunologic reactions to biologics: opportunities for the allergist 2016
132. Role of Biologics in Chronic Rhinosinusitis With Nasal Polyposis: State of the Art Review 2021
133. Meeting the Needs of the Changing Landscape of Food Allergy Management 2025
134. Current View of Chronic Spontaneous Urticaria 2023
135. New treatments for chronic urticaria 2020
136. Treatment of urticarial vasculitis: A systematic review 2019
137. Efficacy and Safety of Biologics for Chronic Rhinosinusitis With Nasal Polyps 2022
138. Treatments of cold urticaria: A systematic review 2019
139. Prevalence, Clinical Manifestations, Treatment, and Clinical Course of Chronic Urticaria in Elderly: A Systematic Review 2022
140. Clinical practice guideline for diagnosis and management of urticaria 2016
141. Delayed Pressure Urticaria: A Systematic Review of Treatment Options 2020
142. Symptomatic Dermographism: A Systematic Review of Treatment Options 2020
143. Biologic agents licensed for severe asthma: A systematic review and meta-analysis of randomised controlled trials 2023
144. MORE THAN MEETS THE EYE: A CASE OF DUPILUMAB-ASSOCIATED UVEITIS 2022
145. Beneficial effects of Omalizumab therapy in allergic bronchopulmonary aspergillosis: A synthesis review of published literature 2017
146. How consistent are the key recommendations, and what is the quality of guidelines and expert consensus regarding paediatric cow’s milk protein allergy? 2024
147. Nine cases of refractory bullous pemphigoid treated with dupilumab and literature review 2023
148. An epigenome-wide association study of total serum immunoglobulin E concentration 2015
149. Airway transcriptomic profiling after bronchial thermoplasty 2019
150. Current and emerging biologic therapies for allergic rhinitis and chronic rhinosinusitis 2020
151. An update on anti-ige therapy in pediatric respiratory diseases 2017
152. Biologic drugs in chronic spontaneous urticaria 2021
153. Emerging and future therapies for Allergic rhinitis 2015
154. Adverse Events for Monoclonal Antibodies in Patients with Allergic Rhinitis: A Systematic Review and Meta-Analysis of Randomized Clinical Trials 2023
155. Evidence-based use of antihistamines for treatment of allergic conditions 2023
156. Therapeutic targets in lung tissue remodelling and fibrosis 2021
157. Efficacy and Safety of Omalizumab for the Treatment of Severe or Poorly Controlled Allergic Diseases in Children: A Systematic Review and Meta-Analysis 2022
158. The Multiple Facets of Cow's Milk Allergy 2025
159. The genome-wide association study of serum IgE levels demonstrated a shared genetic background in allergic diseases 2024
160. Targeted Therapies for Eosinophilic Gastrointestinal Disorders 2020
161. Guidelines on eosinophilic esophagitis: evidence-based statements and recommendations for diagnosis and management in children and adults 2017
162. Update on Potential Therapies for IgE-Mediated Food Allergy 2017
163. Management of allergic bronchopulmonary aspergillosis: A review and update 2012
164. Omalizumab: Other indications and unanswered questions 2005
165. Biologic drugs in treating allergic bronchopulmonary aspergillosis in patients with cystic fibrosis: a systematic review 2022
166. Algorithm for the management of asthma in pregnant women: a protocol to optimize processes in healthcare 2017
167. Oral immunotherapy for food allergy: A spanish guideline. immunotherapy egg and milk spanish guide (ITEMS guide). part i: Cow milk and egg oral immunotherapy: Introduction, methodology, rationale, current state, indications, contraindications, and oral immunotherapy build-up phase 2017
168. Systematic review of omalizumab for refractory clonal and non-clonal mast cell activation syndrome 2025
169. Evidence-Based Guidelines for the Management of Allergic Bronchopulmonary Aspergillosis (ABPA) in Children and Adolescents with Asthma 2023
170. The Crucial Role of IgE as a Predictor of Treatment Response to Omalizumab in Chronic Spontaneous Urticaria 2023
171. Omalizumab treatment in patients with chronic inducible urticaria: A systematic review of published evidence 2018
172. A systematic review and economic evaluation of subcutaneous and sublingual allergen immunotherapy in adults and children with seasonal allergic rhinitis 2013
173. Pharmacotherapeutic strategies for allergic rhinitis: Matching treatment to symptoms, disease progression, and associated conditions 2013
174. Dual biologic or small-molecule therapy in patients with atopic dermatitis: A systematic review 2025
175. The future of targeted therapy in chronic spontaneous urticaria 2024
176. Biologic Therapies in Chronic Rhinosinusitis with Nasal Polyposis: Overview of Systematic Reviews and Updated Systematic Review 2023
177. Journal Club: Biologics and Potential for Immune Modulation in Chronic Obstructive Lung Disease 2022
178. Molecular targets for cystic fibrosis and therapeutic potential of monoclonal antibodies 2022
179. Biological treatments in atopic dermatitis 2015
180. Effects of dupilumab for severe eosinophilic asthma COPD overlap 2021
181. Development of dermatomyositis in patients on biologic therapy: A systematic review 2022
182. Management of United Airway Disease Focused on Patients With Asthma and Chronic Rhinosinusitis With Nasal Polyps: A Systematic Review 2022
183. Biologics in allergic rhinitis 2023
184. A practical guideline of genomics-driven drug discovery in the era of global biobank meta-analysis 2022
185. Comorbid allergic rhinitis and asthma: important clinical considerations 2022
186. Omalizumab-associated eosinophilic granulomatosis with polyangiitis (Churg-Strauss syndrome) 2017
187. New forms of allergy immunotherapy for rhinitis and asthma 2014
188. Current state of biologics in treating eosinophilic esophagitis 2023
189. The Efficacy and Evidence-Based Use of Biologics in Children and Adolescents Using Monoclonal Antibodies and Fusion Proteins as Treatments 2019
190. Impact of Pharmacological Treatments for Chronic Spontaneous Urticaria with an Inadequate Response to H1-Antihistamines on Health-Related Quality of Life: A Systematic Review and Network Meta-Analysis 2022
191. Biologic Therapy for Eosinophilic Granulomatosis With Polyangiitis (Churg-Strauss): Comment on the Article by Jachiet et al 2017
192. Medical therapy as the primary modality for the management of chronic rhinosinusitis 2013
193. Biologics for the treatment of COPD with increased peripheral blood eosinophils: A systematic review and meta-analysis 2021
194. Roles of omalizumab in various allergic diseases 2020
195. Monoclonal antibodies in allergy; Updated applications and promising trials 2015
196. SIT beyond respiratory diseases 2011
197. Reduction in Corticosteroid Use Among Patients Receiving Omalizumab in Real World Settings: A Systematic Literature Review of Non-Randomized Studies 2016
198. Novel therapeutic interventions for allergic rhinitis 2006
199. Editorial: Advanced therapeutic delivery for the management of chronic respiratory diseases 2022
200. Anti-IgE: A treatment option in allergic rhinitis? 2021
201. Biologicals in atopic disease in pregnancy: An EAACI position paper 2021
202. A snapshot of exhaled nitric oxide and asthma characteristics: experience from high to low income countries 2022
203. Basophils from allergy to cancer 2022
204. What's new in pediatric dermatology?: Part II. Treatment 2013
205. Prevalence, Management, and Anaphylaxis Risk of Cold Urticaria: A Systematic Review and Meta-Analysis 2022
206. Review of Dual Biologics in Specialty Pharmacy Practice 2023
207. A report from the 106th International Conference of the American Thoracic Society (May 14-19, 2010 - New Orleans, Louisiana, USA) 2010
208. EoE behaves as a unique Th2 disease: a narrative review 2023
209. Omalizumab as an add-on to allergen-specific immunotherapy: A systematic review 2015
210. Biologic Therapy in Pediatric Chronic Rhinosinusitis: A Systematic Review 2024
211. Rezabakhsh, A. and H. Soleimanpour (2022). Aspirin Desensitization/Challenge in Patients with Cardiovascular Diseases: Current Trends and Advances. 5: 147-189.
212. Anti-IgE and Anti-IL5 Biologic Therapy in the Treatment of Nasal Polyposis: A Systematic Review and Meta-analysis 2017
213. Management of chronic rhinosinusitis with nasal polyps and coexisting asthma: A systematic review 2015
214. Sex differences in adult asthma and COPD therapy: a systematic review 2023
215. Omalizumab treatment leading to excessive weight gain 2017
216. Chronic spontaneous urticaria remission definition and therapy stepping down: World Allergy Organization position paper 2025
217. Latest developments in the management of allergic rhinitis 2004
218. Development of Cutaneous T-Cell Lymphoma Following Biologic Treatment: A Systematic Review 2023
219. Efficacy and safety of allergen-specific immunotherapy in rhinitis, rhinoconjunctivitis, and bee/wasp venom allergies 2005
220. Comparative safety of monoclonal antibodies in chronic inflammatory airway diseases (chronic sinusitis with nasal polyposis and asthma): A network meta-analysis 2024
221. Adverse events for biologics in patients with CRSwNP: A meta-analysis 2022
222. Local Immunoglobulin E in nasal polyps: Role and modulation 2022
223. Current treatment options for idiopathic angioedema 2015
224. Systemic treatments in the management of atopic dermatitis: A systematic review and meta-analysis 2021
225. World allergy organization anaphylaxis guidelines: 2013 update of the evidence base 2013
226. Omalizumab for the Treatment of Solar Urticaria: Case Series and Systematic Review of the Literature 2018
227. Management of allergic rhinitis 2014
228. A practical view of immunotherapy for food allergy 2016
229. Effect of Biologic Therapies on Airway Hyperresponsiveness and Allergic Response: A Systematic Literature Review 2023
230. Effect of Biologics on Allergic Response and Airway Hyperresponsiveness: A Systematic Literature Review 2022
231. Reply to Aranda et al 2024
232. Vaccinations in Patients Receiving Systemic Drugs for Skin Disorders: What Can We Learn for SARS-Cov-2 Vaccination Strategies? 2021
233. The potential role of omalizumab in the treatment of chronic urticaria 2014
234. Roles of ige and histamine in mast cell maturation 2021
235. Omalizumab for the treatment of allergic rhinitis: a systematic review and meta-analysis 2021
236. Omalizumab-related adverse events in clinical trials of allergic rhinitis: A systematic review and meta-analysis 2011
237. Omalizumab for the Treatment of Inadequately Controlled Allergic Rhinitis: A Systematic Review and Meta-Analysis of Randomized Clinical Trials 2014
238. Efficacy of omalizumab for the treatment of inadequately controlled allergic rhinitis: A systematic review and metaanalysis 2012
239. Systematic review of omalizumab for the treatment of chronic rhinosinusitis 2015
240. Monoclonal antibodies for the treatment of chronic rhinosinusitis with nasal polyposis: a systematic review 2018
241. Pediatric allergic rhinitis and asthma: Can the march be halted? 2013
242. Review on immunotherapy in airway allergen sensitised patients 2015
243. Eosinophilic esophagitis: clinical, endoscopic, histologic and therapeutic differences and similarities between children and adults 2021
244. Allergen immunotherapy in allergic respiratory diseases: From mechanisms to meta-analyses 2012
245. A cost-effectiveness analysis comparing single-inhaler extrafine beclomethasone/formoterol/glycopyrronium bromide against other SITTs in adult patients with uncontrolled asthma in England 2025
246. Considerations on biologicals for patients with allergic disease in times of the COVID-19 pandemic: An EAACI statement 2020
247. Effect of monoclonal antibody drug therapy on mucosal biomarkers in airway disease: A systematic review 2020
248. Specific immunotherapy for allergic rhinitis in children 2014
249. Efficacy of different biologics for treating chronic rhinosinusitis with nasal polyps: a network meta-analysis 2025
250. Adverse reactions to biological modifiers 2004
251. Successful Treatment of the Different Urticaria Subtypes 2015
252. International consensus statement on allergy and rhinology: Allergic rhinitis – 2023 2023
253. International consensus statement on allergy and rhinology: allergic rhinitis-executive summary 2018
254. International Consensus Statement on Allergy and Rhinology: Allergic Rhinitis 2018
255. Consensus-based European guidelines for treatment of atopic eczema (atopic dermatitis) in adults and children: part II 2018
256. Presence of positive skin prick tests to inhalant allergens and markers of T2 inflammation in subjects with chronic spontaneous urticaria (CSU): a systematic literature review 2020
257. Clinical dose–response for a broad set of biological products: A model-based meta-analysis 2018
258. Efficacy and safety of omalizumab in chronic rhinosinusitis with nasal polyps: A systematic review and meta-analysis of randomised controlled trials 2021
259. Optimization of Clinical Biological Therapies for Chronic Rhinosinusitis With Nasal Polyps 2024
260. A comprehensive analysis on the safety of two biologics dupilumab and omalizumab 2024
261. Meta-analysis of the adoption of omalizumab in the treatment of pediatric allergic diseases 2024
262. Present state of Japanese cedar pollinosis: The national affliction 2014
263. Efficacy of Biologic Therapies in the Management of Allergic Rhinitis: A Systematic Review 2024
264. Off-Label Use of Monoclonal Antibodies for Eosinophilic Esophagitis in Humans: A Scoping Review 2024
265. Clinical Efficacy and Safety of Omalizumab in the Treatment of Allergic Rhinitis: A Systematic Review and Meta-analysis of Randomized Clinical Trials 2020
266. The office management of recalcitrant rhinosinusitis 2004
267. Frequency of the off-label use of monoclonal antibodies in clinical practice: A systematic review of the literature 2014
268. Combination of omalizumab with allergen immunotherapy versus immunotherapy alone for allergic diseases: A meta-analysis of randomized controlled trials 2024
269. Omalizumab for the treatment of chronic spontaneous urticaria: A meta-analysis of randomized clinical trials 2016
270. Effectiveness and safety of systemic therapy for moderate-to-severe atopic dermatitis in children and adolescent patients: a systematic review 2024
271. Clinical efficacy and safety of MP-AzeFlu for the treatment of allergic rhinitis: a meta-analysis 2021
272. Methods report on the development of the 2013 revision and update of the EAACI/GA2LEN/EDF/WAO guideline for the definition, classification, diagnosis, and management of urticaria 2014
273. GA<SUP>2</SUP>LEN ANACARE consensus statement: Potential of omalizumab in food allergy management 2024
274. The combination of omalizumab and oral immunotherapy induced desensitisation to food allergens and improved quality of life: A systematic review and meta-analysis in patients with IgEmediated food allergy 2021
275. Omalizumab as monotherapy or adjunct to oral immunotherapy in patients with IgE-mediated food allergy is clinically effective: Report on reduced specific IgE levels and skin reactivity to allergens 2021
276. Omalizumab in IgE-Mediated Food Allergy: A Systematic Review and Meta-Analysis 2023
277. 范琦 (2022). 针灸治疗变应性鼻炎的Meta分析及临床随机对照研究 硕士, 中国中医科学院.
278. 耿驰 (2022). 免疫球蛋白E及其受体FcepsilonR1在病理性心脏重塑中的作用和机制研究 博士, 中国医学科学院
279. 抗IgE单抗治疗过敏性联合气道疾病的真实世界研究 %J 中华预防医学杂志 2023
280. 黄立忠 (2009). 抗生素治疗慢性鼻炎的临床研究 硕士, 大连医科大学.
281. 奥马珠单抗治疗阿司匹林加重性呼吸系统疾病的临床疗效观察 %J 中国耳鼻咽喉颅底外科杂志 2025
282. 李德容 (2012). 不伴鼻炎症状的支气管哮喘患者鼻部炎症表现 博士, 广州医科大学.
283. 李华君 (2018). 布地奈德鼻喷雾剂治疗变应性鼻炎的机制研究 硕士, 陆军军医大学.
284. 李家乐 (2011). 小青龙汤治疗变应性鼻炎的文献与实验研究 博士, 南方医科大学.
285. 李林静 (2017). 食物过敏原诱导小鼠结肠炎发生的分子机制研究 博士, 郑州大学.
286. 李姝仪 (2021). ACO疾病动物模型的建立及其潜在机制靶点、候选药物的发现研究 博士, 中国医学科学院
287. 李唯峰 (2023). 人脐带间充质干细胞对变应性鼻炎的干预及机制的初步探讨 硕士, 河北北方学院.
288. 李依娜 (2021). 呼出气一氧化氮在哮喘及鼻炎中的应用研究 博士, 四川大学.
289. 李营营 (2019). RSV毛细支气管炎患儿免疫失衡及hPMSCs对其免疫调控机制的实验研究 博士, 山东大学.
290. 刘博莹 (2012). 吸附于沙尘暴颗粒物上的微生物对卵蛋白诱导的小鼠气道炎症的实验研究 硕士, 中国医科大学.
291. 刘志强 (2013). 半抗原诱导食物过敏发生机制及肥大细胞对Th2型过敏反应的调控作用 博士, 郑州大学.
292. 卢川 (2013). 变应性鼻炎冲击免疫治疗的安全性及有效性研究 硕士, 南方医科大学.
293. 祁雪萍 (2022). 神经介素U通过ERK通路诱导ILC2s活化参与变应性鼻炎发病 博士, 山西医科大学.
294. 钱卫珠 (2011). 新型抗IgE人源化单克隆抗体的结构与功能研究 博士, 第二军医大学.
295. 邱昕 (2016). CRTH2基因与变应性鼻炎遗传易感性的研究 硕士, 解放军总医院
296. 孙丽红 (2015). 儿童变应性鼻炎的下气道炎症表现及干预 博士, 广州医科大学.
297. 孙彦华 (2012). 基于金纳米粒的生物分子化学发光分析新技术 博士, 复旦大学.
298. 王虹茵 (2017). 辽宁地区0-12岁变应性疾病患儿血清变应原谱分析 硕士, 中国医科大学.
299. 王际睿 (2008). 小麦种子蛋白及赤霉病抗性相关基因的分子鉴定 博士, 四川农业大学.
300. 吴洋洋 (2017). 毛细支气管炎临床分析及临床路径实施效果评价 硕士, 温州医科大学.
301. 闫红梅 (2007). 玫瑰糠疹与IgE介导的超敏反应关系的研究 硕士, 河北医科大学.
302. 杨杪 (2005). 热休克蛋白在不同疾病中的生物医学重要性 博士, 华中科技大学.
303. 张家鹏 (2012). 过敏性鼻炎脱敏治疗前后鼻阻力、EOS及ECP相关性研究 硕士, 南方医科大学.
304. 赵桐 (2023). 嗜酸性粒细胞型鼻息肉相关因素分析及预测模型构建 硕士, 青岛大学.

**Cases in the treatment group that did not receive omalizumab therapy or cases in the control group that did not receive placebo therapy(n=220)**

1. Adams, S. G., D. J. Maselli Caceres, S. Durg, H. U. G. O. Dubucq, J. Ritter, N. A. M. I. Pandit-Abid, O. Ledanois, Z. Wang and W. E. I. H. A. N. Cheng (2024). EFFECTIVENESS OF BIOLOGICS IN ASTHMA: COMPARING REAL-WORLD EVIDENCE. 166: A77-A78.
2. UNCONTROLLED TYPE 2 HIGH ASTHMA PRESENTING WITH EXTENSIVE VARICOSE BRONCHIECTASIS AND MUCOUS PLUGGING: COMPLETE RESOLUTION AFTER DUPILUMAB INITIATION 2022
3. Interventions for autumn exacerbations of asthma in children: A systematic review 2018
4. Economic evaluation of biological treatments in patients with severe asthma: a systematic review 2023
5. Novel pharmacological therapies for the treatment of bronchial asthma 2022
6. Advances in the care of adults with asthma and allergy in 2007 2008
7. Are we entering an era of combined biologic use for severe asthma? 2022
8. A systematic review of economic evaluations of therapy in asthma 2010
9. Biologics in severe asthma: the overlap endotype - opportunities and challenges 2020
10. Managing comorbid conditions in severe asthma 2018
11. Magnitude of effect of asthma treatments on Asthma Quality of Life Questionnaire and Asthma Control Questionnaire scores: Systematic review and network meta-analysis 2015
12. Pairwise indirect treatment comparison of dupilumab versus other biologics in patients with uncontrolled persistent asthma 2022
13. Persistent asthma: What approach is best? 2002
14. Guidelines for the management of work-related asthma 2012
15. Bronchial asthma - Issues for the developing world 2015
16. Bayesian network meta-analysis to assess the comparative efficacy and safety of treatments for severe or uncontrolled asthma 2015
17. Editorial: Atopy, asthma, and the elderly: A paradigm for personalized therapy 2017
18. Benson, V., L. Vinals, A. Freitag, G. Sarri, N. I. K. I. Day and R. Alfonso-Cristancho (2024). LONG-TERM SAFETY OF BIOLOGICS IN ASTHMA: A SYSTEMATIC LITERATURE REVIEW. 166: A4636-A4637.
19. Mepolizumab for Treating Severe Eosinophilic Asthma: An Evidence Review Group Perspective of a NICE Single Technology Appraisal 2018
20. EFFICACY OF BIOLOGICS IN PATIENTS WITH SEVERE ALLERGIC ASTHMA, OVERALL AND BY BLOOD EOSINOPHIL COUNT 2022
21. Efficacy of Biologics in Patients with Allergic Severe Asthma, Overall and by Blood Eosinophil Count: A Literature Review 2023
22. Step-up and step-down treatments for optimal asthma control in children and adolescents 2019
23. Evaluating safety of long-acting beta agonists (labas) in patients with asthma 2010
24. Risk and outcomes of covid-19 patients with asthma: A meta-analysis 2020
25. Systematic literature review of asthma biologic self-administration enhanced by a patient perspective 2024
26. The immunology of asthma: Asthma phenotypes and their implications for personalized treatment 2016
27. ERS/EAACI statement on severe exacerbations in asthma in adults: Facts, priorities and key research questions 2019
28. Matching-adjusted comparison of oral corticosteroid reduction in asthma: Systematic review of biologics 2020
29. American Academy of Asthma, Allergy & Immunology - 58th Annual Meeting: 1-6 March 2002, New York, City, NY, USA 2002
30. Effective Management of Severe Asthma with Biologic Medications in Adult Patients: A Literature Review and International Expert Opinion 2022
31. EFFICACY OF BIOLOGIC THERAPIES IN PATIENTS WITH SEVERE, UNCONTROLLED ASTHMA STRATIFIED BY BLOOD EOSINOPHIL COUNT 2021
32. Asthma exacerbations and lung function decline in a pooled analysis of adolescents and adults from randomized controlled trials of omalizumab 2017
33. Comparison of anti-interleukin-5 therapies in patients with severe asthma: global and indirect meta-analyses of randomized placebo-controlled trials 2017
34. Asthma treatments: New and emerging therapies 2015
35. The Impact of Monoclonal Antibodies on Airway Smooth Muscle Contractility in Asthma: A Systematic Review 2021
36. Oral corticosteroids dependence and biologic drugs in severe asthma: Myths or facts? a systematic review of real‐world evidence 2021
37. Monoclonal antibodies in severe asthma: is it worth it? 2019
38. Health economics of asthma: Assessing the value of asthma interventions 2008
39. Combination of Biological Therapy in Severe Asthma: Where We Are? 2023
40. Principal findings of systematic reviews for chronic treatment in childhood asthma 2015
41. Biologics in Asthma and Chronic Obstructive Pulmonary Disorder 2022
42. Biologics for the treatment of severe asthma: Current status report 2023 2023
43. Monoclonal antibodies for the treatment of severe asthma 2020
44. The placebo response on lung function in recent clinical trials of asthma biologics 2019
45. Incidence of Anti-Drug Antibodies to Monoclonal Antibodies in Asthma: A Systematic Review and Meta-Analysis 2023
46. Systematic literature review of the clinical, humanistic, and economic burden associated with asthma uncontrolled by GINA Steps 4 or 5 treatment 2018
47. Targeted interventions for difficult-to-treat asthma 2007
48. Efficacy of benralizumab for patients with severe, uncontrolled atopic asthma by serum immunoglobulin e concentrations 2018
49. Incorporating adherence in cost-effectiveness analyses of asthma: a systematic review 2019
50. The effect of vitamin D as adjunct to allergen immunotherapy (AIT): A systematic review and meta-analysis 2023
51. Gender bias in clinical trials of biological agents for severe asthma: A systematic review 2021
52. The Role of Access and Cost-Effectiveness in Managing Asthma: A Systematic Review 2022
53. Management of patients with exacerbation of bronchial asthma 2006
54. Asthma phenotypes today 2013
55. Childhood asthma: Treatment update 2005
56. BIO-AST Biologics for chronic severe asthma: a network meta-analysis 2022
57. Effects of asthma treatment: The present and future 2006
58. A systematic literature review of burden of illness in adults with uncontrolled moderate/severe asthma 2022
59. Targeted Therapy for Older Patients with Uncontrolled Severe Asthma: Current and Future Prospects 2016
60. Is Inhaler Technique Adequately Assessed and Reported in Clinical Trials of Asthma and Chronic Obstructive Pulmonary Disease Therapy? A Systematic Review and Suggested Best Practice Checklist 2022
61. Specific allergen immunotherapy for the treatment of allergic asthma: A review of current evidence 2017
62. Acute exacerbations of asthma: Epidemiology, biology and the exacerbation-prone phenotype 2009
63. Cost-utility analysis of an integrated care program for children with asthma in a medium-income country 2020
64. Monoclonal antibodies in type 2 asthma: An updated network meta-analysis 2021
65. Dual mechanism of action of T2 inhibitor therapies in virally induced exacerbations of asthma: Evidence for a beneficial counter-regulation 2019
66. A Systematic Review of Decision-Analytic Models for Evaluating Cost-Effectiveness of Asthma Interventions 2019
67. Cost-effectiveness analysis of bronchial thermoplasty versus pharmacological therapies in patients with severe asthma in Mexico, Colombia and Spain 2018
68. Recognition and management of severe asthma: A Canadian Thoracic Society position statement 2017
69. Comparing bronchial thermoplasty with biologicals for severe asthma: Systematic review and network meta-analysis 2023
70. Asthma exacerbation in children: A practical review 2014
71. Reprint of: Perinatal and early childhood environmental factors influencing allergic asthma immunopathogenesis 2014
72. Obesity and asthma: Key clinical questions 2019
73. Impact of body mass index on omalizumab response in adults with moderate-to-severe allergic asthma 2022
74. A review on the effect of COVID-19 in type 2 asthma and its management 2021
75. Evidence for phenotype-driven treatment in asthmatic patients 2011
76. Management of asthma in adults: Current therapy and future directions 2003
77. RECURRENCE OF MRSA EMPYEMA THORACIS IN A PATIENT WITH ASTHMA ON SUCCESSIVE BIOLOGIC THERAPY WITH DUPILUMAB AND BENRALIZUMAB 2023
78. Assessing the Risks and Benefits of Step-Down Asthma Care: A Case-Based Approach 2015
79. Economic evaluation of interventions for the treatment of asthma in children: A systematic review 2020
80. Current drug treatment of asthma 2001
81. Real-life studies of biologics used in asthma patients: key differences and similarities to trials 2019
82. Cytokine and anti-cytokine therapy for the treatment of asthma and allergic disease 2004
83. Pathophysiology of asthma: What has our current understanding taught us about new therapeutic approaches? 2011
84. Stratified approaches to the treatment of asthma 2013
85. Management of severe asthma: A European Respiratory Society/American Thoracic Society guideline 2020
86. Medications and recent patents for status asthmaticus in children 2017
87. Ethnic differences in adverse drug reactions to asthma medications: a systematic review 2016
88. Baseline FEV1 Predicts Response to Biologic Treatment in Severe Asthma 2022
89. Safety and adverse reactions in subcutaneous allergen immunotherapy: a review 2023
90. In the strategies to prevent asthma exacerbations, allergic asthma needs specific treatment 2015
91. Emerging Issues in Pediatric Asthma: Gaps in EPR-3 Guidelines for Infants and Children 2014
92. Role of viruses in asthma 2020
93. Refractory asthma - beyond step 5, the role of new and emerging adjuvant therapies 2015
94. Potential mechanisms connecting asthma, esophageal reflux, and obesity/sleep apnea complex-A hypothetical review 2007
95. Asthma and cystic fibrosis: A tangled web 2014
96. RACIAL/ETHNIC DEMOGRAPHICS OF PARTICIPANTS IN CLINICAL TRIALS OF BIOLOGICS USED FOR ASTHMA 2018
97. Definitions of non-response and response to biological therapy for severe asthma: a systematic review 2023
98. Asthma Cost-Effectiveness Analyses: Are We Using the Recommended Outcomes in Estimating Value? 2018
99. Comparative efficacy of biologics for patients with inadequately controlled asthma: A network meta-analysis 2024
100. Comparative efficacy of biologic agents in patients with difficult-to-treat asthma: a systematic review and network metaanalysis 2022
101. Respiratory reviews in asthma 2013 2014
102. Year in review 2011: Asthma, chronic obstructive pulmonary disease and airway biology 2012
103. Efficacy of Biologics in Severe, Uncontrolled Asthma Stratified by Blood Eosinophil Count: A Systematic Review 2023
104. FCER2 T2206C variant associated with chronic symptoms and exacerbations in steroid-treated asthmatic children 2011
105. Evaluation of real-world mepolizumab use in severe asthma across Europe: the SHARP experience with privacy-preserving federated analysis 2023
106. Economic aspects of application of the Russian biosimilar omalizumab in patients with atopic bronchial asthma of moderate to severe clinical courses 2021
107. Targeting IgE in asthma 2012
108. Biologic agents licensed for severe asthma: a systematic review and meta-analysis of randomised controlled trials 2024
109. Use of Health Related Quality of Life in Clinical Trials for Severe Asthma: A Systematic Review 2021
110. Bronchial thermoplasty or biologics for severe asthma: A systematic review and network meta-analysis 2023
111. Real-world Effectiveness of Mepolizumab in Severe Eosinophilic Asthma: A Systematic Review and Meta-analysis 2021
112. Advanced Biologic Therapies in the Management of Asthma in Children and Adolescents: A Comprehensive Network Meta-Analysis 2024
113. 2020 Updated Asthma Guidelines: Clinical utility of fractional exhaled nitric oxide (FENO) in asthma management 2020
114. Head-To-Head Comparison of Biologic Efficacy in Asthma: What Have We Learned? 2025
115. Effect of Mepolizumab (100 mg Subcutaneous) on Exacerbation Rate in Patients with Severe Eosinophilic Asthma by Omalizumab Eligibility (US Criteria), and Immunoglobulin E and Eosinophilic Subgroups 2018
116. The clinical and pathological histology efficacy of biological therapy for severe asthma with a phenotype of type 2 inflammation - systematic review 2025
117. Identifying super-responders: A review of the road to asthma remission 2025
118. Selecting the right biologic for your patients with severe asthma 2018
119. Safety of Biological Treatments in Asthmatic Patients With Active SARS-CoV-2 Infection: A Systematic Review 2024
120. Periodontal disease associated with corticosteroid in asthma patients-A systematic review 2019
121. An Updated Reappraisal of Dupilumab in Children and Adolescents with Severe Asthma 2024
122. Effects of Therapeutic Antibodies on Gene and Protein Signatures in Asthma Patients: A Comparative Systematic Review 2022
123. Management of asthma in childhood: Study protocol of a systematic evidence update by the Paediatric Asthma in Real Life (PeARL) Think Tank 2021
124. Refractory asthma 2002
125. Cost-Effectiveness of Biological Asthma Treatments: A Systematic Review and Recommendations for Future Economic Evaluations 2018
126. Anti-il5 therapies for severe eosinophilic asthma: Literature review and practical insights 2020
127. Tezepelumab compared with other biologics for the treatment of severe asthma: a systematic review and indirect treatment comparison 2022
128. ERS/TSANZ Task Force Statement on the management of reproduction and pregnancy in women with airways diseases 2020
129. An evaluation of mepolizumab for the treatment of severe asthma 2019
130. Bronchial Thermoplasty in Severe Asthma Topical Collection on Interventional Pulmonology 2017
131. Asthma control in Brazil: a systematic review 2023
132. Safety of therapeutic options for treating asthma in pregnancy 2014
133. The safety of asthma medications during pregnancy: An update for clinicians 2014
134. Pharmacological difficulties in the treatment of asthma in pregnant women 2017
135. Measures to reduce maintenance therapy with oral corticosteroid in adults with severe asthma 2016
136. Impact of omalizumab on quality-of-life outcomes in patients with moderate-to-severe allergic asthma 2006
137. Allergy and asthma prevention 2014 2014
138. Biological treatments in childhood asthma 2024
139. What’s new in the Global Initiative for Asthma 2018 report and beyond 2019
140. Outpatient pharmacotherapy for pediatric asthma 2006
141. Prospects for new and emerging therapeutics in severe asthma: the role of biologics 2017
142. Efficacy of Biologics in Reducing Exacerbations Requiring Hospitalization or an Emergency Department Visit in Patients with Moderate or Severe, Uncontrolled Asthma 2025
143. Efficacy of Biologics for Reducing Exacerbations Requiring Hospitalization or an Emergency Department Visit in Patients With Moderate-to-Severe, Uncontrolled Asthma 2024
144. Biomarker-guided asthma management: Science fiction or images of the imminent future? 2015
145. Allergic Asthma in the Era of Personalized Medicine 2022
146. House dust mite allergens, store-operated Ca2+ channels and asthma 2024
147. The safety of monoclonal antibodies in asthma 2016
148. Molecular Targets for Biological Therapies of Severe Asthma 2020
149. Exploring the Interaction between Fractional Exhaled Nitric Oxide and Biologic Treatment in Severe Asthma: A Systematic Review 2023
150. Interventions for autumn exacerbations of asthma in children 2018
151. Managing problematic severe asthma: beyond the guidelines 2018
152. A comparison of the effectiveness of biologic therapies for asthma: A systematic review and network meta-analysis 2023
153. EE489 The Cost of Treatment with Dupilumab in Severe Asthma Patients With Coexisting Other Type 2 Inflammatory Diseases 2022
154. The treatment of severe refractory asthma 2007
155. Anticholinergics for treatment of Asthma 2015
156. Impact of patient support programmes among patients with severe asthma treated with biological therapies: a systematic literature review and indirect treatment comparison 2024
157. Effect of Anti-IL5, Anti-IL5R, Anti-IL13 Therapy on Asthma Exacerbations: A Network Meta-analysis 2020
158. Cost Effectiveness of Pharmacological Treatments for Asthma: A Systematic Review 2018
159. Predictors of response to medications for asthma in pediatric patients: A systematic review of the literature 2020
160. Eosinophilic asthma 2022
161. Mepolizumab for the reduction of exacerbations in severe eosinophilic asthma 2016
162. Asthma pathophysiology and evidence-based treatment of severe exacerbations 2006
163. Benralizumab treatment reduces the high-affinity IgE receptor on plasmacytoid dendritic cells in patients with severe asthma 2019
164. Immunotherapeutic targeting of allergic disease 2006
165. Markers of severity and predictors of response to treatment in severe asthma 2022
166. Clinical remission attainment, definitions, and correlates among patients with severe asthma treated with biologics: a systematic review and meta-analysis 2025
167. Biomarkers of asthma 2022
168. Plasma protein signatures of adult asthma 2024
169. Effect of Biologics on Biomarkers of Type 2 Inflammation in Asthma: A Review of the Literature 2024
170. The patents on Glucocorticosteroids and selected new therapies for the management of asthma in children: Update 2014
171. Guidelines for collaboration among physicians, pharmacists, and managed care organizations to improve asthma outcomes 2014
172. Characterization of asthma endotypes: implications for therapy 2016
173. Evidence for lifestyle interventions in asthma 2019
174. Comparative Efficacy and Safety of Biologic Therapies in Severe Asthma: A Systematic Review and Meta-Analysis 2025
175. Diagnosis and management of work-related asthma: American College of Chest Physicians consensus statement 2008
176. PRS47 MINIMIZING THE COST OF BIOLOGIC MEDICINES IN SEVERE ASTHMA 2019
177. Determinants of cost-effectiveness results of biological therapies for severe asthma: a systematic methodological assessment 2025
178. Comparative effectiveness analysis of mAb in asthma: The importance of exacerbation definition 2015
179. Asthma and other recurrent wheezing disorders in children (chronic) 2012
180. How do biologicals and other novel therapies effect clinically used biomarkers in severe asthma? 2020
181. Real-life studies and registries of severe asthma: The advent of digital technology 2023
182. Sharp: Enabling generation of real-world evidence on a pan-European scale to improve the lives of individuals with severe asthma 2021
183. Literature in severe asthma: Bronchial thermoplasty and biological agents 2018
184. What is the optimal management option for occupational asthma? 2012
185. Incremental net monetary benefit of biologic therapies in moderate to severe asthma: a systematic review and meta-analysis of economic evaluation studies 2023
186. Long-term treatment in pediatric asthma: an update on chemical pharmacotherapy 2017
187. Consequences of long-term oral corticosteroid therapy and its side-effects in severe asthma in adults: a focused review of the impact data in the literature 2018
188. Pharmacologic therapy for the treatment of asthma in children 2004
189. Anti-IgE and chemotherapy: A critical appraisal of treatment options for severe asthma 2007
190. Efficacy and safety of Yu-Ping-Feng powder for asthma in children: a protocol of systematic review and meta-analysis of randomized controlled trials 2020
191. Management of severe asthma exacerbation in children 2011
192. Fevipiprant in the treatment of asthma 2018
193. Asthma: Diagnosis and management in adults 2016
194. Safety of long-acting beta agonists and inhaled corticosteroids in children and adolescents with asthma 2013
195. World-wide variation in the proportion of patients eligible for asthma biologics: a systematic review 2023
196. The role of the primary care physician in helping adolescent and adult patients improve asthma control 2011
197. Development of an operational definition of treatment escalation in adults with asthma adapted to healthcare administrative databases: A Delphi study 2021
198. Allergen-specific immunotherapy in pediatric allergic asthma 2016
199. Impact of allergen immunotherapy in allergic asthma 2018
200. PRS29 COMPARATIVE PHARMACOECONOMICS OF BX IN THE TREATMENT OF PATIENTS WITH UNCONTROLLED MODERATE AND SEVERE ATOPIC ASTHMA IN THE RUSSIAN HEALTH CARE SETTING 2019
201. 蔡硕 (2021). 射干麻黄汤治疗哮喘的有效性达到及对气道上皮间质转化的影响 博士, 广州中医药大学.
202. 费巧玲 (2020). 双黄连治疗实验性过敏性哮喘的作用及机制研究 博士, 中国医学科学院北京协和医学院.
203. 李博林 (2016). 基于代谢组学的冬病夏治穴位贴敷防治支气管哮喘作用机制及临床疗效研究 博士, 河北医科大学.
204. 李德容 (2012). 支气管哮喘上、下气道炎症及气道高反应性临床相关性规律研究 博士, 广州医科大学
205. 李洪涛 (2007). 糖皮质激素对哮喘树突状细胞分化、成熟和极化状态影响 博士, 中山大学.
206. 李俊 (2006). 吸入皮质激素对首次发作喘息患儿IL-4/IFN-γ失衡的影响 硕士, 中山大学.
207. 厉蓓 (2014). 补肾益气方药治疗肺肾气（阳）虚型哮喘的现代临床及实验研究 博士, 复旦大学.
208. 刘晓 (2021). 基于“气络-血络”理论与免疫细胞成分探讨“新加减定喘汤”的网络药理作用及干预儿童哮喘急性发作的临床研究 博士, 成都中医药大学.
209. 施晔 (2023). 脉冲震荡、呼出气一氧化氮及炎症指标在学龄前儿童哮喘诊断中的价值 硕士, 扬州大学.
210. 孙珺 (2009). 辨证穴位贴药治疗支气管哮喘的临床研究 硕士, 广州中医药大学.
211. 王霞 (2023). 针刺调控micro--RNA--223/NLRP3介导的细胞焦亡通路对哮喘气道炎症的影响 博士, 广州中医药大学.
212. 徐超 (2016). 儿童哮喘与幽门螺杆菌感染及其他因素的相关性研究 硕士, 山西医科大学.
213. 许婷 (2015). 转化生长因子β1在蟑螂过敏原诱导的哮喘中对间充质干细胞募集迁移的影响 博士, 南方医科大学.
214. 杨祎 (2019). 中、重度支气管哮喘的用药综合评价研究 硕士.
215. 姚慧娟 (2013). 呼出气一氧化氮诊断哮喘及哮喘患者呼出气一氧化氮与过敏原sIgE的相关性分析 硕士, 第四军医大学.
216. 哮喘儿童生命质量的影响因素 %J 中国儿童保健杂志 %J Chinese Journal of Child Health Care 2023
217. 张煦 (2012). 支气管哮喘表型分析及治疗方法探讨 硕士, 广州医科大学
218. 广州医学院.
219. 赵文静 (2024). LASSO联合Logistic回归构建可疑哮喘人群的气道高反应预测模型 硕士, 郑州大学.
220. 郑文松 (2020). 百令胶囊辅助治疗儿童哮喘的作用及其机制研究 硕士, 山东中医药大学.

**Review(n=78)**

1. FDA approval of omalizumab for the treatment of bronchial asthma 2003
2. ▼Omalizumab for severe asthma? 2006
3. Omalizumab (Xolair®) for the treatment of severe allergic asthma 2007
4. 过敏性哮喘患儿使用奥马珠单抗的有效性和安全性评估:中国真实世界中的回顾性多中心研究 %J 中华实用儿科临床杂志 2023
5. Efficacy and safety of treatment with biologicals (benralizumab, dupilumab, mepolizumab, omalizumab and reslizumab) for severe eosinophilic asthma. A systematic review for the EAACI Guidelines - recommendations on the use of biologicals in severe asthma 2020
6. Efficacy and safety of treatment with biologicals (benralizumab, dupilumab and omalizumab) for severe allergic asthma: A systematic review for the EAACI Guidelines - recommendations on the use of biologicals in severe asthma 2020
7. The effect of treatment with omalizumab, an anti-IgE antibody, on asthma exacerbations and emergency medical visits in patients with severe persistent asthma 2005
8. Effect of omalizumab on lung function and eosinophil levels in adolescents with moderate-to-severe allergic asthma 2020
9. The effect of omalizumab on lung function in adolescents with moderate to severe allergic asthma 2018
10. Update on interventions in prevention and treatment of pediatric asthma 2018
11. Omalizumab for severe allergic asthma in clinical trials and real-life studies: What we know and what we should address 2015
12. Effectiveness and Safety Studies of Omalizumab in Children and Adolescents With Moderate-To-Severe Asthma 2023
13. Omalizumab in asthma: Approval and postapproval experience 2005
14. Updated review of omalizumab to treat uncontrolled pediatric allergic asthma 2025
15. Current approaches to handling rescue medication in asthma and eczema randomized controlled trials are inadequate: a systematic review 2020
16. Anti-IgE therapy of asthma 2002
17. Comparative effectiveness of mepolizumab and omalizumab in severe asthma: An indirect treatment comparison 2017
18. Omalizumab and long-term quality of life outcomes in patients with moderate-to-severe allergic asthma: a systematic review 2019
19. Exacerbations, emergency visits, and hospitalizations in patients receiving omalizumab in real world settings: A systematic literature review of nonrandomized studies 2015
20. Role of anti-IgE monoclonal antibody (omalizumab) in the treatment of bronchial asthma and allergic respiratory diseases 2006
21. Omalizumab: A novel therapy for allergic asthma 2004
22. Subcutaneous omalizumab for people with asthma 2021
23. Effect of omalizumab on glucocorticoid use, lung function and asthma control in the treatment of allergic bronchopulmonary aspergillosis: a systematic review and meta-analysis 2022
24. Effects of Omalizumab on Blood Eosinophil Numbers in Patients with Allergic Asthma 2019
25. Efficacy of omalizumab, an anti-immunoglobulin E antibody, in patients with allergic asthma at high risk of serious asthma-related morbidity and mortality 2001
26. Efficacy of omalizumab in reducing asthma exacerbation in asian patients: A pooled analysis of two randomized placebo-controlled studies 2019
27. Effect of mepolizumab in severe eosinophilic asthma according to omalizumab eligibility 2019
28. Effect of mepolizumab on exacerbation rate in patients with severe eosinophilic asthma by omalizumab eligibility and prior omalizumab use 2018
29. Numbers needed to treat with omalizumab to prevent an asthma exacerbation, emergency room visit, or hospitalization in patients with severe uncontrolled asthma 2016
30. Pharmacoeconomic analysis of treatment of children with severe uncontrolled asthma with omalizumab in Russia 2016
31. Corrigendum: Long-term efficacy and safety of omalizumab in patients with persistent uncontrolled allergic asthma: a systematic review and meta-analysis 2015
32. Cost analysis of omalizumab use in patients with severe uncontrolled asthma within the mexican public health care system 2012
33. The discovery and development of omalizumab for the treatment of asthma 2015
34. Efficacy and safety of omalizumab in paediatric age: An update of literature data 2016
35. Omalizumab: A review of its use in patients with severe persistent allergic asthma 2013
36. [The effectiveness of omalizumab in the control of severe uncontrolled asthma in Latin America. An exploratory systematic review and meta-analysis] 2020
37. Omalizumab reduces asthma exacerbations and oral corticosteroid use in patients with severe allergic asthma: A systematic review of observational studies 2019
38. Indirect comparison of bronchial thermoplasty (BT) versus omalizumab (OM) for severe uncontrolled asthma 2015
39. Indirect comparison of bronchial thermoplasty versus omalizumab for uncontrolled severe asthma 2018
40. Omalizumab for asthma in adults and children 2014
41. Management of asthma with anti-immunoglobulin E: A review of clinical trials of omalizumab 2006
42. Approval of the anti-IgE antibody omalizumab for the treatment of severe persistent bronchial asthma 2006
43. Efficacy and safety of subcutaneous omalizumab vs placebo as add-on therapy to corticosteroids for children and adults with asthma: A systematic review 2011
44. Cost-utility of omalizumab for the treatment of uncontrolled moderate-to-severe persistent pediatric allergic asthma in a middle-income country 2021
45. Omalizumab therapy for children and adolescents with severe allergic asthma 2015
46. Omalizumab added to corticosteroids reduces exacerbations and corticosteroid use in adults and children with asthma 2011
47. Omalizumab decreases exacerbations and allows a step down in daily inhaled corticosteroid dose in adults and children with moderate-to-severe asthma 2014
48. Efficacy and effectiveness of omalizumab in the treatment of childhood asthma 2018
49. Anti-IgE for chronic asthma 2003
50. Anti-IgE for chronic asthma in adults and children 2004
51. Anti-IgE for chronic asthma in adults and children 2006
52. Bronchial thermoplasty vs omalizumab for severe uncontrolled asthma: Relative efficacy and chronicity of costs of care 2016
53. Cost-effectiveness of omalizumab for the treatment of inadequately controlled severe allergic asthma in Chinese children 2020
54. Omalizumab monotherapy reduced allergic symptoms and improved quality of life in patients with IgE-mediated food allergy: A systematic review and meta-analysis 2021
55. 血清和痰液Galectin-7表达对中度哮喘患儿奥马珠单抗治疗效果的评估价值 %J 国际检验医学杂志 2025
56. 高菲 (2022). 中重度哮喘患者应用奥马珠单抗治疗的疗效研究 硕士, 山东大学.
57. 中重度过敏性哮喘患者使用奥马珠单抗治疗1年的疗效研究 %J 中华预防医学杂志 2023
58. 奥马珠单抗治疗阿司匹林加重性呼吸系统疾病3例并文献复习 %J 中华结核和呼吸杂志 %J Chinese Journal of Tuberculosis and Respiratory Diseases 2022
59. 评估FeNO水平对奥马珠单抗在重度过敏性哮喘患者早期治疗疗效的预测能力 %J 临床肺科杂志 %J Journal of Clinical Pulmonary Medicine 2023
60. 奥马珠单抗能够降低成人及儿童中重度哮喘患者的急性发作和吸入糖皮质激素日用量 %J 英国医学杂志中文版 2014
61. 奥马珠单抗在过敏性哮喘中对哮喘和致敏的作用 %J 医药导报 2021
62. 重度哮喘使用奥马珠单抗治疗无效的患者特征 %J 实用药物与临床 2022
63. 抗IgE单克隆抗体与尘螨皮下免疫治疗在儿童支气管哮喘中的联合应用8例临床分析 %J 临床医学进展 2022
64. 奥马珠单抗治疗过敏性哮喘长期疗效和减量方案的探索 %J 中华临床免疫和变态反应杂志 2024
65. 王健 (2006). 可诱导共刺激分子重组蛋白的研制及其在过敏性哮喘炎症中的治疗作用 博士, 第二军医大学.
66. 奥马珠单抗治疗重度过敏性哮喘的药物经济学评价 %J 中国药房 %J China Pharmacy 2024
67. 奥马珠单抗治疗儿童哮喘的快速卫生技术评估 %J 中国循证医学杂志 2025
68. 奥马珠单抗治疗中重度哮喘的初步临床观察 %J 中华结核和呼吸杂志 2021
69. 奥马珠单抗治疗儿童重度过敏性哮喘的快速卫生技术评估 %J 上海医药 2020
70. Omalizumab in children with uncontrolled allergic asthma: Review of clinical trial and real-world experience 2017
71. Severe asthma new frontiers of treatment 2015
72. Biological Treatments for Severe Asthma: A Comprehensive Review 2025
73. Severe asthma and quality of life 2017
74. The association between cost sharing, prior authorization, and specialty drug utilization: A systematic review 2023
75. Women with asthma: A review of potential variables and preferred medical management 2006
76. Severe Asthma: Have we made progress? 2016
77. A bibliometric and scientific knowledge map study of the drug therapies for asthma-related study from 1982 to 2021 2022
78. 杨红 (2008). 《伤寒论》112方现代临床研究的循证医学评价 博士, 北京中医药大学.

**Deviation from the research theme(n=21)**

1. Meta-analysis of observational studies of the effectiveness of omalizumab in the control of severe allergic asthma 2015
2. SYSTEMATIC REVIEW OF OBSERVATIONAL STUDIES AND RCTS OF OMALIZUMAB IN SEVERE PERSISTENT ALLERGIC ASTHMA AND META-ANALYSIS FEASIBILITY ASSESSMENT 2014
3. Effects of omalizumab in children with asthma: A protocol for systematic review and meta-analysis 2021
4. Investigating the value of omalizumab in the treatment of severe persistent allergic asthma: A systematic review of cost-effectiveness studies 2016
5. Review: Omalizumab reduces exacerbation and steroid use in chronic asthma: Commentary 2007
6. Patient-Reported Outcomes (PROs) in Patients Receiving Omalizumab (OMB): A Systematic Literature Review 2016
7. “Real-life” Effectiveness Studies of Omalizumab in Adult Patients with Severe Allergic Asthma: Meta-analysis 2017
8. SYSTEMATIC REVIEW WITH INDIRECT COMPARISON OF EFFICACY AND SAFETY OF DUPILUMAB VERSUS OMALIZUMAB FOR SEVERE ASTHMA WITH ALLERGIC PHENOTYPE 2024
9. Real-World Effectiveness of Omalizumab in Severe Allergic Asthma: A Meta-Analysis of Observational Studies 2021
10. Efficacy and safety of omalizumab in children and adolescents with moderate-to-severe asthma: A systematic literature review 2017
11. ‘Real-world’ effectiveness of omalizumab in adults with severe allergic asthma: a meta-analysis 2021
12. Predictive biomarkers for response to omalizumab in patients with severe allergic asthma: a meta-analysis 2022
13. Short- and long-term real-world effectiveness of omalizumab in severe allergic asthma: systematic review of 42 studies published 2008-2018 2019
14. Omalizumab versus Mepolizumab as add-on therapy in asthma patients not well controlled on at least an inhaled corticosteroid: A network meta-analysis 2018
15. Omalizumab in the real-world: systematic review of asthma control outcomes reported by patients 2019
16. Omalizumab for the treatment of severe persistent allergic asthma: A systematic review and economic evaluation 2013
17. Targeted therapy for allergic asthma: Predicting and evaluating response to omalizumab 2007
18. Omalizumab to Prevent Exacerbations in Children and Adolescents with Moderate-to-Severe Allergic Asthma: An Overview of Systematic Reviews 2020
19. 陈雪琴 (2022). 奥马珠单抗治疗不同临床表型难治性哮喘的疗效和安全性：系统综述及meta分析 硕士, 南京医科大学.
20. 奥马珠单抗治疗中-重度过敏性哮喘儿童的文献分析 %J 海峡药学 2022
21. 奥马珠单抗治疗儿童中重度支气管哮喘的临床综合评价 %J 临床药物治疗杂志 %J Clinical Medication Journal 2024

**ROBIS**

P: Pass. F: Fail. L: Low risk of bias. H: High risk of bias. Un: Unclear risk of bias.

A:The conclusions of researcher Master Dongze Li.

B:The conclusions of researcher Dr. Zhuang Wang.

C:In case of a difference of opinions, it shall be adjudicated by Professor Yongji Wang.

D:Conclusive conclusion.

1.麦琳,孙蓉菲,李为民.奥马佐单抗治疗过敏性支气管哮喘疗效与安全性的系统评价[J].中国循证医学杂志,2013,13(06):709-716.

| Phase | | A | B | C | D |
| --- | --- | --- | --- | --- | --- |
| Phase 1: Assessing Relevance | | P | P | - | P |
| Phase 2: Identifying Concerns with Review Process | Study Eligibility Criteria | L | L | - | L |
|  | Identification and Selection of Studies | L | L | - | L |
|  | Data Collection and Study Appraisal | L | L | - | L |
|  | Synthesis and Findings | L | L | - | L |
| Phase 3: Judging Risk of Bias | | L | L | - | L |

2.刘长智,郑晓滨,刘茂,等.奥马珠单抗治疗难治性哮喘疗效与安全性的Meta分析[J].中华临床医师杂志(电子版),2013,7(14):6519-6525.

| Phase | | A | B | C | D |
| --- | --- | --- | --- | --- | --- |
| Phase 1: Assessing Relevance | | P | P | - | P |
| Phase 2: Identifying Concerns with Review Process | Study Eligibility Criteria | L | L | - | L |
|  | Identification and Selection of Studies | L | L | - | L |
|  | Data Collection and Study Appraisal | UN | UN | - | UN |
|  | Synthesis and Findings | L | L | - | L |
| Phase 3: Judging Risk of Bias | | L | L | - | L |

3.Lai T, Wang S, Xu Z, Zhang C, Zhao Y, Hu Y, Cao C, Ying S, Chen Z, Li W, Wu B, Shen H. Long-term efficacy and safety of omalizumab in patients with persistent uncontrolled allergic asthma: a systematic review and meta-analysis. Sci Rep. 2015 Feb 3;5:8191. doi: 10.1038/srep08191. Erratum in: Sci Rep. 2015 Aug 14;5:9548. doi: 10.1038/srep09548. PMID: 25645133; PMCID: PMC4314644.

| Phase | | A | B | C | D |
| --- | --- | --- | --- | --- | --- |
| Phase 1: Assessing Relevance | | P | P | - | P |
| Phase 2: Identifying Concerns with Review Process | Study Eligibility Criteria | L | L | - | L |
|  | Identification and Selection of Studies | L | L | - | L |
|  | Data Collection and Study Appraisal | L | L | - | L |
|  | Synthesis and Findings | L | L | - | L |
| Phase 3: Judging Risk of Bias | | L | L | - | L |

4.Rodrigo GJ, Neffen H. Systematic review on the use of omalizumab for the treatment of asthmatic children and adolescents. Pediatr Allergy Immunol. 2015 Sep;26(6):551-6. doi: 10.1111/pai.12405. Epub 2015 Jul 1. PMID: 25963882.

| Phase | | A | B | C | D |
| --- | --- | --- | --- | --- | --- |
| Phase 1: Assessing Relevance | | P | P | - | P |
| Phase 2: Identifying Concerns with Review Process | Study Eligibility Criteria | L | L | - | L |
|  | Identification and Selection of Studies | L | L | - | L |
|  | Data Collection and Study Appraisal | UN | L | L | L |
|  | Synthesis and Findings | L | L | - | L |
| Phase 3: Judging Risk of Bias | | L | L | - | L |

5.苗伟伟,汪凤凤,陈子,等.奥马珠单抗治疗难治性哮喘疗效的Meta分析[J].中国呼吸与危重监护杂志,2015,14(05):449-455.

| Phase | | A | B | C | D |
| --- | --- | --- | --- | --- | --- |
| Phase 1: Assessing Relevance | | P | P | - | P |
| Phase 2: Identifying Concerns with Review Process | Study Eligibility Criteria | L | UN | L | L |
|  | Identification and Selection of Studies | L | L | - | L |
|  | Data Collection and Study Appraisal | L | L | - | L |
|  | Synthesis and Findings | L | L | - | L |
| Phase 3: Judging Risk of Bias | | L | L | - | L |

6季春梅,孟玲. 奥马珠单抗长程治疗与短程治疗过敏性哮喘的有效性及安全性比较的meta分析[C]//2016年江苏省药学大会暨第十六届江苏省药师周 论文集. 2016:105-112.

| Phase | | A | B | C | D |
| --- | --- | --- | --- | --- | --- |
| Phase 1: Assessing Relevance | | P | P | - | P |
| Phase 2: Identifying Concerns with Review Process | Study Eligibility Criteria | L | L | - | L |
|  | Identification and Selection of Studies | L | L | - | L |
|  | Data Collection and Study Appraisal | L | L | - | L |
|  | Synthesis and Findings | L | L | - | L |
| Phase 3: Judging Risk of Bias | | L | L | - | L |

7.Corren J, Kavati A, Ortiz B, Vegesna A, Colby JA, Ruiz K, Panettieri RA Jr. Patient-reported outcomes in moderate-to-severe allergic asthmatics treated with omalizumab: a systematic literature review of randomized controlled trials. Curr Med Res Opin. 2018 Jan;34(1):65-80. doi: 10.1080/03007995.2017.1395734. Epub 2017 Nov 10. PMID: 29057669.

| Phase | | A | B | C | D |
| --- | --- | --- | --- | --- | --- |
| Phase 1: Assessing Relevance | | P | P | - | P |
| Phase 2: Identifying Concerns with Review Process | Study Eligibility Criteria | L | L | - | L |
|  | Identification and Selection of Studies | L | L | - | L |
|  | Data Collection and Study Appraisal | L | L | - | L |
|  | Synthesis and Findings | UN | L | UN | UN |
| Phase 3: Judging Risk of Bias | | L | L | - | L |

8.牟姗,张薇,江德鹏.奥马珠单抗治疗难治性变应性哮喘效果的Meta分析[J].中国医药导报,2019,16(10):75-79+99.

| Phase | | A | B | C | D |
| --- | --- | --- | --- | --- | --- |
| Phase 1: Assessing Relevance | | P | P | - | P |
| Phase 2: Identifying Concerns with Review Process | Study Eligibility Criteria | L | L | - | L |
|  | Identification and Selection of Studies | L | L | - | L |
|  | Data Collection and Study Appraisal | L | L | - | L |
|  | Synthesis and Findings | L | L | - | L |
| Phase 3: Judging Risk of Bias | | L | L | - | L |

9.Henriksen DP, Bodtger U, Sidenius K, Maltbaek N, Pedersen L, Madsen H, Andersson EA, Norgaard O, Madsen LK, Chawes BL. Efficacy of omalizumab in children, adolescents, and adults with severe allergic asthma: a systematic review, meta-analysis, and call for new trials using current guidelines for assessment of severe asthma. Allergy Asthma Clin Immunol. 2020 Jun 18;16:49. doi: 10.1186/s13223-020-00442-0. PMID: 32565844; PMCID: PMC7302157.

| Phase | | A | B | C | D |
| --- | --- | --- | --- | --- | --- |
| Phase 1: Assessing Relevance | | P | P | - | P |
| Phase 2: Identifying Concerns with Review Process | Study Eligibility Criteria | L | L | - | L |
|  | Identification and Selection of Studies | L | L | - | L |
|  | Data Collection and Study Appraisal | L | L | - | L |
|  | Synthesis and Findings | L | L | - | L |
| Phase 3: Judging Risk of Bias | | L | L | - | L |

10.Fu Z, Xu Y, Cai C. Efficacy and safety of omalizumab in children with moderate-to-severe asthma: a meta-analysis. J Asthma. 2021 Oct;58(10):1350-1358. doi: 10.1080/02770903.2020.1789875. Epub 2020 Jul 16. PMID: 32602383.

| Phase | | A | B | C | D |
| --- | --- | --- | --- | --- | --- |
| Phase 1: Assessing Relevance | | P | P | - | P |
| Phase 2: Identifying Concerns with Review Process | Study Eligibility Criteria | L | L | - | L |
|  | Identification and Selection of Studies | L | L | - | L |
|  | Data Collection and Study Appraisal | UN | L | L | L |
|  | Synthesis and Findings | L | L | - | L |
| Phase 3: Judging Risk of Bias | | UN | L | L | L |

11.姜红玉,李娟,胡文凤,等.奥马珠单抗治疗儿童中重度过敏性哮喘的有效性和安全性系统评价[J].中国医院用药评价与分析,2021,21(09):1091-1096.DOI:10.14009/j.issn.1672-2124.2021.09.017.

| Phase | | A | B | C | D |
| --- | --- | --- | --- | --- | --- |
| Phase 1: Assessing Relevance | | P | P | - | P |
| Phase 2: Identifying Concerns with Review Process | Study Eligibility Criteria | L | L | - | L |
|  | Identification and Selection of Studies | UN | L | L | L |
|  | Data Collection and Study Appraisal | UN | L | L | L |
|  | Synthesis and Findings | L | L | - | L |
| Phase 3: Judging Risk of Bias | | L | L | - | L |

12.王亚芹,樊鹏利,吕品,陈博雅,李坤,马培志.奥马珠单抗治疗儿童及青少年过敏性哮喘的系统评价[J].中国药物评价,2021,38(2):111-114

| Phase | | A | B | C | D |
| --- | --- | --- | --- | --- | --- |
| Phase 1: Assessing Relevance | | P | P | - | P |
| Phase 2: Identifying Concerns with Review Process | Study Eligibility Criteria | L | UN | L | L |
|  | Identification and Selection of Studies | L | L | - | L |
|  | Data Collection and Study Appraisal | L | L | - | L |
|  | Synthesis and Findings | L | L | - | L |
| Phase 3: Judging Risk of Bias | | L | L | - | L |

13.陈雪琴,贾心予,吴晶晶,等. 奥马珠单抗治疗难治性过敏性哮喘疗效和安全性的荟萃分析[J]. 中华医学杂志,2022,102(28):2201-2209. DOI:10.3760/cma.j.cn112137-20211109-02480.

| Phase | | A | B | C | D |
| --- | --- | --- | --- | --- | --- |
| Phase 1: Assessing Relevance | | P | P | - | P |
| Phase 2: Identifying Concerns with Review Process | Study Eligibility Criteria | L | L | - | L |
|  | Identification and Selection of Studies | L | L | - | L |
|  | Data Collection and Study Appraisal | L | L | - | L |
|  | Synthesis and Findings | L | L | - | L |
| Phase 3: Judging Risk of Bias | | L | L | - | L |

14.Fenu G, La Tessa A, Calogero C, Lombardi E. Severe pediatric asthma therapy: Omalizumab-A systematic review and meta-analysis of efficacy and safety profile. Front Pediatr. 2023 Mar 3;10:1033511. doi: 10.3389/fped.2022.1033511. PMID: 36937051; PMCID: PMC10020639.

| Phase | | A | B | C | D |
| --- | --- | --- | --- | --- | --- |
| Phase 1: Assessing Relevance | | P | P | - | P |
| Phase 2: Identifying Concerns with Review Process | Study Eligibility Criteria | L | L | - | L |
|  | Identification and Selection of Studies | L | L | - | L |
|  | Data Collection and Study Appraisal | L | L | - | L |
|  | Synthesis and Findings | L | L | - | L |
| Phase 3: Judging Risk of Bias | | L | L | - | L |

15.Lang D, Liu Z, Li D. Safety and Tolerability of Omalizumab in Children with Allergic (IgE-Mediated) Asthma: A Systematic Review and Meta-Analysis. Discov Med. 2023 Jun;35(176):233-241. doi: 10.24976/Discov.Med.202335176.24. PMID: 37272090.

| Phase | | A | B | C | D |
| --- | --- | --- | --- | --- | --- |
| Phase 1: Assessing Relevance | | P | P | - | P |
| Phase 2: Identifying Concerns with Review Process | Study Eligibility Criteria | L | L | - | L |
|  | Identification and Selection of Studies | L | L | - | L |
|  | Data Collection and Study Appraisal | L | L | - | L |
|  | Synthesis and Findings | L | L | - | L |
| Phase 3: Judging Risk of Bias | | L | L | - | L |

16.阮俊文.抗IgE单克隆抗体在支气管哮喘患者升阶梯治疗中有效性和安全性的系统评价与Meta分析[D].赣南医学院,2023.DOI:10.27959/d.cnki.ggnyx.2023.000137.

| Phase | | A | B | C | D |
| --- | --- | --- | --- | --- | --- |
| Phase 1: Assessing Relevance | | P | P | - | P |
| Phase 2: Identifying Concerns with Review Process | Study Eligibility Criteria | L | L | - | L |
|  | Identification and Selection of Studies | L | L | - | L |
|  | Data Collection and Study Appraisal | L | L | - | L |
|  | Synthesis and Findings | L | L | - | L |
| Phase 3: Judging Risk of Bias | | L | L | - | L |

17.薛宽宽.奥马珠单抗治疗中重度过敏性哮喘效果的Meta分析[D].山西医科大学,2023.DOI:10.27288/d.cnki.gsxyu.2023.000328.

| Phase | | A | B | C | D |
| --- | --- | --- | --- | --- | --- |
| Phase 1: Assessing Relevance | | P | P | - | P |
| Phase 2: Identifying Concerns with Review Process | Study Eligibility Criteria | L | L | - | L |
|  | Identification and Selection of Studies | UN | L | L | L |
|  | Data Collection and Study Appraisal | L | L | - | L |
|  | Synthesis and Findings | L | L | - | L |
| Phase 3: Judging Risk of Bias | | L | L | - | L |

18.廖浚邑.奥马珠单抗治疗对中重度过敏性哮喘患者肺功能改善的Meta分析[D].重庆医科大学,2024.DOI:10.27674/d.cnki.gcyku.2024.000768.

| Phase | | A | B | C | D |
| --- | --- | --- | --- | --- | --- |
| Phase 1: Assessing Relevance | | P | P | - | P |
| Phase 2: Identifying Concerns with Review Process | Study Eligibility Criteria | L | L | - | L |
|  | Identification and Selection of Studies | UN | L | L | L |
|  | Data Collection and Study Appraisal | L | L | - | L |
|  | Synthesis and Findings | L | L | - | L |
| Phase 3: Judging Risk of Bias | | L | L | - | L |

19.刘香.奥马珠单抗治疗儿童中重度过敏性哮喘有效性及安全性Meta分析[D].南昌大学,2024.DOI:10.27232/d.cnki.gnchu.2024.004079.

| Phase | | A | B | C | D |
| --- | --- | --- | --- | --- | --- |
| Phase 1: Assessing Relevance | | P | P | - | P |
| Phase 2: Identifying Concerns with Review Process | Study Eligibility Criteria | L | L | - | L |
|  | Identification and Selection of Studies | L | L | - | L |
|  | Data Collection and Study Appraisal | H | L | H | H |
|  | Synthesis and Findings | L | L | - | L |
| Phase 3: Judging Risk of Bias | | L | L | - | L |

**AMSTAR-2**

Y: Yes; N: No; PY: Partial Yes.

A:The conclusions of researcher Ph.D. Zhuang Wang.

B:The conclusions of researcher Master Dongze Li.

C:In case of a difference of opinions, it shall be adjudicated by Professor Yongji Wang.

D:Conclusive conclusion.

1.麦琳,孙蓉菲,李为民.奥马佐单抗治疗过敏性支气管哮喘疗效与安全性的系统评价[J].中国循证医学杂志,2013,13(06):709-716.

| Entry | | A | B | C | D |
| --- | --- | --- | --- | --- | --- |
| 1 | Did the research questions and inclusion criteria for the review include the components of PICO? | Y | Y | - | Y |
| 2 | Did the report of the review contain an explicit statement that the review methods were established prior to the conduct of the review and did the report justify any significantdeviations from the protocol? | PY | PY | - | PY |
| 3 | Did the review authors explain their selection of the study designs for inclusion in the review? | Y | Y | - | Y |
| 4 | Did the review authors use a comprehensive literature search strategy? | Y | Y | - | Y |
| 5 | Did the review authors perform study selection in duplicate? | Y | Y | - | Y |
| 6 | Did the review authors perform data extraction in duplicate? | Y | Y | - | Y |
| 7 | Did the review authors provide a list of excluded studies and justify the exclusions? | N | Y | N | N |
| 8 | Did the review authors describe the included studies in adequate detail? | Y | Y | - | Y |
| 9 | Did the review authors use a satisfactory technique for assessing the risk of bias (RoB) in individual studies that were included in the review? | Y | Y | - | Y |
| 10 | Did the review authors report on the sources of funding for the studies included in the review? | N | N | - | N |
| 11 | If meta-analysis was performed, did the review authors use appropriate methods for statistical combination of results? | Y | Y | - | Y |
| 12 | If meta-analysis was performed, did the review authors assess the potential impact of RoB in individual studies on the results of the meta-analysis or other evidence synthesis? | Y | PY | PY | PY |
| 13 | Did the review authors account for RoB in primary studies when interpreting/discussing the results of the review? | Y | PY | PY | PY |
| 14 | Did the review authors provide a satisfactory explanation for, and discussion of, any heterogeneity observed in the results of the review? | Y | Y | - | Y |
| 15 | If they performed quantitative synthesis did the review authors carry out an adequate investigation of publication bias (small study bias) and discuss its likely impact on the results of the review? | PY | Y | N | N |
| 16 | Did the review authors report any potential sources of conflict of interest, including any funding they received for conducting the review? | N | N | - | N |

2.刘长智,郑晓滨,刘茂,等.奥马珠单抗治疗难治性哮喘疗效与安全性的Meta分析[J].中华临床医师杂志(电子版),2013,7(14):6519-6525.

| Entry | | A | B | C | D |
| --- | --- | --- | --- | --- | --- |
| 1 | Did the research questions and inclusion criteria for the review include the components of PICO? | Y | Y | - | Y |
| 2 | Did the report of the review contain an explicit statement that the review methods were established prior to the conduct of the review and did the report justify any significantdeviations from the protocol? | PY | PY | - | PY |
| 3 | Did the review authors explain their selection of the study designs for inclusion in the review? | Y | Y | - | Y |
| 4 | Did the review authors use a comprehensive literature search strategy? | Y | Y | - | Y |
| 5 | Did the review authors perform study selection in duplicate? | Y | Y | - | Y |
| 6 | Did the review authors perform data extraction in duplicate? | Y | Y | - | Y |
| 7 | Did the review authors provide a list of excluded studies and justify the exclusions? | PY | Y | PY | PY |
| 8 | Did the review authors describe the included studies in adequate detail? | Y | Y | - | Y |
| 9 | Did the review authors use a satisfactory technique for assessing the risk of bias (RoB) in individual studies that were included in the review? | Y | Y | - | Y |
| 10 | Did the review authors report on the sources of funding for the studies included in the review? | N | N | - | N |
| 11 | If meta-analysis was performed, did the review authors use appropriate methods for statistical combination of results? | Y | Y | - | Y |
| 12 | If meta-analysis was performed, did the review authors assess the potential impact of RoB in individual studies on the results of the meta-analysis or other evidence synthesis? | PY | PY | - | PY |
| 13 | Did the review authors account for RoB in primary studies when interpreting/discussing the results of the review? | PY | PY | - | PY |
| 14 | Did the review authors provide a satisfactory explanation for, and discussion of, any heterogeneity observed in the results of the review? | Y | Y | - | Y |
| 15 | If they performed quantitative synthesis did the review authors carry out an adequate investigation of publication bias (small study bias) and discuss its likely impact on the results of the review? | Y | Y | - | Y |
| 16 | Did the review authors report any potential sources of conflict of interest, including any funding they received for conducting the review? | N | N | - | N |

3.Lai T, Wang S, Xu Z, Zhang C, Zhao Y, Hu Y, Cao C, Ying S, Chen Z, Li W, Wu B, Shen H. Long-term efficacy and safety of omalizumab in patients with persistent uncontrolled allergic asthma: a systematic review and meta-analysis. Sci Rep. 2015 Feb 3;5:8191. doi: 10.1038/srep08191. Erratum in: Sci Rep. 2015 Aug 14;5:9548. doi: 10.1038/srep09548. PMID: 25645133; PMCID: PMC4314644.

| Entry | | A | B | C | D |
| --- | --- | --- | --- | --- | --- |
| 1 | Did the research questions and inclusion criteria for the review include the components of PICO? | Y | Y | - | Y |
| 2 | Did the report of the review contain an explicit statement that the review methods were established prior to the conduct of the review and did the report justify any significantdeviations from the protocol? | Y | PY | Y | Y |
| 3 | Did the review authors explain their selection of the study designs for inclusion in the review? | Y | Y | - | Y |
| 4 | Did the review authors use a comprehensive literature search strategy? | Y | Y | - | Y |
| 5 | Did the review authors perform study selection in duplicate? | Y | PY | Y | Y |
| 6 | Did the review authors perform data extraction in duplicate? | Y | PY | Y | Y |
| 7 | Did the review authors provide a list of excluded studies and justify the exclusions? | Y | Y | PY | PY |
| 8 | Did the review authors describe the included studies in adequate detail? | Y | Y | - | Y |
| 9 | Did the review authors use a satisfactory technique for assessing the risk of bias (RoB) in individual studies that were included in the review? | Y | Y | - | Y |
| 10 | Did the review authors report on the sources of funding for the studies included in the review? | Y | N | N | N |
| 11 | If meta-analysis was performed, did the review authors use appropriate methods for statistical combination of results? | Y | Y | - | Y |
| 12 | If meta-analysis was performed, did the review authors assess the potential impact of RoB in individual studies on the results of the meta-analysis or other evidence synthesis? | Y | PY | Y | Y |
| 13 | Did the review authors account for RoB in primary studies when interpreting/discussing the results of the review? | Y | PY | Y | Y |
| 14 | Did the review authors provide a satisfactory explanation for, and discussion of, any heterogeneity observed in the results of the review? | Y | PY | Y | Y |
| 15 | If they performed quantitative synthesis did the review authors carry out an adequate investigation of publication bias (small study bias) and discuss its likely impact on the results of the review? | Y | N | Y | Y |
| 16 | Did the review authors report any potential sources of conflict of interest, including any funding they received for conducting the review? | Y | Y | - | Y |

4.Rodrigo GJ, Neffen H. Systematic review on the use of omalizumab for the treatment of asthmatic children and adolescents. Pediatr Allergy Immunol. 2015 Sep;26(6):551-6. doi: 10.1111/pai.12405. Epub 2015 Jul 1. PMID: 25963882.

| Entry | | A | B | C | D |
| --- | --- | --- | --- | --- | --- |
| 1 | Did the research questions and inclusion criteria for the review include the components of PICO? | Y | Y | - | Y |
| 2 | Did the report of the review contain an explicit statement that the review methods were established prior to the conduct of the review and did the report justify any significantdeviations from the protocol? | Y | N | PY | PY |
| 3 | Did the review authors explain their selection of the study designs for inclusion in the review? | Y | Y | - | Y |
| 4 | Did the review authors use a comprehensive literature search strategy? | Y | Y | - | Y |
| 5 | Did the review authors perform study selection in duplicate? | Y | Y | - | Y |
| 6 | Did the review authors perform data extraction in duplicate? | Y | Y | - | Y |
| 7 | Did the review authors provide a list of excluded studies and justify the exclusions? | Y | Y | - | Y |
| 8 | Did the review authors describe the included studies in adequate detail? | Y | Y | - | Y |
| 9 | Did the review authors use a satisfactory technique for assessing the risk of bias (RoB) in individual studies that were included in the review? | Y | Y | - | Y |
| 10 | Did the review authors report on the sources of funding for the studies included in the review? | Y | Y | - | Y |
| 11 | If meta-analysis was performed, did the review authors use appropriate methods for statistical combination of results? | Y | Y | - | Y |
| 12 | If meta-analysis was performed, did the review authors assess the potential impact of RoB in individual studies on the results of the meta-analysis or other evidence synthesis? | Y | Y | - | Y |
| 13 | Did the review authors account for RoB in primary studies when interpreting/discussing the results of the review? | Y | Y | - | Y |
| 14 | Did the review authors provide a satisfactory explanation for, and discussion of, any heterogeneity observed in the results of the review? | Y | Y | - | Y |
| 15 | If they performed quantitative synthesis did the review authors carry out an adequate investigation of publication bias (small study bias) and discuss its likely impact on the results of the review? | PY | N | N | N |
| 16 | Did the review authors report any potential sources of conflict of interest, including any funding they received for conducting the review? | Y | PY | Y | Y |

5.苗伟伟,汪凤凤,陈子,等.奥马珠单抗治疗难治性哮喘疗效的Meta分析[J].中国呼吸与危重监护杂志,2015,14(05):449-455.

| Entry | | A | B | C | D |
| --- | --- | --- | --- | --- | --- |
| 1 | Did the research questions and inclusion criteria for the review include the components of PICO? | Y | Y | - | Y |
| 2 | Did the report of the review contain an explicit statement that the review methods were established prior to the conduct of the review and did the report justify any significantdeviations from the protocol? | PY | PY | - | PY |
| 3 | Did the review authors explain their selection of the study designs for inclusion in the review? | Y | Y | - | Y |
| 4 | Did the review authors use a comprehensive literature search strategy? | Y | Y | - | Y |
| 5 | Did the review authors perform study selection in duplicate? | Y | Y | - | Y |
| 6 | Did the review authors perform data extraction in duplicate? | Y | Y | - | Y |
| 7 | Did the review authors provide a list of excluded studies and justify the exclusions? | Y | Y | - | Y |
| 8 | Did the review authors describe the included studies in adequate detail? | Y | Y | - | Y |
| 9 | Did the review authors use a satisfactory technique for assessing the risk of bias (RoB) in individual studies that were included in the review? | Y | Y | - | Y |
| 10 | Did the review authors report on the sources of funding for the studies included in the review? | N | N | - | N |
| 11 | If meta-analysis was performed, did the review authors use appropriate methods for statistical combination of results? | Y | Y | - | Y |
| 12 | If meta-analysis was performed, did the review authors assess the potential impact of RoB in individual studies on the results of the meta-analysis or other evidence synthesis? | Y | PY | PY | PY |
| 13 | Did the review authors account for RoB in primary studies when interpreting/discussing the results of the review? | Y | PY | Y | Y |
| 14 | Did the review authors provide a satisfactory explanation for, and discussion of, any heterogeneity observed in the results of the review? | Y | Y | - | Y |
| 15 | If they performed quantitative synthesis did the review authors carry out an adequate investigation of publication bias (small study bias) and discuss its likely impact on the results of the review? | Y | Y | - | Y |
| 16 | Did the review authors report any potential sources of conflict of interest, including any funding they received for conducting the review? | N | N | - | N |

6.季春梅,孟玲. 奥马珠单抗长程治疗与短程治疗过敏性哮喘的有效性及安全性比较的meta分析[C]//2016年江苏省药学大会暨第十六届江苏省药师周 论文集. 2016:105-112.

| Entry | | A | B | C | D |
| --- | --- | --- | --- | --- | --- |
| 1 | Did the research questions and inclusion criteria for the review include the components of PICO? | Y | Y | - | Y |
| 2 | Did the report of the review contain an explicit statement that the review methods were established prior to the conduct of the review and did the report justify any significantdeviations from the protocol? | Y | N | PY | PY |
| 3 | Did the review authors explain their selection of the study designs for inclusion in the review? | Y | Y | - | Y |
| 4 | Did the review authors use a comprehensive literature search strategy? | Y | Y | - | Y |
| 5 | Did the review authors perform study selection in duplicate? | Y | Y | - | Y |
| 6 | Did the review authors perform data extraction in duplicate? | Y | Y | - | Y |
| 7 | Did the review authors provide a list of excluded studies and justify the exclusions? | Y | Y | - | Y |
| 8 | Did the review authors describe the included studies in adequate detail? | Y | Y | - | Y |
| 9 | Did the review authors use a satisfactory technique for assessing the risk of bias (RoB) in individual studies that were included in the review? | Y | Y | - | Y |
| 10 | Did the review authors report on the sources of funding for the studies included in the review? | N | N | - | N |
| 11 | If meta-analysis was performed, did the review authors use appropriate methods for statistical combination of results? | Y | Y | - | Y |
| 12 | If meta-analysis was performed, did the review authors assess the potential impact of RoB in individual studies on the results of the meta-analysis or other evidence synthesis? | Y | Y | - | Y |
| 13 | Did the review authors account for RoB in primary studies when interpreting/discussing the results of the review? | Y | Y | - | Y |
| 14 | Did the review authors provide a satisfactory explanation for, and discussion of, any heterogeneity observed in the results of the review? | Y | Y | - | Y |
| 15 | If they performed quantitative synthesis did the review authors carry out an adequate investigation of publication bias (small study bias) and discuss its likely impact on the results of the review? | Y | Y | - | Y |
| 16 | Did the review authors report any potential sources of conflict of interest, including any funding they received for conducting the review? | Y | Y | - | Y |

7.Corren J, Kavati A, Ortiz B, Vegesna A, Colby JA, Ruiz K, Panettieri RA Jr. Patient-reported outcomes in moderate-to-severe allergic asthmatics treated with omalizumab: a systematic literature review of randomized controlled trials. Curr Med Res Opin. 2018 Jan;34(1):65-80. doi: 10.1080/03007995.2017.1395734. Epub 2017 Nov 10. PMID: 29057669.

| Entry | | A | B | C | D |
| --- | --- | --- | --- | --- | --- |
| 1 | Did the research questions and inclusion criteria for the review include the components of PICO? | Y | Y | - | Y |
| 2 | Did the report of the review contain an explicit statement that the review methods were established prior to the conduct of the review and did the report justify any significantdeviations from the protocol? | Y | PY | PY | PY |
| 3 | Did the review authors explain their selection of the study designs for inclusion in the review? | Y | Y | - | Y |
| 4 | Did the review authors use a comprehensive literature search strategy? | Y | Y | - | Y |
| 5 | Did the review authors perform study selection in duplicate? | Y | Y | - | Y |
| 6 | Did the review authors perform data extraction in duplicate? | Y | Y | - | Y |
| 7 | Did the review authors provide a list of excluded studies and justify the exclusions? | Y | Y | - | Y |
| 8 | Did the review authors describe the included studies in adequate detail? | Y | Y | - | Y |
| 9 | Did the review authors use a satisfactory technique for assessing the risk of bias (RoB) in individual studies that were included in the review? | Y | Y | - | Y |
| 10 | Did the review authors report on the sources of funding for the studies included in the review? | Y | N | N | N |
| 11 | If meta-analysis was performed, did the review authors use appropriate methods for statistical combination of results? | PY | N | N | N |
| 12 | If meta-analysis was performed, did the review authors assess the potential impact of RoB in individual studies on the results of the meta-analysis or other evidence synthesis? | PY | N | N | N |
| 13 | Did the review authors account for RoB in primary studies when interpreting/discussing the results of the review? | Y | Y | - | Y |
| 14 | Did the review authors provide a satisfactory explanation for, and discussion of, any heterogeneity observed in the results of the review? | Y | PY | PY | PY |
| 15 | If they performed quantitative synthesis did the review authors carry out an adequate investigation of publication bias (small study bias) and discuss its likely impact on the results of the review? | PY | N | N | N |
| 16 | Did the review authors report any potential sources of conflict of interest, including any funding they received for conducting the review? | Y | Y | - | Y |

8.牟姗,张薇,江德鹏.奥马珠单抗治疗难治性变应性哮喘效果的Meta分析[J].中国医药导报,2019,16(10):75-79+99.

| Entry | | A | B | C | D |
| --- | --- | --- | --- | --- | --- |
| 1 | Did the research questions and inclusion criteria for the review include the components of PICO? | Y | Y | - | Y |
| 2 | Did the report of the review contain an explicit statement that the review methods were established prior to the conduct of the review and did the report justify any significantdeviations from the protocol? | Y | Y | - | Y |
| 3 | Did the review authors explain their selection of the study designs for inclusion in the review? | Y | Y | - | Y |
| 4 | Did the review authors use a comprehensive literature search strategy? | Y | Y | - | Y |
| 5 | Did the review authors perform study selection in duplicate? | Y | Y | - | Y |
| 6 | Did the review authors perform data extraction in duplicate? | Y | Y | - | Y |
| 7 | Did the review authors provide a list of excluded studies and justify the exclusions? | Y | Y | - | Y |
| 8 | Did the review authors describe the included studies in adequate detail? | Y | Y | - | Y |
| 9 | Did the review authors use a satisfactory technique for assessing the risk of bias (RoB) in individual studies that were included in the review? | Y | Y | - | Y |
| 10 | Did the review authors report on the sources of funding for the studies included in the review? | PY | N | PY | PY |
| 11 | If meta-analysis was performed, did the review authors use appropriate methods for statistical combination of results? | Y | Y | - | Y |
| 12 | If meta-analysis was performed, did the review authors assess the potential impact of RoB in individual studies on the results of the meta-analysis or other evidence synthesis? | Y | Y | - | Y |
| 13 | Did the review authors account for RoB in primary studies when interpreting/discussing the results of the review? | Y | Y | - | Y |
| 14 | Did the review authors provide a satisfactory explanation for, and discussion of, any heterogeneity observed in the results of the review? | Y | Y | - | Y |
| 15 | If they performed quantitative synthesis did the review authors carry out an adequate investigation of publication bias (small study bias) and discuss its likely impact on the results of the review? | Y | Y | - | Y |
| 16 | Did the review authors report any potential sources of conflict of interest, including any funding they received for conducting the review? | PY | N | PY | PY |

9.Henriksen DP, Bodtger U, Sidenius K, Maltbaek N, Pedersen L, Madsen H, Andersson EA, Norgaard O, Madsen LK, Chawes BL. Efficacy of omalizumab in children, adolescents, and adults with severe allergic asthma: a systematic review, meta-analysis, and call for new trials using current guidelines for assessment of severe asthma. Allergy Asthma Clin Immunol. 2020 Jun 18;16:49. doi: 10.1186/s13223-020-00442-0. PMID: 32565844; PMCID: PMC7302157.

| Entry | | A | B | C | D |
| --- | --- | --- | --- | --- | --- |
| 1 | Did the research questions and inclusion criteria for the review include the components of PICO? | Y | Y | - | Y |
| 2 | Did the report of the review contain an explicit statement that the review methods were established prior to the conduct of the review and did the report justify any significantdeviations from the protocol? | Y | Y | - | Y |
| 3 | Did the review authors explain their selection of the study designs for inclusion in the review? | Y | Y | - | Y |
| 4 | Did the review authors use a comprehensive literature search strategy? | Y | Y | - | Y |
| 5 | Did the review authors perform study selection in duplicate? | Y | Y | - | Y |
| 6 | Did the review authors perform data extraction in duplicate? | Y | Y | - | Y |
| 7 | Did the review authors provide a list of excluded studies and justify the exclusions? | Y | Y | - | Y |
| 8 | Did the review authors describe the included studies in adequate detail? | Y | Y | - | Y |
| 9 | Did the review authors use a satisfactory technique for assessing the risk of bias (RoB) in individual studies that were included in the review? | Y | Y | - | Y |
| 10 | Did the review authors report on the sources of funding for the studies included in the review? | PY | N | PY | PY |
| 11 | If meta-analysis was performed, did the review authors use appropriate methods for statistical combination of results? | Y | Y | - | Y |
| 12 | If meta-analysis was performed, did the review authors assess the potential impact of RoB in individual studies on the results of the meta-analysis or other evidence synthesis? | Y | Y | - | Y |
| 13 | Did the review authors account for RoB in primary studies when interpreting/discussing the results of the review? | Y | Y | - | Y |
| 14 | Did the review authors provide a satisfactory explanation for, and discussion of, any heterogeneity observed in the results of the review? | Y | Y | - | Y |
| 15 | If they performed quantitative synthesis did the review authors carry out an adequate investigation of publication bias (small study bias) and discuss its likely impact on the results of the review? | PY | N | PY | PY |
| 16 | Did the review authors report any potential sources of conflict of interest, including any funding they received for conducting the review? | Y | Y | - | Y |

10.Fu Z, Xu Y, Cai C. Efficacy and safety of omalizumab in children with moderate-to-severe asthma: a meta-analysis. J Asthma. 2021 Oct;58(10):1350-1358. doi: 10.1080/02770903.2020.1789875. Epub 2020 Jul 16. PMID: 32602383.

| Entry | | A | B | C | D |
| --- | --- | --- | --- | --- | --- |
| 1 | Did the research questions and inclusion criteria for the review include the components of PICO? | Y | Y | - | Y |
| 2 | Did the report of the review contain an explicit statement that the review methods were established prior to the conduct of the review and did the report justify any significantdeviations from the protocol? | PY | N | PY | PY |
| 3 | Did the review authors explain their selection of the study designs for inclusion in the review? | Y | Y | - | Y |
| 4 | Did the review authors use a comprehensive literature search strategy? | Y | Y | - | Y |
| 5 | Did the review authors perform study selection in duplicate? | Y | Y | - | Y |
| 6 | Did the review authors perform data extraction in duplicate? | Y | Y | - | Y |
| 7 | Did the review authors provide a list of excluded studies and justify the exclusions? | Y | Y | - | Y |
| 8 | Did the review authors describe the included studies in adequate detail? | Y | Y | - | Y |
| 9 | Did the review authors use a satisfactory technique for assessing the risk of bias (RoB) in individual studies that were included in the review? | PY | Y | PY | PY |
| 10 | Did the review authors report on the sources of funding for the studies included in the review? | N | N | - | N |
| 11 | If meta-analysis was performed, did the review authors use appropriate methods for statistical combination of results? | Y | Y | - | Y |
| 12 | If meta-analysis was performed, did the review authors assess the potential impact of RoB in individual studies on the results of the meta-analysis or other evidence synthesis? | PY | Y | PY | PY |
| 13 | Did the review authors account for RoB in primary studies when interpreting/discussing the results of the review? | PY | Y | PY | PY |
| 14 | Did the review authors provide a satisfactory explanation for, and discussion of, any heterogeneity observed in the results of the review? | Y | Y | - | Y |
| 15 | If they performed quantitative synthesis did the review authors carry out an adequate investigation of publication bias (small study bias) and discuss its likely impact on the results of the review? | Y | Y | - | Y |
| 16 | Did the review authors report any potential sources of conflict of interest, including any funding they received for conducting the review? | Y | Y | - | Y |

11.姜红玉,李娟,胡文凤,等.奥马珠单抗治疗儿童中重度过敏性哮喘的有效性和安全性系统评价[J].中国医院用药评价与分析,2021,21(09):1091-1096.DOI:10.14009/j.issn.1672-2124.2021.09.017.

| Entry | | A | B | C | D |
| --- | --- | --- | --- | --- | --- |
| 1 | Did the research questions and inclusion criteria for the review include the components of PICO? | Y | Y | - | Y |
| 2 | Did the report of the review contain an explicit statement that the review methods were established prior to the conduct of the review and did the report justify any significantdeviations from the protocol? | Y | PY | N | N |
| 3 | Did the review authors explain their selection of the study designs for inclusion in the review? | Y | Y | - | Y |
| 4 | Did the review authors use a comprehensive literature search strategy? | Y | Y | - | Y |
| 5 | Did the review authors perform study selection in duplicate? | Y | Y | - | Y |
| 6 | Did the review authors perform data extraction in duplicate? | PY | Y | Y | Y |
| 7 | Did the review authors provide a list of excluded studies and justify the exclusions? | PY | Y | PY | PY |
| 8 | Did the review authors describe the included studies in adequate detail? | Y | Y | - | Y |
| 9 | Did the review authors use a satisfactory technique for assessing the risk of bias (RoB) in individual studies that were included in the review? | Y | Y | - | Y |
| 10 | Did the review authors report on the sources of funding for the studies included in the review? | N | N | - | N |
| 11 | If meta-analysis was performed, did the review authors use appropriate methods for statistical combination of results? | Y | Y | - | Y |
| 12 | If meta-analysis was performed, did the review authors assess the potential impact of RoB in individual studies on the results of the meta-analysis or other evidence synthesis? | Y | Y | - | PY |
| 13 | Did the review authors account for RoB in primary studies when interpreting/discussing the results of the review? | Y | Y | - | PY |
| 14 | Did the review authors provide a satisfactory explanation for, and discussion of, any heterogeneity observed in the results of the review? | Y | Y | - | PY |
| 15 | If they performed quantitative synthesis did the review authors carry out an adequate investigation of publication bias (small study bias) and discuss its likely impact on the results of the review? | PY | N | N | N |
| 16 | Did the review authors report any potential sources of conflict of interest, including any funding they received for conducting the review? | N | N | - | N |

12.王亚芹,樊鹏利,吕品,陈博雅,李坤,马培志.奥马珠单抗治疗儿童及青少年过敏性哮喘的系统评价[J].中国药物评价,2021,38(2):111-114

| Entry | | A | B | C | D |
| --- | --- | --- | --- | --- | --- |
| 1 | Did the research questions and inclusion criteria for the review include the components of PICO? | Y | Y | - | Y |
| 2 | Did the report of the review contain an explicit statement that the review methods were established prior to the conduct of the review and did the report justify any significantdeviations from the protocol? | PY | PY | - | PY |
| 3 | Did the review authors explain their selection of the study designs for inclusion in the review? | Y | Y | - | Y |
| 4 | Did the review authors use a comprehensive literature search strategy? | Y | Y | - | Y |
| 5 | Did the review authors perform study selection in duplicate? | Y | Y | - | Y |
| 6 | Did the review authors perform data extraction in duplicate? | PY | Y | Y | Y |
| 7 | Did the review authors provide a list of excluded studies and justify the exclusions? | PY | Y | PY | PY |
| 8 | Did the review authors describe the included studies in adequate detail? | Y | Y | - | Y |
| 9 | Did the review authors use a satisfactory technique for assessing the risk of bias (RoB) in individual studies that were included in the review? | Y | Y | - | Y |
| 10 | Did the review authors report on the sources of funding for the studies included in the review? | N | N | - | N |
| 11 | If meta-analysis was performed, did the review authors use appropriate methods for statistical combination of results? | Y | N | Y | Y |
| 12 | If meta-analysis was performed, did the review authors assess the potential impact of RoB in individual studies on the results of the meta-analysis or other evidence synthesis? | PY | PY | - | PY |
| 13 | Did the review authors account for RoB in primary studies when interpreting/discussing the results of the review? | PY | PY | - | PY |
| 14 | Did the review authors provide a satisfactory explanation for, and discussion of, any heterogeneity observed in the results of the review? | Y | PY | PY | PY |
| 15 | If they performed quantitative synthesis did the review authors carry out an adequate investigation of publication bias (small study bias) and discuss its likely impact on the results of the review? | N | N | - | N |
| 16 | Did the review authors report any potential sources of conflict of interest, including any funding they received for conducting the review? | N | Y | Y | Y |

13.陈雪琴,贾心予,吴晶晶,等. 奥马珠单抗治疗难治性过敏性哮喘疗效和安全性的荟萃分析[J]. 中华医学杂志,2022,102(28):2201-2209. DOI:10.3760/cma.j.cn112137-20211109-02480.

| Entry | | A | B | C | D |
| --- | --- | --- | --- | --- | --- |
| 1 | Did the research questions and inclusion criteria for the review include the components of PICO? | Y | Y | - | Y |
| 2 | Did the report of the review contain an explicit statement that the review methods were established prior to the conduct of the review and did the report justify any significantdeviations from the protocol? | Y | PY | PY | PY |
| 3 | Did the review authors explain their selection of the study designs for inclusion in the review? | Y | Y | - | Y |
| 4 | Did the review authors use a comprehensive literature search strategy? | Y | Y | - | Y |
| 5 | Did the review authors perform study selection in duplicate? | Y | Y | - | Y |
| 6 | Did the review authors perform data extraction in duplicate? | Y | Y | - | Y |
| 7 | Did the review authors provide a list of excluded studies and justify the exclusions? | Y | Y | - | Y |
| 8 | Did the review authors describe the included studies in adequate detail? | Y | Y | - | Y |
| 9 | Did the review authors use a satisfactory technique for assessing the risk of bias (RoB) in individual studies that were included in the review? | Y | Y | - | Y |
| 10 | Did the review authors report on the sources of funding for the studies included in the review? | N | N | - | N |
| 11 | If meta-analysis was performed, did the review authors use appropriate methods for statistical combination of results? | Y | Y | - | Y |
| 12 | If meta-analysis was performed, did the review authors assess the potential impact of RoB in individual studies on the results of the meta-analysis or other evidence synthesis? | Y | Y | - | Y |
| 13 | Did the review authors account for RoB in primary studies when interpreting/discussing the results of the review? | Y | Y | - | Y |
| 14 | Did the review authors provide a satisfactory explanation for, and discussion of, any heterogeneity observed in the results of the review? | Y | Y | - | Y |
| 15 | If they performed quantitative synthesis did the review authors carry out an adequate investigation of publication bias (small study bias) and discuss its likely impact on the results of the review? | Y | Y | - | Y |
| 16 | Did the review authors report any potential sources of conflict of interest, including any funding they received for conducting the review? | Y | Y | - | Y |

14.Fenu G, La Tessa A, Calogero C, Lombardi E. Severe pediatric asthma therapy: Omalizumab-A systematic review and meta-analysis of efficacy and safety profile. Front Pediatr. 2023 Mar 3;10:1033511. doi: 10.3389/fped.2022.1033511. PMID: 36937051; PMCID: PMC10020639.

| Entry | | A | B | C | D |
| --- | --- | --- | --- | --- | --- |
| 1 | Did the research questions and inclusion criteria for the review include the components of PICO? | Y | Y | - | Y |
| 2 | Did the report of the review contain an explicit statement that the review methods were established p15rior to the conduct of the review and did the report justify any significantdeviations from the protocol? | Y | PY | Y | Y |
| 3 | Did the review authors explain their selection of the study designs for inclusion in the review? | Y | Y | - | Y |
| 4 | Did the review authors use a comprehensive literature search strategy? | Y | Y | - | Y |
| 5 | Did the review authors perform study selection in duplicate? | PY | N | PY | PY |
| 6 | Did the review authors perform data extraction in duplicate? | PY | N | PY | PY |
| 7 | Did the review authors provide a list of excluded studies and justify the exclusions? | Y | Y | - | Y |
| 8 | Did the review authors describe the included studies in adequate detail? | Y | Y | - | Y |
| 9 | Did the review authors use a satisfactory technique for assessing the risk of bias (RoB) in individual studies that were included in the review? | Y | Y | - | Y |
| 10 | Did the review authors report on the sources of funding for the studies included in the review? | Y | N | Y | Y |
| 11 | If meta-analysis was performed, did the review authors use appropriate methods for statistical combination of results? | Y | Y | - | Y |
| 12 | If meta-analysis was performed, did the review authors assess the potential impact of RoB in individual studies on the results of the meta-analysis or other evidence synthesis? | Y | PY | Y | Y |
| 13 | Did the review authors account for RoB in primary studies when interpreting/discussing the results of the review? | Y | PY | Y | Y |
| 14 | Did the review authors provide a satisfactory explanation for, and discussion of, any heterogeneity observed in the results of the review? | Y | Y | - | Y |
| 15 | If they performed quantitative synthesis did the review authors carry out an adequate investigation of publication bias (small study bias) and discuss its likely impact on the results of the review? | Y | N | N | N |
| 16 | Did the review authors report any potential sources of conflict of interest, including any funding they received for conducting the review? | Y | Y | - | Y |

15.Lang D, Liu Z, Li D. Safety and Tolerability of Omalizumab in Children with Allergic (IgE-Mediated) Asthma: A Systematic Review and Meta-Analysis. Discov Med. 2023 Jun;35(176):233-241. doi: 10.24976/Discov.Med.202335176.24. PMID: 37272090.

| Entry | | A | B | C | D |
| --- | --- | --- | --- | --- | --- |
| 1 | Did the research questions and inclusion criteria for the review include the components of PICO? | Y | Y | - | Y |
| 2 | Did the report of the review contain an explicit statement that the review methods were established prior to the conduct of the review and did the report justify any significantdeviations from the protocol? | Y | N | PY | PY |
| 3 | Did the review authors explain their selection of the study designs for inclusion in the review? | Y | Y | - | Y |
| 4 | Did the review authors use a comprehensive literature search strategy? | Y | Y | - | Y |
| 5 | Did the review authors perform study selection in duplicate? | Y | Y | - | Y |
| 6 | Did the review authors perform data extraction in duplicate? | Y | Y | - | Y |
| 7 | Did the review authors provide a list of excluded studies and justify the exclusions? | Y | Y | - | Y |
| 8 | Did the review authors describe the included studies in adequate detail? | Y | Y | - | Y |
| 9 | Did the review authors use a satisfactory technique for assessing the risk of bias (RoB) in individual studies that were included in the review? | Y | Y | - | Y |
| 10 | Did the review authors report on the sources of funding for the studies included in the review? | PY | N | PY | PY |
| 11 | If meta-analysis was performed, did the review authors use appropriate methods for statistical combination of results? | Y | Y | - | Y |
| 12 | If meta-analysis was performed, did the review authors assess the potential impact of RoB in individual studies on the results of the meta-analysis or other evidence synthesis? | Y | PY | Y | Y |
| 13 | Did the review authors account for RoB in primary studies when interpreting/discussing the results of the review? | Y | PY | Y | Y |
| 14 | Did the review authors provide a satisfactory explanation for, and discussion of, any heterogeneity observed in the results of the review? | Y | PY | Y | Y |
| 15 | If they performed quantitative synthesis did the review authors carry out an adequate investigation of publication bias (small study bias) and discuss its likely impact on the results of the review? | Y | N | Y | Y |
| 16 | Did the review authors report any potential sources of conflict of interest, including any funding they received for conducting the review? | Y | Y | - | Y |

16.阮俊文.抗IgE单克隆抗体在支气管哮喘患者升阶梯治疗中有效性和安全性的系统评价与Meta分析[D].赣南医学院,2023.DOI:10.27959/d.cnki.ggnyx.2023.000137.

| Entry | | A | B | C | D |
| --- | --- | --- | --- | --- | --- |
| 1 | Did the research questions and inclusion criteria for the review include the components of PICO? | Y | Y | - | Y |
| 2 | Did the report of the review contain an explicit statement that the review methods were established prior to the conduct of the review and did the report justify any significantdeviations from the protocol? | PY | PY | - | PY |
| 3 | Did the review authors explain their selection of the study designs for inclusion in the review? | Y | Y | - | Y |
| 4 | Did the review authors use a comprehensive literature search strategy? | Y | Y | - | Y |
| 5 | Did the review authors perform study selection in duplicate? | Y | PY | Y | Y |
| 6 | Did the review authors perform data extraction in duplicate? | PY | PY | - | PY |
| 7 | Did the review authors provide a list of excluded studies and justify the exclusions? | Y | Y | - | Y |
| 8 | Did the review authors describe the included studies in adequate detail? | Y | Y | - | Y |
| 9 | Did the review authors use a satisfactory technique for assessing the risk of bias (RoB) in individual studies that were included in the review? | Y | Y | - | Y |
| 10 | Did the review authors report on the sources of funding for the studies included in the review? | N | N | - | N |
| 11 | If meta-analysis was performed, did the review authors use appropriate methods for statistical combination of results? | Y | Y | - | Y |
| 12 | If meta-analysis was performed, did the review authors assess the potential impact of RoB in individual studies on the results of the meta-analysis or other evidence synthesis? | Y | Y | - | Y |
| 13 | Did the review authors account for RoB in primary studies when interpreting/discussing the results of the review? | Y | Y | - | Y |
| 14 | Did the review authors provide a satisfactory explanation for, and discussion of, any heterogeneity observed in the results of the review? | Y | Y | - | Y |
| 15 | If they performed quantitative synthesis did the review authors carry out an adequate investigation of publication bias (small study bias) and discuss its likely impact on the results of the review? | Y | Y | - | Y |
| 16 | Did the review authors report any potential sources of conflict of interest, including any funding they received for conducting the review? | N | N | - | N |

17.薛宽宽.奥马珠单抗治疗中重度过敏性哮喘效果的Meta分析[D].山西医科大学,2023.DOI:10.27288/d.cnki.gsxyu.2023.000328.

| Entry | | A | B | C | D |
| --- | --- | --- | --- | --- | --- |
| 1 | Did the research questions and inclusion criteria for the review include the components of PICO? | PY | Y | Y | Y |
| 2 | Did the report of the review contain an explicit statement that the review methods were established prior to the conduct of the review and did the report justify any significantdeviations from the protocol? | N | PY | PY | PY |
| 3 | Did the review authors explain their selection of the study designs for inclusion in the review? | Y | Y | - | Y |
| 4 | Did the review authors use a comprehensive literature search strategy? | Y | Y | - | Y |
| 5 | Did the review authors perform study selection in duplicate? | Y | Y | - | Y |
| 6 | Did the review authors perform data extraction in duplicate? | Y | Y | - | Y |
| 7 | Did the review authors provide a list of excluded studies and justify the exclusions? | Y | Y | - | Y |
| 8 | Did the review authors describe the included studies in adequate detail? | Y | Y | - | Y |
| 9 | Did the review authors use a satisfactory technique for assessing the risk of bias (RoB) in individual studies that were included in the review? | Y | Y | - | Y |
| 10 | Did the review authors report on the sources of funding for the studies included in the review? | N | N | - | N |
| 11 | If meta-analysis was performed, did the review authors use appropriate methods for statistical combination of results? | Y | Y | - | Y |
| 12 | If meta-analysis was performed, did the review authors assess the potential impact of RoB in individual studies on the results of the meta-analysis or other evidence synthesis? | Y | Y | - | Y |
| 13 | Did the review authors account for RoB in primary studies when interpreting/discussing the results of the review? | Y | Y | - | Y |
| 14 | Did the review authors provide a satisfactory explanation for, and discussion of, any heterogeneity observed in the results of the review? | Y | Y | - | Y |
| 15 | If they performed quantitative synthesis did the review authors carry out an adequate investigation of publication bias (small study bias) and discuss its likely impact on the results of the review? | Y | PY | PY | PY |
| 16 | Did the review authors report any potential sources of conflict of interest, including any funding they received for conducting the review? | N | N | - | N |

18.廖浚邑.奥马珠单抗治疗对中重度过敏性哮喘患者肺功能改善的Meta分析[D].重庆医科大学,2024.DOI:10.27674/d.cnki.gcyku.2024.000768.

| Entry | | A | B | C | D |
| --- | --- | --- | --- | --- | --- |
| 1 | Did the research questions and inclusion criteria for the review include the components of PICO? | Y | Y | - | Y |
| 2 | Did the report of the review contain an explicit statement that the review methods were established prior to the conduct of the review and did the report justify any significantdeviations from the protocol? | PY | PY | - | PY |
| 3 | Did the review authors explain their selection of the study designs for inclusion in the review? | Y | Y | - | Y |
| 4 | Did the review authors use a comprehensive literature search strategy? | Y | Y | - | Y |
| 5 | Did the review authors perform study selection in duplicate? | Y | Y | - | Y |
| 6 | Did the review authors perform data extraction in duplicate? | Y | Y | - | Y |
| 7 | Did the review authors provide a list of excluded studies and justify the exclusions? | PY | Y | PY | PY |
| 8 | Did the review authors describe the included studies in adequate detail? | Y | Y | - | Y |
| 9 | Did the review authors use a satisfactory technique for assessing the risk of bias (RoB) in individual studies that were included in the review? | Y | Y | - | Y |
| 10 | Did the review authors report on the sources of funding for the studies included in the review? | N | N | - | N |
| 11 | If meta-analysis was performed, did the review authors use appropriate methods for statistical combination of results? | Y | Y | - | Y |
| 12 | If meta-analysis was performed, did the review authors assess the potential impact of RoB in individual studies on the results of the meta-analysis or other evidence synthesis? | Y | Y | - | Y |
| 13 | Did the review authors account for RoB in primary studies when interpreting/discussing the results of the review? | Y | Y | - | Y |
| 14 | Did the review authors provide a satisfactory explanation for, and discussion of, any heterogeneity observed in the results of the review? | Y | Y | - | Y |
| 15 | If they performed quantitative synthesis did the review authors carry out an adequate investigation of publication bias (small study bias) and discuss its likely impact on the results of the review? | N | N | - | N |
| 16 | Did the review authors report any potential sources of conflict of interest, including any funding they received for conducting the review? | N | N | - | N |

19.刘香.奥马珠单抗治疗儿童中重度过敏性哮喘有效性及安全性Meta分析[D].南昌大学,2024.DOI:10.27232/d.cnki.gnchu.2024.004079.

| Entry | | A | B | C | D |
| --- | --- | --- | --- | --- | --- |
| 1 | Did the research questions and inclusion criteria for the review include the components of PICO? | Y | Y | - | Y |
| 2 | Did the report of the review contain an explicit statement that the review methods were established prior to the conduct of the review and did the report justify any significantdeviations from the protocol? | Y | PY | PY | PY |
| 3 | Did the review authors explain their selection of the study designs for inclusion in the review? | Y | Y | - | Y |
| 4 | Did the review authors use a comprehensive literature search strategy? | Y | Y | - | Y |
| 5 | Did the review authors perform study selection in duplicate? | Y | PY | Y | Y |
| 6 | Did the review authors perform data extraction in duplicate? | Y | PY | Y | Y |
| 7 | Did the review authors provide a list of excluded studies and justify the exclusions? | Y | Y | PY | PY |
| 8 | Did the review authors describe the included studies in adequate detail? | Y | Y | - | Y |
| 9 | Did the review authors use a satisfactory technique for assessing the risk of bias (RoB) in individual studies that were included in the review? | Y | Y | - | Y |
| 10 | Did the review authors report on the sources of funding for the studies included in the review? | N | N | - | N |
| 11 | If meta-analysis was performed, did the review authors use appropriate methods for statistical combination of results? | Y | Y | - | Y |
| 12 | If meta-analysis was performed, did the review authors assess the potential impact of RoB in individual studies on the results of the meta-analysis or other evidence synthesis? | Y | PY | Y | Y |
| 13 | Did the review authors account for RoB in primary studies when interpreting/discussing the results of the review? | Y | PY | Y | Y |
| 14 | Did the review authors provide a satisfactory explanation for, and discussion of, any heterogeneity observed in the results of the review? | Y | PY | Y | Y |
| 15 | If they performed quantitative synthesis did the review authors carry out an adequate investigation of publication bias (small study bias) and discuss its likely impact on the results of the review? | PY | PY | - | PY |
| 16 | Did the review authors report any potential sources of conflict of interest, including any funding they received for conducting the review? | N | N | - | N |

**PRISMA 2020**

Y: Yes; N: No; PY: Partial Yes.

A:The conclusions of researcher Ph.D. Zhuang Wang.

B:The conclusions of researcher Master Dongze Li.

C:In case of a difference of opinions, it shall be adjudicated by Professor Yongji Wang.

D:Conclusive conclusion.

1.麦琳,孙蓉菲,李为民.奥马佐单抗治疗过敏性支气管哮喘疗效与安全性的系统评价[J].中国循证医学杂志,2013,13(06):709-716.

| Section and topic | Item # | Checklist item | A | B | C | D |
| --- | --- | --- | --- | --- | --- | --- |
| **Title** | | | | | | |
| Title | 1 | Identify the report as a systematic review. | Y | Y | - | Y |
| **Abstract** | | | | | | |
| Abstract | 2 | See the PRISMA 2020 for Abstracts checklist (table 2). | PY | PY | - | PY |
| **Introduction** | | | | | | |
| Rationale | 3 | Describe the rationale for the review in the context of existing knowledge | Y | Y | - | Y |
| Objectives | 4 | Provide an explicit statement of the objective(s) or question(s) the review addresses. | Y | Y | - | Y |
| **Methods** | | | | | | |
| Eligibility criteria | 5 | Specify the inclusion and exclusion criteria for the review and how studies were grouped for the syntheses. | Y | Y | - | Y |
| Information sources | 6 | Specify all databases, registers, websites, organisations, reference lists and other sources searched or consulted to identify studies. Specify the date when each source was last searched or consulted. | PY | Y | Y | Y |
| Search strategy | 7 | Present the full search strategies for all databases, registers and websites, including any filters and limits used | PY | Y | PY | PY |
| Selection process | 8 | Specify the methods used to decide whether a study met the inclusion criteria of the review, including how many reviewers screened each record and each report retrieved, whether they worked independently, and if applicable, details of automation tools  used in the process. | Y | PY | PY | PY |
| Data collection  process | 9 | Specify the methods used to collect data from reports, including how many reviewers collected data from each report, whether they worked independently, any processes for obtaining or confirming data from study investigators, and if applicable, details of automation tools used in the process. | Y | Y | - | Y |
| Data items | 10a | List and define all outcomes for which data were sought. Specify whether all results that were compatible with each outcome domain in each study were sought (e.g. for all measures, time points, analyses), and if not, the methods used to decide which  results to collect. | Y | Y | - | Y |
|  | 10b | List and define all other variables for which data were sought (e.g. participant and intervention characteristics, funding sources). Describe any assumptions made about any missing or unclear information. | Y | Y | - | Y |
| Study risk of bias  assessment | 11 | Specify the methods used to assess risk of bias in the included studies, including details of the tool(s) used, how many reviewers assessed each study and whether they worked independently, and if applicable, details of automation tools used in the process. | Y | Y | - | Y |
| Effect measures | 12 | Specify for each outcome the effect measure(s) (e.g. risk ratio, mean difference) used in the synthesis or presentation of results. | Y | Y | - | Y |
| Synthesis methods | 13a | Describe the processes used to decide which studies were eligible for each synthesis (e.g. tabulating the study intervention characteristics and comparing against the planned groups for each synthesis (item #5)). | Y | Y | - | Y |
|  | 13b | Describe any methods required to prepare the data for presentation or synthesis, such as handling of missing summary statistics, or data conversions. | Y | Y | - | Y |
|  | 13c | Describe any methods used to tabulate or visually display results of individual studies and syntheses. | Y | Y | - | Y |
|  | 13d | Describe any methods used to synthesise results and provide a rationale for the choice(s). If meta-analysis was performed, describe the model(s), method(s) to identify the presence and extent of statistical heterogeneity, and software package(s) used. | Y | Y | - | Y |
|  | 13e | Describe any methods used to explore possible causes of heterogeneity among study results (e.g. subgroup analysis, meta regression). | PY | PY | - | PY |
|  | 13f | Describe any sensitivity analyses conducted to assess robustness of the synthesised results. | PY | PY | - | PY |
| Reporting bias  assessment | 14 | Describe any methods used to assess risk of bias due to missing results in a synthesis (arising from reporting biases). | N | PY | PY | PY |
| Certainty assessment | 15 | Describe any methods used to assess certainty (or confidence) in the body of evidence for an outcome. | N | N | - | N |
| **Results** | | | | | | |
| Study selection | 16a | Describe the results of the search and selection process, from the number of records identified in the search to the number of studies included in the review, ideally using a flow diagram (see fig 1). | Y | Y | - | Y |
|  | 16b | Cite studies that might appear to meet the inclusion criteria, but which were excluded, and explain why they were excluded. | N | Y | PY | PY |
| Study characteristics | 17 | Cite each included study and present its characteristics. | Y | Y | - | Y |
| Risk of bias in studies | 18 | Present assessments of risk of bias for each included study. | Y | Y | - | Y |
| Results of individual  studies | 19 | For all outcomes, present, for each study: (a) summary statistics for each group (where appropriate) and (b) an effect estimate and its precision (e.g. confidence/credible interval), ideally using structured tables or plots. | Y | Y | - | Y |
| Results of syntheses | 20a | For each synthesis, briefly summarise the characteristics and risk of bias among contributing studies. | Y | Y | - | Y |
|  | 20b | Present results of all statistical syntheses conducted. If meta-analysis was done, present for each the summary estimate and its precision (e.g. confidence/credible interval) and measures of statistical heterogeneity. If comparing groups, describe the direction of the effect. | Y | Y | - | Y |
|  | 20c | Present results of all investigations of possible causes of heterogeneity among study results. | PY | PY | - | PY |
|  | 20d | Present results of all sensitivity analyses conducted to assess the robustness of the synthesised results. | PY | PY | - | PY |
| Reporting biases | 21 | Present assessments of risk of bias due to missing results (arising from reporting biases) for each synthesis assessed. | N | PY | PY | PY |
| Certainty of evidence | 22 | Present assessments of certainty (or confidence) in the body of evidence for each outcome assessed. | N | N | - | N |
| **Discussion** | | | | | | |
| Discussion | 23a | Provide a general interpretation of the results in the context of other evidence. | Y | Y | - | Y |
|  | 23b | Discuss any limitations of the evidence included in the review. | Y | Y | - | Y |
|  | 23c | Discuss any limitations of the review processes used. | Y | Y | - | Y |
|  | 23d | Discuss implications of the results for practice, policy, and future research. | Y | Y | - | Y |
| **Other information** | | | | | | |
| Registration and  protocol | 24a | Provide registration information for the review, including register name and registration number, or state that the review was not registered. | N | N | - | N |
|  | 24b | Indicate where the review protocol can be accessed, or state that a protocol was not prepared. | N | N | - | N |
|  | 24c | Describe and explain any amendments to information provided at registration or in the protocol. | N | N | - | N |
| Support | 25 | Describe sources of financial or non-financial support for the review, and the role of the funders or sponsors in the review. | N | PY | PY | PY |
| Competing interests | 26 | Declare any competing interests of review authors. | N | N | - | N |
| Availability of data,  code, and other  materials | 27 | Report which of the following are publicly available and where they can be found: template data collection forms; data extracted from included studies; data used for all analyses; analytic code; any other materials used in the review. | N | N | - | N |

2.刘长智,郑晓滨,刘茂,等.奥马珠单抗治疗难治性哮喘疗效与安全性的Meta分析[J].中华临床医师杂志(电子版),2013,7(14):6519-6525.

| Section and topic | Item # | Checklist item | A | B | C | D |
| --- | --- | --- | --- | --- | --- | --- |
| **Title** | | | | | | |
| Title | 1 | Identify the report as a systematic review. | Y | Y | - | Y |
| **Abstract** | | | | | | |
| Abstract | 2 | See the PRISMA 2020 for Abstracts checklist (table 2). | PY | PY | - | PY |
| **Introduction** | | | | | | |
| Rationale | 3 | Describe the rationale for the review in the context of existing knowledge | Y | Y | - | Y |
| Objectives | 4 | Provide an explicit statement of the objective(s) or question(s) the review addresses. | Y | Y | - | Y |
| **Methods** | | | | | | |
| Eligibility criteria | 5 | Specify the inclusion and exclusion criteria for the review and how studies were grouped for the syntheses. | Y | Y | - | Y |
| Information sources | 6 | Specify all databases, registers, websites, organisations, reference lists and other sources searched or consulted to identify studies. Specify the date when each source was last searched or consulted. | PY | Y | Y | Y |
| Search strategy | 7 | Present the full search strategies for all databases, registers and websites, including any filters and limits used | N | PY | PY | PY |
| Selection process | 8 | Specify the methods used to decide whether a study met the inclusion criteria of the review, including how many reviewers screened each record and each report retrieved, whether they worked independently, and if applicable, details of automation tools  used in the process. | PY | PY | - | PY |
| Data collection  process | 9 | Specify the methods used to collect data from reports, including how many reviewers collected data from each report, whether they worked independently, any processes for obtaining or confirming data from study investigators, and if applicable, details of automation tools used in the process. | PY | PY | - | PY |
| Data items | 10a | List and define all outcomes for which data were sought. Specify whether all results that were compatible with each outcome domain in each study were sought (e.g. for all measures, time points, analyses), and if not, the methods used to decide which  results to collect. | Y | Y | - | Y |
|  | 10b | List and define all other variables for which data were sought (e.g. participant and intervention characteristics, funding sources). Describe any assumptions made about any missing or unclear information. | Y | Y | - | Y |
| Study risk of bias  assessment | 11 | Specify the methods used to assess risk of bias in the included studies, including details of the tool(s) used, how many reviewers assessed each study and whether they worked independently, and if applicable, details of automation tools used in the process. | PY | Y | - | PY |
| Effect measures | 12 | Specify for each outcome the effect measure(s) (e.g. risk ratio, mean difference) used in the synthesis or presentation of results. | Y | Y | - | Y |
| Synthesis methods | 13a | Describe the processes used to decide which studies were eligible for each synthesis (e.g. tabulating the study intervention characteristics and comparing against the planned groups for each synthesis (item #5)). | N | Y | Y | Y |
|  | 13b | Describe any methods required to prepare the data for presentation or synthesis, such as handling of missing summary statistics, or data conversions. | N | Y | PY | PY |
|  | 13c | Describe any methods used to tabulate or visually display results of individual studies and syntheses. | N | Y | Y | Y |
|  | 13d | Describe any methods used to synthesise results and provide a rationale for the choice(s). If meta-analysis was performed, describe the model(s), method(s) to identify the presence and extent of statistical heterogeneity, and software package(s) used. | Y | Y | - | Y |
|  | 13e | Describe any methods used to explore possible causes of heterogeneity among study results (e.g. subgroup analysis, meta regression). | N | Y | PY | PY |
|  | 13f | Describe any sensitivity analyses conducted to assess robustness of the synthesised results. | N | N | - | N |
| Reporting bias  assessment | 14 | Describe any methods used to assess risk of bias due to missing results in a synthesis (arising from reporting biases). | N | Y | PY | PY |
| Certainty assessment | 15 | Describe any methods used to assess certainty (or confidence) in the body of evidence for an outcome. | N | Y | N | N |
| **Results** | | | | | | |
| Study selection | 16a | Describe the results of the search and selection process, from the number of records identified in the search to the number of studies included in the review, ideally using a flow diagram (see fig 1). | PY | Y | PY | PY |
|  | 16b | Cite studies that might appear to meet the inclusion criteria, but which were excluded, and explain why they were excluded. | N | Y | PY | PY |
| Study characteristics | 17 | Cite each included study and present its characteristics. | Y | Y | - | Y |
| Risk of bias in studies | 18 | Present assessments of risk of bias for each included study. | Y | Y | - | Y |
| Results of individual  studies | 19 | For all outcomes, present, for each study: (a) summary statistics for each group (where appropriate) and (b) an effect estimate and its precision (e.g. confidence/credible interval), ideally using structured tables or plots. | Y | Y | - | Y |
| Results of syntheses | 20a | For each synthesis, briefly summarise the characteristics and risk of bias among contributing studies. | Y | Y | - | Y |
|  | 20b | Present results of all statistical syntheses conducted. If meta-analysis was done, present for each the summary estimate and its precision (e.g. confidence/credible interval) and measures of statistical heterogeneity. If comparing groups, describe the direction of the effect. | Y | Y | - | Y |
|  | 20c | Present results of all investigations of possible causes of heterogeneity among study results. | N | Y | PY | PY |
|  | 20d | Present results of all sensitivity analyses conducted to assess the robustness of the synthesised results. | N | PY | PY | PY |
| Reporting biases | 21 | Present assessments of risk of bias due to missing results (arising from reporting biases) for each synthesis assessed. | N | Y | PY | PY |
| Certainty of evidence | 22 | Present assessments of certainty (or confidence) in the body of evidence for each outcome assessed. | N | Y | N | N |
| **Discussion** | | | | | | |
| Discussion | 23a | Provide a general interpretation of the results in the context of other evidence. | Y | Y | - | Y |
|  | 23b | Discuss any limitations of the evidence included in the review. | Y | Y | - | Y |
|  | 23c | Discuss any limitations of the review processes used. | Y | Y | - | Y |
|  | 23d | Discuss implications of the results for practice, policy, and future research. | Y | Y | - | Y |
| **Other information** | | | | | | |
| Registration and  protocol | 24a | Provide registration information for the review, including register name and registration number, or state that the review was not registered. | N | N | - | N |
|  | 24b | Indicate where the review protocol can be accessed, or state that a protocol was not prepared. | N | N | - | N |
|  | 24c | Describe and explain any amendments to information provided at registration or in the protocol. | N | N | - | N |
| Support | 25 | Describe sources of financial or non-financial support for the review, and the role of the funders or sponsors in the review. | N | Y | PY | PY |
| Competing interests | 26 | Declare any competing interests of review authors. | N | Y | N | N |
| Availability of data,  code, and other  materials | 27 | Report which of the following are publicly available and where they can be found: template data collection forms; data extracted from included studies; data used for all analyses; analytic code; any other materials used in the review. | N | N | - | N |

3.Lai T, Wang S, Xu Z, Zhang C, Zhao Y, Hu Y, Cao C, Ying S, Chen Z, Li W, Wu B, Shen H. Long-term efficacy and safety of omalizumab in patients with persistent uncontrolled allergic asthma: a systematic review and meta-analysis. Sci Rep. 2015 Feb 3;5:8191. doi: 10.1038/srep08191. Erratum in: Sci Rep. 2015 Aug 14;5:9548. doi: 10.1038/srep09548. PMID: 25645133; PMCID: PMC4314644.

| Section and topic | Item # | Checklist item | A | B | C | D |
| --- | --- | --- | --- | --- | --- | --- |
| **Title** | | | | | | |
| Title | 1 | Identify the report as a systematic review. | Y | Y | - | Y |
| **Abstract** | | | | | | |
| Abstract | 2 | See the PRISMA 2020 for Abstracts checklist (table 2). | PY | PY | - | PY |
| **Introduction** | | | | | | |
| Rationale | 3 | Describe the rationale for the review in the context of existing knowledge | Y | Y | - | Y |
| Objectives | 4 | Provide an explicit statement of the objective(s) or question(s) the review addresses. | Y | Y | - | Y |
| **Methods** | | | | | | |
| Eligibility criteria | 5 | Specify the inclusion and exclusion criteria for the review and how studies were grouped for the syntheses. | Y | Y | - | Y |
| Information sources | 6 | Specify all databases, registers, websites, organisations, reference lists and other sources searched or consulted to identify studies. Specify the date when each source was last searched or consulted. | PY | Y | PY | PY |
| Search strategy | 7 | Present the full search strategies for all databases, registers and websites, including any filters and limits used | N | Y | PY | PY |
| Selection process | 8 | Specify the methods used to decide whether a study met the inclusion criteria of the review, including how many reviewers screened each record and each report retrieved, whether they worked independently, and if applicable, details of automation tools  used in the process. | Y | Y | - | Y |
| Data collection  process | 9 | Specify the methods used to collect data from reports, including how many reviewers collected data from each report, whether they worked independently, any processes for obtaining or confirming data from study investigators, and if applicable, details of automation tools used in the process. | Y | Y | - | Y |
| Data items | 10a | List and define all outcomes for which data were sought. Specify whether all results that were compatible with each outcome domain in each study were sought (e.g. for all measures, time points, analyses), and if not, the methods used to decide which  results to collect. | Y | Y | - | Y |
|  | 10b | List and define all other variables for which data were sought (e.g. participant and intervention characteristics, funding sources). Describe any assumptions made about any missing or unclear information. | Y | Y | - | Y |
| Study risk of bias  assessment | 11 | Specify the methods used to assess risk of bias in the included studies, including details of the tool(s) used, how many reviewers assessed each study and whether they worked independently, and if applicable, details of automation tools used in the process. | Y | Y | - | Y |
| Effect measures | 12 | Specify for each outcome the effect measure(s) (e.g. risk ratio, mean difference) used in the synthesis or presentation of results. | Y | Y | - | Y |
| Synthesis methods | 13a | Describe the processes used to decide which studies were eligible for each synthesis (e.g. tabulating the study intervention characteristics and comparing against the planned groups for each synthesis (item #5)). | Y | Y | - | Y |
|  | 13b | Describe any methods required to prepare the data for presentation or synthesis, such as handling of missing summary statistics, or data conversions. | Y | Y | - | Y |
|  | 13c | Describe any methods used to tabulate or visually display results of individual studies and syntheses. | Y | Y | - | Y |
|  | 13d | Describe any methods used to synthesise results and provide a rationale for the choice(s). If meta-analysis was performed, describe the model(s), method(s) to identify the presence and extent of statistical heterogeneity, and software package(s) used. | Y | Y | - | Y |
|  | 13e | Describe any methods used to explore possible causes of heterogeneity among study results (e.g. subgroup analysis, meta regression). | Y | Y | - | Y |
|  | 13f | Describe any sensitivity analyses conducted to assess robustness of the synthesised results. | Y | Y | - | Y |
| Reporting bias  assessment | 14 | Describe any methods used to assess risk of bias due to missing results in a synthesis (arising from reporting biases). | N | Y | PY | PY |
| Certainty assessment | 15 | Describe any methods used to assess certainty (or confidence) in the body of evidence for an outcome. | N | N | - | N |
| **Results** | | | | | | |
| Study selection | 16a | Describe the results of the search and selection process, from the number of records identified in the search to the number of studies included in the review, ideally using a flow diagram (see fig 1). | Y | Y | - | Y |
|  | 16b | Cite studies that might appear to meet the inclusion criteria, but which were excluded, and explain why they were excluded. | N | N | - | N |
| Study characteristics | 17 | Cite each included study and present its characteristics. | Y | Y | - | Y |
| Risk of bias in studies | 18 | Present assessments of risk of bias for each included study. | Y | Y | - | Y |
| Results of individual  studies | 19 | For all outcomes, present, for each study: (a) summary statistics for each group (where appropriate) and (b) an effect estimate and its precision (e.g. confidence/credible interval), ideally using structured tables or plots. | Y | Y | - | Y |
| Results of syntheses | 20a | For each synthesis, briefly summarise the characteristics and risk of bias among contributing studies. | Y | Y | - | Y |
|  | 20b | Present results of all statistical syntheses conducted. If meta-analysis was done, present for each the summary estimate and its precision (e.g. confidence/credible interval) and measures of statistical heterogeneity. If comparing groups, describe the direction of the effect. | Y | Y | - | Y |
|  | 20c | Present results of all investigations of possible causes of heterogeneity among study results. | Y | Y | - | Y |
|  | 20d | Present results of all sensitivity analyses conducted to assess the robustness of the synthesised results. | Y | PY | Y | Y |
| Reporting biases | 21 | Present assessments of risk of bias due to missing results (arising from reporting biases) for each synthesis assessed. | N | Y | PY | PY |
| Certainty of evidence | 22 | Present assessments of certainty (or confidence) in the body of evidence for each outcome assessed. | N | N | - | N |
| **Discussion** | | | | | | |
| Discussion | 23a | Provide a general interpretation of the results in the context of other evidence. | Y | Y | - | Y |
|  | 23b | Discuss any limitations of the evidence included in the review. | Y | Y | - | Y |
|  | 23c | Discuss any limitations of the review processes used. | Y | Y | - | Y |
|  | 23d | Discuss implications of the results for practice, policy, and future research. | Y | Y | - | Y |
| **Other information** | | | | | | |
| Registration and  protocol | 24a | Provide registration information for the review, including register name and registration number, or state that the review was not registered. | N | N | - | N |
|  | 24b | Indicate where the review protocol can be accessed, or state that a protocol was not prepared. | N | N | - | N |
|  | 24c | Describe and explain any amendments to information provided at registration or in the protocol. | N | N | - | N |
| Support | 25 | Describe sources of financial or non-financial support for the review, and the role of the funders or sponsors in the review. | Y | Y | - | Y |
| Competing interests | 26 | Declare any competing interests of review authors. | Y | Y | - | Y |
| Availability of data,  code, and other  materials | 27 | Report which of the following are publicly available and where they can be found: template data collection forms; data extracted from included studies; data used for all analyses; analytic code; any other materials used in the review. | N | N | - | N |

4..Rodrigo GJ, Neffen H. Systematic review on the use of omalizumab for the treatment of asthmatic children and adolescents. Pediatr Allergy Immunol. 2015 Sep;26(6):551-6. doi: 10.1111/pai.12405. Epub 2015 Jul 1. PMID: 25963882.

| Section and topic | Item # | Checklist item | A | B | C | D |
| --- | --- | --- | --- | --- | --- | --- |
| **Title** | | | | | | |
| Title | 1 | Identify the report as a systematic review. | Y | Y | - | Y |
| **Abstract** | | | | | | |
| Abstract | 2 | See the PRISMA 2020 for Abstracts checklist (table 2). | PY | PY | - | PY |
| **Introduction** | | | | | | |
| Rationale | 3 | Describe the rationale for the review in the context of existing knowledge | Y | Y | - | Y |
| Objectives | 4 | Provide an explicit statement of the objective(s) or question(s) the review addresses. | Y | Y | - | Y |
| **Methods** | | | | | | |
| Eligibility criteria | 5 | Specify the inclusion and exclusion criteria for the review and how studies were grouped for the syntheses. | Y | Y | - | Y |
| Information sources | 6 | Specify all databases, registers, websites, organisations, reference lists and other sources searched or consulted to identify studies. Specify the date when each source was last searched or consulted. | Y | Y | - | Y |
| Search strategy | 7 | Present the full search strategies for all databases, registers and websites, including any filters and limits used | N | PY | Y | Y |
| Selection process | 8 | Specify the methods used to decide whether a study met the inclusion criteria of the review, including how many reviewers screened each record and each report retrieved, whether they worked independently, and if applicable, details of automation tools  used in the process. | Y | Y | - | Y |
| Data collection  process | 9 | Specify the methods used to collect data from reports, including how many reviewers collected data from each report, whether they worked independently, any processes for obtaining or confirming data from study investigators, and if applicable, details of automation tools used in the process. | Y | Y | - | Y |
| Data items | 10a | List and define all outcomes for which data were sought. Specify whether all results that were compatible with each outcome domain in each study were sought (e.g. for all measures, time points, analyses), and if not, the methods used to decide which  results to collect. | Y | Y | - | Y |
|  | 10b | List and define all other variables for which data were sought (e.g. participant and intervention characteristics, funding sources). Describe any assumptions made about any missing or unclear information. | Y | Y | - | Y |
| Study risk of bias  assessment | 11 | Specify the methods used to assess risk of bias in the included studies, including details of the tool(s) used, how many reviewers assessed each study and whether they worked independently, and if applicable, details of automation tools used in the process. | Y | Y | - | Y |
| Effect measures | 12 | Specify for each outcome the effect measure(s) (e.g. risk ratio, mean difference) used in the synthesis or presentation of results. | Y | Y | - | Y |
| Synthesis methods | 13a | Describe the processes used to decide which studies were eligible for each synthesis (e.g. tabulating the study intervention characteristics and comparing against the planned groups for each synthesis (item #5)). | Y | Y | - | Y |
|  | 13b | Describe any methods required to prepare the data for presentation or synthesis, such as handling of missing summary statistics, or data conversions. | Y | Y | - | Y |
|  | 13c | Describe any methods used to tabulate or visually display results of individual studies and syntheses. | Y | Y | - | Y |
|  | 13d | Describe any methods used to synthesise results and provide a rationale for the choice(s). If meta-analysis was performed, describe the model(s), method(s) to identify the presence and extent of statistical heterogeneity, and software package(s) used. | Y | Y | - | Y |
|  | 13e | Describe any methods used to explore possible causes of heterogeneity among study results (e.g. subgroup analysis, meta regression). | Y | Y | - | Y |
|  | 13f | Describe any sensitivity analyses conducted to assess robustness of the synthesised results. | PY | N | Y | Y |
| Reporting bias  assessment | 14 | Describe any methods used to assess risk of bias due to missing results in a synthesis (arising from reporting biases). | N | PY | PY | PY |
| Certainty assessment | 15 | Describe any methods used to assess certainty (or confidence) in the body of evidence for an outcome. | N | PY | PY | PY |
| **Results** | | | | | | |
| Study selection | 16a | Describe the results of the search and selection process, from the number of records identified in the search to the number of studies included in the review, ideally using a flow diagram (see fig 1). | Y | Y | - | Y |
|  | 16b | Cite studies that might appear to meet the inclusion criteria, but which were excluded, and explain why they were excluded. | N | Y | PY | PY |
| Study characteristics | 17 | Cite each included study and present its characteristics. | Y | Y | - | Y |
| Risk of bias in studies | 18 | Present assessments of risk of bias for each included study. | Y | Y | - | Y |
| Results of individual  studies | 19 | For all outcomes, present, for each study: (a) summary statistics for each group (where appropriate) and (b) an effect estimate and its precision (e.g. confidence/credible interval), ideally using structured tables or plots. | Y | Y | - | Y |
| Results of syntheses | 20a | For each synthesis, briefly summarise the characteristics and risk of bias among contributing studies. | Y | Y | - | Y |
|  | 20b | Present results of all statistical syntheses conducted. If meta-analysis was done, present for each the summary estimate and its precision (e.g. confidence/credible interval) and measures of statistical heterogeneity. If comparing groups, describe the direction of the effect. | Y | Y | - | Y |
|  | 20c | Present results of all investigations of possible causes of heterogeneity among study results. | Y | Y | - | Y |
|  | 20d | Present results of all sensitivity analyses conducted to assess the robustness of the synthesised results. | PY | PY | - | PY |
| Reporting biases | 21 | Present assessments of risk of bias due to missing results (arising from reporting biases) for each synthesis assessed. | N | PY | PY | PY |
| Certainty of evidence | 22 | Present assessments of certainty (or confidence) in the body of evidence for each outcome assessed. | N | PY | PY | PY |
| **Discussion** | | | | | | |
| Discussion | 23a | Provide a general interpretation of the results in the context of other evidence. | Y | Y | - | Y |
|  | 23b | Discuss any limitations of the evidence included in the review. | Y | Y | - | Y |
|  | 23c | Discuss any limitations of the review processes used. | Y | Y | - | Y |
|  | 23d | Discuss implications of the results for practice, policy, and future research. | Y | Y | - | Y |
| **Other information** | | | | | | |
| Registration and  protocol | 24a | Provide registration information for the review, including register name and registration number, or state that the review was not registered. | N | N | - | N |
|  | 24b | Indicate where the review protocol can be accessed, or state that a protocol was not prepared. | N | N | - | N |
|  | 24c | Describe and explain any amendments to information provided at registration or in the protocol. | N | N | - | N |
| Support | 25 | Describe sources of financial or non-financial support for the review, and the role of the funders or sponsors in the review. | Y | Y | - | Y |
| Competing interests | 26 | Declare any competing interests of review authors. | Y | Y | - | Y |
| Availability of data,  code, and other  materials | 27 | Report which of the following are publicly available and where they can be found: template data collection forms; data extracted from included studies; data used for all analyses; analytic code; any other materials used in the review. | N | N | - | N |

5.苗伟伟,汪凤凤,陈子,等.奥马珠单抗治疗难治性哮喘疗效的Meta分析[J].中国呼吸与危重监护杂志,2015,14(05):449-455.

| Section and topic | Item # | Checklist item | A | B | C | D |
| --- | --- | --- | --- | --- | --- | --- |
| **Title** | | | | | | |
| Title | 1 | Identify the report as a systematic review. | Y | Y | - | Y |
| **Abstract** | | | | | | |
| Abstract | 2 | See the PRISMA 2020 for Abstracts checklist (table 2). | PY | PY | - | PY |
| **Introduction** | | | | | | |
| Rationale | 3 | Describe the rationale for the review in the context of existing knowledge | Y | Y | - | Y |
| Objectives | 4 | Provide an explicit statement of the objective(s) or question(s) the review addresses. | Y | Y | - | Y |
| **Methods** | | | | | | |
| Eligibility criteria | 5 | Specify the inclusion and exclusion criteria for the review and how studies were grouped for the syntheses. | Y | Y | - | Y |
| Information sources | 6 | Specify all databases, registers, websites, organisations, reference lists and other sources searched or consulted to identify studies. Specify the date when each source was last searched or consulted. | PY | Y | PY | PY |
| Search strategy | 7 | Present the full search strategies for all databases, registers and websites, including any filters and limits used | N | PY | PY | PY |
| Selection process | 8 | Specify the methods used to decide whether a study met the inclusion criteria of the review, including how many reviewers screened each record and each report retrieved, whether they worked independently, and if applicable, details of automation tools  used in the process. | Y | Y | - | Y |
| Data collection  process | 9 | Specify the methods used to collect data from reports, including how many reviewers collected data from each report, whether they worked independently, any processes for obtaining or confirming data from study investigators, and if applicable, details of automation tools used in the process. | Y | Y | - | Y |
| Data items | 10a | List and define all outcomes for which data were sought. Specify whether all results that were compatible with each outcome domain in each study were sought (e.g. for all measures, time points, analyses), and if not, the methods used to decide which  results to collect. | Y | Y | - | Y |
|  | 10b | List and define all other variables for which data were sought (e.g. participant and intervention characteristics, funding sources). Describe any assumptions made about any missing or unclear information. | Y | Y | - | Y |
| Study risk of bias  assessment | 11 | Specify the methods used to assess risk of bias in the included studies, including details of the tool(s) used, how many reviewers assessed each study and whether they worked independently, and if applicable, details of automation tools used in the process. | Y | Y | - | Y |
| Effect measures | 12 | Specify for each outcome the effect measure(s) (e.g. risk ratio, mean difference) used in the synthesis or presentation of results. | Y | Y | - | Y |
| Synthesis methods | 13a | Describe the processes used to decide which studies were eligible for each synthesis (e.g. tabulating the study intervention characteristics and comparing against the planned groups for each synthesis (item #5)). | Y | Y | - | Y |
|  | 13b | Describe any methods required to prepare the data for presentation or synthesis, such as handling of missing summary statistics, or data conversions. | Y | Y | - | Y |
|  | 13c | Describe any methods used to tabulate or visually display results of individual studies and syntheses. | Y | Y | - | Y |
|  | 13d | Describe any methods used to synthesise results and provide a rationale for the choice(s). If meta-analysis was performed, describe the model(s), method(s) to identify the presence and extent of statistical heterogeneity, and software package(s) used. | Y | Y | - | Y |
|  | 13e | Describe any methods used to explore possible causes of heterogeneity among study results (e.g. subgroup analysis, meta regression). | PY | PY | - | PY |
|  | 13f | Describe any sensitivity analyses conducted to assess robustness of the synthesised results. | PY | PY | - | PY |
| Reporting bias  assessment | 14 | Describe any methods used to assess risk of bias due to missing results in a synthesis (arising from reporting biases). | PY | PY | - | PY |
| Certainty assessment | 15 | Describe any methods used to assess certainty (or confidence) in the body of evidence for an outcome. | N | PY | PY | PY |
| **Results** | | | | | | |
| Study selection | 16a | Describe the results of the search and selection process, from the number of records identified in the search to the number of studies included in the review, ideally using a flow diagram (see fig 1). | Y | Y | - | Y |
|  | 16b | Cite studies that might appear to meet the inclusion criteria, but which were excluded, and explain why they were excluded. | N | Y | PY | PY |
| Study characteristics | 17 | Cite each included study and present its characteristics. | Y | Y | - | Y |
| Risk of bias in studies | 18 | Present assessments of risk of bias for each included study. | Y | Y | - | Y |
| Results of individual  studies | 19 | For all outcomes, present, for each study: (a) summary statistics for each group (where appropriate) and (b) an effect estimate and its precision (e.g. confidence/credible interval), ideally using structured tables or plots. | Y | Y | - | Y |
| Results of syntheses | 20a | For each synthesis, briefly summarise the characteristics and risk of bias among contributing studies. | Y | Y | - | Y |
|  | 20b | Present results of all statistical syntheses conducted. If meta-analysis was done, present for each the summary estimate and its precision (e.g. confidence/credible interval) and measures of statistical heterogeneity. If comparing groups, describe the direction of the effect. | Y | Y | - | Y |
|  | 20c | Present results of all investigations of possible causes of heterogeneity among study results. | PY | PY | - | PY |
|  | 20d | Present results of all sensitivity analyses conducted to assess the robustness of the synthesised results. | PY | PY | - | PY |
| Reporting biases | 21 | Present assessments of risk of bias due to missing results (arising from reporting biases) for each synthesis assessed. | N | Y | PY | PY |
| Certainty of evidence | 22 | Present assessments of certainty (or confidence) in the body of evidence for each outcome assessed. | N | N | - | N |
| **Discussion** | | | | | | |
| Discussion | 23a | Provide a general interpretation of the results in the context of other evidence. | Y | Y | - | Y |
|  | 23b | Discuss any limitations of the evidence included in the review. | Y | Y | - | Y |
|  | 23c | Discuss any limitations of the review processes used. | Y | Y | - | Y |
|  | 23d | Discuss implications of the results for practice, policy, and future research. | Y | Y | - | Y |
| **Other information** | | | | | | |
| Registration and  protocol | 24a | Provide registration information for the review, including register name and registration number, or state that the review was not registered. | N | N | - | N |
|  | 24b | Indicate where the review protocol can be accessed, or state that a protocol was not prepared. | N | N | - | N |
|  | 24c | Describe and explain any amendments to information provided at registration or in the protocol. | N | N | - | N |
| Support | 25 | Describe sources of financial or non-financial support for the review, and the role of the funders or sponsors in the review. | Y | Y | - | Y |
| Competing interests | 26 | Declare any competing interests of review authors. | N | Y | PY | PY |
| Availability of data,  code, and other  materials | 27 | Report which of the following are publicly available and where they can be found: template data collection forms; data extracted from included studies; data used for all analyses; analytic code; any other materials used in the review. | N | N | - | N |

6.季春梅,孟玲. 奥马珠单抗长程治疗与短程治疗过敏性哮喘的有效性及安全性比较的meta分析[C]//2016年江苏省药学大会暨第十六届江苏省药师周 论文集. 2016:105-112.

| Section and topic | Item # | Checklist item | A | B | C | D |
| --- | --- | --- | --- | --- | --- | --- |
| **Title** | | | | | | |
| Title | 1 | Identify the report as a systematic review. | Y | Y | - | Y |
| **Abstract** | | | | | | |
| Abstract | 2 | See the PRISMA 2020 for Abstracts checklist (table 2). | PY | PY | - | PY |
| **Introduction** | | | | | | |
| Rationale | 3 | Describe the rationale for the review in the context of existing knowledge | Y | Y | - | Y |
| Objectives | 4 | Provide an explicit statement of the objective(s) or question(s) the review addresses. | Y | Y | - | Y |
| **Methods** | | | | | | |
| Eligibility criteria | 5 | Specify the inclusion and exclusion criteria for the review and how studies were grouped for the syntheses. | Y | Y | - | Y |
| Information sources | 6 | Specify all databases, registers, websites, organisations, reference lists and other sources searched or consulted to identify studies. Specify the date when each source was last searched or consulted. | PY | Y | Y | Y |
| Search strategy | 7 | Present the full search strategies for all databases, registers and websites, including any filters and limits used | N | Y | PY | PY |
| Selection process | 8 | Specify the methods used to decide whether a study met the inclusion criteria of the review, including how many reviewers screened each record and each report retrieved, whether they worked independently, and if applicable, details of automation tools  used in the process. | Y | Y | - | Y |
| Data collection  process | 9 | Specify the methods used to collect data from reports, including how many reviewers collected data from each report, whether they worked independently, any processes for obtaining or confirming data from study investigators, and if applicable, details of automation tools used in the process. | Y | Y | - | Y |
| Data items | 10a | List and define all outcomes for which data were sought. Specify whether all results that were compatible with each outcome domain in each study were sought (e.g. for all measures, time points, analyses), and if not, the methods used to decide which  results to collect. | Y | Y | - | Y |
|  | 10b | List and define all other variables for which data were sought (e.g. participant and intervention characteristics, funding sources). Describe any assumptions made about any missing or unclear information. | Y | Y | - | Y |
| Study risk of bias  assessment | 11 | Specify the methods used to assess risk of bias in the included studies, including details of the tool(s) used, how many reviewers assessed each study and whether they worked independently, and if applicable, details of automation tools used in the process. | Y | Y | - | Y |
| Effect measures | 12 | Specify for each outcome the effect measure(s) (e.g. risk ratio, mean difference) used in the synthesis or presentation of results. | Y | Y | - | Y |
| Synthesis methods | 13a | Describe the processes used to decide which studies were eligible for each synthesis (e.g. tabulating the study intervention characteristics and comparing against the planned groups for each synthesis (item #5)). | Y | Y | - | Y |
|  | 13b | Describe any methods required to prepare the data for presentation or synthesis, such as handling of missing summary statistics, or data conversions. | Y | Y | - | Y |
|  | 13c | Describe any methods used to tabulate or visually display results of individual studies and syntheses. | Y | Y | - | Y |
|  | 13d | Describe any methods used to synthesise results and provide a rationale for the choice(s). If meta-analysis was performed, describe the model(s), method(s) to identify the presence and extent of statistical heterogeneity, and software package(s) used. | Y | Y | - | Y |
|  | 13e | Describe any methods used to explore possible causes of heterogeneity among study results (e.g. subgroup analysis, meta regression). | Y | Y | - | Y |
|  | 13f | Describe any sensitivity analyses conducted to assess robustness of the synthesised results. | PY | N | PY | PY |
| Reporting bias  assessment | 14 | Describe any methods used to assess risk of bias due to missing results in a synthesis (arising from reporting biases). | Y | Y | - | Y |
| Certainty assessment | 15 | Describe any methods used to assess certainty (or confidence) in the body of evidence for an outcome. | N | Y | PY | PY |
| **Results** | | | | | | |
| Study selection | 16a | Describe the results of the search and selection process, from the number of records identified in the search to the number of studies included in the review, ideally using a flow diagram (see fig 1). | Y | Y | - | Y |
|  | 16b | Cite studies that might appear to meet the inclusion criteria, but which were excluded, and explain why they were excluded. | PY | Y | PY | PY |
| Study characteristics | 17 | Cite each included study and present its characteristics. | Y | Y | - | Y |
| Risk of bias in studies | 18 | Present assessments of risk of bias for each included study. | Y | Y | - | Y |
| Results of individual  studies | 19 | For all outcomes, present, for each study: (a) summary statistics for each group (where appropriate) and (b) an effect estimate and its precision (e.g. confidence/credible interval), ideally using structured tables or plots. | Y | Y | - | Y |
| Results of syntheses | 20a | For each synthesis, briefly summarise the characteristics and risk of bias among contributing studies. | Y | Y | - | Y |
|  | 20b | Present results of all statistical syntheses conducted. If meta-analysis was done, present for each the summary estimate and its precision (e.g. confidence/credible interval) and measures of statistical heterogeneity. If comparing groups, describe the direction of the effect. | Y | Y | - | Y |
|  | 20c | Present results of all investigations of possible causes of heterogeneity among study results. | Y | Y | - | Y |
|  | 20d | Present results of all sensitivity analyses conducted to assess the robustness of the synthesised results. | PY | PY | - | PY |
| Reporting biases | 21 | Present assessments of risk of bias due to missing results (arising from reporting biases) for each synthesis assessed. | Y | Y | - | Y |
| Certainty of evidence | 22 | Present assessments of certainty (or confidence) in the body of evidence for each outcome assessed. | N | Y | PY | PY |
| **Discussion** | | | | | | |
| Discussion | 23a | Provide a general interpretation of the results in the context of other evidence. | Y | Y | - | Y |
|  | 23b | Discuss any limitations of the evidence included in the review. | Y | Y | - | Y |
|  | 23c | Discuss any limitations of the review processes used. | Y | Y | - | Y |
|  | 23d | Discuss implications of the results for practice, policy, and future research. | Y | Y | - | Y |
| **Other information** | | | | | | |
| Registration and  protocol | 24a | Provide registration information for the review, including register name and registration number, or state that the review was not registered. | N | N | - | N |
|  | 24b | Indicate where the review protocol can be accessed, or state that a protocol was not prepared. | N | N | - | N |
|  | 24c | Describe and explain any amendments to information provided at registration or in the protocol. | N | N | - | N |
| Support | 25 | Describe sources of financial or non-financial support for the review, and the role of the funders or sponsors in the review. | Y | Y | - | Y |
| Competing interests | 26 | Declare any competing interests of review authors. | Y | Y | - | Y |
| Availability of data,  code, and other  materials | 27 | Report which of the following are publicly available and where they can be found: template data collection forms; data extracted from included studies; data used for all analyses; analytic code; any other materials used in the review. | N | N | - | N |

7.Corren J, Kavati A, Ortiz B, Vegesna A, Colby JA, Ruiz K, Panettieri RA Jr. Patient-reported outcomes in moderate-to-severe allergic asthmatics treated with omalizumab: a systematic literature review of randomized controlled trials. Curr Med Res Opin. 2018 Jan;34(1):65-80. doi: 10.1080/03007995.2017.1395734. Epub 2017 Nov 10. PMID: 29057669.

| Section and topic | Item # | Checklist item | A | B | C | D |
| --- | --- | --- | --- | --- | --- | --- |
| **Title** | | | | | | |
| Title | 1 | Identify the report as a systematic review. | Y | Y | - | Y |
| **Abstract** | | | | | | |
| Abstract | 2 | See the PRISMA 2020 for Abstracts checklist (table 2). | PY | PY | - | PY |
| **Introduction** | | | | | | |
| Rationale | 3 | Describe the rationale for the review in the context of existing knowledge | Y | Y | - | Y |
| Objectives | 4 | Provide an explicit statement of the objective(s) or question(s) the review addresses. | Y | Y | - | Y |
| **Methods** | | | | | | |
| Eligibility criteria | 5 | Specify the inclusion and exclusion criteria for the review and how studies were grouped for the syntheses. | Y | Y | - | Y |
| Information sources | 6 | Specify all databases, registers, websites, organisations, reference lists and other sources searched or consulted to identify studies. Specify the date when each source was last searched or consulted. | Y | Y | - | Y |
| Search strategy | 7 | Present the full search strategies for all databases, registers and websites, including any filters and limits used | N | Y | Y | Y |
| Selection process | 8 | Specify the methods used to decide whether a study met the inclusion criteria of the review, including how many reviewers screened each record and each report retrieved, whether they worked independently, and if applicable, details of automation tools  used in the process. | Y | Y | - | Y |
| Data collection  process | 9 | Specify the methods used to collect data from reports, including how many reviewers collected data from each report, whether they worked independently, any processes for obtaining or confirming data from study investigators, and if applicable, details of automation tools used in the process. | Y | Y | - | Y |
| Data items | 10a | List and define all outcomes for which data were sought. Specify whether all results that were compatible with each outcome domain in each study were sought (e.g. for all measures, time points, analyses), and if not, the methods used to decide which  results to collect. | Y | Y | - | Y |
|  | 10b | List and define all other variables for which data were sought (e.g. participant and intervention characteristics, funding sources). Describe any assumptions made about any missing or unclear information. | Y | Y | - | Y |
| Study risk of bias  assessment | 11 | Specify the methods used to assess risk of bias in the included studies, including details of the tool(s) used, how many reviewers assessed each study and whether they worked independently, and if applicable, details of automation tools used in the process. | Y | Y | - | Y |
| Effect measures | 12 | Specify for each outcome the effect measure(s) (e.g. risk ratio, mean difference) used in the synthesis or presentation of results. | Y | Y | - | Y |
| Synthesis methods | 13a | Describe the processes used to decide which studies were eligible for each synthesis (e.g. tabulating the study intervention characteristics and comparing against the planned groups for each synthesis (item #5)). | PY | Y | PY | PY |
|  | 13b | Describe any methods required to prepare the data for presentation or synthesis, such as handling of missing summary statistics, or data conversions. | PY | Y | PY | PY |
|  | 13c | Describe any methods used to tabulate or visually display results of individual studies and syntheses. | PY | Y | PY | PY |
|  | 13d | Describe any methods used to synthesise results and provide a rationale for the choice(s). If meta-analysis was performed, describe the model(s), method(s) to identify the presence and extent of statistical heterogeneity, and software package(s) used. | PY | Y | PY | PY |
|  | 13e | Describe any methods used to explore possible causes of heterogeneity among study results (e.g. subgroup analysis, meta regression). | PY | Y | PY | PY |
|  | 13f | Describe any sensitivity analyses conducted to assess robustness of the synthesised results. | PY | Y | PY | PY |
| Reporting bias  assessment | 14 | Describe any methods used to assess risk of bias due to missing results in a synthesis (arising from reporting biases). | N | Y | PY | PY |
| Certainty assessment | 15 | Describe any methods used to assess certainty (or confidence) in the body of evidence for an outcome. | N | Y | PY | PY |
| **Results** | | | | | | |
| Study selection | 16a | Describe the results of the search and selection process, from the number of records identified in the search to the number of studies included in the review, ideally using a flow diagram (see fig 1). | Y | Y | - | Y |
|  | 16b | Cite studies that might appear to meet the inclusion criteria, but which were excluded, and explain why they were excluded. | Y | Y | - | Y |
| Study characteristics | 17 | Cite each included study and present its characteristics. | Y | Y | - | Y |
| Risk of bias in studies | 18 | Present assessments of risk of bias for each included study. | Y | Y | - | Y |
| Results of individual  studies | 19 | For all outcomes, present, for each study: (a) summary statistics for each group (where appropriate) and (b) an effect estimate and its precision (e.g. confidence/credible interval), ideally using structured tables or plots. | Y | Y | - | Y |
| Results of syntheses | 20a | For each synthesis, briefly summarise the characteristics and risk of bias among contributing studies. | Y | Y | - | Y |
|  | 20b | Present results of all statistical syntheses conducted. If meta-analysis was done, present for each the summary estimate and its precision (e.g. confidence/credible interval) and measures of statistical heterogeneity. If comparing groups, describe the direction of the effect. | Y | Y | - | Y |
|  | 20c | Present results of all investigations of possible causes of heterogeneity among study results. | Y | Y | - | Y |
|  | 20d | Present results of all sensitivity analyses conducted to assess the robustness of the synthesised results. | PY | Y | PY | PY |
| Reporting biases | 21 | Present assessments of risk of bias due to missing results (arising from reporting biases) for each synthesis assessed. | N | Y | PY | PY |
| Certainty of evidence | 22 | Present assessments of certainty (or confidence) in the body of evidence for each outcome assessed. | N | Y | PY | PY |
| **Discussion** | | | | | | |
| Discussion | 23a | Provide a general interpretation of the results in the context of other evidence. | Y | Y | - | Y |
|  | 23b | Discuss any limitations of the evidence included in the review. | Y | Y | - | Y |
|  | 23c | Discuss any limitations of the review processes used. | Y | Y | - | Y |
|  | 23d | Discuss implications of the results for practice, policy, and future research. | Y | Y | - | Y |
| **Other information** | | | | | | |
| Registration and  protocol | 24a | Provide registration information for the review, including register name and registration number, or state that the review was not registered. | N | Y | N | N |
|  | 24b | Indicate where the review protocol can be accessed, or state that a protocol was not prepared. | N | Y | N | N |
|  | 24c | Describe and explain any amendments to information provided at registration or in the protocol. | N | Y | N | N |
| Support | 25 | Describe sources of financial or non-financial support for the review, and the role of the funders or sponsors in the review. | Y | Y | - | Y |
| Competing interests | 26 | Declare any competing interests of review authors. | Y | Y | - | Y |
| Availability of data,  code, and other  materials | 27 | Report which of the following are publicly available and where they can be found: template data collection forms; data extracted from included studies; data used for all analyses; analytic code; any other materials used in the review. | N | Y | N | N |

8.牟姗,张薇,江德鹏.奥马珠单抗治疗难治性变应性哮喘效果的Meta分析[J].中国医药导报,2019,16(10):75-79+99.

| Section and topic | Item # | Checklist item | A | B | C | D |
| --- | --- | --- | --- | --- | --- | --- |
| **Title** | | | | | | |
| Title | 1 | Identify the report as a systematic review. | Y | Y | - | Y |
| **Abstract** | | | | | | |
| Abstract | 2 | See the PRISMA 2020 for Abstracts checklist (table 2). | PY | PY | - | PY |
| **Introduction** | | | | | | |
| Rationale | 3 | Describe the rationale for the review in the context of existing knowledge | Y | Y | - | Y |
| Objectives | 4 | Provide an explicit statement of the objective(s) or question(s) the review addresses. | Y | Y | - | Y |
| **Methods** | | | | | | |
| Eligibility criteria | 5 | Specify the inclusion and exclusion criteria for the review and how studies were grouped for the syntheses. | Y | Y | - | Y |
| Information sources | 6 | Specify all databases, registers, websites, organisations, reference lists and other sources searched or consulted to identify studies. Specify the date when each source was last searched or consulted. | Y | Y | - | Y |
| Search strategy | 7 | Present the full search strategies for all databases, registers and websites, including any filters and limits used | N | PY | PY | PY |
| Selection process | 8 | Specify the methods used to decide whether a study met the inclusion criteria of the review, including how many reviewers screened each record and each report retrieved, whether they worked independently, and if applicable, details of automation tools  used in the process. | Y | Y | - | Y |
| Data collection  process | 9 | Specify the methods used to collect data from reports, including how many reviewers collected data from each report, whether they worked independently, any processes for obtaining or confirming data from study investigators, and if applicable, details of automation tools used in the process. | Y | Y | - | Y |
| Data items | 10a | List and define all outcomes for which data were sought. Specify whether all results that were compatible with each outcome domain in each study were sought (e.g. for all measures, time points, analyses), and if not, the methods used to decide which  results to collect. | Y | Y | - | Y |
|  | 10b | List and define all other variables for which data were sought (e.g. participant and intervention characteristics, funding sources). Describe any assumptions made about any missing or unclear information. | Y | Y | - | Y |
| Study risk of bias  assessment | 11 | Specify the methods used to assess risk of bias in the included studies, including details of the tool(s) used, how many reviewers assessed each study and whether they worked independently, and if applicable, details of automation tools used in the process. | Y | Y | - | Y |
| Effect measures | 12 | Specify for each outcome the effect measure(s) (e.g. risk ratio, mean difference) used in the synthesis or presentation of results. | Y | Y | - | Y |
| Synthesis methods | 13a | Describe the processes used to decide which studies were eligible for each synthesis (e.g. tabulating the study intervention characteristics and comparing against the planned groups for each synthesis (item #5)). | Y | Y | - | Y |
|  | 13b | Describe any methods required to prepare the data for presentation or synthesis, such as handling of missing summary statistics, or data conversions. | Y | Y | - | Y |
|  | 13c | Describe any methods used to tabulate or visually display results of individual studies and syntheses. | Y | Y | - | Y |
|  | 13d | Describe any methods used to synthesise results and provide a rationale for the choice(s). If meta-analysis was performed, describe the model(s), method(s) to identify the presence and extent of statistical heterogeneity, and software package(s) used. | Y | Y | - | Y |
|  | 13e | Describe any methods used to explore possible causes of heterogeneity among study results (e.g. subgroup analysis, meta regression). | Y | PY | Y | Y |
|  | 13f | Describe any sensitivity analyses conducted to assess robustness of the synthesised results. | Y | N | Y | Y |
| Reporting bias  assessment | 14 | Describe any methods used to assess risk of bias due to missing results in a synthesis (arising from reporting biases). | Y | Y | - | Y |
| Certainty assessment | 15 | Describe any methods used to assess certainty (or confidence) in the body of evidence for an outcome. | N | PY | PY | PY |
| **Results** | | | | | | |
| Study selection | 16a | Describe the results of the search and selection process, from the number of records identified in the search to the number of studies included in the review, ideally using a flow diagram (see fig 1). | Y | Y | - | Y |
|  | 16b | Cite studies that might appear to meet the inclusion criteria, but which were excluded, and explain why they were excluded. | N | Y | PY | PY |
| Study characteristics | 17 | Cite each included study and present its characteristics. | Y | Y | - | Y |
| Risk of bias in studies | 18 | Present assessments of risk of bias for each included study. | Y | Y | - | Y |
| Results of individual  studies | 19 | For all outcomes, present, for each study: (a) summary statistics for each group (where appropriate) and (b) an effect estimate and its precision (e.g. confidence/credible interval), ideally using structured tables or plots. | Y | Y | - | Y |
| Results of syntheses | 20a | For each synthesis, briefly summarise the characteristics and risk of bias among contributing studies. | Y | Y | - | Y |
|  | 20b | Present results of all statistical syntheses conducted. If meta-analysis was done, present for each the summary estimate and its precision (e.g. confidence/credible interval) and measures of statistical heterogeneity. If comparing groups, describe the direction of the effect. | Y | Y | - | Y |
|  | 20c | Present results of all investigations of possible causes of heterogeneity among study results. | Y | Y | - | Y |
|  | 20d | Present results of all sensitivity analyses conducted to assess the robustness of the synthesised results. | PY | PY | - | PY |
| Reporting biases | 21 | Present assessments of risk of bias due to missing results (arising from reporting biases) for each synthesis assessed. | Y | Y | - | Y |
| Certainty of evidence | 22 | Present assessments of certainty (or confidence) in the body of evidence for each outcome assessed. | N | PY | PY | PY |
| **Discussion** | | | | | | |
| Discussion | 23a | Provide a general interpretation of the results in the context of other evidence. | Y | Y | - | Y |
|  | 23b | Discuss any limitations of the evidence included in the review. | Y | Y | - | Y |
|  | 23c | Discuss any limitations of the review processes used. | Y | Y | - | Y |
|  | 23d | Discuss implications of the results for practice, policy, and future research. | Y | Y | - | Y |
| **Other information** | | | | | | |
| Registration and  protocol | 24a | Provide registration information for the review, including register name and registration number, or state that the review was not registered. | N | N | - | N |
|  | 24b | Indicate where the review protocol can be accessed, or state that a protocol was not prepared. | N | N | - | N |
|  | 24c | Describe and explain any amendments to information provided at registration or in the protocol. | N | N | - | N |
| Support | 25 | Describe sources of financial or non-financial support for the review, and the role of the funders or sponsors in the review. | Y | PY | Y | Y |
| Competing interests | 26 | Declare any competing interests of review authors. | N | N | - | N |
| Availability of data,  code, and other  materials | 27 | Report which of the following are publicly available and where they can be found: template data collection forms; data extracted from included studies; data used for all analyses; analytic code; any other materials used in the review. | N | N | - | N |

9.Henriksen DP, Bodtger U, Sidenius K, Maltbaek N, Pedersen L, Madsen H, Andersson EA, Norgaard O, Madsen LK, Chawes BL. Efficacy of omalizumab in children, adolescents, and adults with severe allergic asthma: a systematic review, meta-analysis, and call for new trials using current guidelines for assessment of severe asthma. Allergy Asthma Clin Immunol. 2020 Jun 18;16:49. doi: 10.1186/s13223-020-00442-0. PMID: 32565844; PMCID: PMC7302157.

| Section and topic | Item # | Checklist item | A | B | C | D |
| --- | --- | --- | --- | --- | --- | --- |
| **Title** | | | | | | |
| Title | 1 | Identify the report as a systematic review. | Y | Y | - | Y |
| **Abstract** | | | | | | |
| Abstract | 2 | See the PRISMA 2020 for Abstracts checklist (table 2). | PY | PY | - | PY |
| **Introduction** | | | | | | |
| Rationale | 3 | Describe the rationale for the review in the context of existing knowledge | Y | Y | - | Y |
| Objectives | 4 | Provide an explicit statement of the objective(s) or question(s) the review addresses. | Y | Y | - | Y |
| **Methods** | | | | | | |
| Eligibility criteria | 5 | Specify the inclusion and exclusion criteria for the review and how studies were grouped for the syntheses. | Y | Y | - | Y |
| Information sources | 6 | Specify all databases, registers, websites, organisations, reference lists and other sources searched or consulted to identify studies. Specify the date when each source was last searched or consulted. | Y | Y | - | Y |
| Search strategy | 7 | Present the full search strategies for all databases, registers and websites, including any filters and limits used | PY | Y | Y | Y |
| Selection process | 8 | Specify the methods used to decide whether a study met the inclusion criteria of the review, including how many reviewers screened each record and each report retrieved, whether they worked independently, and if applicable, details of automation tools  used in the process. | Y | Y | - | Y |
| Data collection  process | 9 | Specify the methods used to collect data from reports, including how many reviewers collected data from each report, whether they worked independently, any processes for obtaining or confirming data from study investigators, and if applicable, details of automation tools used in the process. | Y | Y | - | Y |
| Data items | 10a | List and define all outcomes for which data were sought. Specify whether all results that were compatible with each outcome domain in each study were sought (e.g. for all measures, time points, analyses), and if not, the methods used to decide which  results to collect. | Y | Y | - | Y |
|  | 10b | List and define all other variables for which data were sought (e.g. participant and intervention characteristics, funding sources). Describe any assumptions made about any missing or unclear information. | Y | Y | - | Y |
| Study risk of bias  assessment | 11 | Specify the methods used to assess risk of bias in the included studies, including details of the tool(s) used, how many reviewers assessed each study and whether they worked independently, and if applicable, details of automation tools used in the process. | Y | Y | - | Y |
| Effect measures | 12 | Specify for each outcome the effect measure(s) (e.g. risk ratio, mean difference) used in the synthesis or presentation of results. | Y | Y | - | Y |
| Synthesis methods | 13a | Describe the processes used to decide which studies were eligible for each synthesis (e.g. tabulating the study intervention characteristics and comparing against the planned groups for each synthesis (item #5)). | Y | Y | - | Y |
|  | 13b | Describe any methods required to prepare the data for presentation or synthesis, such as handling of missing summary statistics, or data conversions. | Y | Y | - | Y |
|  | 13c | Describe any methods used to tabulate or visually display results of individual studies and syntheses. | Y | Y | - | Y |
|  | 13d | Describe any methods used to synthesise results and provide a rationale for the choice(s). If meta-analysis was performed, describe the model(s), method(s) to identify the presence and extent of statistical heterogeneity, and software package(s) used. | Y | Y | - | Y |
|  | 13e | Describe any methods used to explore possible causes of heterogeneity among study results (e.g. subgroup analysis, meta regression). | Y | Y | - | Y |
|  | 13f | Describe any sensitivity analyses conducted to assess robustness of the synthesised results. | Y | N | Y | Y |
| Reporting bias  assessment | 14 | Describe any methods used to assess risk of bias due to missing results in a synthesis (arising from reporting biases). | Y | Y | - | Y |
| Certainty assessment | 15 | Describe any methods used to assess certainty (or confidence) in the body of evidence for an outcome. | Y | Y | - | Y |
| **Results** | | | | | | |
| Study selection | 16a | Describe the results of the search and selection process, from the number of records identified in the search to the number of studies included in the review, ideally using a flow diagram (see fig 1). | Y | Y | - | Y |
|  | 16b | Cite studies that might appear to meet the inclusion criteria, but which were excluded, and explain why they were excluded. | Y | Y | - | Y |
| Study characteristics | 17 | Cite each included study and present its characteristics. | Y | Y | - | Y |
| Risk of bias in studies | 18 | Present assessments of risk of bias for each included study. | Y | Y | - | Y |
| Results of individual  studies | 19 | For all outcomes, present, for each study: (a) summary statistics for each group (where appropriate) and (b) an effect estimate and its precision (e.g. confidence/credible interval), ideally using structured tables or plots. | Y | Y | - | Y |
| Results of syntheses | 20a | For each synthesis, briefly summarise the characteristics and risk of bias among contributing studies. | Y | Y | - | Y |
|  | 20b | Present results of all statistical syntheses conducted. If meta-analysis was done, present for each the summary estimate and its precision (e.g. confidence/credible interval) and measures of statistical heterogeneity. If comparing groups, describe the direction of the effect. | Y | Y | - | Y |
|  | 20c | Present results of all investigations of possible causes of heterogeneity among study results. | Y | Y | - | Y |
|  | 20d | Present results of all sensitivity analyses conducted to assess the robustness of the synthesised results. | Y | PY | Y | Y |
| Reporting biases | 21 | Present assessments of risk of bias due to missing results (arising from reporting biases) for each synthesis assessed. | Y | Y | - | Y |
| Certainty of evidence | 22 | Present assessments of certainty (or confidence) in the body of evidence for each outcome assessed. | Y | Y | - | Y |
| **Discussion** | | | | | | |
| Discussion | 23a | Provide a general interpretation of the results in the context of other evidence. | Y | Y | - | Y |
|  | 23b | Discuss any limitations of the evidence included in the review. | Y | Y | - | Y |
|  | 23c | Discuss any limitations of the review processes used. | Y | Y | - | Y |
|  | 23d | Discuss implications of the results for practice, policy, and future research. | Y | Y | - | Y |
| **Other information** | | | | | | |
| Registration and  protocol | 24a | Provide registration information for the review, including register name and registration number, or state that the review was not registered. | PY | Y | Y | Y |
|  | 24b | Indicate where the review protocol can be accessed, or state that a protocol was not prepared. | PY | Y | Y | Y |
|  | 24c | Describe and explain any amendments to information provided at registration or in the protocol. | Y | N | Y | Y |
| Support | 25 | Describe sources of financial or non-financial support for the review, and the role of the funders or sponsors in the review. | Y | Y | - | Y |
| Competing interests | 26 | Declare any competing interests of review authors. | Y | Y | - | Y |
| Availability of data,  code, and other  materials | 27 | Report which of the following are publicly available and where they can be found: template data collection forms; data extracted from included studies; data used for all analyses; analytic code; any other materials used in the review. | PY | Y | PY | PY |

10.Fu Z, Xu Y, Cai C. Efficacy and safety of omalizumab in children with moderate-to-severe asthma: a meta-analysis. J Asthma. 2021 Oct;58(10):1350-1358. doi: 10.1080/02770903.2020.1789875. Epub 2020 Jul 16. PMID: 32602383.

| Section and topic | Item # | Checklist item | A | B | C | D |
| --- | --- | --- | --- | --- | --- | --- |
| **Title** | | | | | | |
| Title | 1 | Identify the report as a systematic review. | Y | Y | - | Y |
| **Abstract** | | | | | | |
| Abstract | 2 | See the PRISMA 2020 for Abstracts checklist (table 2). | PY | PY | - | PY |
| **Introduction** | | | | | | |
| Rationale | 3 | Describe the rationale for the review in the context of existing knowledge | Y | Y | - | Y |
| Objectives | 4 | Provide an explicit statement of the objective(s) or question(s) the review addresses. | Y | Y | - | Y |
| **Methods** | | | | | | |
| Eligibility criteria | 5 | Specify the inclusion and exclusion criteria for the review and how studies were grouped for the syntheses. | Y | Y | - | Y |
| Information sources | 6 | Specify all databases, registers, websites, organisations, reference lists and other sources searched or consulted to identify studies. Specify the date when each source was last searched or consulted. | Y | Y | - | Y |
| Search strategy | 7 | Present the full search strategies for all databases, registers and websites, including any filters and limits used | Y | Y | - | Y |
| Selection process | 8 | Specify the methods used to decide whether a study met the inclusion criteria of the review, including how many reviewers screened each record and each report retrieved, whether they worked independently, and if applicable, details of automation tools  used in the process. | Y | Y | - | Y |
| Data collection  process | 9 | Specify the methods used to collect data from reports, including how many reviewers collected data from each report, whether they worked independently, any processes for obtaining or confirming data from study investigators, and if applicable, details of automation tools used in the process. | Y | Y | - | Y |
| Data items | 10a | List and define all outcomes for which data were sought. Specify whether all results that were compatible with each outcome domain in each study were sought (e.g. for all measures, time points, analyses), and if not, the methods used to decide which  results to collect. | Y | Y | - | Y |
|  | 10b | List and define all other variables for which data were sought (e.g. participant and intervention characteristics, funding sources). Describe any assumptions made about any missing or unclear information. | Y | Y | - | Y |
| Study risk of bias  assessment | 11 | Specify the methods used to assess risk of bias in the included studies, including details of the tool(s) used, how many reviewers assessed each study and whether they worked independently, and if applicable, details of automation tools used in the process. | Y | Y | - | Y |
| Effect measures | 12 | Specify for each outcome the effect measure(s) (e.g. risk ratio, mean difference) used in the synthesis or presentation of results. | Y | Y | - | Y |
| Synthesis methods | 13a | Describe the processes used to decide which studies were eligible for each synthesis (e.g. tabulating the study intervention characteristics and comparing against the planned groups for each synthesis (item #5)). | Y | Y | - | Y |
|  | 13b | Describe any methods required to prepare the data for presentation or synthesis, such as handling of missing summary statistics, or data conversions. | Y | Y | - | Y |
|  | 13c | Describe any methods used to tabulate or visually display results of individual studies and syntheses. | Y | Y | - | Y |
|  | 13d | Describe any methods used to synthesise results and provide a rationale for the choice(s). If meta-analysis was performed, describe the model(s), method(s) to identify the presence and extent of statistical heterogeneity, and software package(s) used. | Y | Y | - | Y |
|  | 13e | Describe any methods used to explore possible causes of heterogeneity among study results (e.g. subgroup analysis, meta regression). | Y | Y | - | Y |
|  | 13f | Describe any sensitivity analyses conducted to assess robustness of the synthesised results. | Y | Y | - | Y |
| Reporting bias  assessment | 14 | Describe any methods used to assess risk of bias due to missing results in a synthesis (arising from reporting biases). | Y | Y | - | Y |
| Certainty assessment | 15 | Describe any methods used to assess certainty (or confidence) in the body of evidence for an outcome. | N | N | - | N |
| **Results** | | | | | | |
| Study selection | 16a | Describe the results of the search and selection process, from the number of records identified in the search to the number of studies included in the review, ideally using a flow diagram (see fig 1). | Y | Y | - | Y |
|  | 16b | Cite studies that might appear to meet the inclusion criteria, but which were excluded, and explain why they were excluded. | PY | Y | PY | PY |
| Study characteristics | 17 | Cite each included study and present its characteristics. | Y | Y | - | Y |
| Risk of bias in studies | 18 | Present assessments of risk of bias for each included study. | Y | Y | - | Y |
| Results of individual  studies | 19 | For all outcomes, present, for each study: (a) summary statistics for each group (where appropriate) and (b) an effect estimate and its precision (e.g. confidence/credible interval), ideally using structured tables or plots. | Y | Y | - | Y |
| Results of syntheses | 20a | For each synthesis, briefly summarise the characteristics and risk of bias among contributing studies. | Y | Y | - | Y |
|  | 20b | Present results of all statistical syntheses conducted. If meta-analysis was done, present for each the summary estimate and its precision (e.g. confidence/credible interval) and measures of statistical heterogeneity. If comparing groups, describe the direction of the effect. | Y | Y | - | Y |
|  | 20c | Present results of all investigations of possible causes of heterogeneity among study results. | Y | Y | - | Y |
|  | 20d | Present results of all sensitivity analyses conducted to assess the robustness of the synthesised results. | Y | Y | - | Y |
| Reporting biases | 21 | Present assessments of risk of bias due to missing results (arising from reporting biases) for each synthesis assessed. | Y | Y | - | Y |
| Certainty of evidence | 22 | Present assessments of certainty (or confidence) in the body of evidence for each outcome assessed. | N | N | - | N |
| **Discussion** | | | | | | |
| Discussion | 23a | Provide a general interpretation of the results in the context of other evidence. | Y | Y | - | Y |
|  | 23b | Discuss any limitations of the evidence included in the review. | Y | Y | - | Y |
|  | 23c | Discuss any limitations of the review processes used. | Y | Y | - | Y |
|  | 23d | Discuss implications of the results for practice, policy, and future research. | Y | Y | - | Y |
| **Other information** | | | | | | |
| Registration and  protocol | 24a | Provide registration information for the review, including register name and registration number, or state that the review was not registered. | N | N | - | N |
|  | 24b | Indicate where the review protocol can be accessed, or state that a protocol was not prepared. | N | N | - | N |
|  | 24c | Describe and explain any amendments to information provided at registration or in the protocol. | N | N | - | N |
| Support | 25 | Describe sources of financial or non-financial support for the review, and the role of the funders or sponsors in the review. | Y | Y | - | Y |
| Competing interests | 26 | Declare any competing interests of review authors. | Y | Y | - | Y |
| Availability of data,  code, and other  materials | 27 | Report which of the following are publicly available and where they can be found: template data collection forms; data extracted from included studies; data used for all analyses; analytic code; any other materials used in the review. | N | N | - | N |

11.姜红玉,李娟,胡文凤,等.奥马珠单抗治疗儿童中重度过敏性哮喘的有效性和安全性系统评价[J].中国医院用药评价与分析,2021,21(09):1091-1096.DOI:10.14009/j.issn.1672-2124.2021.09.017.

| Section and topic | Item # | Checklist item | A | B | C | D |
| --- | --- | --- | --- | --- | --- | --- |
| **Title** | | | | | | |
| Title | 1 | Identify the report as a systematic review. | Y | Y | - | Y |
| **Abstract** | | | | | | |
| Abstract | 2 | See the PRISMA 2020 for Abstracts checklist (table 2). | PY | PY | - | PY |
| **Introduction** | | | | | | |
| Rationale | 3 | Describe the rationale for the review in the context of existing knowledge | Y | Y | - | Y |
| Objectives | 4 | Provide an explicit statement of the objective(s) or question(s) the review addresses. | Y | Y | - | Y |
| **Methods** | | | | | | |
| Eligibility criteria | 5 | Specify the inclusion and exclusion criteria for the review and how studies were grouped for the syntheses. | Y | Y | - | Y |
| Information sources | 6 | Specify all databases, registers, websites, organisations, reference lists and other sources searched or consulted to identify studies. Specify the date when each source was last searched or consulted. | Y | Y | - | Y |
| Search strategy | 7 | Present the full search strategies for all databases, registers and websites, including any filters and limits used | PY | Y | Y | Y |
| Selection process | 8 | Specify the methods used to decide whether a study met the inclusion criteria of the review, including how many reviewers screened each record and each report retrieved, whether they worked independently, and if applicable, details of automation tools  used in the process. | Y | Y | - | Y |
| Data collection  process | 9 | Specify the methods used to collect data from reports, including how many reviewers collected data from each report, whether they worked independently, any processes for obtaining or confirming data from study investigators, and if applicable, details of automation tools used in the process. | Y | Y | - | Y |
| Data items | 10a | List and define all outcomes for which data were sought. Specify whether all results that were compatible with each outcome domain in each study were sought (e.g. for all measures, time points, analyses), and if not, the methods used to decide which  results to collect. | Y | Y | - | Y |
|  | 10b | List and define all other variables for which data were sought (e.g. participant and intervention characteristics, funding sources). Describe any assumptions made about any missing or unclear information. | Y | Y | - | Y |
| Study risk of bias  assessment | 11 | Specify the methods used to assess risk of bias in the included studies, including details of the tool(s) used, how many reviewers assessed each study and whether they worked independently, and if applicable, details of automation tools used in the process. | Y | Y | - | Y |
| Effect measures | 12 | Specify for each outcome the effect measure(s) (e.g. risk ratio, mean difference) used in the synthesis or presentation of results. | Y | Y | - | Y |
| Synthesis methods | 13a | Describe the processes used to decide which studies were eligible for each synthesis (e.g. tabulating the study intervention characteristics and comparing against the planned groups for each synthesis (item #5)). | Y | Y | - | Y |
|  | 13b | Describe any methods required to prepare the data for presentation or synthesis, such as handling of missing summary statistics, or data conversions. | Y | Y | - | Y |
|  | 13c | Describe any methods used to tabulate or visually display results of individual studies and syntheses. | Y | Y | - | Y |
|  | 13d | Describe any methods used to synthesise results and provide a rationale for the choice(s). If meta-analysis was performed, describe the model(s), method(s) to identify the presence and extent of statistical heterogeneity, and software package(s) used. | Y | Y | - | Y |
|  | 13e | Describe any methods used to explore possible causes of heterogeneity among study results (e.g. subgroup analysis, meta regression). | PY | Y | PY | PY |
|  | 13f | Describe any sensitivity analyses conducted to assess robustness of the synthesised results. | PY | Y | N | N |
| Reporting bias  assessment | 14 | Describe any methods used to assess risk of bias due to missing results in a synthesis (arising from reporting biases). | N | N | - | N |
| Certainty assessment | 15 | Describe any methods used to assess certainty (or confidence) in the body of evidence for an outcome. | N | N | - | N |
| **Results** | | | | | | |
| Study selection | 16a | Describe the results of the search and selection process, from the number of records identified in the search to the number of studies included in the review, ideally using a flow diagram (see fig 1). | Y | Y | - | Y |
|  | 16b | Cite studies that might appear to meet the inclusion criteria, but which were excluded, and explain why they were excluded. | PY | Y | PY | PY |
| Study characteristics | 17 | Cite each included study and present its characteristics. | Y | Y | - | Y |
| Risk of bias in studies | 18 | Present assessments of risk of bias for each included study. | Y | Y | - | Y |
| Results of individual  studies | 19 | For all outcomes, present, for each study: (a) summary statistics for each group (where appropriate) and (b) an effect estimate and its precision (e.g. confidence/credible interval), ideally using structured tables or plots. | Y | Y | - | Y |
| Results of syntheses | 20a | For each synthesis, briefly summarise the characteristics and risk of bias among contributing studies. | Y | Y | - | Y |
|  | 20b | Present results of all statistical syntheses conducted. If meta-analysis was done, present for each the summary estimate and its precision (e.g. confidence/credible interval) and measures of statistical heterogeneity. If comparing groups, describe the direction of the effect. | Y | Y | - | Y |
|  | 20c | Present results of all investigations of possible causes of heterogeneity among study results. | PY | Y | PY | PY |
|  | 20d | Present results of all sensitivity analyses conducted to assess the robustness of the synthesised results. | PY | Y | N | N |
| Reporting biases | 21 | Present assessments of risk of bias due to missing results (arising from reporting biases) for each synthesis assessed. | N | N | - | N |
| Certainty of evidence | 22 | Present assessments of certainty (or confidence) in the body of evidence for each outcome assessed. | N | N | - | N |
| **Discussion** | | | | | | |
| Discussion | 23a | Provide a general interpretation of the results in the context of other evidence. | Y | Y | - | Y |
|  | 23b | Discuss any limitations of the evidence included in the review. | Y | Y | - | Y |
|  | 23c | Discuss any limitations of the review processes used. | Y | Y | - | Y |
|  | 23d | Discuss implications of the results for practice, policy, and future research. | Y | Y | - | Y |
| **Other information** | | | | | | |
| Registration and  protocol | 24a | Provide registration information for the review, including register name and registration number, or state that the review was not registered. | N | N | - | N |
|  | 24b | Indicate where the review protocol can be accessed, or state that a protocol was not prepared. | N | N | - | N |
|  | 24c | Describe and explain any amendments to information provided at registration or in the protocol. | N | N | - | N |
| Support | 25 | Describe sources of financial or non-financial support for the review, and the role of the funders or sponsors in the review. | Y | Y | - | Y |
| Competing interests | 26 | Declare any competing interests of review authors. | N | Y | N | N |
| Availability of data,  code, and other  materials | 27 | Report which of the following are publicly available and where they can be found: template data collection forms; data extracted from included studies; data used for all analyses; analytic code; any other materials used in the review. | N | N | - | N |

12.王亚芹,樊鹏利,吕品,陈博雅,李坤,马培志.奥马珠单抗治疗儿童及青少年过敏性哮喘的系统评价[J].中国药物评价,2021,38(2):111-114

| Section and topic | Item # | Checklist item | A | B | C | D |
| --- | --- | --- | --- | --- | --- | --- |
| **Title** | | | | | | |
| Title | 1 | Identify the report as a systematic review. | Y | Y | - | Y |
| **Abstract** | | | | | | |
| Abstract | 2 | See the PRISMA 2020 for Abstracts checklist (table 2). | PY | PY | - | PY |
| **Introduction** | | | | | | |
| Rationale | 3 | Describe the rationale for the review in the context of existing knowledge | Y | Y | - | Y |
| Objectives | 4 | Provide an explicit statement of the objective(s) or question(s) the review addresses. | Y | Y | - | Y |
| **Methods** | | | | | | |
| Eligibility criteria | 5 | Specify the inclusion and exclusion criteria for the review and how studies were grouped for the syntheses. | Y | Y | - | Y |
| Information sources | 6 | Specify all databases, registers, websites, organisations, reference lists and other sources searched or consulted to identify studies. Specify the date when each source was last searched or consulted. | Y | Y | - | Y |
| Search strategy | 7 | Present the full search strategies for all databases, registers and websites, including any filters and limits used | N | PY | PY | PY |
| Selection process | 8 | Specify the methods used to decide whether a study met the inclusion criteria of the review, including how many reviewers screened each record and each report retrieved, whether they worked independently, and if applicable, details of automation tools  used in the process. | Y | Y | - | Y |
| Data collection  process | 9 | Specify the methods used to collect data from reports, including how many reviewers collected data from each report, whether they worked independently, any processes for obtaining or confirming data from study investigators, and if applicable, details of automation tools used in the process. | Y | Y | - | Y |
| Data items | 10a | List and define all outcomes for which data were sought. Specify whether all results that were compatible with each outcome domain in each study were sought (e.g. for all measures, time points, analyses), and if not, the methods used to decide which  results to collect. | Y | Y | - | Y |
|  | 10b | List and define all other variables for which data were sought (e.g. participant and intervention characteristics, funding sources). Describe any assumptions made about any missing or unclear information. | Y | Y | - | Y |
| Study risk of bias  assessment | 11 | Specify the methods used to assess risk of bias in the included studies, including details of the tool(s) used, how many reviewers assessed each study and whether they worked independently, and if applicable, details of automation tools used in the process. | Y | Y | - | Y |
| Effect measures | 12 | Specify for each outcome the effect measure(s) (e.g. risk ratio, mean difference) used in the synthesis or presentation of results. | Y | Y | - | Y |
| Synthesis methods | 13a | Describe the processes used to decide which studies were eligible for each synthesis (e.g. tabulating the study intervention characteristics and comparing against the planned groups for each synthesis (item #5)). | N | Y | Y | Y |
|  | 13b | Describe any methods required to prepare the data for presentation or synthesis, such as handling of missing summary statistics, or data conversions. | N | Y | PY | PY |
|  | 13c | Describe any methods used to tabulate or visually display results of individual studies and syntheses. | N | Y | Y | Y |
|  | 13d | Describe any methods used to synthesise results and provide a rationale for the choice(s). If meta-analysis was performed, describe the model(s), method(s) to identify the presence and extent of statistical heterogeneity, and software package(s) used. | Y | Y | - | Y |
|  | 13e | Describe any methods used to explore possible causes of heterogeneity among study results (e.g. subgroup analysis, meta regression). | N | PY | PY | PY |
|  | 13f | Describe any sensitivity analyses conducted to assess robustness of the synthesised results. | N | PY | PY | PY |
| Reporting bias  assessment | 14 | Describe any methods used to assess risk of bias due to missing results in a synthesis (arising from reporting biases). | N | PY | PY | PY |
| Certainty assessment | 15 | Describe any methods used to assess certainty (or confidence) in the body of evidence for an outcome. | N | N | - | N |
| **Results** | | | | | | |
| Study selection | 16a | Describe the results of the search and selection process, from the number of records identified in the search to the number of studies included in the review, ideally using a flow diagram (see fig 1). | Y | Y | - | Y |
|  | 16b | Cite studies that might appear to meet the inclusion criteria, but which were excluded, and explain why they were excluded. | N | PY | PY | PY |
| Study characteristics | 17 | Cite each included study and present its characteristics. | Y | Y | - | Y |
| Risk of bias in studies | 18 | Present assessments of risk of bias for each included study. | Y | Y | - | Y |
| Results of individual  studies | 19 | For all outcomes, present, for each study: (a) summary statistics for each group (where appropriate) and (b) an effect estimate and its precision (e.g. confidence/credible interval), ideally using structured tables or plots. | PY | Y | Y | Y |
| Results of syntheses | 20a | For each synthesis, briefly summarise the characteristics and risk of bias among contributing studies. | Y | Y | - | Y |
|  | 20b | Present results of all statistical syntheses conducted. If meta-analysis was done, present for each the summary estimate and its precision (e.g. confidence/credible interval) and measures of statistical heterogeneity. If comparing groups, describe the direction of the effect. | Y | Y | - | Y |
|  | 20c | Present results of all investigations of possible causes of heterogeneity among study results. | N | PY | PY | PY |
|  | 20d | Present results of all sensitivity analyses conducted to assess the robustness of the synthesised results. | N | PY | PY | PY |
| Reporting biases | 21 | Present assessments of risk of bias due to missing results (arising from reporting biases) for each synthesis assessed. | N | PY | PY | PY |
| Certainty of evidence | 22 | Present assessments of certainty (or confidence) in the body of evidence for each outcome assessed. | N | N | - | N |
| **Discussion** | | | | | | |
| Discussion | 23a | Provide a general interpretation of the results in the context of other evidence. | Y | Y | - | Y |
|  | 23b | Discuss any limitations of the evidence included in the review. | Y | Y | - | Y |
|  | 23c | Discuss any limitations of the review processes used. | Y | Y | - | Y |
|  | 23d | Discuss implications of the results for practice, policy, and future research. | Y | Y | - | Y |
| **Other information** | | | | | | |
| Registration and  protocol | 24a | Provide registration information for the review, including register name and registration number, or state that the review was not registered. | N | N | - | N |
|  | 24b | Indicate where the review protocol can be accessed, or state that a protocol was not prepared. | N | N | - | N |
|  | 24c | Describe and explain any amendments to information provided at registration or in the protocol. | N | N | - | N |
| Support | 25 | Describe sources of financial or non-financial support for the review, and the role of the funders or sponsors in the review. | Y | PY | Y | Y |
| Competing interests | 26 | Declare any competing interests of review authors. | N | N | - | N |
| Availability of data,  code, and other  materials | 27 | Report which of the following are publicly available and where they can be found: template data collection forms; data extracted from included studies; data used for all analyses; analytic code; any other materials used in the review. | N | N | - | N |

13.陈雪琴,贾心予,吴晶晶,等. 奥马珠单抗治疗难治性过敏性哮喘疗效和安全性的荟萃分析[J]. 中华医学杂志,2022,102(28):2201-2209. DOI:10.3760/cma.j.cn112137-20211109-02480.

| Section and topic | Item # | Checklist item | A | B | C | D |
| --- | --- | --- | --- | --- | --- | --- |
| **Title** | | | | | | |
| Title | 1 | Identify the report as a systematic review. | Y | Y | - | Y |
| **Abstract** | | | | | | |
| Abstract | 2 | See the PRISMA 2020 for Abstracts checklist (table 2). | PY | PY | - | PY |
| **Introduction** | | | | | | |
| Rationale | 3 | Describe the rationale for the review in the context of existing knowledge | Y | Y | - | Y |
| Objectives | 4 | Provide an explicit statement of the objective(s) or question(s) the review addresses. | Y | Y | - | Y |
| **Methods** | | | | | | |
| Eligibility criteria | 5 | Specify the inclusion and exclusion criteria for the review and how studies were grouped for the syntheses. | Y | Y | - | Y |
| Information sources | 6 | Specify all databases, registers, websites, organisations, reference lists and other sources searched or consulted to identify studies. Specify the date when each source was last searched or consulted. | Y | Y | - | Y |
| Search strategy | 7 | Present the full search strategies for all databases, registers and websites, including any filters and limits used | Y | Y | - | Y |
| Selection process | 8 | Specify the methods used to decide whether a study met the inclusion criteria of the review, including how many reviewers screened each record and each report retrieved, whether they worked independently, and if applicable, details of automation tools  used in the process. | Y | Y | - | Y |
| Data collection  process | 9 | Specify the methods used to collect data from reports, including how many reviewers collected data from each report, whether they worked independently, any processes for obtaining or confirming data from study investigators, and if applicable, details of automation tools used in the process. | Y | Y | - | Y |
| Data items | 10a | List and define all outcomes for which data were sought. Specify whether all results that were compatible with each outcome domain in each study were sought (e.g. for all measures, time points, analyses), and if not, the methods used to decide which  results to collect. | Y | Y | - | Y |
|  | 10b | List and define all other variables for which data were sought (e.g. participant and intervention characteristics, funding sources). Describe any assumptions made about any missing or unclear information. | Y | Y | - | Y |
| Study risk of bias  assessment | 11 | Specify the methods used to assess risk of bias in the included studies, including details of the tool(s) used, how many reviewers assessed each study and whether they worked independently, and if applicable, details of automation tools used in the process. | Y | Y | - | Y |
| Effect measures | 12 | Specify for each outcome the effect measure(s) (e.g. risk ratio, mean difference) used in the synthesis or presentation of results. | Y | Y | - | Y |
| Synthesis methods | 13a | Describe the processes used to decide which studies were eligible for each synthesis (e.g. tabulating the study intervention characteristics and comparing against the planned groups for each synthesis (item #5)). | Y | Y | - | Y |
|  | 13b | Describe any methods required to prepare the data for presentation or synthesis, such as handling of missing summary statistics, or data conversions. | Y | Y | - | Y |
|  | 13c | Describe any methods used to tabulate or visually display results of individual studies and syntheses. | Y | Y | - | Y |
|  | 13d | Describe any methods used to synthesise results and provide a rationale for the choice(s). If meta-analysis was performed, describe the model(s), method(s) to identify the presence and extent of statistical heterogeneity, and software package(s) used. | Y | Y | - | Y |
|  | 13e | Describe any methods used to explore possible causes of heterogeneity among study results (e.g. subgroup analysis, meta regression). | Y | Y | - | Y |
|  | 13f | Describe any sensitivity analyses conducted to assess robustness of the synthesised results. | Y | Y | - | Y |
| Reporting bias  assessment | 14 | Describe any methods used to assess risk of bias due to missing results in a synthesis (arising from reporting biases). | Y | Y | - | Y |
| Certainty assessment | 15 | Describe any methods used to assess certainty (or confidence) in the body of evidence for an outcome. | N | N | - | N |
| **Results** | | | | | | |
| Study selection | 16a | Describe the results of the search and selection process, from the number of records identified in the search to the number of studies included in the review, ideally using a flow diagram (see fig 1). | Y | Y | - | Y |
|  | 16b | Cite studies that might appear to meet the inclusion criteria, but which were excluded, and explain why they were excluded. | Y | Y | - | Y |
| Study characteristics | 17 | Cite each included study and present its characteristics. | Y | Y | - | Y |
| Risk of bias in studies | 18 | Present assessments of risk of bias for each included study. | Y | Y | - | Y |
| Results of individual  studies | 19 | For all outcomes, present, for each study: (a) summary statistics for each group (where appropriate) and (b) an effect estimate and its precision (e.g. confidence/credible interval), ideally using structured tables or plots. | Y | Y | - | Y |
| Results of syntheses | 20a | For each synthesis, briefly summarise the characteristics and risk of bias among contributing studies. | Y | Y | - | Y |
|  | 20b | Present results of all statistical syntheses conducted. If meta-analysis was done, present for each the summary estimate and its precision (e.g. confidence/credible interval) and measures of statistical heterogeneity. If comparing groups, describe the direction of the effect. | Y | Y | - | Y |
|  | 20c | Present results of all investigations of possible causes of heterogeneity among study results. | Y | Y | - | Y |
|  | 20d | Present results of all sensitivity analyses conducted to assess the robustness of the synthesised results. | Y | Y | - | Y |
| Reporting biases | 21 | Present assessments of risk of bias due to missing results (arising from reporting biases) for each synthesis assessed. | Y | Y | - | Y |
| Certainty of evidence | 22 | Present assessments of certainty (or confidence) in the body of evidence for each outcome assessed. | N | N | - | N |
| **Discussion** | | | | | | |
| Discussion | 23a | Provide a general interpretation of the results in the context of other evidence. | Y | Y | - | Y |
|  | 23b | Discuss any limitations of the evidence included in the review. | Y | Y | - | Y |
|  | 23c | Discuss any limitations of the review processes used. | Y | Y | - | Y |
|  | 23d | Discuss implications of the results for practice, policy, and future research. | Y | Y | - | Y |
| **Other information** | | | | | | |
| Registration and  protocol | 24a | Provide registration information for the review, including register name and registration number, or state that the review was not registered. | N | N | - | N |
|  | 24b | Indicate where the review protocol can be accessed, or state that a protocol was not prepared. | N | N | - | N |
|  | 24c | Describe and explain any amendments to information provided at registration or in the protocol. | N | N | - | N |
| Support | 25 | Describe sources of financial or non-financial support for the review, and the role of the funders or sponsors in the review. | Y | Y | - | Y |
| Competing interests | 26 | Declare any competing interests of review authors. | Y | Y | - | Y |
| Availability of data,  code, and other  materials | 27 | Report which of the following are publicly available and where they can be found: template data collection forms; data extracted from included studies; data used for all analyses; analytic code; any other materials used in the review. | N | N | - | N |

14.Fenu G, La Tessa A, Calogero C, Lombardi E. Severe pediatric asthma therapy: Omalizumab-A systematic review and meta-analysis of efficacy and safety profile. Front Pediatr. 2023 Mar 3;10:1033511. doi: 10.3389/fped.2022.1033511. PMID: 36937051; PMCID: PMC10020639.

| Section and topic | Item # | Checklist item | A | B | C | D |
| --- | --- | --- | --- | --- | --- | --- |
| **Title** | | | | | | |
| Title | 1 | Identify the report as a systematic review. | Y | Y | - | Y |
| **Abstract** | | | | | | |
| Abstract | 2 | See the PRISMA 2020 for Abstracts checklist (table 2). | PY | PY | - | PY |
| **Introduction** | | | | | | |
| Rationale | 3 | Describe the rationale for the review in the context of existing knowledge | Y | Y | - | Y |
| Objectives | 4 | Provide an explicit statement of the objective(s) or question(s) the review addresses. | Y | Y | - | Y |
| **Methods** | | | | | | |
| Eligibility criteria | 5 | Specify the inclusion and exclusion criteria for the review and how studies were grouped for the syntheses. | Y | Y | - | Y |
| Information sources | 6 | Specify all databases, registers, websites, organisations, reference lists and other sources searched or consulted to identify studies. Specify the date when each source was last searched or consulted. | Y | Y | - | Y |
| Search strategy | 7 | Present the full search strategies for all databases, registers and websites, including any filters and limits used | N | PY | PY | PY |
| Selection process | 8 | Specify the methods used to decide whether a study met the inclusion criteria of the review, including how many reviewers screened each record and each report retrieved, whether they worked independently, and if applicable, details of automation tools  used in the process. | PY | PY | - | PY |
| Data collection  process | 9 | Specify the methods used to collect data from reports, including how many reviewers collected data from each report, whether they worked independently, any processes for obtaining or confirming data from study investigators, and if applicable, details of automation tools used in the process. | PY | PY | - | PY |
| Data items | 10a | List and define all outcomes for which data were sought. Specify whether all results that were compatible with each outcome domain in each study were sought (e.g. for all measures, time points, analyses), and if not, the methods used to decide which  results to collect. | Y | Y | - | Y |
|  | 10b | List and define all other variables for which data were sought (e.g. participant and intervention characteristics, funding sources). Describe any assumptions made about any missing or unclear information. | Y | Y | - | Y |
| Study risk of bias  assessment | 11 | Specify the methods used to assess risk of bias in the included studies, including details of the tool(s) used, how many reviewers assessed each study and whether they worked independently, and if applicable, details of automation tools used in the process. | PY | Y | Y | Y |
| Effect measures | 12 | Specify for each outcome the effect measure(s) (e.g. risk ratio, mean difference) used in the synthesis or presentation of results. | Y | Y | - | Y |
| Synthesis methods | 13a | Describe the processes used to decide which studies were eligible for each synthesis (e.g. tabulating the study intervention characteristics and comparing against the planned groups for each synthesis (item #5)). | N | Y | Y | Y |
|  | 13b | Describe any methods required to prepare the data for presentation or synthesis, such as handling of missing summary statistics, or data conversions. | N | PY | Y | Y |
|  | 13c | Describe any methods used to tabulate or visually display results of individual studies and syntheses. | Y | Y | - | Y |
|  | 13d | Describe any methods used to synthesise results and provide a rationale for the choice(s). If meta-analysis was performed, describe the model(s), method(s) to identify the presence and extent of statistical heterogeneity, and software package(s) used. | N | Y | Y | Y |
|  | 13e | Describe any methods used to explore possible causes of heterogeneity among study results (e.g. subgroup analysis, meta regression). | N | PY | Y | Y |
|  | 13f | Describe any sensitivity analyses conducted to assess robustness of the synthesised results. | N | PY | Y | Y |
| Reporting bias  assessment | 14 | Describe any methods used to assess risk of bias due to missing results in a synthesis (arising from reporting biases). | N | PY | PY | PY |
| Certainty assessment | 15 | Describe any methods used to assess certainty (or confidence) in the body of evidence for an outcome. | N | PY | PY | PY |
| **Results** | | | | | | |
| Study selection | 16a | Describe the results of the search and selection process, from the number of records identified in the search to the number of studies included in the review, ideally using a flow diagram (see fig 1). | Y | Y | - | Y |
|  | 16b | Cite studies that might appear to meet the inclusion criteria, but which were excluded, and explain why they were excluded. | N | N | - | N |
| Study characteristics | 17 | Cite each included study and present its characteristics. | Y | Y | - | Y |
| Risk of bias in studies | 18 | Present assessments of risk of bias for each included study. | N | Y | Y | Y |
| Results of individual  studies | 19 | For all outcomes, present, for each study: (a) summary statistics for each group (where appropriate) and (b) an effect estimate and its precision (e.g. confidence/credible interval), ideally using structured tables or plots. | Y | Y | - | Y |
| Results of syntheses | 20a | For each synthesis, briefly summarise the characteristics and risk of bias among contributing studies. | Y | Y | - | Y |
|  | 20b | Present results of all statistical syntheses conducted. If meta-analysis was done, present for each the summary estimate and its precision (e.g. confidence/credible interval) and measures of statistical heterogeneity. If comparing groups, describe the direction of the effect. | Y | Y | - | Y |
|  | 20c | Present results of all investigations of possible causes of heterogeneity among study results. | N | PY | Y | Y |
|  | 20d | Present results of all sensitivity analyses conducted to assess the robustness of the synthesised results. | N | PY | Y | Y |
| Reporting biases | 21 | Present assessments of risk of bias due to missing results (arising from reporting biases) for each synthesis assessed. | N | PY | PY | PY |
| Certainty of evidence | 22 | Present assessments of certainty (or confidence) in the body of evidence for each outcome assessed. | N | PY | PY | PY |
| **Discussion** | | | | | | |
| Discussion | 23a | Provide a general interpretation of the results in the context of other evidence. | Y | Y | - | Y |
|  | 23b | Discuss any limitations of the evidence included in the review. | Y | Y | - | Y |
|  | 23c | Discuss any limitations of the review processes used. | Y | Y | - | Y |
|  | 23d | Discuss implications of the results for practice, policy, and future research. | Y | Y | - | Y |
| **Other information** | | | | | | |
| Registration and  protocol | 24a | Provide registration information for the review, including register name and registration number, or state that the review was not registered. | Y | Y | - | Y |
|  | 24b | Indicate where the review protocol can be accessed, or state that a protocol was not prepared. | N | Y | PY | PY |
|  | 24c | Describe and explain any amendments to information provided at registration or in the protocol. | N | Y | PY | PY |
| Support | 25 | Describe sources of financial or non-financial support for the review, and the role of the funders or sponsors in the review. | Y | Y | - | Y |
| Competing interests | 26 | Declare any competing interests of review authors. | Y | Y | - | Y |
| Availability of data,  code, and other  materials | 27 | Report which of the following are publicly available and where they can be found: template data collection forms; data extracted from included studies; data used for all analyses; analytic code; any other materials used in the review. | N | PY | Y | Y |

15.Lang D, Liu Z, Li D. Safety and Tolerability of Omalizumab in Children with Allergic (IgE-Mediated) Asthma: A Systematic Review and Meta-Analysis. Discov Med. 2023 Jun;35(176):233-241. doi: 10.24976/Discov.Med.202335176.24. PMID: 37272090.

| Section and topic | Item # | Checklist item | A | B | C | D |
| --- | --- | --- | --- | --- | --- | --- |
| **Title** | | | | | | |
| Title | 1 | Identify the report as a systematic review. | Y | Y | - | Y |
| **Abstract** | | | | | | |
| Abstract | 2 | See the PRISMA 2020 for Abstracts checklist (table 2). | PY | PY | - | PY |
| **Introduction** | | | | | | |
| Rationale | 3 | Describe the rationale for the review in the context of existing knowledge | Y | Y | - | Y |
| Objectives | 4 | Provide an explicit statement of the objective(s) or question(s) the review addresses. | Y | Y | - | Y |
| **Methods** | | | | | | |
| Eligibility criteria | 5 | Specify the inclusion and exclusion criteria for the review and how studies were grouped for the syntheses. | Y | Y | - | Y |
| Information sources | 6 | Specify all databases, registers, websites, organisations, reference lists and other sources searched or consulted to identify studies. Specify the date when each source was last searched or consulted. | Y | Y | - | Y |
| Search strategy | 7 | Present the full search strategies for all databases, registers and websites, including any filters and limits used | PY | PY | - | PY |
| Selection process | 8 | Specify the methods used to decide whether a study met the inclusion criteria of the review, including how many reviewers screened each record and each report retrieved, whether they worked independently, and if applicable, details of automation tools  used in the process. | Y | Y | - | Y |
| Data collection  process | 9 | Specify the methods used to collect data from reports, including how many reviewers collected data from each report, whether they worked independently, any processes for obtaining or confirming data from study investigators, and if applicable, details of automation tools used in the process. | Y | Y | - | Y |
| Data items | 10a | List and define all outcomes for which data were sought. Specify whether all results that were compatible with each outcome domain in each study were sought (e.g. for all measures, time points, analyses), and if not, the methods used to decide which  results to collect. | Y | Y | - | Y |
|  | 10b | List and define all other variables for which data were sought (e.g. participant and intervention characteristics, funding sources). Describe any assumptions made about any missing or unclear information. | Y | Y | - | Y |
| Study risk of bias  assessment | 11 | Specify the methods used to assess risk of bias in the included studies, including details of the tool(s) used, how many reviewers assessed each study and whether they worked independently, and if applicable, details of automation tools used in the process. | Y | Y | - | Y |
| Effect measures | 12 | Specify for each outcome the effect measure(s) (e.g. risk ratio, mean difference) used in the synthesis or presentation of results. | Y | Y | - | Y |
| Synthesis methods | 13a | Describe the processes used to decide which studies were eligible for each synthesis (e.g. tabulating the study intervention characteristics and comparing against the planned groups for each synthesis (item #5)). | Y | Y | - | Y |
|  | 13b | Describe any methods required to prepare the data for presentation or synthesis, such as handling of missing summary statistics, or data conversions. | Y | Y | - | Y |
|  | 13c | Describe any methods used to tabulate or visually display results of individual studies and syntheses. | Y | Y | - | Y |
|  | 13d | Describe any methods used to synthesise results and provide a rationale for the choice(s). If meta-analysis was performed, describe the model(s), method(s) to identify the presence and extent of statistical heterogeneity, and software package(s) used. | Y | Y | - | Y |
|  | 13e | Describe any methods used to explore possible causes of heterogeneity among study results (e.g. subgroup analysis, meta regression). | Y | Y | - | Y |
|  | 13f | Describe any sensitivity analyses conducted to assess robustness of the synthesised results. | Y | Y | - | Y |
| Reporting bias  assessment | 14 | Describe any methods used to assess risk of bias due to missing results in a synthesis (arising from reporting biases). | Y | Y | - | Y |
| Certainty assessment | 15 | Describe any methods used to assess certainty (or confidence) in the body of evidence for an outcome. | N | N | - | N |
| **Results** | | | | | | |
| Study selection | 16a | Describe the results of the search and selection process, from the number of records identified in the search to the number of studies included in the review, ideally using a flow diagram (see fig 1). | Y | Y | - | Y |
|  | 16b | Cite studies that might appear to meet the inclusion criteria, but which were excluded, and explain why they were excluded. | PY | N | PY | PY |
| Study characteristics | 17 | Cite each included study and present its characteristics. | Y | Y | - | Y |
| Risk of bias in studies | 18 | Present assessments of risk of bias for each included study. | Y | Y | - | Y |
| Results of individual  studies | 19 | For all outcomes, present, for each study: (a) summary statistics for each group (where appropriate) and (b) an effect estimate and its precision (e.g. confidence/credible interval), ideally using structured tables or plots. | Y | Y | - | Y |
| Results of syntheses | 20a | For each synthesis, briefly summarise the characteristics and risk of bias among contributing studies. | Y | Y | - | Y |
|  | 20b | Present results of all statistical syntheses conducted. If meta-analysis was done, present for each the summary estimate and its precision (e.g. confidence/credible interval) and measures of statistical heterogeneity. If comparing groups, describe the direction of the effect. | Y | Y | - | Y |
|  | 20c | Present results of all investigations of possible causes of heterogeneity among study results. | Y | Y | - | Y |
|  | 20d | Present results of all sensitivity analyses conducted to assess the robustness of the synthesised results. | Y | Y | - | Y |
| Reporting biases | 21 | Present assessments of risk of bias due to missing results (arising from reporting biases) for each synthesis assessed. | Y | Y | - | Y |
| Certainty of evidence | 22 | Present assessments of certainty (or confidence) in the body of evidence for each outcome assessed. | N | N | - | N |
| **Discussion** | | | | | | |
| Discussion | 23a | Provide a general interpretation of the results in the context of other evidence. | Y | Y | - | Y |
|  | 23b | Discuss any limitations of the evidence included in the review. | Y | Y | - | Y |
|  | 23c | Discuss any limitations of the review processes used. | Y | Y | - | Y |
|  | 23d | Discuss implications of the results for practice, policy, and future research. | Y | Y | - | Y |
| **Other information** | | | | | | |
| Registration and  protocol | 24a | Provide registration information for the review, including register name and registration number, or state that the review was not registered. | N | N | - | N |
|  | 24b | Indicate where the review protocol can be accessed, or state that a protocol was not prepared. | N | N | - | N |
|  | 24c | Describe and explain any amendments to information provided at registration or in the protocol. | N | N | - | N |
| Support | 25 | Describe sources of financial or non-financial support for the review, and the role of the funders or sponsors in the review. | Y | Y | - | Y |
| Competing interests | 26 | Declare any competing interests of review authors. | Y | Y | - | Y |
| Availability of data,  code, and other  materials | 27 | Report which of the following are publicly available and where they can be found: template data collection forms; data extracted from included studies; data used for all analyses; analytic code; any other materials used in the review. | N | N | - | N |

16.阮俊文.抗IgE单克隆抗体在支气管哮喘患者升阶梯治疗中有效性和安全性的系统评价与Meta分析[D].赣南医学院,2023.DOI:10.27959/d.cnki.ggnyx.2023.000137.

| Section and topic | Item # | Checklist item | A | B | C | D |
| --- | --- | --- | --- | --- | --- | --- |
| **Title** | | | | | | |
| Title | 1 | Identify the report as a systematic review. | Y | Y | - | Y |
| **Abstract** | | | | | | |
| Abstract | 2 | See the PRISMA 2020 for Abstracts checklist (table 2). | PY | PY | - | PY |
| **Introduction** | | | | | | |
| Rationale | 3 | Describe the rationale for the review in the context of existing knowledge | Y | Y | - | Y |
| Objectives | 4 | Provide an explicit statement of the objective(s) or question(s) the review addresses. | Y | Y | - | Y |
| **Methods** | | | | | | |
| Eligibility criteria | 5 | Specify the inclusion and exclusion criteria for the review and how studies were grouped for the syntheses. | Y | Y | - | Y |
| Information sources | 6 | Specify all databases, registers, websites, organisations, reference lists and other sources searched or consulted to identify studies. Specify the date when each source was last searched or consulted. | Y | Y | - | Y |
| Search strategy | 7 | Present the full search strategies for all databases, registers and websites, including any filters and limits used | PY | PY | - | PY |
| Selection process | 8 | Specify the methods used to decide whether a study met the inclusion criteria of the review, including how many reviewers screened each record and each report retrieved, whether they worked independently, and if applicable, details of automation tools  used in the process. | Y | Y | - | Y |
| Data collection  process | 9 | Specify the methods used to collect data from reports, including how many reviewers collected data from each report, whether they worked independently, any processes for obtaining or confirming data from study investigators, and if applicable, details of automation tools used in the process. | Y | Y | - | Y |
| Data items | 10a | List and define all outcomes for which data were sought. Specify whether all results that were compatible with each outcome domain in each study were sought (e.g. for all measures, time points, analyses), and if not, the methods used to decide which  results to collect. | Y | Y | - | Y |
|  | 10b | List and define all other variables for which data were sought (e.g. participant and intervention characteristics, funding sources). Describe any assumptions made about any missing or unclear information. | Y | Y | - | Y |
| Study risk of bias  assessment | 11 | Specify the methods used to assess risk of bias in the included studies, including details of the tool(s) used, how many reviewers assessed each study and whether they worked independently, and if applicable, details of automation tools used in the process. | Y | Y | - | Y |
| Effect measures | 12 | Specify for each outcome the effect measure(s) (e.g. risk ratio, mean difference) used in the synthesis or presentation of results. | Y | Y | - | Y |
| Synthesis methods | 13a | Describe the processes used to decide which studies were eligible for each synthesis (e.g. tabulating the study intervention characteristics and comparing against the planned groups for each synthesis (item #5)). | Y | Y | - | Y |
|  | 13b | Describe any methods required to prepare the data for presentation or synthesis, such as handling of missing summary statistics, or data conversions. | Y | Y | - | Y |
|  | 13c | Describe any methods used to tabulate or visually display results of individual studies and syntheses. | Y | Y | - | Y |
|  | 13d | Describe any methods used to synthesise results and provide a rationale for the choice(s). If meta-analysis was performed, describe the model(s), method(s) to identify the presence and extent of statistical heterogeneity, and software package(s) used. | Y | Y | - | Y |
|  | 13e | Describe any methods used to explore possible causes of heterogeneity among study results (e.g. subgroup analysis, meta regression). | Y | Y | - | Y |
|  | 13f | Describe any sensitivity analyses conducted to assess robustness of the synthesised results. | PY | N | Y | Y |
| Reporting bias  assessment | 14 | Describe any methods used to assess risk of bias due to missing results in a synthesis (arising from reporting biases). | PY | PY | - | PY |
| Certainty assessment | 15 | Describe any methods used to assess certainty (or confidence) in the body of evidence for an outcome. | N | PY | PY | PY |
| **Results** | | | | | | |
| Study selection | 16a | Describe the results of the search and selection process, from the number of records identified in the search to the number of studies included in the review, ideally using a flow diagram (see fig 1). | Y | Y | - | Y |
|  | 16b | Cite studies that might appear to meet the inclusion criteria, but which were excluded, and explain why they were excluded. | Y | Y | - | Y |
| Study characteristics | 17 | Cite each included study and present its characteristics. | Y | Y | - | Y |
| Risk of bias in studies | 18 | Present assessments of risk of bias for each included study. | Y | Y | - | Y |
| Results of individual  studies | 19 | For all outcomes, present, for each study: (a) summary statistics for each group (where appropriate) and (b) an effect estimate and its precision (e.g. confidence/credible interval), ideally using structured tables or plots. | Y | Y | - | Y |
| Results of syntheses | 20a | For each synthesis, briefly summarise the characteristics and risk of bias among contributing studies. | Y | Y | - | Y |
|  | 20b | Present results of all statistical syntheses conducted. If meta-analysis was done, present for each the summary estimate and its precision (e.g. confidence/credible interval) and measures of statistical heterogeneity. If comparing groups, describe the direction of the effect. | Y | Y | - | Y |
|  | 20c | Present results of all investigations of possible causes of heterogeneity among study results. | Y | Y | - | Y |
|  | 20d | Present results of all sensitivity analyses conducted to assess the robustness of the synthesised results. | PY | PY | - | PY |
| Reporting biases | 21 | Present assessments of risk of bias due to missing results (arising from reporting biases) for each synthesis assessed. | PY | PY | - | PY |
| Certainty of evidence | 22 | Present assessments of certainty (or confidence) in the body of evidence for each outcome assessed. | N | PY | PY | PY |
| **Discussion** | | | | | | |
| Discussion | 23a | Provide a general interpretation of the results in the context of other evidence. | Y | Y | - | Y |
|  | 23b | Discuss any limitations of the evidence included in the review. | Y | Y | - | Y |
|  | 23c | Discuss any limitations of the review processes used. | Y | Y | - | Y |
|  | 23d | Discuss implications of the results for practice, policy, and future research. | Y | Y | - | Y |
| **Other information** | | | | | | |
| Registration and  protocol | 24a | Provide registration information for the review, including register name and registration number, or state that the review was not registered. | N | N | - | N |
|  | 24b | Indicate where the review protocol can be accessed, or state that a protocol was not prepared. | N | N | - | N |
|  | 24c | Describe and explain any amendments to information provided at registration or in the protocol. | N | N | - | N |
| Support | 25 | Describe sources of financial or non-financial support for the review, and the role of the funders or sponsors in the review. | N | PY | PY | PY |
| Competing interests | 26 | Declare any competing interests of review authors. | N | N | - | N |
| Availability of data,  code, and other  materials | 27 | Report which of the following are publicly available and where they can be found: template data collection forms; data extracted from included studies; data used for all analyses; analytic code; any other materials used in the review. | N | N | - | N |

17.薛宽宽.奥马珠单抗治疗中重度过敏性哮喘效果的Meta分析[D].山西医科大学,2023.DOI:10.27288/d.cnki.gsxyu.2023.000328.

| Section and topic | Item # | Checklist item | A | B | C | D |
| --- | --- | --- | --- | --- | --- | --- |
| **Title** | | | | | | |
| Title | 1 | Identify the report as a systematic review. | Y | Y | - | Y |
| **Abstract** | | | | | | |
| Abstract | 2 | See the PRISMA 2020 for Abstracts checklist (table 2). | PY | PY | - | PY |
| **Introduction** | | | | | | |
| Rationale | 3 | Describe the rationale for the review in the context of existing knowledge | Y | Y | - | Y |
| Objectives | 4 | Provide an explicit statement of the objective(s) or question(s) the review addresses. | Y | Y | - | Y |
| **Methods** | | | | | | |
| Eligibility criteria | 5 | Specify the inclusion and exclusion criteria for the review and how studies were grouped for the syntheses. | Y | Y | - | Y |
| Information sources | 6 | Specify all databases, registers, websites, organisations, reference lists and other sources searched or consulted to identify studies. Specify the date when each source was last searched or consulted. | PY | Y | PY | PY |
| Search strategy | 7 | Present the full search strategies for all databases, registers and websites, including any filters and limits used | N | Y | N | N |
| Selection process | 8 | Specify the methods used to decide whether a study met the inclusion criteria of the review, including how many reviewers screened each record and each report retrieved, whether they worked independently, and if applicable, details of automation tools  used in the process. | Y | Y | - | Y |
| Data collection  process | 9 | Specify the methods used to collect data from reports, including how many reviewers collected data from each report, whether they worked independently, any processes for obtaining or confirming data from study investigators, and if applicable, details of automation tools used in the process. | Y | Y | - | Y |
| Data items | 10a | List and define all outcomes for which data were sought. Specify whether all results that were compatible with each outcome domain in each study were sought (e.g. for all measures, time points, analyses), and if not, the methods used to decide which  results to collect. | Y | Y | - | Y |
|  | 10b | List and define all other variables for which data were sought (e.g. participant and intervention characteristics, funding sources). Describe any assumptions made about any missing or unclear information. | Y | Y | - | Y |
| Study risk of bias  assessment | 11 | Specify the methods used to assess risk of bias in the included studies, including details of the tool(s) used, how many reviewers assessed each study and whether they worked independently, and if applicable, details of automation tools used in the process. | Y | Y | - | Y |
| Effect measures | 12 | Specify for each outcome the effect measure(s) (e.g. risk ratio, mean difference) used in the synthesis or presentation of results. | Y | Y | - | Y |
| Synthesis methods | 13a | Describe the processes used to decide which studies were eligible for each synthesis (e.g. tabulating the study intervention characteristics and comparing against the planned groups for each synthesis (item #5)). | Y | Y | - | Y |
|  | 13b | Describe any methods required to prepare the data for presentation or synthesis, such as handling of missing summary statistics, or data conversions. | Y | Y | - | Y |
|  | 13c | Describe any methods used to tabulate or visually display results of individual studies and syntheses. | Y | Y | - | Y |
|  | 13d | Describe any methods used to synthesise results and provide a rationale for the choice(s). If meta-analysis was performed, describe the model(s), method(s) to identify the presence and extent of statistical heterogeneity, and software package(s) used. | Y | Y | - | Y |
|  | 13e | Describe any methods used to explore possible causes of heterogeneity among study results (e.g. subgroup analysis, meta regression). | Y | Y | - | Y |
|  | 13f | Describe any sensitivity analyses conducted to assess robustness of the synthesised results. | Y | Y | - | Y |
| Reporting bias  assessment | 14 | Describe any methods used to assess risk of bias due to missing results in a synthesis (arising from reporting biases). | N | Y | N | N |
| Certainty assessment | 15 | Describe any methods used to assess certainty (or confidence) in the body of evidence for an outcome. | N | Y | N | N |
| **Results** | | | | | | |
| Study selection | 16a | Describe the results of the search and selection process, from the number of records identified in the search to the number of studies included in the review, ideally using a flow diagram (see fig 1). | PY | Y | Y | Y |
|  | 16b | Cite studies that might appear to meet the inclusion criteria, but which were excluded, and explain why they were excluded. | Y | Y | - | Y |
| Study characteristics | 17 | Cite each included study and present its characteristics. | Y | Y | - | Y |
| Risk of bias in studies | 18 | Present assessments of risk of bias for each included study. | Y | Y | - | Y |
| Results of individual  studies | 19 | For all outcomes, present, for each study: (a) summary statistics for each group (where appropriate) and (b) an effect estimate and its precision (e.g. confidence/credible interval), ideally using structured tables or plots. | Y | Y | - | Y |
| Results of syntheses | 20a | For each synthesis, briefly summarise the characteristics and risk of bias among contributing studies. | Y | Y | - | Y |
|  | 20b | Present results of all statistical syntheses conducted. If meta-analysis was done, present for each the summary estimate and its precision (e.g. confidence/credible interval) and measures of statistical heterogeneity. If comparing groups, describe the direction of the effect. | Y | Y | - | Y |
|  | 20c | Present results of all investigations of possible causes of heterogeneity among study results. | Y | Y | - | Y |
|  | 20d | Present results of all sensitivity analyses conducted to assess the robustness of the synthesised results. | Y | Y | - | Y |
| Reporting biases | 21 | Present assessments of risk of bias due to missing results (arising from reporting biases) for each synthesis assessed. | N | Y | N | N |
| Certainty of evidence | 22 | Present assessments of certainty (or confidence) in the body of evidence for each outcome assessed. | N | Y | N | N |
| **Discussion** | | | | | | |
| Discussion | 23a | Provide a general interpretation of the results in the context of other evidence. | Y | Y | - | Y |
|  | 23b | Discuss any limitations of the evidence included in the review. | Y | Y | - | Y |
|  | 23c | Discuss any limitations of the review processes used. | Y | Y | - | Y |
|  | 23d | Discuss implications of the results for practice, policy, and future research. | Y | Y | - | Y |
| **Other information** | | | | | | |
| Registration and  protocol | 24a | Provide registration information for the review, including register name and registration number, or state that the review was not registered. | N | N | - | N |
|  | 24b | Indicate where the review protocol can be accessed, or state that a protocol was not prepared. | N | N | - | N |
|  | 24c | Describe and explain any amendments to information provided at registration or in the protocol. | N | N | - | N |
| Support | 25 | Describe sources of financial or non-financial support for the review, and the role of the funders or sponsors in the review. | N | Y | N | N |
| Competing interests | 26 | Declare any competing interests of review authors. | N | Y | N | N |
| Availability of data,  code, and other  materials | 27 | Report which of the following are publicly available and where they can be found: template data collection forms; data extracted from included studies; data used for all analyses; analytic code; any other materials used in the review. | N | N | - | N |

18.廖浚邑.奥马珠单抗治疗对中重度过敏性哮喘患者肺功能改善的Meta分析[D].重庆医科大学,2024.DOI:10.27674/d.cnki.gcyku.2024.000768.

| Section and topic | Item # | Checklist item | A | B | C | D |
| --- | --- | --- | --- | --- | --- | --- |
| **Title** | | | | | | |
| Title | 1 | Identify the report as a systematic review. | Y | Y | - | Y |
| **Abstract** | | | | | | |
| Abstract | 2 | See the PRISMA 2020 for Abstracts checklist (table 2). | PY | PY | - | PY |
| **Introduction** | | | | | | |
| Rationale | 3 | Describe the rationale for the review in the context of existing knowledge | Y | Y | - | Y |
| Objectives | 4 | Provide an explicit statement of the objective(s) or question(s) the review addresses. | Y | Y | - | Y |
| **Methods** | | | | | | |
| Eligibility criteria | 5 | Specify the inclusion and exclusion criteria for the review and how studies were grouped for the syntheses. | Y | Y | - | Y |
| Information sources | 6 | Specify all databases, registers, websites, organisations, reference lists and other sources searched or consulted to identify studies. Specify the date when each source was last searched or consulted. | PY | Y | Y | Y |
| Search strategy | 7 | Present the full search strategies for all databases, registers and websites, including any filters and limits used | N | Y | PY | PY |
| Selection process | 8 | Specify the methods used to decide whether a study met the inclusion criteria of the review, including how many reviewers screened each record and each report retrieved, whether they worked independently, and if applicable, details of automation tools  used in the process. | Y | Y | - | Y |
| Data collection  process | 9 | Specify the methods used to collect data from reports, including how many reviewers collected data from each report, whether they worked independently, any processes for obtaining or confirming data from study investigators, and if applicable, details of automation tools used in the process. | Y | Y | - | Y |
| Data items | 10a | List and define all outcomes for which data were sought. Specify whether all results that were compatible with each outcome domain in each study were sought (e.g. for all measures, time points, analyses), and if not, the methods used to decide which  results to collect. | Y | Y | - | Y |
|  | 10b | List and define all other variables for which data were sought (e.g. participant and intervention characteristics, funding sources). Describe any assumptions made about any missing or unclear information. | Y | Y | - | Y |
| Study risk of bias  assessment | 11 | Specify the methods used to assess risk of bias in the included studies, including details of the tool(s) used, how many reviewers assessed each study and whether they worked independently, and if applicable, details of automation tools used in the process. | Y | Y | - | Y |
| Effect measures | 12 | Specify for each outcome the effect measure(s) (e.g. risk ratio, mean difference) used in the synthesis or presentation of results. | Y | Y | - | Y |
| Synthesis methods | 13a | Describe the processes used to decide which studies were eligible for each synthesis (e.g. tabulating the study intervention characteristics and comparing against the planned groups for each synthesis (item #5)). | Y | Y | - | Y |
|  | 13b | Describe any methods required to prepare the data for presentation or synthesis, such as handling of missing summary statistics, or data conversions. | Y | Y | - | Y |
|  | 13c | Describe any methods used to tabulate or visually display results of individual studies and syntheses. | Y | Y | - | Y |
|  | 13d | Describe any methods used to synthesise results and provide a rationale for the choice(s). If meta-analysis was performed, describe the model(s), method(s) to identify the presence and extent of statistical heterogeneity, and software package(s) used. | Y | Y | - | Y |
|  | 13e | Describe any methods used to explore possible causes of heterogeneity among study results (e.g. subgroup analysis, meta regression). | Y | Y | - | Y |
|  | 13f | Describe any sensitivity analyses conducted to assess robustness of the synthesised results. | Y | Y | - | Y |
| Reporting bias  assessment | 14 | Describe any methods used to assess risk of bias due to missing results in a synthesis (arising from reporting biases). | N | PY | PY | PY |
| Certainty assessment | 15 | Describe any methods used to assess certainty (or confidence) in the body of evidence for an outcome. | N | PY | PY | PY |
| **Results** | | | | | | |
| Study selection | 16a | Describe the results of the search and selection process, from the number of records identified in the search to the number of studies included in the review, ideally using a flow diagram (see fig 1). | Y | Y | - | Y |
|  | 16b | Cite studies that might appear to meet the inclusion criteria, but which were excluded, and explain why they were excluded. | Y | Y | - | Y |
| Study characteristics | 17 | Cite each included study and present its characteristics. | Y | Y | - | Y |
| Risk of bias in studies | 18 | Present assessments of risk of bias for each included study. | Y | Y | - | Y |
| Results of individual  studies | 19 | For all outcomes, present, for each study: (a) summary statistics for each group (where appropriate) and (b) an effect estimate and its precision (e.g. confidence/credible interval), ideally using structured tables or plots. | Y | Y | - | Y |
| Results of syntheses | 20a | For each synthesis, briefly summarise the characteristics and risk of bias among contributing studies. | Y | Y | - | Y |
|  | 20b | Present results of all statistical syntheses conducted. If meta-analysis was done, present for each the summary estimate and its precision (e.g. confidence/credible interval) and measures of statistical heterogeneity. If comparing groups, describe the direction of the effect. | Y | Y | - | Y |
|  | 20c | Present results of all investigations of possible causes of heterogeneity among study results. | Y | Y | - | Y |
|  | 20d | Present results of all sensitivity analyses conducted to assess the robustness of the synthesised results. | Y | Y | - | Y |
| Reporting biases | 21 | Present assessments of risk of bias due to missing results (arising from reporting biases) for each synthesis assessed. | N | PY | PY | PY |
| Certainty of evidence | 22 | Present assessments of certainty (or confidence) in the body of evidence for each outcome assessed. | N | PY | PY | PY |
| **Discussion** | | | | | | |
| Discussion | 23a | Provide a general interpretation of the results in the context of other evidence. | Y | Y | - | Y |
|  | 23b | Discuss any limitations of the evidence included in the review. | Y | Y | - | Y |
|  | 23c | Discuss any limitations of the review processes used. | Y | Y | - | Y |
|  | 23d | Discuss implications of the results for practice, policy, and future research. | Y | Y | - | Y |
| **Other information** | | | | | | |
| Registration and  protocol | 24a | Provide registration information for the review, including register name and registration number, or state that the review was not registered. | N | N | - | N |
|  | 24b | Indicate where the review protocol can be accessed, or state that a protocol was not prepared. | N | N | - | N |
|  | 24c | Describe and explain any amendments to information provided at registration or in the protocol. | N | N | - | N |
| Support | 25 | Describe sources of financial or non-financial support for the review, and the role of the funders or sponsors in the review. | N | N | - | N |
| Competing interests | 26 | Declare any competing interests of review authors. | N | N | - | N |
| Availability of data,  code, and other  materials | 27 | Report which of the following are publicly available and where they can be found: template data collection forms; data extracted from included studies; data used for all analyses; analytic code; any other materials used in the review. | N | N | - | N |

19.刘香.奥马珠单抗治疗儿童中重度过敏性哮喘有效性及安全性Meta分析[D].南昌大学,2024.DOI:10.27232/d.cnki.gnchu.2024.004079.

| Section and topic | Item # | Checklist item | A | B | C | D |
| --- | --- | --- | --- | --- | --- | --- |
| **Title** | | | | | | |
| Title | 1 | Identify the report as a systematic review. | Y | Y | - | Y |
| **Abstract** | | | | | | |
| Abstract | 2 | See the PRISMA 2020 for Abstracts checklist (table 2). | PY | PY | - | PY |
| **Introduction** | | | | | | |
| Rationale | 3 | Describe the rationale for the review in the context of existing knowledge | Y | Y | - | Y |
| Objectives | 4 | Provide an explicit statement of the objective(s) or question(s) the review addresses. | Y | Y | - | Y |
| **Methods** | | | | | | |
| Eligibility criteria | 5 | Specify the inclusion and exclusion criteria for the review and how studies were grouped for the syntheses. | Y | Y | - | Y |
| Information sources | 6 | Specify all databases, registers, websites, organisations, reference lists and other sources searched or consulted to identify studies. Specify the date when each source was last searched or consulted. | Y | Y | - | Y |
| Search strategy | 7 | Present the full search strategies for all databases, registers and websites, including any filters and limits used | Y | Y | - | Y |
| Selection process | 8 | Specify the methods used to decide whether a study met the inclusion criteria of the review, including how many reviewers screened each record and each report retrieved, whether they worked independently, and if applicable, details of automation tools  used in the process. | Y | Y | - | Y |
| Data collection  process | 9 | Specify the methods used to collect data from reports, including how many reviewers collected data from each report, whether they worked independently, any processes for obtaining or confirming data from study investigators, and if applicable, details of automation tools used in the process. | Y | Y | - | Y |
| Data items | 10a | List and define all outcomes for which data were sought. Specify whether all results that were compatible with each outcome domain in each study were sought (e.g. for all measures, time points, analyses), and if not, the methods used to decide which  results to collect. | Y | Y | - | Y |
|  | 10b | List and define all other variables for which data were sought (e.g. participant and intervention characteristics, funding sources). Describe any assumptions made about any missing or unclear information. | Y | Y | - | Y |
| Study risk of bias  assessment | 11 | Specify the methods used to assess risk of bias in the included studies, including details of the tool(s) used, how many reviewers assessed each study and whether they worked independently, and if applicable, details of automation tools used in the process. | Y | Y | - | Y |
| Effect measures | 12 | Specify for each outcome the effect measure(s) (e.g. risk ratio, mean difference) used in the synthesis or presentation of results. | Y | Y | - | Y |
| Synthesis methods | 13a | Describe the processes used to decide which studies were eligible for each synthesis (e.g. tabulating the study intervention characteristics and comparing against the planned groups for each synthesis (item #5)). | Y | Y | - | Y |
|  | 13b | Describe any methods required to prepare the data for presentation or synthesis, such as handling of missing summary statistics, or data conversions. | Y | Y | - | Y |
|  | 13c | Describe any methods used to tabulate or visually display results of individual studies and syntheses. | Y | Y | - | Y |
|  | 13d | Describe any methods used to synthesise results and provide a rationale for the choice(s). If meta-analysis was performed, describe the model(s), method(s) to identify the presence and extent of statistical heterogeneity, and software package(s) used. | Y | Y | - | Y |
|  | 13e | Describe any methods used to explore possible causes of heterogeneity among study results (e.g. subgroup analysis, meta regression). | Y | Y | - | Y |
|  | 13f | Describe any sensitivity analyses conducted to assess robustness of the synthesised results. | Y | Y | - | Y |
| Reporting bias  assessment | 14 | Describe any methods used to assess risk of bias due to missing results in a synthesis (arising from reporting biases). | N | PY | PY | PY |
| Certainty assessment | 15 | Describe any methods used to assess certainty (or confidence) in the body of evidence for an outcome. | N | N | - | N |
| **Results** | | | | | | |
| Study selection | 16a | Describe the results of the search and selection process, from the number of records identified in the search to the number of studies included in the review, ideally using a flow diagram (see fig 1). | Y | Y | - | Y |
|  | 16b | Cite studies that might appear to meet the inclusion criteria, but which were excluded, and explain why they were excluded. | PY | PY | - | PY |
| Study characteristics | 17 | Cite each included study and present its characteristics. | Y | Y | - | Y |
| Risk of bias in studies | 18 | Present assessments of risk of bias for each included study. | Y | Y | - | Y |
| Results of individual  studies | 19 | For all outcomes, present, for each study: (a) summary statistics for each group (where appropriate) and (b) an effect estimate and its precision (e.g. confidence/credible interval), ideally using structured tables or plots. | Y | Y | - | Y |
| Results of syntheses | 20a | For each synthesis, briefly summarise the characteristics and risk of bias among contributing studies. | Y | Y | - | Y |
|  | 20b | Present results of all statistical syntheses conducted. If meta-analysis was done, present for each the summary estimate and its precision (e.g. confidence/credible interval) and measures of statistical heterogeneity. If comparing groups, describe the direction of the effect. | Y | Y | - | Y |
|  | 20c | Present results of all investigations of possible causes of heterogeneity among study results. | Y | Y | - | Y |
|  | 20d | Present results of all sensitivity analyses conducted to assess the robustness of the synthesised results. | Y | Y | - | Y |
| Reporting biases | 21 | Present assessments of risk of bias due to missing results (arising from reporting biases) for each synthesis assessed. | PY | PY | - | PY |
| Certainty of evidence | 22 | Present assessments of certainty (or confidence) in the body of evidence for each outcome assessed. | PY | N | PY | PY |
| **Discussion** | | | | | | |
| Discussion | 23a | Provide a general interpretation of the results in the context of other evidence. | Y | Y | - | Y |
|  | 23b | Discuss any limitations of the evidence included in the review. | Y | Y | - | Y |
|  | 23c | Discuss any limitations of the review processes used. | Y | Y | - | Y |
|  | 23d | Discuss implications of the results for practice, policy, and future research. | Y | Y | - | Y |
| **Other information** | | | | | | |
| Registration and  protocol | 24a | Provide registration information for the review, including register name and registration number, or state that the review was not registered. | N | PY | N | N |
|  | 24b | Indicate where the review protocol can be accessed, or state that a protocol was not prepared. | N | N | - | N |
|  | 24c | Describe and explain any amendments to information provided at registration or in the protocol. | N | N | - | N |
| Support | 25 | Describe sources of financial or non-financial support for the review, and the role of the funders or sponsors in the review. | N | PY | Y | Y |
| Competing interests | 26 | Declare any competing interests of review authors. | N | N | - | N |
| Availability of data,  code, and other  materials | 27 | Report which of the following are publicly available and where they can be found: template data collection forms; data extracted from included studies; data used for all analyses; analytic code; any other materials used in the review. | N | Y | N | N |

**GRADE**

①Methodological quality of included studies was low, with biases in randomization, allocation concealment, and blinding. ②The heterogeneity was large and low confidence interval overlap. ③The population was not broadly representative. ④Small sample size, 95% confidence intervals include null values. ⑤Few studies were included, the funnel plot was not symmetrical, Egger’s test found that publication bias or results were positive, and there was no publication bias evaluation.

A:The conclusions of researcher Ph.D. Zhuang Wang.

B:The conclusions of researcher Master Dongze Li.

C:In case of a difference of opinions, it shall be adjudicated by Professor Yongji Wang.

D:Conclusive conclusion.

1.麦琳,孙蓉菲,李为民.奥马佐单抗治疗过敏性支气管哮喘疗效与安全性的系统评价[J].中国循证医学杂志,2013,13(06):709-716.

| Endpoint measure | Downgrading factor | A | B | C | D |
| --- | --- | --- | --- | --- | --- |
| Acute exacerbation rate of asthma | Risk of bias | 0 | 0 | - | 0 |
|  | Inconsistency | 0 | 0 | - | 0 |
|  | Indirectness | 0 | 0 | - | 0 |
|  | Impression | 0 | 0 | - | 0 |
|  | Publication bias | 0 | 0 | - | 0 |
| GETE excellent/ good rate | Risk of bias | 0 | 0 | - | 0 |
|  | Inconsistency | -1^②^ | -1^②^ | ^-^ | -1^②^ |
|  | Indirectness | 0 | 0 | - | 0 |
|  | Impression | 0 | 0 | - | 0 |
|  | Publication bias | 0 | 0 | - | 0 |
| ICS reduction | Risk of bias | 0 | 0 | - | 0 |
|  | Inconsistency | 0 | 0 | - | 0 |
|  | Indirectness | 0 | 0 | - | 0 |
|  | Impression | 0 | 0 | - | 0 |
|  | Publication bias | 0 | 0 | - | 0 |
| Reduction of emergency drugs | Risk of bias | 0 | -1^①^ | 0 | 0 |
|  | Inconsistency | 0 | 0 | - | 0 |
|  | Indirectness | 0 | 0 | 0 | 0 |
|  | Impression | -1^④^ | 0 | - | 0 |
|  | Publication bias | 0 | 0 | - | 0 |
| Improvement of lung function | Risk of bias | 0 | -1^①^ | 0 | 0 |
|  | Inconsistency | 0 | 0 | - | 0 |
|  | Indirectness | -1^③^ | 0 | 0 | 0 |
|  | Impression | 0 | 0 | - | 0 |
|  | Publication bias | 0 | 0 | - | 0 |
| AQLQ score(Total score improvement ≥ 0.5 points) | Risk of bias | 0 | -1^①^ | 0 | 0 |
|  | Inconsistency | -1^②^ | 0 | 0 | 0 |
|  | Indirectness | 0 | 0 | - | 0 |
|  | Impression | 0 | -1^④^ | 0 | 0 |
|  | Publication bias | 0 | 0 | - | 0 |
| AQLQ score(Total score improvement ≥ 1.5 points) | Risk of bias | 0 | 0 | - | 0 |
|  | Inconsistency | -1^②^ | -1^②^ | - | -1^②^ |
|  | Indirectness | 0 | 0 | - | 0 |
|  | Impression | 0 | -1^④^ | 0 | 0 |
|  | Publication bias | 0 | 0 | - | 0 |
| Overall adverse reaction rate | Risk of bias | 0 | 0 | - | 0 |
|  | Inconsistency | 0 | 0 | - | 0 |
|  | Indirectness | 0 | 0 | - | 0 |
|  | Impression | 0 | 0 | - | 0 |
|  | Publication bias | 0 | 0 | - | 0 |
| Severe adverse reaction rate | Risk of bias | 0 | 0 | - | 0 |
|  | Inconsistency | 0 | 0 | - | 0 |
|  | Indirectness | 0 | 0 | - | 0 |
|  | Impression | 0 | 0 | - | 0 |
|  | Publication bias | 0 | 0 | - | 0 |

2.刘长智,郑晓滨,刘茂,等.奥马珠单抗治疗难治性哮喘疗效与安全性的Meta分析[J].中华临床医师杂志(电子版),2013,7(14):6519-6525.

| Endpoint measure | Downgrading factor | A | B | C | D |
| --- | --- | --- | --- | --- | --- |
| Number of asthma exacerbations(stable steroid phase) | Risk of bias | 0 | 0 | - | 0 |
|  | Inconsistency | -1^②^ | -1^②^ | - | -1^②^ |
|  | Indirectness | 0 | 0 | - | 0 |
|  | Impression | 0 | 0 | - | 0 |
|  | Publication bias | 0 | 0 | - | 0 |
| Number of asthma exacerbations(steroid reduction phase) | Risk of bias | 0 | 0 | - | 0 |
|  | Inconsistency | 0 | 0 | - | -0 |
|  | Indirectness | 0 | 0 | - | 0 |
|  | Impression | 0 | 0 | - | 0 |
|  | Publication bias | 0 | 0 | - | 0 |
| Number of patients with complete withdrawal of inhaled corticosteroids (ICS) | Risk of bias | 0 | 0 | - | 0 |
|  | Inconsistency | -1^②^ | -1^②^ | - | -1^②^ |
|  | Indirectness | 0 | 0 | - | 0 |
|  | Impression | 0 | 0 | - | 0 |
|  | Publication bias | 0 | 0 | - | 0 |
| Number of patients with ICS reduction >50% | Risk of bias | 0 | 0 | - | 0 |
|  | Inconsistency | 0 | 0 | - | 0 |
|  | Indirectness | 0 | 0 | - | 0 |
|  | Impression | 0 | 0 | - | 0 |
|  | Publication bias | 0 | 0 | - | 0 |
| Number of patients with global evaluation of therapy effective (GETE) rated as "excellent" or "good" | Risk of bias | 0 | 0 | - | 0 |
|  | Inconsistency | 0 | 0 | - | 0 |
|  | Indirectness | 0 | 0 | - | 0 |
|  | Impression | 0 | 0 | - | 0 |
|  | Publication bias | 0 | 0 | - | 0 |
| Dosage of reliever medication (salbutamol) | Risk of bias | 0 | 0 | - | 0 |
|  | Inconsistency | 0 | 0 | - | 0 |
|  | Indirectness | 0 | 0 | - | 0 |
|  | Impression | 0 | 0 | - | 0 |
|  | Publication bias | 0 | 0 | - | 0 |
| Asthma symptom score | Risk of bias | 0 | 0 | - | 0 |
|  | Inconsistency | 0 | 0 | - | 0 |
|  | Indirectness | 0 | 0 | - | 0 |
|  | Impression | 0 | 0 | - | 0 |
|  | Publication bias | 0 | 0 | - | 0 |
| Peak expiratory flow (PEF) | Risk of bias | -1^①^ | 0 | -1^①^ | -1^①^ |
|  | Inconsistency | 0 | 0 | - | 0 |
|  | Indirectness | 0 | 0 | - | 0 |
|  | Impression | -1^④^ | -1^④^ | - | -1^④^ |
|  | Publication bias | 0 | 0 | - | 0 |
| Forced expiratory volume in the first second (FEV1) | Risk of bias | -1^①^ | 0 | -1^①^ | -1^①^ |
|  | Inconsistency | 0 | 0 | - | 0 |
|  | Indirectness | 0 | 0 | - | 0 |
|  | Impression | -1^④^ | -1^④^ | - | -1^④^ |
|  | Publication bias | 0 | 0 | - | 0 |
| Total adverse reactions | Risk of bias | 0 | 0 | - | 0 |
|  | Inconsistency | 0 | 0 | - | 0 |
|  | Indirectness | 0 | 0 | - | 0 |
|  | Impression | 0 | -1^④^ | 0 | 0 |
|  | Publication bias | 0 | 0 | - | 0 |

3.Lai T, Wang S, Xu Z, Zhang C, Zhao Y, Hu Y, Cao C, Ying S, Chen Z, Li W, Wu B, Shen H. Long-term efficacy and safety of omalizumab in patients with persistent uncontrolled allergic asthma: a systematic review and meta-analysis. Sci Rep. 2015 Feb 3;5:8191. doi: 10.1038/srep08191. Erratum in: Sci Rep. 2015 Aug 14;5:9548. doi: 10.1038/srep09548. PMID: 25645133; PMCID: PMC4314644.

| Endpoint measure | Downgrading factor | A | B | C | D |
| --- | --- | --- | --- | --- | --- |
| Rate of asthma exacerbations | Risk of bias | -1^①^ | -1^①^ | - | -1^①^ |
|  | Inconsistency | 0 | 0 | - | 0 |
|  | Indirectness | 0 | 0 | - | 0 |
|  | Impression | 0 | 0 | - | 0 |
|  | Publication bias | 0 | 0 | - | 0 |
| Complete withdrawal from inhaled corticosteroid therapy | Risk of bias | 0 | 0 | - | 0 |
|  | Inconsistency | -1^①^ | -1^①^ | - | -1^①^ |
|  | Indirectness | 0 | 0 | - | 0 |
|  | Impression | 0 | 0 | - | 0 |
|  | Publication bias | 0 | 0 | - | 0 |
| Global Evaluation of Treatment Effectiveness（GETE） | Risk of bias | -1^①^ | -1^①^ | - | -1^①^ |
|  | Inconsistency | 0 | 0 | - | 0 |
|  | Indirectness | 0 | 0 | - | 0 |
|  | Impression | 0 | 0 | - | 0 |
|  | Publication bias | 0 | 0 | - | 0 |
| Asthma Quality of Life Questionnaire（AQLQ）score | Risk of bias | -1^①^ | -1^①^ | - | -1^①^ |
|  | Inconsistency | 0 | 0 | - | 0 |
|  | Indirectness | 0 | 0 | - | 0 |
|  | Impression | 0 | 0 | - | 0 |
|  | Publication bias | 0 | 0 | - | 0 |
| Adverse events | Risk of bias | 0 | 0 | - | 0 |
|  | Inconsistency | 0 | 0 | - | 0 |
|  | Indirectness | 0 | 0 | - | 0 |
|  | Impression | 0 | 0 | - | 0 |
|  | Publication bias | 0 | 0 | - | 0 |

4.Rodrigo GJ, Neffen H. Systematic review on the use of omalizumab for the treatment of asthmatic children and adolescents. Pediatr Allergy Immunol. 2015 Sep;26(6):551-6. doi: 10.1111/pai.12405. Epub 2015 Jul 1. PMID: 25963882.

| Endpoint measure | Downgrading factor | A | B | C | D |
| --- | --- | --- | --- | --- | --- |
| Asthma exacerbations | Risk of bias | -1^①^ | -1^①^ | - | -1^①^ |
|  | Inconsistency | 0 | 0 | - | 0 |
|  | Indirectness | 0 | 0 | - | 0 |
|  | Impression | 0 | 0 | - | 0 |
|  | Publication bias | 0 | 0 | - | 0 |
| Asthma symptom score | Risk of bias | -1^①^ | 0 | - | -1^①^ |
|  | Inconsistency | 0 | 0 | - | 0 |
|  | Indirectness | 0 | 0 | - | 0 |
|  | Impression | 0 | 0 | - | 0 |
|  | Publication bias | -1^⑤^ | 0 | -1^⑤^ | -1^⑤^ |
| Pulmonary function | Risk of bias | 0 | -1^①^ | -1^①^ | -1^①^ |
|  | Inconsistency | 0 | 0 | - | 0 |
|  | Indirectness | 0 | 0 | - | 0 |
|  | Impression | 0 | -1^④^ | 0 | 0 |
|  | Publication bias | -1^⑤^ | 0 | -1^⑤^ | -1^⑤^ |
| Rescue medication use | Risk of bias | -1^①^ | -1^①^ | - | -1^①^ |
|  | Inconsistency | 0 | 0 | - | 0 |
|  | Indirectness | 0 | 0 | - | 0 |
|  | Impression | 0 | -1^④^ | 0 | 0 |
|  | Publication bias | 0 | 0 | - | 0 |
| Health-related quality of life | Risk of bias | -1^①^ | 0 | 0 | 0 |
|  | Inconsistency | 0 | 0 | - | 0 |
|  | Indirectness | 0 | 0 | - | 0 |
|  | Impression | 0 | 0 | - | 0 |
|  | Publication bias | 0 | 0 | - | 0 |
| Adverse events | Risk of bias | 0 | -1^①^ | -1^①^ | -1^①^ |
|  | Inconsistency | 0 | 0 | - | 0 |
|  | Indirectness | 0 | 0 | - | 0 |
|  | Impression | 0 | 0 | - | 0 |
|  | Publication bias | 0 | -1^⑤^ | 0 | 0 |

5.苗伟伟,汪凤凤,陈子,等.奥马珠单抗治疗难治性哮喘疗效的Meta分析[J].中国呼吸与危重监护杂志,2015,14(05):449-455.

| Endpoint measure | Downgrading factor | A | B | C | D |
| --- | --- | --- | --- | --- | --- |
| Rate of exacerbation（Stable-steroid phase） | Risk of bias | -1^①^ | 0 | -1^①^ | -1^①^ |
|  | Inconsistency | -1^②^ | -1^②^ | - | -1^②^ |
|  | Indirectness | 0 | 0 | - | 0 |
|  | Impression | 0 | 0 | - | 0 |
|  | Publication bias | 0 | 0 | - | 0 |
| Rate of exacerbation（Steroid-reduction phase） | Risk of bias | 0 | 0 | - | 0 |
|  | Inconsistency | 0 | 0 | - | 0 |
|  | Indirectness | 0 | 0 | - | 0 |
|  | Impression | 0 | 0 | - | 0 |
|  | Publication bias | 0 | 0 | - | 0 |
| Dosage of glucocorticoid | Risk of bias | -1^①^ | 0 | 0 | 0 |
|  | Inconsistency | -1^②^ | -1^②^ | - | -1^②^ |
|  | Indirectness | 0 | 0 | - | 0 |
|  | Impression | 0 | 0 | - | 0 |
|  | Publication bias | 0 | 0 | - | 0 |
| Rate of emergency visits | Risk of bias | 0 | 0 | - | 0 |
|  | Inconsistency | -1^②^ | -1^②^ | - | -1^②^ |
|  | Indirectness | 0 | 0 | - | 0 |
|  | Impression | -1^④^ | 0 | 0 | 0 |
|  | Publication bias | 0 | 0 | - | 0 |
| Asthma Quality of Life Questionnaire score | Risk of bias | 0 | 0 | - | 0 |
|  | Inconsistency | 0 | 0 | - | 0 |
|  | Indirectness | 0 | 0 | - | 0 |
|  | Impression | 0 | 0 | - | 0 |
|  | Publication bias | 0 | 0 | - | 0 |

6.季春梅,孟玲. 奥马珠单抗长程治疗与短程治疗过敏性哮喘的有效性及安全性比较的meta分析[C]//2016年江苏省药学大会暨第十六届江苏省药师周 论文集. 2016:105-112.

| Endpoint measure | Downgrading factor | A | B | C | D |
| --- | --- | --- | --- | --- | --- |
| Asthma Exacerbation | Risk of bias | 0 | 0 | - | 0 |
|  | Inconsistency | -1^②^ | -1^②^ | - | -1^②^ |
|  | Indirectness | 0 | 0 | - | 0 |
|  | Impression | 0 | 0 | - | 0 |
|  | Publication bias | 0 | 0 | - | 0 |
| Reduction in Medication | Risk of bias | 0 | 0 | - | 0 |
|  | Inconsistency | -1^②^ | -1^②^ | - | -1^②^ |
|  | Indirectness | 0 | 0 | - | 0 |
|  | Impression | 0 | 0 | - | 0 |
|  | Publication bias | 0 | 0 | - | 0 |
| Adverse Events | Risk of bias | 0 | 0 | - | 0 |
|  | Inconsistency | 0 | 0 | - | 0 |
|  | Indirectness | 0 | 0 | - | 0 |
|  | Impression | 0 | 0 | - | 0 |
|  | Publication bias | 0 | 0 | - | 0 |
| Serious Adverse Events | Risk of bias | 0 | 0 | - | 0 |
|  | Inconsistency | -1^②^ | 0 | 0 | 0 |
|  | Indirectness | 0 | 0 | - | 0 |
|  | Impression | 0 | 0 | - | 0 |
|  | Publication bias | 0 | 0 | - | 0 |

8.牟姗,张薇,江德鹏.奥马珠单抗治疗难治性变应性哮喘效果的Meta分析[J].中国医药导报,2019,16(10):75-79+99.

| Endpoint measure | Downgrading factor | A | B | C | D |
| --- | --- | --- | --- | --- | --- |
| severe exacerbations | Risk of bias | 0 | 0 | - | 0 |
|  | Inconsistency | 0 | 0 | - | 0 |
|  | Indirectness | 0 | 0 | - | 0 |
|  | Impression | 0 | 0 | - | 0 |
|  | Publication bias | 0 | 0 | - | 0 |
| Proportion of patients with IGETE rated as "excellent" or "good"(Stable Steroid Dose Phase) | Risk of bias | 0 | 0 | - | 0 |
|  | Inconsistency | 0 | 0 | - | 0 |
|  | Indirectness | 0 | 0 | - | 0 |
|  | Impression | 0 | 0 | - | 0 |
|  | Publication bias | 0 | 0 | - | 0 |
| Proportion of patients with IGETE rated as "excellent" or "good"(Steroid Dose Reduction Phase) | Risk of bias | 0 | 0 | - | 0 |
|  | Inconsistency | 0 | 0 | - | 0 |
|  | Indirectness | 0 | 0 | - | 0 |
|  | Impression | 0 | 0 | - | 0 |
|  | Publication bias | 0 | 0 | - | 0 |
| Proportion of patients with AQLQ score ≥1.5(Stable Steroid Dose Phase) | Risk of bias | 0 | 0 | - | 0 |
|  | Inconsistency | 0 | 0 | - | 0 |
|  | Indirectness | 0 | 0 | - | 0 |
|  | Impression | 0 | 0 | - | 0 |
|  | Publication bias | 0 | 0 | - | 0 |
| Proportion of patients with AQLQ score ≥1.5(Steroid Dose Reduction Phase) | Risk of bias | 0 | 0 | - | 0 |
|  | Inconsistency | 0 | 0 | - | 0 |
|  | Indirectness | 0 | 0 | - | 0 |
|  | Impression | 0 | 0 | - | 0 |
|  | Publication bias | 0 | 0 | - | 0 |

9.Henriksen DP, Bodtger U, Sidenius K, Maltbaek N, Pedersen L, Madsen H, Andersson EA, Norgaard O, Madsen LK, Chawes BL. Efficacy of omalizumab in children, adolescents, and adults with severe allergic asthma: a systematic review, meta-analysis, and call for new trials using current guidelines for assessment of severe asthma. Allergy Asthma Clin Immunol. 2020 Jun 18;16:49. doi: 10.1186/s13223-020-00442-0. PMID: 32565844; PMCID: PMC7302157.

| Endpoint measure | Downgrading factor | A | B | C | D |
| --- | --- | --- | --- | --- | --- |
| Exacerbation rate | Risk of bias | -1^①^ | -1^①^ | - | -1^①^ |
|  | Inconsistency | -1^②^ | -1^②^ | - | -1^②^ |
|  | Indirectness | -1^③^ | -1^③^ | - | -1^③^ |
|  | Impression | -1^④^ | -1^④^ | - | -1^④^ |
|  | Publication bias | -1^⑤^ | 0 | 0 | 0 |
| Oral corticosteroid-maintenance treatment | Risk of bias | -1^①^ | -1^①^ | - | -1^①^ |
|  | Inconsistency | -1^②^ | 0 | - | -1^②^ |
|  | Indirectness | -1^③^ | -1^③^ | - | -1^③^ |
|  | Impression | -1^④^ | -1^④^ | - | -1^④^ |
|  | Publication bias | -1^⑤^ | 0 | -1^⑤^ | -1^⑤^ |
| Lung function（FEV1） | Risk of bias | -1^①^ | -1^①^ | - | -1^①^ |
|  | Inconsistency | -1^②^ | -1^②^ | - | -1^②^ |
|  | Indirectness | -1^③^ | -1^③^ | - | -1^③^ |
|  | Impression | -1^④^ | -1^④^ | - | -1^④^ |
|  | Publication bias | -1^⑤^ | 0 | 0 | 0 |
| Asthma control | Risk of bias | -1^①^ | -1^①^ | - | -1^①^ |
|  | Inconsistency | -1^②^ | -1^②^ | - | -1^②^ |
|  | Indirectness | -1^③^ | -1^③^ | - | -1^③^ |
|  | Impression | -1^④^ | -1^④^ | - | -1^④^ |
|  | Publication bias | -1^⑤^ | 0 | -1^⑤^ | -1^⑤^ |
| Quality of life（QoL） | Risk of bias | -1^①^ | -1^①^ | - | -1^①^ |
|  | Inconsistency | -1^②^ | -1^②^ | - | -1^②^ |
|  | Indirectness | -1^③^ | -1^③^ | - | -1^③^ |
|  | Impression | -1^④^ | -1^④^ | - | -1^④^ |
|  | Publication bias | -1^⑤^ | 0 | -1^⑤^ | -1^⑤^ |
| Serious adverse events（SAEs） | Risk of bias | -1^①^ | -1^①^ | - | -1^①^ |
|  | Inconsistency | 0 | 0 | - | 0 |
|  | Indirectness | -1^③^ | -1^③^ | - | -1^③^ |
|  | Impression | -1^④^ | -1^④^ | - | -1^④^ |
|  | Publication bias | -1^⑤^ | 0 | 0 | 0 |
| Drop-out rate | Risk of bias | -1^①^ | -1^①^ | - | -1^①^ |
|  | Inconsistency | -1^②^ | -1^②^ | - | -1^②^ |
|  | Indirectness | -1^③^ | -1^③^ | - | -1^③^ |
|  | Impression | -1^④^ | -1^④^ | - | -1^④^ |
|  | Publication bias | -1^⑤^ | 0 | 0 | 0 |
| Sick leave | Risk of bias | -1^①^ | -1^①^ | - | -1^①^ |
|  | Inconsistency | -1^②^ | 0 | -1^②^ | -1^②^ |
|  | Indirectness | -1^③^ | -1^③^ | - | -1^③^ |
|  | Impression | -1^④^ | -1^④^ | - | -1^④^ |
|  | Publication bias | -1^⑤^ | 0 | 0 | 0 |

10.Fu Z, Xu Y, Cai C. Efficacy and safety of omalizumab in children with moderate-to-severe asthma: a meta-analysis. J Asthma. 2021 Oct;58(10):1350-1358. doi: 10.1080/02770903.2020.1789875. Epub 2020 Jul 16. PMID: 32602383.

| Endpoint measure | Downgrading factor | A | B | C | D |
| --- | --- | --- | --- | --- | --- |
| Asthma exacerbations rate | Risk of bias | -1^①^ | -1^①^ | - | -1^①^ |
|  | Inconsistency | 0 | 0 | - | 0 |
|  | Indirectness | 0 | 0 | - | 0 |
|  | Impression | 0 | 0 | - | 0 |
|  | Publication bias | 0 | 0 | - | 0 |
| Global evaluation of treatment effectiveness | Risk of bias | -1^①^ | -1^①^ | - | -1^①^ |
|  | Inconsistency | -1^②^ | 0 | 0 | 0 |
|  | Indirectness | 0 | 0 | - | 0 |
|  | Impression | 0 | 0 | - | 0 |
|  | Publication bias | 0 | 0 | - | 0 |
| Decrease in inhaled corticosteroid (ICS) dose | Risk of bias | -1^①^ | -1^①^ | - | -1^①^ |
|  | Inconsistency | 0 | 0 | - | 0 |
|  | Indirectness | 0 | 0 | - | 0 |
|  | Impression | 0 | 0 | - | 0 |
|  | Publication bias | 0 | 0 | - | 0 |
| Incidence of severe adverse events | Risk of bias | -1^①^ | -1^①^ | - | -1^①^ |
|  | Inconsistency | 0 | 0 | - | 0 |
|  | Indirectness | 0 | 0 | - | 0 |
|  | Impression | 0 | 0 | - | 0 |
|  | Publication bias | 0 | 0 | - | 0 |

11.姜红玉,李娟,胡文凤,等.奥马珠单抗治疗儿童中重度过敏性哮喘的有效性和安全性系统评价[J].中国医院用药评价与分析,2021,21(09):1091-1096.DOI:10.14009/j.issn.1672-2124.2021.09.017.

| Endpoint measure | Downgrading factor | A | B | C | D |
| --- | --- | --- | --- | --- | --- |
| Exacerbation incidence of asthma | Risk of bias | 0 | -1^①^ | -1^①^ | -1^①^ |
|  | Inconsistency | 0 | 0 | - | 0 |
|  | Indirectness | 0 | 0 | - | 0 |
|  | Impression | 0 | -1^④^ | 0 | 0 |
|  | Publication bias | 0 | -1^⑤^ | -1^⑤^ | -1^⑤^ |
| GETE excellent rate | Risk of bias | 0 | -1^①^ | -1^①^ | -1^①^ |
|  | Inconsistency | 0 | 0 | - | 0 |
|  | Indirectness | 0 | 0 | - | 0 |
|  | Impression | 0 | -1^④^ | 0 | 0 |
|  | Publication bias | -1^⑤^ | -1^⑤^ | - | -1^⑤^ |
| C-ACT score | Risk of bias | 0 | -1^①^ | -1^①^ | -1^①^ |
|  | Inconsistency | 0 | 0 | - | 0 |
|  | Indirectness | 0 | 0 | - | 0 |
|  | Impression | 0 | 0 | - | 0 |
|  | Publication bias | -1^⑤^ | -1^⑤^ | - | -1^⑤^ |
| Forced Expiratory Volume in 1 second to predicted value, FEV1% | Risk of bias | 0 | -1^①^ | -1^①^ | -1^①^ |
|  | Inconsistency | 0 | 0 | - | 0 |
|  | Indirectness | 0 | 0 | - | 0 |
|  | Impression | 0 | -1^④^ | 0 | 0 |
|  | Publication bias | -1^⑤^ | -1^⑤^ | - | -1^⑤^ |
| Forced Expiratory Volume in 1 second to Forced Vital Capacity, FEV1/FVC | Risk of bias | 0 | -1^①^ | -1^①^ | -1^①^ |
|  | Inconsistency | 0 | 0 | - | 0 |
|  | Indirectness | 0 | 0 | - | 0 |
|  | Impression | -1^④^ | -1^④^ | - | -1^④^ |
|  | Publication bias | -1^⑤^ | -1^⑤^ | - | -1^⑤^ |
| Time of absence from school, days | Risk of bias | -1^①^ | -1^①^ | - | -1^①^ |
|  | Inconsistency | -1^②^ | 0 | -1^②^ | -1^②^ |
|  | Indirectness | 0 | 0 | - | 0 |
|  | Impression | 0 | -1^④^ | 0 | 0 |
|  | Publication bias | -1^⑤^ | 1^⑤^ | - | 1^⑤^ |
| SAE incidence | Risk of bias | 0 | -1^①^ | -1^①^ | -1^①^ |
|  | Inconsistency | 0 | 0 | - | 0 |
|  | Indirectness | 0 | 0 | - | 0 |
|  | Impression | 0 | -1^④^ | 0 | 0 |
|  | Publication bias | -1^⑤^ | -1^⑤^ | - | -1^⑤^ |
| PAQLQ score | Risk of bias | -1^①^ | -1^①^ | - | -1^①^ |
|  | Inconsistency | -1^②^ | -1^②^ | - | -1^②^ |
|  | Indirectness | 0 | 0 | - | 0 |
|  | Impression | 0 | -1^④^ | 1^④^ | 1^④^ |
|  | Publication bias | -1^⑤^ | -1^⑤^ | - | -1^⑤^ |

12.王亚芹,樊鹏利,吕品,陈博雅,李坤,马培志.奥马珠单抗治疗儿童及青少年过敏性哮喘的系统评价[J].中国药物评价,2021,38(2):111-114

| Endpoint measure | Downgrading factor | A | B | C | D |
| --- | --- | --- | --- | --- | --- |
| Rate of asthma exacerbation | Risk of bias | -1^①^ | -1^①^ | - | -1^①^ |
|  | Inconsistency | 0 | 0 | - | 0 |
|  | Indirectness | 0 | 0 | - | 0 |
|  | Impression | 0 | 0 | - | 0 |
|  | Publication bias | -1^⑤^ | -1^⑤^ | - | -1^⑤^ |
| Asthma treatment effect rating | Risk of bias | -1^①^ | 0 | - | 0 |
|  | Inconsistency | 0 | 0 | - | 0 |
|  | Indirectness | 0 | 0 | - | 0 |
|  | Impression | 0 | 0 | - | 0 |
|  | Publication bias | -1^⑤^ | 0 | - | 0 |
| Adverse event rate | Risk of bias | -1^①^ | -1^①^ | - | -1^①^ |
|  | Inconsistency | -1^②^ | -1^②^ | - | -1^②^ |
|  | Indirectness | 0 | 0 | - | 0 |
|  | Impression | -1^④^ | 0 | -1^④^ | -1^④^ |
|  | Publication bias | -1^⑤^ | 0 | -1^⑤^ | -1^⑤^ |
| Serious adverse event rate | Risk of bias | -1^①^ | -1^①^ | - | -1^①^ |
|  | Inconsistency | -1^②^ | 0 | 0 | 0 |
|  | Indirectness | 0 | 0 | - | 0 |
|  | Impression | -1^④^ | 0 | -1^④^ | -1^④^ |
|  | Publication bias | -1^⑤^ | 0 | -1^⑤^ | -1^⑤^ |

13.陈雪琴,贾心予,吴晶晶,等. 奥马珠单抗治疗难治性过敏性哮喘疗效和安全性的荟萃分析[J]. 中华医学杂志,2022,102(28):2201-2209. DOI:10.3760/cma.j.cn112137-20211109-02480.

| Endpoint measure | Downgrading factor | A | B | C | D |
| --- | --- | --- | --- | --- | --- |
| Asthma exacerbations(stable steroid phase) | Risk of bias | -1^①^ | 0 | 0 | 0 |
|  | Inconsistency | 0 | 0 | - | 0 |
|  | Indirectness | 0 | 0 | - | 0 |
|  | Impression | 0 | 0 | - | 0 |
|  | Publication bias | 0 | 0 | - | 0 |
| Asthma exacerbations(steroid reduction phase) | Risk of bias | -1^①^ | 0 | 0 | 0 |
|  | Inconsistency | 0 | 0 | - | 0 |
|  | Indirectness | 0 | 0 | - | 0 |
|  | Impression | 0 | 0 | - | 0 |
|  | Publication bias | 0 | 0 | - | 0 |
| Emergency visits | Risk of bias | -1^①^ | 0 | 0 | 0 |
|  | Inconsistency | 0 | 0 | - | 0 |
|  | Indirectness | 0 | 0 | - | 0 |
|  | Impression | 0 | 0 | - | 0 |
|  | Publication bias | 0 | 0 | - | 0 |
| ICS reduction >50% | Risk of bias | -1^①^ | 0 | 0 | 0 |
|  | Inconsistency | 0 | 0 | - | 0 |
|  | Indirectness | 0 | 0 | - | 0 |
|  | Impression | 0 | 0 | - | 0 |
|  | Publication bias | 0 | 0 | - | 0 |
| ICS complete withdrawal | Risk of bias | -1^①^ | 0 | 0 | 0 |
|  | Inconsistency | -1^②^ | -1^②^ | - | -1^②^ |
|  | Indirectness | 0 | 0 | - | 0 |
|  | Impression | 0 | 0 | - | 0 |
|  | Publication bias | 0 | 0 | - | 0 |
| AQLQ score improvement >1.5 points | Risk of bias | 0 | 0 | - | 0 |
|  | Inconsistency | 0 | 0 | - | 0 |
|  | Indirectness | 0 | 0 | - | 0 |
|  | Impression | 0 | 0 | - | 0 |
|  | Publication bias | 0 | 0 | - | 0 |
| AQLQ score improvement >0.5 points | Risk of bias | 0 | 0 | 0 | 0 |
|  | Inconsistency | -1^②^ | -1^②^ | - | -1^②^ |
|  | Indirectness | 0 | 0 | - | 0 |
|  | Impression | 0 | 0 | - | 0 |
|  | Publication bias | 0 | 0 | - | 0 |
| Use of Rescue Bronchodilators | Risk of bias | -1^①^ | 0 | -1^①^ | -1^①^ |
|  | Inconsistency | 0 | 0 | - | 0 |
|  | Indirectness | 0 | 0 | - | 0 |
|  | Impression | 0 | 0 | - | 0 |
|  | Publication bias | 0 | 0 | - | 0 |
| iGETE rated as "excellent" or "good" | Risk of bias | -1^①^ | 0 | -1^①^ | -1^①^ |
|  | Inconsistency | -1^②^ | -1^②^ | - | -1^②^ |
|  | Indirectness | 0 | 0 | - | 0 |
|  | Impression | 0 | 0 | - | 0 |
|  | Publication bias | 0 | 0 | - | 0 |
| Adverse events | Risk of bias | -1^①^ | 0 | -1^①^ | -1^①^ |
|  | Inconsistency | -1^②^ | 0 | 0 | 0 |
|  | Indirectness | 0 | 0 | - | 0 |
|  | Impression | 0 | 0 | - | 0 |
|  | Publication bias | 0 | 0 | - | 0 |

14.Fenu G, La Tessa A, Calogero C, Lombardi E. Severe pediatric asthma therapy: Omalizumab-A systematic review and meta-analysis of efficacy and safety profile. Front Pediatr. 2023 Mar 3;10:1033511. doi: 10.3389/fped.2022.1033511. PMID: 36937051; PMCID: PMC10020639.

| Endpoint measure | Downgrading factor | A | B | C | D |
| --- | --- | --- | --- | --- | --- |
| Asthma exacerbation rate | Risk of bias | -1^①^ | 0 | 0 | 0 |
|  | Inconsistency | 0 | 0 | - | 0 |
|  | Indirectness | 0 | 0 | - | 0 |
|  | Impression | 0 | 0 | - | 0 |
|  | Publication bias | 0 | 0 | - | 0 |
| Reduction in inhaled corticosteroid (ICS) use | Risk of bias | -1^①^ | 0 | -1^①^ | -1^①^ |
|  | Inconsistency | 0 | 0 | - | 0 |
|  | Indirectness | 0 | 0 | - | 0 |
|  | Impression | 0 | 0 | - | 0 |
|  | Publication bias | 0 | 0 | - | 0 |
| Improvement in lung function | Risk of bias | -1^①^ | 0 | 0 | 0 |
|  | Inconsistency | 0 | 0 | -1^②^ | -1^②^ |
|  | Indirectness | 0 | 0 | - | 0 |
|  | Impression | -1^④^ | -1^④^ | - | -1^④^ |
|  | Publication bias | 0 | 0 | - | 0 |
| Drug-related adverse events | Risk of bias | -1^①^ | 0 | -1^①^ | -1^①^ |
|  | Inconsistency | 0 | -1^②^ | 1^②^ | 1^②^ |
|  | Indirectness | 0 | 0 | - | 0 |
|  | Impression | -1^④^ | 0 | -1^④^ | -1^④^ |
|  | Publication bias | 0 | 0 | - | 0 |

15.Lang D, Liu Z, Li D. Safety and Tolerability of Omalizumab in Children with Allergic (IgE-Mediated) Asthma: A Systematic Review and Meta-Analysis. Discov Med. 2023 Jun;35(176):233-241. doi: 10.24976/Discov.Med.202335176.24. PMID: 37272090.

| Endpoint measure | Downgrading factor | A | B | C | D |
| --- | --- | --- | --- | --- | --- |
| GETE response rate | Risk of bias | -1^①^ | 0 | -1^①^ | -1^①^ |
|  | Inconsistency | -1^②^ | -1^②^ | - | -1^②^ |
|  | Indirectness | 0 | 0 | - | 0 |
|  | Impression | 0 | 0 | - | 0 |
|  | Publication bias | 0 | 0 | - | 0 |
| Incidence of significant clinical exacerbation within 24 weeks | Risk of bias | -1^①^ | 0 | -1^①^ | -1^①^ |
|  | Inconsistency | -1^②^ | -1^②^ | - | -1^②^ |
|  | Indirectness | 0 | 0 | - | 0 |
|  | Impression | 0 | 0 | - | 0 |
|  | Publication bias | 0 | 0 | - | 0 |
| Incidence of significant clinical exacerbation within 52 weeks | Risk of bias | -1^①^ | 0 | -1^①^ | -1^①^ |
|  | Inconsistency | -1^①^ | -1^②^ | - | -1^②^ |
|  | Indirectness | 0 | 0 | - | 0 |
|  | Impression | 0 | 0 | - | 0 |
|  | Publication bias | 0 | 0 | - | 0 |
| Total incidence of adverse reactions | Risk of bias | -1^①^ | 0 | 0 | 0 |
|  | Inconsistency | 0 | 0 | - | 0 |
|  | Indirectness | 0 | 0 | - | 0 |
|  | Impression | 0 | 0 | - | 0 |
|  | Publication bias | 0 | 0 | - | 0 |
| Incidence of serious adverse reactions | Risk of bias | -1^①^ | 0 | 0 | 0 |
|  | Inconsistency | 0 | 0 | - | 0 |
|  | Indirectness | 0 | 0 | - | 0 |
|  | Impression | 0 | 0 | - | 0 |
|  | Publication bias | 0 | 0 | - | 0 |

16.阮俊文.抗IgE单克隆抗体在支气管哮喘患者升阶梯治疗中有效性和安全性的系统评价与Meta分析[D].赣南医学院,2023.DOI:10.27959/d.cnki.ggnyx.2023.000137.

| Endpoint measure | Downgrading factor | A | B | C | D |
| --- | --- | --- | --- | --- | --- |
| Total clinical effective rate | Risk of bias | -1^①^ | 0 | -1^①^ | -1^①^ |
|  | Inconsistency | -1^②^ | -1^②^ | - | -1^②^ |
|  | Indirectness | 0 | 0 | - | 0 |
|  | Impression | 0 | 0 | - | 0 |
|  | Publication bias | 0 | 0 | - | 0 |
| Asthma control test (ACT) | Risk of bias | 0 | 0 | - | 0 |
|  | Inconsistency | 0 | 0 | - | 0 |
|  | Indirectness | 0 | 0 | - | 0 |
|  | Impression | 0 | 0 | - | 0 |
|  | Publication bias | 0 | 0 | - | 0 |
| Asthma control questionnaire (ACQ) | Risk of bias | -1^①^ | -1^①^ | - | -1^①^ |
|  | Inconsistency | -1^②^ | -1^②^ | - | -1^②^ |
|  | Indirectness | 0 | 0 | - | 0 |
|  | Impression | -1^④^ | -1^④^ | - | -1^④^ |
|  | Publication bias | -1^⑤^ | 0 | 0 | 0 |
| Asthma quality of life questionnaire (AQLQ) | Risk of bias | -1^①^ | -1^①^ | - | -1^①^ |
|  | Inconsistency | -1^②^ | -1^②^ | - | -1^②^ |
|  | Indirectness | 0 | 0 | - | 0 |
|  | Impression | -1^④^ | -1^④^ | - | -1^④^ |
|  | Publication bias | -1^⑤^ | 0 | -1^⑤^ | -1^⑤^ |
| Immunoglobulin E (IgE) | Risk of bias | -1^①^ | 0 | -1^①^ | -1^①^ |
|  | Inconsistency | -1^②^ | -1^②^ | - | -1^②^ |
|  | Indirectness | 0 | 0 | - | 0 |
|  | Impression | 0 | -1^④^ | -1^④^ | -1^④^ |
|  | Publication bias | 0 | 0 | - | 0 |
| Fractional concentration of exhaled nitric oxide (FeNO) | Risk of bias | -1^①^ | 0 | -1^①^ | -1^①^ |
|  | Inconsistency | -1^②^ | -1^②^ | - | -1^②^ |
|  | Indirectness | 0 | 0 | - | 0 |
|  | Impression | -1^④^ | -1^④^ | - | -1^④^ |
|  | Publication bias | 0 | 0 | - | 0 |
| Forced expiratory volume in 1s (FEV1) increased value | Risk of bias | -1^①^ | 0 | -1^①^ | -1^①^ |
|  | Inconsistency | -1^②^ | -1^②^ | - | -1^②^ |
|  | Indirectness | 0 | 0 | - | 0 |
|  | Impression | 0 | 0 | - | 0 |
|  | Publication bias | 0 | 0 | - | 0 |
| FEV1% predicted value (FEV1%Pred) increased value | Risk of bias | -1^①^ | -1^①^ | -1^①^ | -1^①^ |
|  | Inconsistency | -1^②^ | -1^②^ | - | -1^②^ |
|  | Indirectness | 0 | 0 | - | 0 |
|  | Impression | 0 | -1^④^ | -1^④^ | -1^④^ |
|  | Publication bias | 0 | 0 | - | 0 |
| Number of acute exacerbation of asthma | Risk of bias | 0 | 0 | - | 0 |
|  | Inconsistency | 0 | 0 | - | 0 |
|  | Indirectness | 0 | 0 | - | 0 |
|  | Impression | 0 | 0 | - | 0 |
|  | Publication bias | 0 | 0 | - | 0 |
| Frequency of acute exacerbation of asthma | Risk of bias | 0 | 0 | - | 0 |
|  | Inconsistency | 0 | 0 | - | 0 |
|  | Indirectness | 0 | 0 | - | 0 |
|  | Impression | -1^④^ | 0 | -1^④^ | -1^④^ |
|  | Publication bias | 0 | 0 | - | 0 |
| Adverse reaction (AR) | Risk of bias | 0 | 0 | - | 0 |
|  | Inconsistency | 0 | 0 | - | 0 |
|  | Indirectness | 0 | 0 | - | 0 |
|  | Impression | 0 | 0 | - | 0 |
|  | Publication bias | 0 | 0 | - | 0 |

17.薛宽宽.奥马珠单抗治疗中重度过敏性哮喘效果的Meta分析[D].山西医科大学,2023.DOI:10.27288/d.cnki.gsxyu.2023.000328.

| Endpoint measure | Downgrading factor | A | B | C | D |
| --- | --- | --- | --- | --- | --- |
| The proportion of patients with at least one acute asthma attack | Risk of bias | -1^①^ | -1^①^ | - | -1^①^ |
|  | Inconsistency | -1^②^ | -1^②^ | - | -1^②^ |
|  | Indirectness | 0 | 0 | - | 0 |
|  | Impression | 0 | 0 | - | 0 |
|  | Publication bias | 0 | 0 | - | 0 |
| Frequency of acute asthma attacks in patients | Risk of bias | -1^①^ | -1^①^ | - | -1^①^ |
|  | Inconsistency | -1^②^ | -1^②^ | - | -1^②^ |
|  | Indirectness | 0 | 0 | - | 0 |
|  | Impression | 0 | 0 | - | 0 |
|  | Publication bias | 0 | 0 | - | 0 |
| Change in total AQLQ score from baseline during the study period | Risk of bias | -1^①^ | 0 | -1^①^ | -1^①^ |
|  | Inconsistency | 0 | 0 | - | 0 |
|  | Indirectness | 0 | 0 | - | 0 |
|  | Impression | 0 | 0 | - | 0 |
|  | Publication bias | 0 | 0 | - | 0 |
| Incidence of adverse reactions in patients during the study period | Risk of bias | 0 | -1^①^ | -1^①^ | -1^①^ |
|  | Inconsistency | 0 | 0 | - | 0 |
|  | Indirectness | 0 | 0 | - | 0 |
|  | Impression | 0 | 0 | - | 0 |
|  | Publication bias | 0 | 0 | - | 0 |
| Incidence of severe adverse reactions in patients during the study period | Risk of bias | 0 | -1^①^ | -1^①^ | -1^①^ |
|  | Inconsistency | 0 | 0 | - | 0 |
|  | Indirectness | 0 | 0 | - | 0 |
|  | Impression | 0 | 0 | - | 0 |
|  | Publication bias | 0 | 0 | - | 0 |

18.廖浚邑.奥马珠单抗治疗对中重度过敏性哮喘患者肺功能改善的Meta分析[D].重庆医科大学,2024.DOI:10.27674/d.cnki.gcyku.2024.000768.

| Endpoint measure | Downgrading factor | A | B | C | D |
| --- | --- | --- | --- | --- | --- |
| FEV1% | Risk of bias | -1^①^ | -1^①^ | - | -1^①^ |
|  | Inconsistency | -1^②^ | -1^②^ | - | -1^②^ |
|  | Indirectness | 0 | 0 | - | 0 |
|  | Impression | 0 | 0 | - | 0 |
|  | Publication bias | -1^⑤^ | -1^⑤^ | - | -1^⑤^ |
| mPEF | Risk of bias | -1^①^ | -1^①^ | - | -1^①^ |
|  | Inconsistency | -1^②^ | -1^②^ | - | -1^②^ |
|  | Indirectness | 0 | 0 | - | 0 |
|  | Impression | 0 | -1^④^ | -1^④^ | -1^④^ |
|  | Publication bias | -1^⑤^ | -1^⑤^ | - | -1^⑤^ |
| FEV1 | Risk of bias | -1^①^ | -1^①^ | - | -1^①^ |
|  | Inconsistency | 0 | 0 | - | 0 |
|  | Indirectness | 0 | 0 | - | 0 |
|  | Impression | 0 | 0 | - | 0 |
|  | Publication bias | -1^⑤^ | -1^⑤^ | - | -1^⑤^ |

19.刘香.奥马珠单抗治疗儿童中重度过敏性哮喘有效性及安全性Meta分析[D].南昌大学,2024.DOI:10.27232/d.cnki.gnchu.2024.004079.

| Endpoint measure | Downgrading factor | A | B | C | D |
| --- | --- | --- | --- | --- | --- |
| Overall effective rate of treatment | Risk of bias | -1^①^ | -1^①^ | - | -1^①^ |
|  | Inconsistency | -1^②^ | 0 | -1^②^ | -1^②^ |
|  | Indirectness | 0 | 0 | - | 0 |
|  | Impression | 0 | 0 | - | 0 |
|  | Publication bias | -1^⑤^ | -1^⑤^ | - | -1^⑤^ |
| Asthma control score | Risk of bias | 0 | 0 | - | 0 |
|  | Inconsistency | 0 | 0 | - | 0 |
|  | Indirectness | 0 | 0 | - | 0 |
|  | Impression | 0 | 0 | - | 0 |
|  | Publication bias | -1^⑤^ | -1^⑤^ | - | -1^⑤^ |
| Acute asthma attack rate | Risk of bias | 0 | 0 | - | 0 |
|  | Inconsistency | 0 | 0 | - | 0 |
|  | Indirectness | 0 | 0 | - | 0 |
|  | Impression | 0 | 0 | - | 0 |
|  | Publication bias | -1^⑤^ | -1^⑤^ | - | -1^⑤^ |
| FEV1 | Risk of bias | -1^①^ | -1^①^ | - | -1^①^ |
|  | Inconsistency | -1^②^ | -1^②^ | - | -1^②^ |
|  | Indirectness | 0 | 0 | - | 0 |
|  | Impression | 0 | 0 | - | 0 |
|  | Publication bias | -1^⑤^ | -1^⑤^ | - | -1^⑤^ |
| FEV1/FVC | Risk of bias | -1^①^ | -1^①^ | - | -1^①^ |
|  | Inconsistency | -1^②^ | -1^②^ | - | -1^②^ |
|  | Indirectness | 0 | 0 | - | 0 |
|  | Impression | -1^④^ | -1^④^ | - | -1^④^ |
|  | Publication bias | -1^⑤^ | -1^⑤^ | - | -1^⑤^ |
| IgE | Risk of bias | -1^①^ | 0 | -1^①^ | -1^①^ |
|  | Inconsistency | -1^②^ | -1^②^ | - | -1^②^ |
|  | Indirectness | 0 | 0 | - | 0 |
|  | Impression | 0 | 0 | - | 0 |
|  | Publication bias | -1^⑤^ | -1^⑤^ | - | -1^⑤^ |
| CD3 | Risk of bias | -1^①^ | 0 | -1^①^ | -1^①^ |
|  | Inconsistency | 0 | 0 | - | 0 |
|  | Indirectness | 0 | 0 | - | 0 |
|  | Impression | -1^④^ | 0 | -1^④^ | -1^④^ |
|  | Publication bias | -1^⑤^ | -1^⑤^ | - | -1^⑤^ |
| CD4 | Risk of bias | -1^①^ | 0 | -1^①^ | -1^①^ |
|  | Inconsistency | 0 | 0 | - | 0 |
|  | Indirectness | 0 | 0 | - | 0 |
|  | Impression | -1^④^ | 0 | -1^④^ | -1^④^ |
|  | Publication bias | -1^⑤^ | -1^⑤^ | - | -1^⑤^ |
| IgA | Risk of bias | -1^①^ | -1^①^ | - | -1^①^ |
|  | Inconsistency | 0 | 0 | - | 0 |
|  | Indirectness | 0 | 0 | - | 0 |
|  | Impression | -1^④^ | -1^④^ | - | -1^④^ |
|  | Publication bias | -1^⑤^ | -1^⑤^ | - | -1^⑤^ |
| IgG | Risk of bias | -1^①^ | -1^①^ | - | -1^①^ |
|  | Inconsistency | 0 | 0 | - | 0 |
|  | Indirectness | 0 | 0 | - | 0 |
|  | Impression | -1^④^ | -1^④^ | - | -1^④^ |
|  | Publication bias | -1^⑤^ | -1^⑤^ | - | -1^⑤^ |
| Incidence of adverse reactions | Risk of bias | 0 | 0 | - | 0 |
|  | Inconsistency | 0 | 0 | - | 0 |
|  | Indirectness | 0 | 0 | - | 0 |
|  | Impression | 0 | 0 | - | 0 |
|  | Publication bias | -1^⑤^ | -1^⑤^ | - | -1^⑤^ |
| Incidence of serious adverse reactions | Risk of bias | 0 | 0 | - | 0 |
|  | Inconsistency | 0 | 0 | - | 0 |
|  | Indirectness | 0 | 0 | - | 0 |
|  | Impression | 0 | 0 | - | 0 |
|  | Publication bias | -1^⑤^ | -1^⑤^ | - | -1^⑤^ |
